# The organelle genomes of the endangered seagrass *Zostera caespitosa* reveal sequence divergences, massive gene transfer, and uncommon RNA editing types

**DOI:** 10.3389/fpls.2025.1550467

**Published:** 2025-02-17

**Authors:** Yushun Yong, Yulian Wang, Dawei Wang, Xingfang Yuan, Quansheng Zhang

**Affiliations:** ^1^ Ocean School, Yantai University, Yantai, China; ^2^ No. 6 Geological Team, Shandong Provincial Bureau of Geology and Mineral Resources, Weihai, China

**Keywords:** *Zostera caespitosa*, mitochondrial genome, chloroplast genome, sequence evolution, gene transfer, RNA editing

## Abstract

**Introduction:**

*Zostera caespitosa*, a rare submerged angiosperm, is considered endemic to the northwestern Pacific.

**Methods:**

This study assembled and compared the mitochondrial (mt) and chloroplast (cp) genomes of *Z. caespitosa* to understand the organelle evolutionary patterns.

**Results and discussion:**

The cp genome (143,972 bp) was the second smallest within the seagrasses, whereas the mt genomes (192,246 bp) of *Z. caespitosa* and other seagrasses were smaller compared to those of other monocotyledons. The protein-coding genes (PCGs) in the organelle genome exhibit a strong A/U bias at codon endings, a selection-driven codon bias. The rates of nonsynonymous (Ka) and synonymous (Ks) substitutions in the mt genes of *Zostera* were two times higher than those in the cp genes. Additionally, 50 mitochondrial plastid DNA (MTPT) segments, totaling 44,662 bp, were identified, constituting 23.23% of the mt genome, which is significantly higher than those in most land plants. Phylogenetic analysis of 13 seagrass core cp-PCGs supported previous studies showing two genera in family Zosteraceae: *Phyllospadix* and *Zostera*, the latter comprising *Zostera* and *Zosterella* as subgenera. RNA editing was remarkably abundant in the 167 mt-PCGs and 172 in cp-PCGs, particularly in the cp genome. There are 11 different RNA editing types in the cp and 3 in the mt, most of which are C to U. Unexpectedly rare editing events, such as A to C, A to U, U to A, G to C, and U to G, have also been found in the cp.

## 1 Introduction

*Zostera caespitosa* is a submerged perennial herb belonging to the class Monocotyledoneae, order Alismatales, family Zosteraceae, and genus *Zostera*. The distribution of *Z. caespitosa* is local, with only a few populations found along the east coast of Korea, the southern coast of Japan, and the coastal areas of Liaoning and Shandong in China (Short et al., 2007; Xu et al., 2021)*. Z. caespitosa* is among the typical representative species in seagrass beds, playing a vital role in erosion protection, bacterial suppression, nutrient cycling, and significantly regulating carbon sequestration (Fourqurean et al., 2012; Lamb et al., 2017; Unsworth et al., 2022). However, the increasing pressures from human activities, such as ocean warming, coastal modification, and water quality degradation, are causing global seagrass loss (Orth et al., 2006; Waycott et al., 2009). This marine plant species was listed as “endangered” on the IUCN and China Biodiversity - Higher Plants Volume in 2021 and 2022 Red Lists, respectively (Short et al., 2011; Jiang et al., 2024). Previous studies on *Z. caespitosa* have mainly focused on its distribution and biological traits (Short et al., 2007; Xu et al., 2021; Jiang et al., 2024; Im et al., 2024). Nevertheless, it lacks basic genetic resources, limiting further research and conservation efforts.

Mitochondrial (mt) and chloroplast (cp) are endosymbiotic organelles with independent genetic material separate from those in the cell nucleus (Birky, 2001). These organelles share common traits, including replication methods, mutation patterns, and inheritance mechanisms and are significantly different in land plants (Sloan et al., 2012; Maréchal and Brisson, 2010; Birky, 1995). Plant mt genomes are typically larger and more complex, with a wide range of genome sizes (ca. 100–10,000 kb), varied structures, low density of genes, and numerous repetitive sequences (Jansen and Ruhlman, 2012), making their conformation challenging (Gualberto et al., 2014). In contrast, cp genomes of angiosperms exhibit a relatively simple structure, typically consisting of approximately 120–130 genes and a smaller size (~107–218 kb) (Daniell et al., 2016). These genomes comprise a conserved four-part structure with large single copy (LSC), small single copy (SSC), and inverted repeats (IRs) region (Henry, 2005; Jansen and Ruhlman, 2012). Thus, they are ideal systems for investigating the phylogenetic relationships among different plant species (Mower and Vickrey, 2018; Nizam et al., 2022). In addition, the RNA editing process was extremely abundant, diverse, and remarkably complex in organellar RNAs (Kaur, 2020; Takenaka et al., 2013). To date, no research has been conducted on identifying RNA editing events through RNA-Seq read mapping in *Z. caespitosa*.

Recent developments in sequencing technologies have significantly increased the number of plant cp and mt genomes. Currently, the NCBI database contains approximately 13,000 cp genomes and 673 mt genomes, yet only 285 species have both genomes assembled (Wang et al., 2024). Although various evolutionary patterns have been proposed for these genomes, knowledge gaps persist, primarily due to unequal sampling. Seagrasses, comprising approximately 74 species, represent a crucial transformative event in higher plant evolution (Olsen et al., 2016). Unfortunately, complete organelle genomes have been published for only five seagrass species: *Z. marina*, *Z. japonica*, *Phyllospadix iwatensis*, *Ruppia sinensis*, and *Cymodocea nodosa* (Petersen et al., 2017; Chen et al., 2021, 2022, 2023; Ma et al., 2024). In the Yellow–Bohai Sea, a temperate seagrass habitat in the North Pacific, five species from three genera (*Zostera*, *Phyllospadix*, and *Ruppia*) have currently been identified, namely, *Z. marina*, *Z. japonica*, *Z. caespitosa*, *P. iwatensis*, and *R. sinensis* (Xu et al., 2021). Recent research has extensively used mt and cp genomes to explore molecular evolution (Li et al., 2023; Huang et al., 2020). As a major member of the Yellow–Bohai Seas seagrass species, the differences between the mt and cp genomes of *Z. caespitosa* remain uncharacterized, limiting insights into its origin and adaptive evolution.

Thus, this study sequenced, assembled, and annotated the organelle genome of *Z. caespitosa*. Our objectives were to (1) describe the features of the organelle genomes; (2) analyze evolutionary differences in sequences (repeated elements, codon bias, mutation rate, and phylogenetic relationships); (3) identify horizontal gene transfer (HGT) events; and (4) explore the characteristics of RNA editing events.

## 2 Results

### 2.1 Composition of the *Z. caespitosa* organelle genome

**Figure 1:**
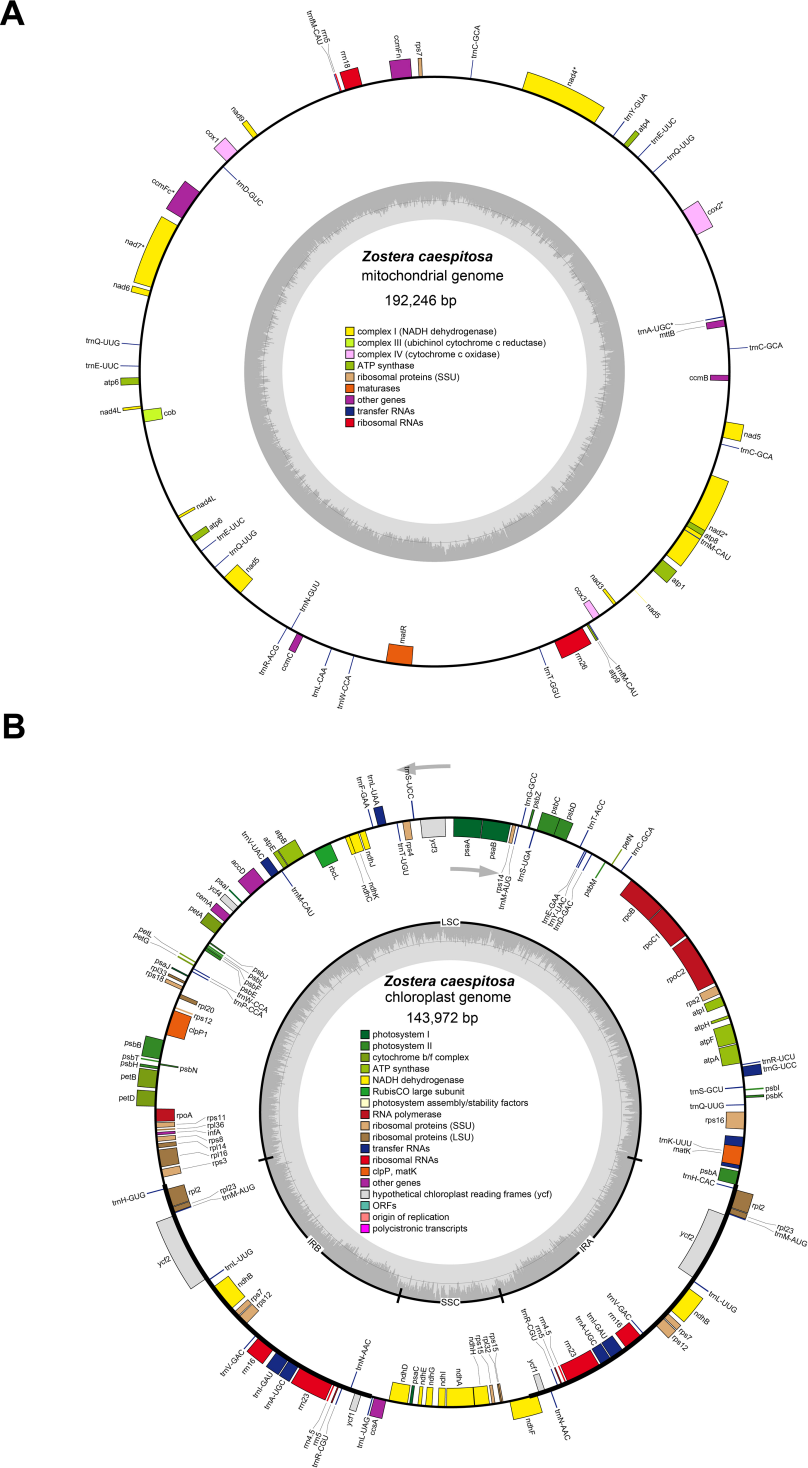
The organelle genomes map of *Z. caespitosa*. **(A ,B)** represent the mt and cpgenomes, respectively. The genes located inside the circles are transcribed in a clockwise direction, while those outside the circle are transcribed counterclockwise. Different colored genes represent different functions.

The complete circular mt genome of *Z. caespitosa* was 192,246 bp long with 45.63% in GC content (**Figure 1A**). Furthermore, the mt genome has 86.02%, 13.98%, 0.75%, and 2.71% intergenic regions, protein-coding genes (PCGs), tRNA, and rRNA, respectively (**Supplementary Table S1**). The mt genome encodes 50 genes, namely, 27 PCGs, 20 tRNA, and 3 rRNA (*rrn5*, *rrn18*, and *rrn26*) (**Table 1**). The PCGs comprised several genes, including five ATP synthase (*atp1*, *atp4*, *atp6*, *atp8*, and *atp9*), four cytochrome c biogenesis (*ccmB*, *ccmC*, *ccmFc*, and *ccmFn*), three cytochrome c oxidase (*cox1*, *cox2*, and *cox3*), and one ubiquinol cytochrome c reductase (*cob*). Additionally, PCGs have one maturase (*matR*), one membrane transport protein (*mttB*), nine NADH dehydrogenase *(nad1*, *nad2*, *nad3*, *nad4*, *nad4L*, *nad5*, *nad6*, *nad7*, and *nad9*), one ribosomal protein (SSU) (*rps7*), and two copies of *atp6* and *nad4L* genes. Gene *nad1*, *nad2*, *nad5*, and *nad7* possessed four introns, *nad4* possessed three introns, and *cox2* and *ccmFc* possessed one intron.

**Table 1:** List of genes encoded by the mt genome of *Z. caespitosa*.

Group of genes	Gene name
ATP synthase	*atp1*, *atp4*, *atp6*(×2), *atp8*, *atp9*
Cytohrome c biogenesis	*ccmB*, *ccmC*, *ccmFc^a^*, *ccmFn*
Ubichinol cytochrome c reductase	*cob*
Cytochrome c oxidase	*cox1*, *cox2^a^*, *cox3*
Maturases	*matR*
Transport membrane protein	*mttB*
NADH dehydrogenase	*nad1^d^*, *nad2^d^*, *nad3*, *nad4^c^*, *nad4L*(×2), *nad5^d^*, *nad6*, *nad7^d^*, *nad9*
Ribosomal proteins (LSU)	
Ribosomal proteins (SSU)	*rps7*
Succinate dehydrogenase	
Ribosomal RNAs	*rrn18*, *rrn26*, *rrn5*
Transfer RNAs	*trnA-UGC^a^*, *trnC-GCA*(×3), *trnD-GUC*, *trnE-UUC*(×3), *trnL-CAA*, *trnM-CAU*, *trnN-GUU*, *trnQ-UUG*(×3), *trnR-ACG*, *trnT-GGU*, *trnW-CCA*, *trnY-GUA*, *trnfM-CAU*(×2)

Gene^a^: Gene with one intron.

Gene^c^: Gene with three introns.

Gene^d^: Gene with four introns.

The complete cp genome of *Z. caespitosa* was 143,972 bp, and its typical structure includes an SSC of 12,405 bp, an LSC of 83,313 bp, and two IRs region, each 24,127 bp (**Figure 1B** and **Supplementary Table S1**). Further, a total of 131 genes were annotated in the *Z. caespitosa* cp genome, corresponding to 85 PCGs (counting genes in the inverted repeats twice), 38 rRNAs, and 8 tRNAs (**Table 2**). Seventeen genes had introns in the cp genome with two introns for three genes (*clpP1*, *rps12*, and *ycf3*) and one intron for the other 14 (*ndhA*, *ndhB*, *petB*, *petD*, *atpF*, *rpl16*, *rpl2*, *rpoC1*, *trnA-UGC*, *trnG-UUC*, *trnI-GAU*, *trnK-UUU*, *trnL-UAA*, and *trnA-UAC*).

**Table 2:** List of genes encoded by the cp genome of *Z. caespitosa*.

Category	Gene group	Gene name
Photosynthesis	Subunits of photosystem I	*psaA*, *psaB*, *psaC*, *psaI*, *psaJ*
	Subunits of photosystem II	*psbA*, *psbB*, *psbC*, *psbD*, *psbE*, *psbF*, *psbH*, *psbI*, *psbJ*, *psbK*, *psbL*, *psbT*, *psbZ*
	Subunits of NADH dehydrogenase	*ndhA^a^*, *ndhB^a^* (×2), *ndhC*, *ndhD*, *ndhE*, *ndhF*, *ndhG*, *ndhH*, *ndhI*, *ndhJ*, *ndhK*
	Subunits of cytochrome b/f complex	*petA*, *petB^a^*, *petD^a^*, *petG*, *petG*, *petL*, *petN*
	Subunits of ATP synthase	*atpA*, *atpB*, *atpE*, *atpF^a^*, *atpH*, *atpI*
	Large subunit of rubisco	*rbcL*
	Subunits photochlorophyllide reductase	–
Self-replication	Proteins of large ribosomal subunit	*rpl14*, *rpl16^a^*, *rpl2^a^* (×2), *rpl20*, *rpl22*, *rpl23*(×2), *rpl33*, *rpl36*
	Proteins of small ribosomal subunit	*rps11*, *rps12^b^* (×2), *rps14*, *rps15*(×2), *rps18*, *rps2*, *rps3*, *rps4*, *rps7*(×2), *rps8*
	Subunits of RNA polymerase	*rpoA*, *rpoB*, *rpoC1^a^*, *rpoC2*
	Ribosomal RNAs	*rrn16*(×2), *rrn23*(×2), *rrn4.5*(×2), *rrn5*(×2)
	Transfer RNAs	*trnA-UGC^a^* (×2), *trnC-GCA*, *trnD-GAC*, *trnE-GAA*, *trnF-GAA*, *trnG-GCC*, *trnG-UCC^a^*, *trnH-CAC*, *trnH-GUG*, *trnI-GAU^a^* (×2), *trnK-UUU ^a^*, *trnL-UAA^a^*, *trnL-UAG*, *trnL-UUG*(×2), *trnM-AUG*(×3), *trnM-CAU*, *trnN-AAC*(×2), *trnP-CCA*, *trnQ-UUG*, *trnR-CGU*(×2), *trnR-UCU*, *trnS-GCU*, *trnS-UCC*, *trnS-UGA*, *trnT-UGU*, *trnV-GAC*(×2), *trnV-UAC^a^*, *trnW-CCA*, *trnY-UAC*
Other genes	Maturase	*matK*
	Protease	*clpP1^b^*
	Envelope membrane protein	*cemA*
	Acetyl-CoA carboxylase	*accD*
	c-type cytochrome synthesis gene	*ccsA*
	Translation initiation factor	*infA*
	other	*-*
Genes of unknown function	Conserved hypothetical chloroplast ORF	*ycf1*(×2), *ycf2*(×2), *ycf3^b^*, *ycf4*

Gene^a^: Gene with one intron.

Gene^b^: Gene with two introns.

### 2.2 Repeat sequence analysis

**Figure 2:**
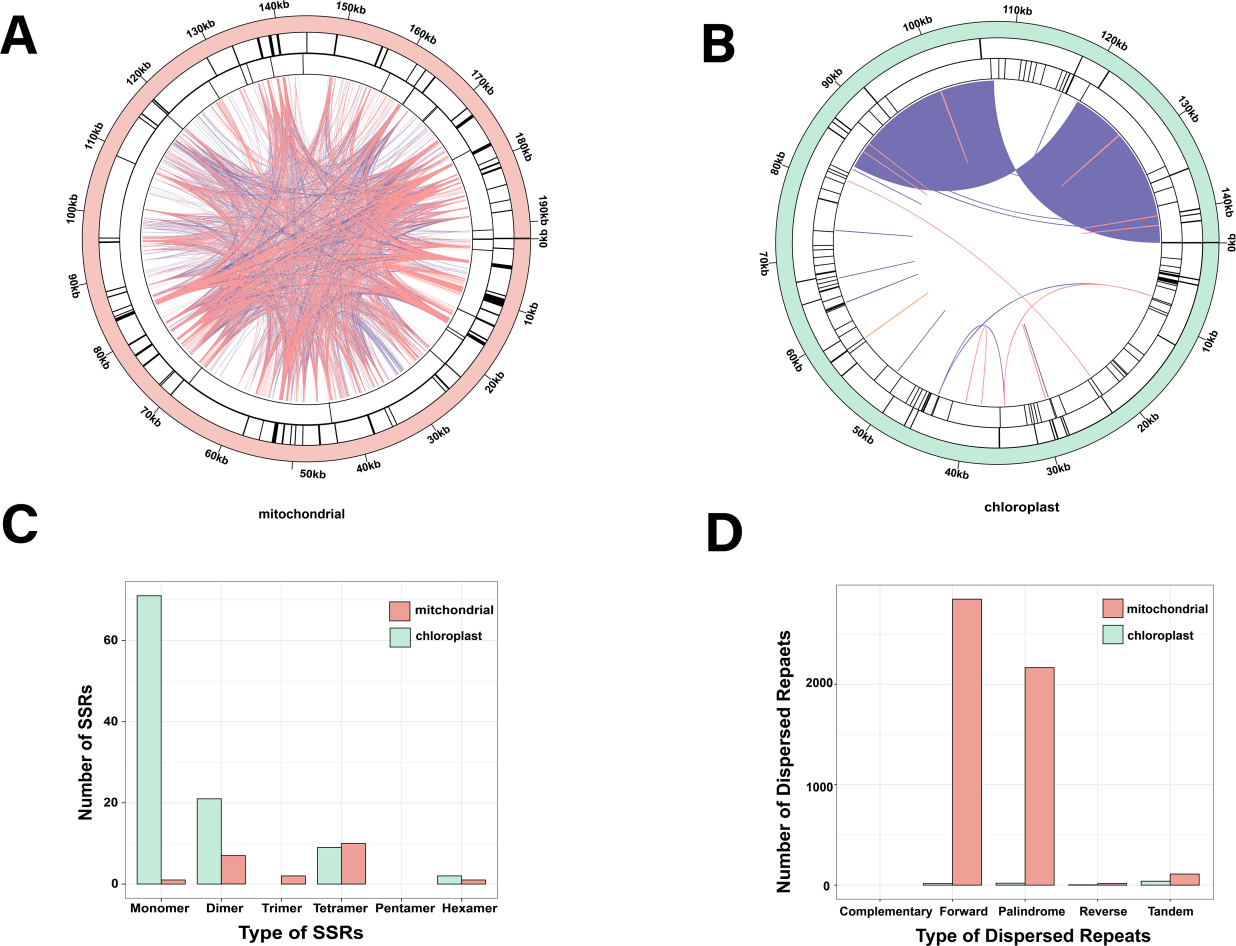
Repeat sequences within mt and cp genomes. Colored lines in the inner circle represent dispersed repeats: pink for forward, purple for palindromic, and orange for reverse repeats. The black line in the first circle marks tandem repeats, while the second circle highlights SSRs. **(A, B)** show the physical distribution of repeats in mt and cp genomes, while panels **(C, D)** illustrate the different types of dispersed repeats and SSRs.

A total of 21 and 103 microsatellites [simple repeat sequences (SSRs)] were identified in the mt and cp genomes, respectively, which were 1 to 6 bp DNA fragments (**Figure 2** and **Supplementary Table S2**). Motifs 1, 2, 3, 4, 5, and 6 were 1, 7, 2, 10, 0, and 1 mt repeats, respectively. In contrast, their cp repeats measured 71, 21, 0, 9, 0, and 2 bp, respectively (**Figure 2C**). Tetramer SSRs were the most dominant in the mt genome (47.6%), whereas monomer SSRs dominated the cp genome (68.9%). Furthermore, the mt and cp genomes had 110 and 39 tandem repeats, respectively, besides dispersed repeats that were also prevalent throughout these genomes (**Figure 2** and **Supplementary Table S3**). The mt and cp genomes had 5,028 and 37 pairs of dispersed repeats, respectively, depicting significantly higher dispersed repeats in the mt genome (**Figure 2** and **Supplementary Tables S4**, **S5**). There were 0 complementary, 2,847 forward, 2,165 palindromic, and 16 reverse repeats in the mt genome, while there were 0 complementary, 15 forward, 19 palindromic repeats, and 3 reverse repeats in the cp genome (**Figure 2D**). The organellar genomes lacked complementary repeats but had the abundant forward and palindromic repeats.

### 2.3 Codon bias analysis

**Figure 3:**
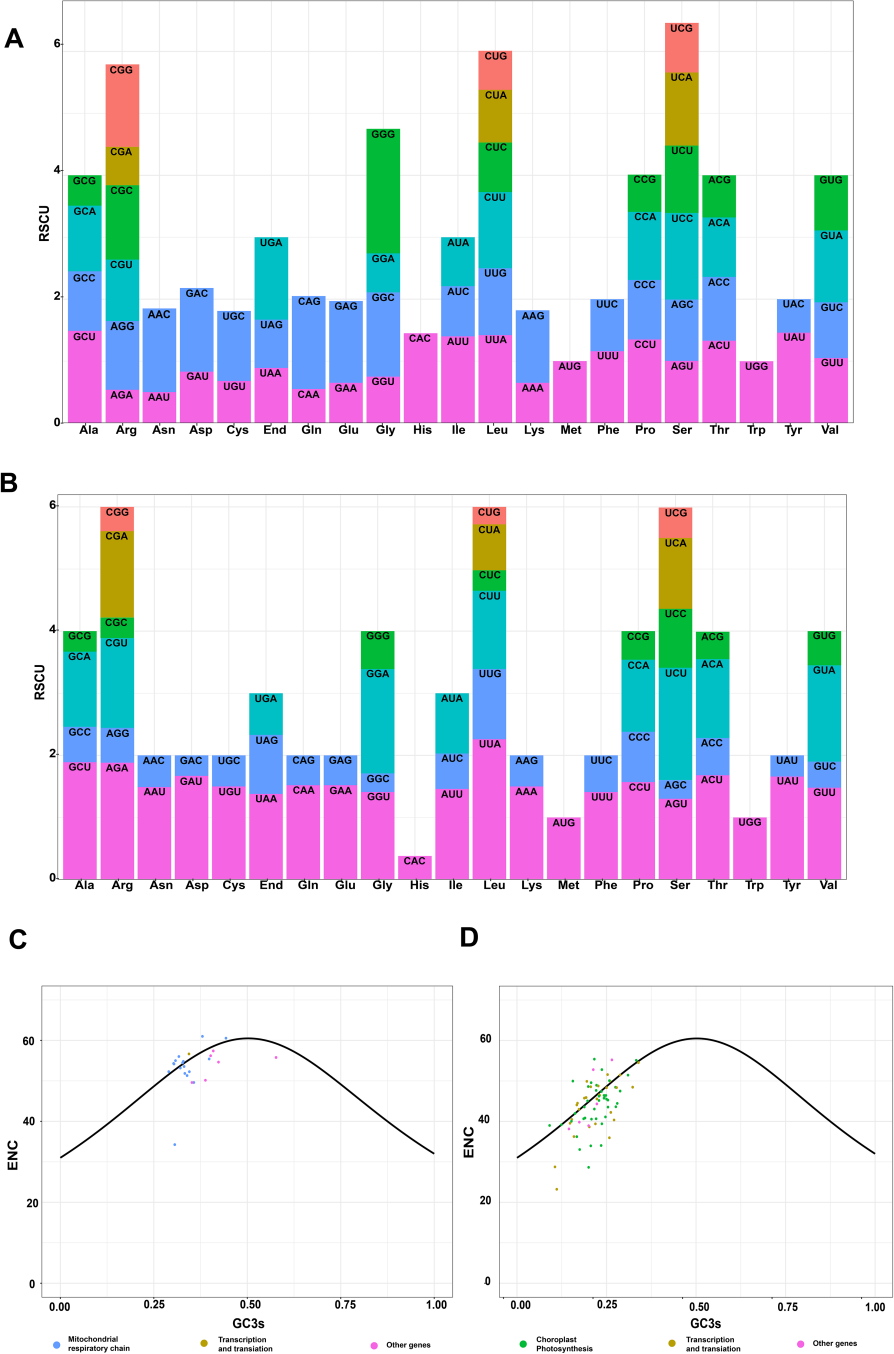
Summary of codon usage in PCGs of the *Z. caespitosa* organelle genome: **(A, B)** show RSCU distribution for mt and cp genomes, with horizontal axes representing 21 amino acids and vertical axes showing RSCU values. **(C, D)** plot ENC against GC3s for mt and cp genes, respectively. The solid line represents the expected trend based on GC3s composition. Blue dots indicate respiratory chain or photosynthesis genes, brown dots mark translation-related/transcription genes, and pink dots represent other genes.

The different organelles displayed a degree of variation in the codon usage frequency. The Relative Synonymous Codon Usage (RSCU) values in the mt genome were 0.49 (GCG in alanine) to 1.5 (CAA in glutamine) and 0.28 (CUG in leucine) to 2.26 (UUA in leucine) in the cp genome (**Figures 3A, B** and **Supplementary Table S6**). Only UGG (tryptophan) and AUG (start codon) had RSCU values 1 in the mt and cp genome; the rest were >1 or <1, indicating a general preference for codons in PCGs. The RSCU values of 30 codons in the mt and 29 codons in the cp genomes exceeded 1, indicating that these codons showed biased usage. The *Z. caespitosa* organellar genomes have a strong A/U preference in codon usage.

Effective codon numbers versus the third position GC content (ENC-GC3s) plots are commonly used to assess factors influencing codon usage patterns. The mt and cp genomes exhibited similar bias patterns, with most PCG genes deviating from the standard curve and a minority positioned on or close to it (**Figures 3C, D** and **Supplementary Table S7**). Respiration caused the remarkably pronounced deviations in mt, while photosynthesis primarily caused those in cp. The mt PCG gene *atp9* has ENC values of 33.25 (less than 35). In the cp genome, PCG genes with <35 ENC included *psbI* (33.96), *petN* (28.65), *psaI* (34.04), *psaJ* (33.05), *rpl36* (23.23), and *rpl32* (28.76).

### 2.4 Mutation rate analysis of *Zostera* species

**Figure 4:**
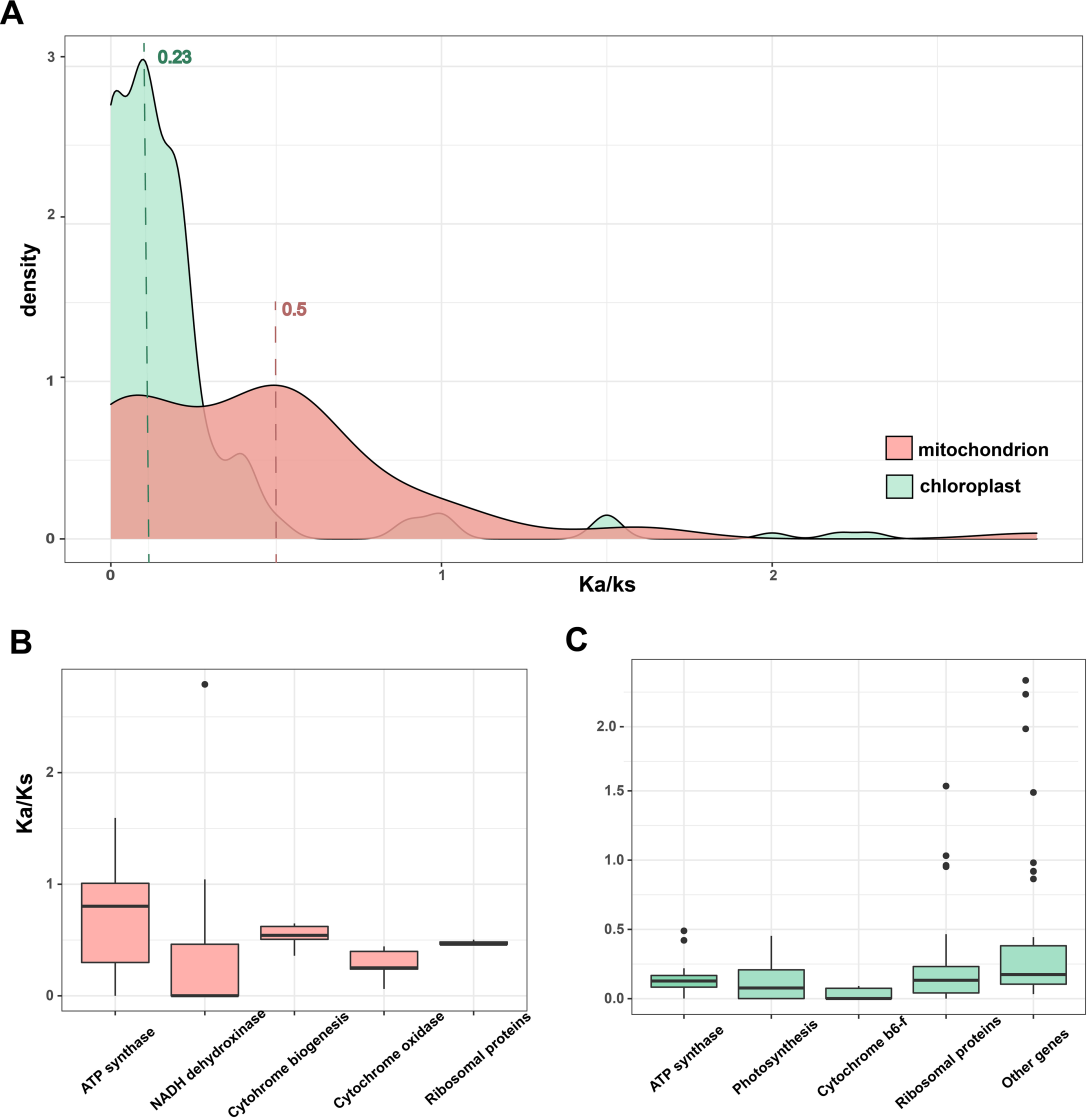
**(A)** The density distribution of Ka/Ks. **(B, C)** Boxplots show the median Ka/Ks for classes of mt and cp genes across the *Zostera* species.

To resolve the variations in evolutionary rates across the organelle genomes of *Zostera*, we examined the rates of evolution for the PCGs common to all *Zostera* species. The rates of nonsynonymous (Ka) and synonymous (Ks) substitutions of cp genome is roughly 1/2 of that of the mt genome, indicating that the cp genome probably suffered strong purifying selection and is more conserved at the level of PCGs (**Figure 4A**). The Ka/Ks value also reveals that the document rate overlaps between loci across the two organelles. Comparing the Ka/Ks of each gene from both organelles corroborated that mt genes evolve faster than cp (**Figures 4B,C**). Most genes from all major organellar classes are under strong purifying selection. ATP synthase genes had particularly the lowest Ka/Ks ratios in the cp genome, while they had the highest Ka/Ks ratios in the mt genome. In contrast to photosystem genes, ribosomal genes possibly suffered weaker purifying selection, with significantly greater Ka/Ks values from mt than cp genes.

### 2.5 Phylogenetic analysis

**Figure 5:**
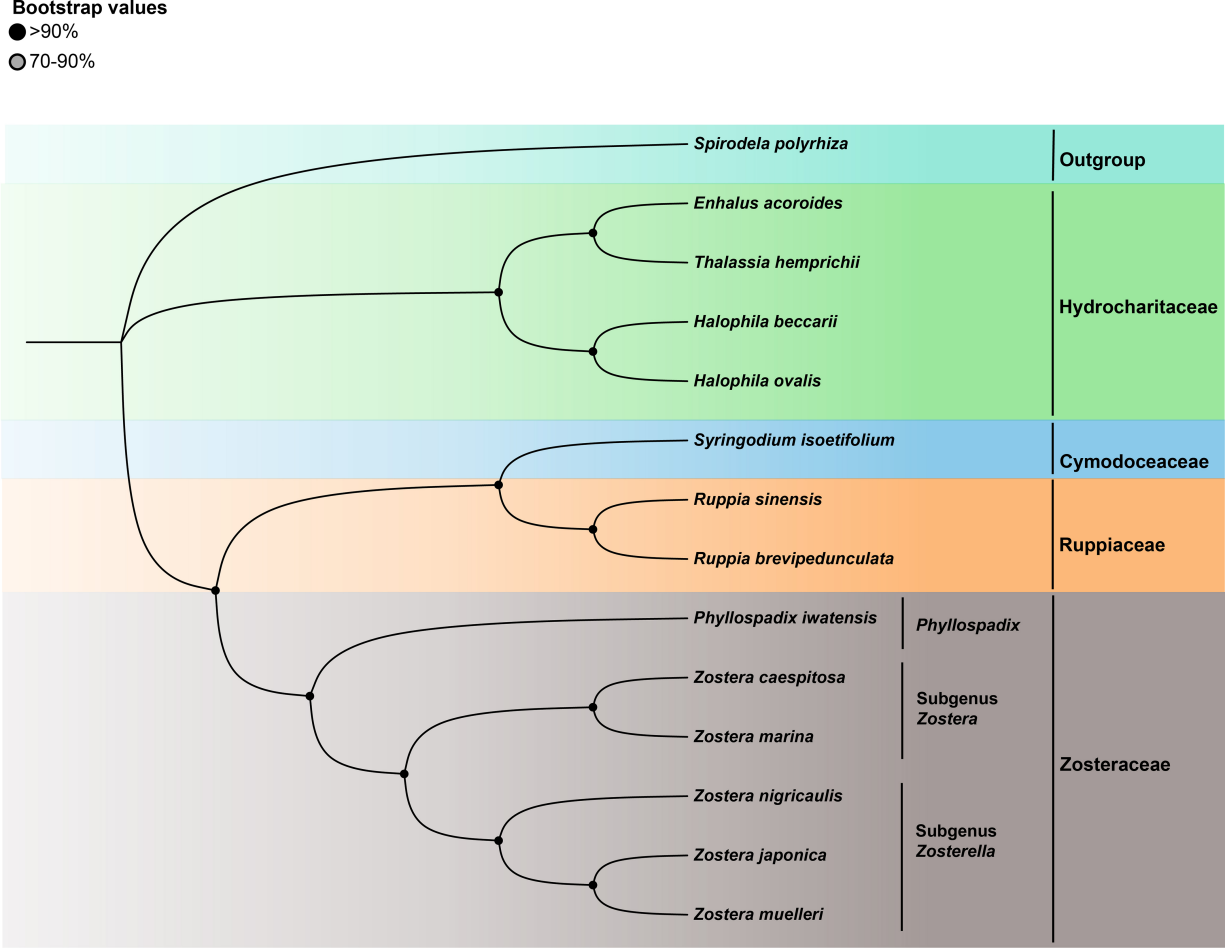
The ML tree of 13 seagrasses based on all shared cp-PCGs.

To investigate the phylogenetic relationship of Zosterceae, we analyzed the shared PCGs of the cp genome of 13 seagrass species. *Spirodela pholyhiza* was selected as the outgroup. Because of the high congruence between the ML and BI analyses of cp genomes, only the ML topology is presented here. Phylogenomic analysis using common 57 PCGs genes revealed four primary clades: Hydrocharitaceae, Cymodoceaceae, Ruppiaceae, and Zosteraceae, of which Cymodoceaceae and Ruppiaceae had a relatively close relationship (**Figure 5**). Furthermore, Zosteraceae were divided into three major clades: *Phyllospadix*, subgenus *Zostera*, and subgenus *Zosterella*. Within Zosteraceae, *Z. caespitosa* was more closely related to *Z. marina*, followed by *Z. nigricaulis*, *Z. japonica*, *Z. muelleri*, and *P. iwatensis*.

### 2.6 Identification of homologous sequences

**Figure 6:**
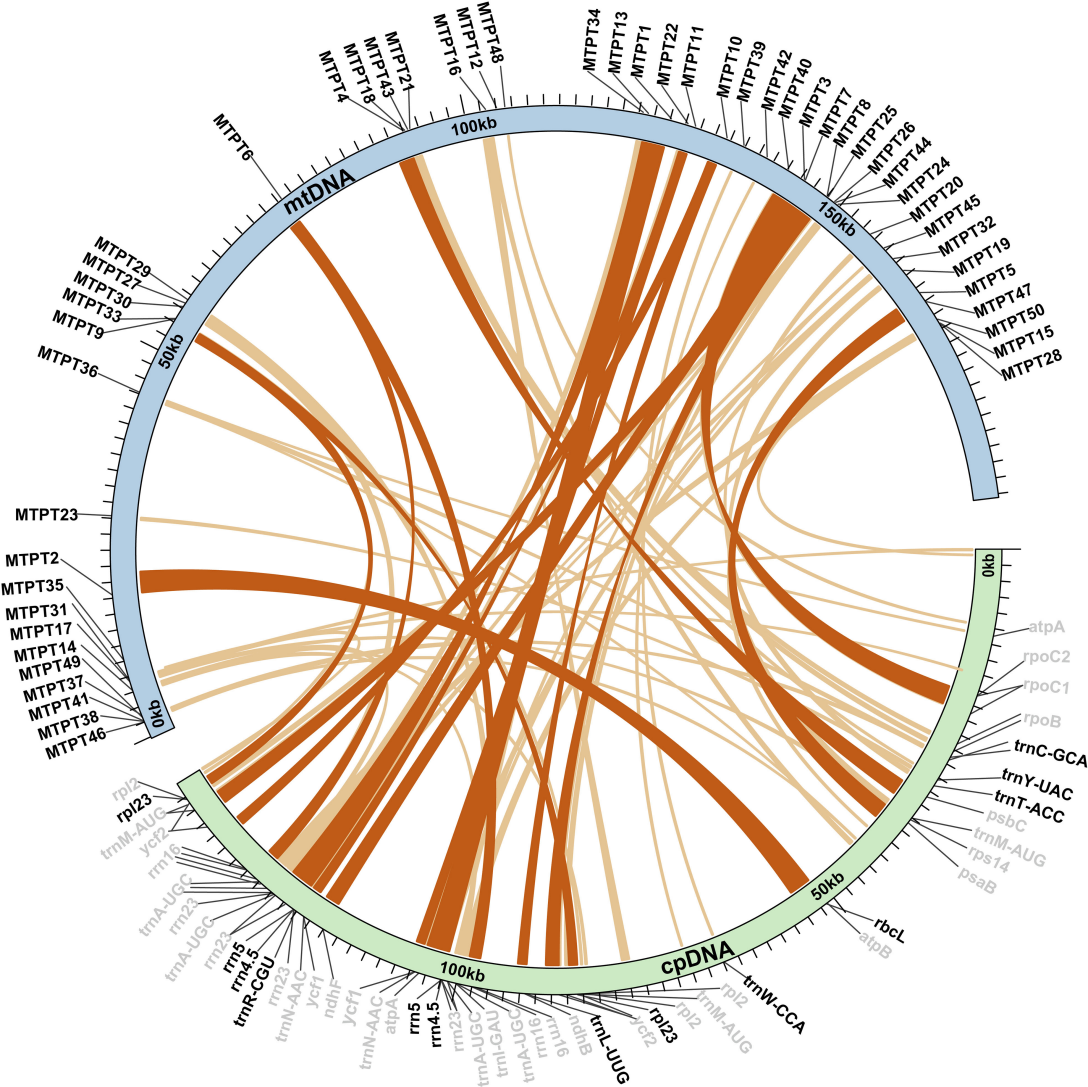
Homologous sequences between organelle genomes are shown as blue (mt) and green (cp) arcs. The inner arcs represent MTPT fragments, while outer gray and black labels indicate partial and intact genes, respectively.

Homologous sequences between the mt and cp genomes of *Z. caespitosa* were searched to further investigate gene residues from cp genome in the mt genome. Sequence similarity analysis identified 50 mitochondrial-plastid DNA transfers (MTPTs) in *Z. caespitosa* (**Figure 6**). The MTPTs were 44,662 bp long, accounting for 23.23% of the mt genome length and 31.02% of the total cp genome (**Supplementary Table S8**). Eleven fragments were longer than 1,000 bp, with the longest being MTPT1 (2,961 bp), which consists of the *rrn4.5*, *rrn5*, and *rrn23* genes. Most MTPTs in the mt genome of *Z. caespitosa* are derived from the tRNA gene and rRNA gene in the cp genome. Two proteins (*rbcL* and *rpl23*), two rRNA genes (*rrn4.5* and *rrn5*), and six tRNA genes (*trnC-GCA*, *trnY-UAC*, *trnT-ACC*, *trnW-CCA*, *trnL-UUG*, and *trnR-CGU*) were fully intact, and others were partial sequences.

### 2.7 RNA editing events

**Figure 7:**
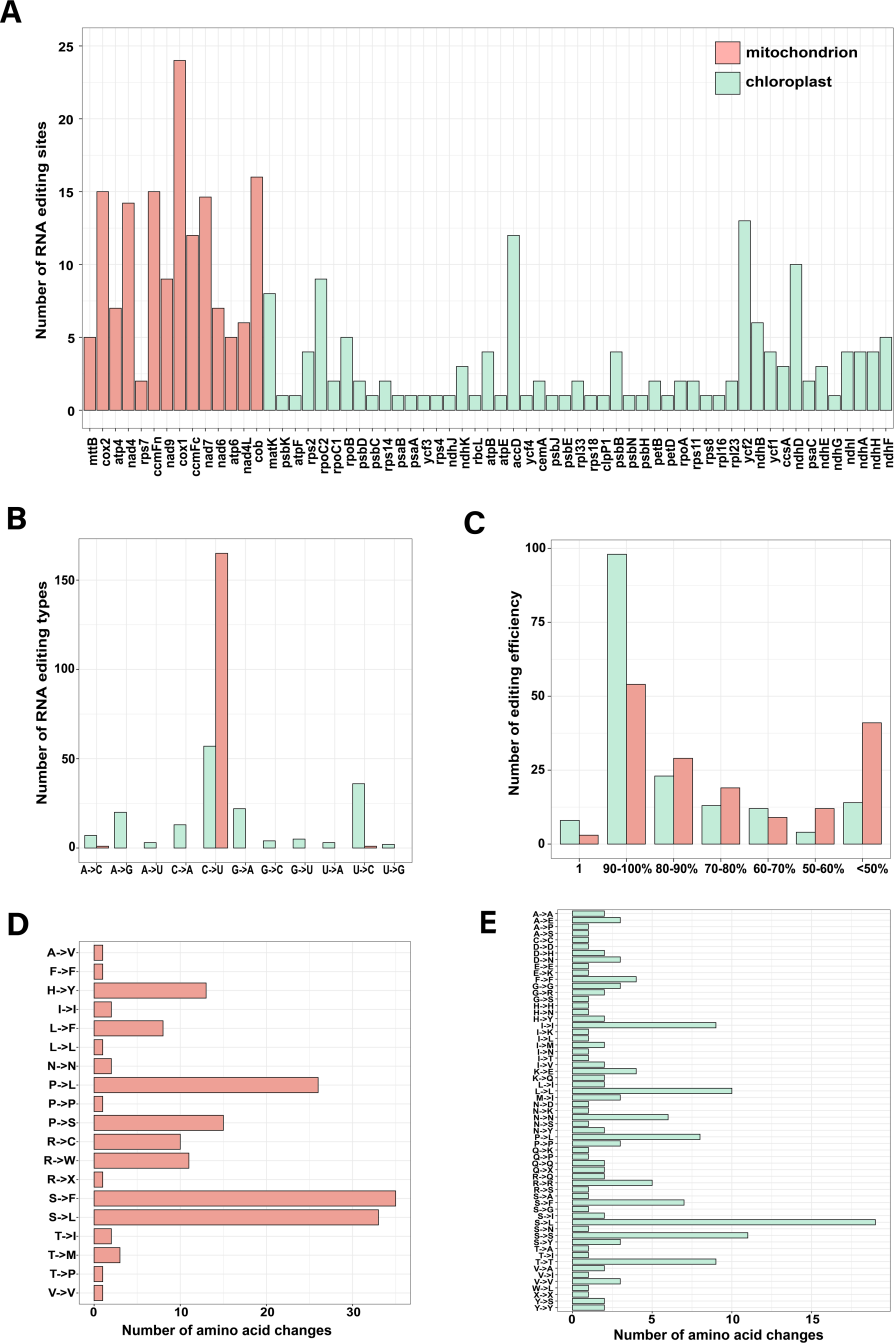
The features of RNA editing sites identified in PCGs of the organelle genome include **(A)** the number of RNA editing sites in each PCG; **(B)** types of RNA editing; **(C)** efficiency of RNA editing; and **(D, E)** the number of amino acid changes in mt and cp, respectively.

To identify RNA editing events of *Z. caespitosa*, we mapped RNA-seq data to the mt and cp genomes. A total of 167 and 172 RNA editing sites were identified in the mt and cp PCGs, respectively (**Figure 7A** and **Supplementary Tables S9**, **S10**). Four mt (*nad7*, *cox1*, *nad4*, and *cob*) and cp genes (*matK*, *rpoC2*, *accD*, and *ndhD*) had many editing sites. In addition, the fewest edits were observed in most cp genes, with only one RNA editing site. Furthermore, cp PCGs had 11 different types of RNA editing sites, including 33% of C–U, 21% of U–C, 13% of G–A, 12% of A–G, 7% of C–A, 4% of A–C, 3% of G–C, 3% of G–U, 2% of A–U, 2% of U–A, and 1% of U–G (**Figure 7B**). Most mt PCGs had this C-to-U editing type. Over 62% (106) cp and 34% (57) mt editing sites exhibited near 100% editing efficiency, indicating a more efficient editing process than initially expected (**Figure 7C**). Most importantly, 92.21% and 55.81% in mt and cp occurred above the first two bases of the codon, changing the corresponding amino acids (**Supplementary Tables S9**, **S10**). We further examined the number of the amino acid changes and found that 95.2% (159) in mt and 57.6% (99) in cp caused amino acid alterations. The most frequent amino acid changes in the *Z. caespitosa* organelle genome were from Pro to Leu and Ser to Leu (**Figures 7D, E**).

## 3 Discussion

### 3.1 The structure and size of the organelle genomes in *Z. caespitosa*

Studying the genomes of plant organelles enhances our comprehension of their functions, inheritance patterns, and replication mechanisms, while also offering valuable insights into their evolution and adaptation (Wang et al., 2024). This study successfully *de novo* assembled the complete organelle genomes of *Z. caespitosa*. Consistent with the common structure of organelle genomes in seagrass (Petersen et al., 2017; Chen et al., 2021), both genomes are circular, sized at 192,246 bp and 143,972 bp, respectively. However, a recent study found varying degrees of genome size, completeness, and fragmentation in the seagrass mt genomes, which are attributed to recombination (Ma et al., 2024). Compared to the cp genome, the larger mt genome is due to a high abundance of repetitive sequences, particularly dispersed repeats (the mt genome contains 5,028 dispersed repeats, and cp has 37) (**Supplementary Tables S4**, **S5**). Similar disparities in repeat content have been observed in *Dystaenia takeshimana* (Park and Park, 2023). The length of the *Z. caespitosa* mt genome is well among the ranges reported for seagrasses (Chen et al., 2022). Notably, seagrasses’ mt genomes were smaller compared to those of other monocotyledons (**Supplementary Table S11**). Several factors, including horizontal gene transfer (HGT), the number of repeated sequences, and the gain or loss of large intrageneric fragments, influence variations in plant mt genome sizes (Wu et al., 2022).

Furthermore, the cp genome of *Z. caespitosa* contains a distinctive quadripartite organization, with two IRs, an LSC, and an SSC, similar to the structural characteristics of cp genomes of other angiosperms (Daniell et al., 2016). This genome is the second smallest within the Zosteraceae family, with cp genomes ranging from 143,877 bp in *Z. marina* to 178,261 bp in *Thalassia hemprichii* (Chen et al., 2023). This difference may be caused by very short *ycf1* genes in the IR regions in *Z. caespitosa* (Olsen et al., 2016). The lengths of the cp genomes in the same family were similar (**Supplementary Table S11**), suggesting that the cp genome size variability is linked to phylogenetic relationships and evolutionary history (Zheng et al., 2017). Additionally, the expansion and contraction of the SC border and IR regions are major factors contributing to size variations in cp genomes, influencing their evolutionary rate (Dodsworth et al., 2015).

### 3.2 Codon usage pattern analysis in *Z. caespitosa*

Codon usage bias in genes, a notable evolutionary feature, occurs in various prokaryotic and eukaryotic organisms (Wang et al., 2023). These biases result from a complex interaction of natural selection, genetic drift, and mutational forces across long evolutionary periods (Chen et al., 2004; Zhang et al., 2022). According to angiosperm organelle genomes, this research showed that most codons ending with A/U have >1 RSCU values, possibly because of composition bias toward a high A/T ratio (Duan et al., 2021). This pattern is likely due to the structural stability of polyA and polyT, compared to polyC and polyG (Gragg et al., 2002).

The ENC-GC3 analysis suggests that mutations predominantly affect genes closely aligned with or overlapping the standard curve in the ENC-GC3 plot, probably altering their codon bias. Conversely, genes deviating from this curve are likely influenced by natural selection (Li et al., 2023; Wright, 1990). This study demonstrates that the majority of photosynthesis-associated genes in the cp genome and respiration-related genes in the mt genome of *Z. caespitosa* depart from the standard curve, indicating a greater impact of natural selection. This deviation pattern is consistent across species within the tea plant family (Li et al., 2023). Additionally, this study reveals that mt genome genes like *atp9*, with an ENC value of 33.25, and cp genome genes including *psbI* (33.96), *petN* (28.65), *psaI* (34.04), *psaJ* (33.05), *rpl36* (23.23), and *rpl32* (28.76), all with ENC values below 35, exhibit mutation-influenced codon usage biases. In contrast, genes with ENC values above 35 are predominantly shaped by natural or artificial selection (Wright, 1990).

### 3.3 Mutation rate analysis of *Zostera* sp*ecies*

The Ka/Ks is employed to evaluate the evolutionary rate of nucleotides and selective pressure, serving as an important indicator in species evolution (Li et al., 1985). In this study, the Ka/Ks of the cp genome is roughly 1/2 of that of the mt genome. The result was consistent with a previous study on the red alga *Porphyra* and phytoplankton *Phaeocystis* (Haptophyta), which have secondary cp; their mt mutation rates were higher than their cp rates (Smith et al., 2012, 2014). However, this trend contrasts most land plants and green algae, where the Ka/Ks of cp were three times higher than that of mt (Clegg et al., 1994; Smith, 2015; Smith and Keeling, 2015). The mutation rate variation across organisms is unclear but likely reflects the differences in the efficiency of DNA replication machinery between organelles and across taxonomic lineages (Drouin et al., 2008; Zhu et al., 2014; Smith, 2015; Gualberto and Newton, 2017). Additionally, the endosymbiotic history, specifically the number of endosymbiosis events, may be important, but their role in influencing these rates is poorly understood (Smith and Keeling, 2015).

### 3.4 Phylogenetic relationships in seagrass

Phylogenetic relationships within the family Zosteraceae, particularly regarding genus *Zostera* species, have been subjects of intense debate (Tanaka et al., 2003; Coyer et al., 2013). These studies used core barcodes to distinguish *Zostera* species within the Zosteraceae, which have potential limitations. In response, researchers have increasingly turned to cp genomes as a super barcode, which has proven successful in identifying numerous species and individuals across diverse studies (Kane et al., 2012; Chen et al., 2019). This study constructed phylogenetic trees based on common PCGs from 13 seagrass cp genomes. Results showed monophyly of subgenus *Zostera* and subgenus *Zosterella*. Interestingly, one good phylogenomic bootstrap branch indicated that *Z. caespitosa* typically emerged as a sister to *Z. marina*, demonstrating that *Z. caespitosa* was more closely related to *Z. marina* than other Zosteraceae species. Additionally, the results support the infrageneric classifications proposed by Chen et al., which divide seagrasses into four major clades: Zosteraceae, Ruppiaceae, Cymodoceaceae, and Hydrocharitaceae, with Cymodoceaceae and Ruppiaceae showing a relatively close relationship (Les and Tippery, 2013; Chen et al., 2022).

### 3.5 Horizontal gene transfers between organellar genomes in *Z. caespitosa*

Horizontal gene transfer between mt and cp genomes has been a significant phenomenon in the long-term evolution of angiosperms (Sloan and Wu, 2014; Gui et al., 2016). This study identified 50 MTPTs between the cp and mt genomes of *Z. caespitosa*, with a total length of 44,662 bp, accounting for 23.23% of the mt genome. Generally, the ratio of MTPTs in mt genomes varies from 0.56% in *Marchantia polymorpha* to 10.85% in *Phoenix dactylifera* (Zhao et al., 2018). The percentage in the mt genome of *Z. caespitosa* far exceeds the highest previously reported rates of approximately 10%–12% in *Boea* and *Cucurbita* (Zhang et al., 2012; Alverson et al., 2010). In the *Z. caespitosa* and *Z. marina* mt genomes, the frequent occurrence of MTPTs is linked to the recent integration of several large sequence transfers (Petersen et al., 2017). Among these MTPTs, MTPT1 is the longest. Previous studies have also documented the presence of large fragments of transferred cpDNA in mt genomes (Zhu et al., 2023). These large fragments likely play a significant role in angiosperms evolution by enhancing genetic diversity. Additionally, the origin of tRNAs in angiosperms mt is twofold: some are derived from the mt ancestor, while others are obtained from the cp via HGT (Sprinzl and Vassilenko, 2005). In addition to gene transfer between mt and cp genomes, HGT most frequently occurs from organelles to the nuclear genome, resulting in the production of nuclear plastid DNA (NUPTs) and nuclear mitochondrial DNA (NUMTs). Recent studies on *Thalassia testudinum* have found large, uninterrupted nuclear mitochondrial DNA sequences (NUMTs), indicating a recent mitochondrial DNA transfer. In contrast, *Z. marina* shows fewer such sequences, suggesting less frequent mitochondrial DNA integration. These large, uninterrupted NUMTs in *T. testudinum* may result from genome instability caused by TE expansion (Ma et al., 2024).

### 3.6 RNA editing events

Recent studies have demonstrated that RNA editing is prevalent across plant organelle genes and is crucial in energy metabolism and regulating genetics (Li et al., 2019) In this study, the RNA-seq data identified 167 RNA editing sites across mt-PCGs and 172 RNA across cp-PCGs. The number of editing sites is higher than those reported in most angiosperm cp genomes (20–60) (Knoop, 2023). Mt genes such as *nad7*, *cox1*, *nad4*, *cob*, and cp genes, including *matK*, *rpoC2*, *accD*, and *ndhD* had notably high editing frequencies. This study further investigated the impact of editing on protein structure, confirming that no new transmembrane domains were created (**Supplementary Figures S1**, **S2**). Additionally, editing nine differential sites (*accD-*157, *accD-*624, *accD-*1220, *rpoC2-*2255, *rpoC2-*2752, *rpoC2-*2822, *mat-*748, *matK-*178, and *matK-*799) changed the secondary structure composition surrounding the editing sites in the cp (**Supplementary Figure S3**). Editing 12 different sites (*nad7-*927, *cox1-*254, *cox1-*352, *cox1-*868, *cox1-*1037, *cox1-*1279*, nad4-*29, *nad4-*197*, nad4-*362*, nad4-*599*, cob-*400, and *cob-*794) altered the secondary structure composition surrounding the editing sites in the mt. This editing mechanism effectively repairs potential defects by disrupting and folding alpha-helices, enhancing structural protein stability (Yuar and Go, 2008).

Similarly, previous studies revealed C-to-U as the predominant type of editing in mt-PCGs (98.8%) and pt-PCGs (40%) of *Z. caespitosa* (Gray and Covello, 1993). Additionally, *Z. caespitosa* had non-canonical edits such as U to C, G to U, C to A, A to G, and G to A, typically found only in ancestral land plants (Chateigner-Boutin and Small, 2010; Uthaipaisanwong et al., 2012) *Z. caespitosa* also had unexpectedly rare editing events like A to C, A to U, U to A, G to C, and U to G, not previously reported in other species. These RNA editing events potentially alter protein structure or interaction by nonsynonymously replacing conserved amino acids, most notably changing Pro to Leu and Ser to Leu. These substitution shifts the physicochemical properties from hydrophilic to hydrophobic, increasing the hydrophobicity of interface residues, crucial for protein–protein interactions and enzyme efficiency (Jobson and Qiu, 2008; Giegé and Brennicke, 1999).

## 4 Conclusions

This study reported the complete organelle genomes of *Z. caespitosa*, enabling their comprehensive comparison. The PCGs in the organelle genome exhibits a strong A/U bias at codon endings, a selection-driven codon bias. The mt genome includes abundant cpDNA transferred fragments, much higher than in most angiosperms. The Ka/Ks of the *Zostera* mt genome are twofold higher than those in the cp genome. Remarkably, numerous RNA editing events were identified in cp-PCGs, including five rare types of RNA editing.

## 5 Materials and methods

### 5.1 Sample collection and organelle genome sequencing

Fresh *Z. caespitosa* leaves were collected from Yantai, China (121.4534 E, 37.5193 N). Prof. Quansheng Zhang verified the voucher specimens stored in the Ocean School of Yantai University. Leaves’ DNA was extracted using the DNA extraction kit (Invitrogen, CA, USA). DNA library was constructed using the Illumina TruSeq Library Preparation Kit (Illumina, CA, USA), in accordance with the manufacturer’s guidelines. These libraries were sequenced using the Illumina NovaSeq 6000 (Biozeron, Shanghai, China), producing raw data consisting of 150-bp paired-end reads. Constructing the long fragment library, its quality was assessed with Qubit, and then it was sequenced on the ONT platform (Biozeron, Shanghai, China).

### 5.2 Assembly of *Z. caespitosa* mt genome

The Illumina sequencing raw data were filtered with Trimmomatic v0.39 before the mt genome assembly (Bolger et al., 2014). All ONT-generated long reads were aligned to the *Z. marina* (NC_035345.1) mt genome, with Minimap2 v2.10-r761 using its default settings, processed into a Pairwise Mapping Format (PAF) file (Li, 2018). Homologous reads with a mapping quality >20 were treated as potential mt sequences. Subsequently, *de novo* assembly was performed on the homologous ONT long reads and the clean paired-end reads using Canu v2.2 and GetOrganelle v1.7.5 with default settings (Koren et al., 2017; Jin et al., 2020). BLASTN was used to align the draft contigs from Canu and GetOrganelle with the *Z. marina* mt CDSs to detect the candidate mt genome contigs. Using overlapping markers as a guide, the selected contigs were manually joined to generate the complete mt genome sequences. The circularity of the assembly was confirmed using the “check_circularity.pl” script, part of the sprai package (http://zombie.cb.k.u-tokyo.ac.jp/sprai/).

The alignment of long ONT reads and short Illumina to the mt genomes was performed using BWA, removing multi-mapped reads, unmapped reads, and PCR duplicates. Coverage information for the mt genomes was obtained by sorting the Binary Alignment/Map (BAM) files, and the accuracy was manually checked using the Integrative Genomics Viewer (IGV) with the BAM files as references.

### 5.3 Assembly of *Z. caespitosa* cp genome

Quality control was performed on the raw sequencing reads using FastQC, followed by trimming of redundant reads or low-quality reads (*Q* < 20) using Trimmomatic v0.39 (Bolger et al., 2014). The trimmed reads were assembled using GetOrganelle (v1.7.5) with default parameters (Jin et al., 2020). The *Z. marina (*NC_036014.1) cp genome sequence served as the reference for re-assembly, generating multiple potential cp genome assemblies. The final cp genome assembly’s accuracy, especially regarding the IR order and continuity, was checked and manually corrected as needed, using BLASTN (E-value cutoff of 10{sp}−5{/sp}) against reference cp genomes. The “check_circularity.pl” script from the sprai package was used to evaluate the circularity of the cp assembly. The boundaries of the SSC, LSC, IR, and regions were determined using BLASTN self-alignment.

### 5.4 Mt and cp genome of *Z. caespitosa* annotation

The mt and cp genes were annotated using GeSeq (Tillich et al., 2017) and BLASTN, with *Z. marina* (NC_035345.1) and *Z. marina* (NC_036014.1) as references, respectively. Exon/intron and start/stop codons boundaries in PCGs were manually corrected with Snap Gene Viewer, using reference gene models for guidance. The Organellar Genome DRAW (OGDRAW) software was used to visualize the mt and cp genomes graphically (Greiner et al., 2019). These mt and cp genome sequences are available in GenBank with the accession numbers PP566026 and PP566025, respectively.

### 5.5 Repeat sequence analysis

Here, SSRs were identified using the MISA tool (Beier et al., 2017), with the minimum repeat numbers set to 10, 5, 4, 3, 3, and 3 for mono-, di-, tri-, tetra-, penta-, and hexa-nucleotides, respectively. Dispersed repetitive, including forward (F), reverse (R), complementary (C), and palindromic (P) repeats, were identified using the REPuter software (Kurtz et al., 2001). The analysis was conducted with a 30-bp minimum repeat length, a 90-bp maximum repeat size, and a Hamming distance threshold of three. The detection of tandem repeats was performed using Tandem Repeats Finder (Benson, 1999).

### 5.6 Codon usage bias analysis

Phylosuite (v1.1.16) was used to extract the PCGs from mt and cp genomes. The codon preference of PCGs in these genomes was assessed using MEGA (v7.0), and RSCU values were calculated (Kumar et al., 2016). CodonW (v1.4.4) was employed to calculate the ENC-GC3 values, and the data were visualized with ggplot2 in R package.

### 5.7 Selective pressure estimation and phylogenetic analysis

Using MAFFT version 7.427 (Katoh and Standley, 2013), the core PCG sequences were aligned, and the Ka/Ks ratios for *Z. caespitosa*, *Z. marina*, and *Z. japonica* were calculated via the MLWL-based Ka/Ks Calculator (Wang et al., 2010). Python and R software were used to visualize the plots. The core CDSs of 13 seagrass cp genomes were aligned using MAFFT, and the alignments were used to construct Bayesian trees with MrBayes 3.2. The most suitable nucleotide substitution model was chosen through ModelFinder (Nguyen et al., 2015). Further, ML analyses were performed with IQ-TREE version 1.5.5 (Nguyen et al., 2015) utilizing ultrafast bootstrap (1,000 replicates) and a partition model. Finally, the trees were visualized using normal tree tools.

### 5.8 Gene transfer between mt and cp genomes

The mt and cp genome sequences for *Z. caespitosa* were aligned using the BLASTn tool, with the default parameters. Subsequently, the distribution of MTPTs was displayed using TBtools, which integrates the Circos package (Chen et al., 2020). Additionally, GeSeq was used to annotate the MTPTs, and duplicates, such as the two MTPTs in the cp genome’s IRs, were excluded.

### 5.9 Identification of RNA editing events

RNA editing events in the PCGs of the *Z. caespitosa* organelle genomes were identified using RNA-seq and genomic sequencing data. Quality control of the raw RNA reads was conducted via FastQC using default parameters. The RNA-seq reads were then mapped to the cp and mt genomes of *Z. caespitosa* using Tophat (Trapnell et al., 2009). Picard (https://github.com/broadinstitute/picard) was employed to remove duplicate records from the resulting BAM files. Next, single-nucleotide polymorphisms (SNPs) were identified using GATK (McKenna et al., 2010) and Samtools (Li et al., 2009) for genotyping analysis. The IGV was used to examine the mapped reads. The efficiency of RNA editing was quantified as the ratio of edited reads to total mapped reads (Li et al., 2009).

## Data Availability

The raw data were deposited at the NCBI SRA database https://www.ncbi.nlm.nih.gov/sra; with the accession number: SRR31595597–SRR31595599.
