# Supplementary material for: The organelle genomes of the endangered seagrass Zostera caespitosa reveal sequence divergences, massive gene transfer, and uncommon RNA editing types
Source: Front Plant Sci. 2025 Feb 17;16:1550467. doi: 10.3389/fpls.2025.1550467 (PMC11873085; doi:10.3389/fpls.2025.1550467)
Supplement: Supplementary file 1 [file DataSheet1.docx]

Supplementary Material

**1 Supplementary** **Tables and Figures**

## Supplementary Tables

**Supplementary Table S1** General features of mitochondrial and chloroplast genomes of *Z. caespitosa*

|  | **Mitochondrial genome** |
| --- | --- |
| Genbank accession number | PP566026 |
| Genome length (bp) | 192,246 |
| GC (%) | 45.89 |
| CDS total length (bp) | 26,874 |
| Intergenic region length (bp) | 165,372 |
| Number of total gene | 50 |
| Number of CDS | 27 |
| tRNA | 20 |
| tRNA length (bp) | 1,449 |
| rRNA | 3 |
| rRNA length (bp) | 5,220 |
| ORF | 120 |
|  | **Chloroplast genome** |
| Genbank accession number | PP566025 |
| Genome length (bp) | 143,972 |
| LSC length (bp) | 83,313 |
| SSC length (bp) | 12,405 |
| IRs length (bp) | 24,127 |
| Gene total length (bp) | 71,619 |
| Total GC content (%) | 35.43 |
| Number of genes | 131 |
| Number of protein-coding genes | 85 |
| Number of tRNAs | 38 |
| Number of rRNAs | 8 |
| Number of repeat genes | 12 |

**Supplementary Table S2** SSRs in the mitochondrial and chloroplast of *Z. caespitosa*. p1, p2, p3, p4, p5, p6 in SSR type means Monomeric, Dimeric, Trimeric, Tetrameric, Pentameric, Hexamer.

| **Genome type** | **SSR nr.** | **SSR type** | **SSR** | **Size** | **Start** | **End** |
| --- | --- | --- | --- | --- | --- | --- |
| Mitochondrial genome | 1 | p2 | (TA)5 | 10 | 1958 | 1967 |
|  | 2 | p1 | (T)33 | 33 | 13293 | 13325 |
|  | 3 | p2 | (CT)5 | 10 | 17107 | 17116 |
|  | 4 | p4 | (AGGC)3 | 12 | 17654 | 17665 |
|  | 5 | p4 | (CAAA)3 | 12 | 22551 | 22562 |
|  | 6 | p2 | (TA)5 | 10 | 43906 | 43915 |
|  | 7 | p3 | (TCT)4 | 12 | 68898 | 68909 |
|  | 8 | p4 | (TTTG)3 | 12 | 118463 | 118474 |
|  | 9 | p4 | (ATTT)3 | 12 | 127331 | 127342 |
|  | 10 | p2 | (AT)6 | 12 | 133077 | 133088 |
|  | 11 | p2 | (AT)5 | 10 | 133094 | 133103 |
|  | 12 | p4 | (ACAT)3 | 12 | 134187 | 134198 |
|  | 13 | p3 | (TTG)4 | 12 | 138243 | 138254 |
|  | 14 | p2 | (AT)5 | 10 | 143661 | 143670 |
|  | 15 | p4 | (AAGT)3 | 12 | 158522 | 158533 |
|  | 16 | p4 | (CAAA)3 | 12 | 163155 | 163166 |
|  | 17 | p4 | (AAAG)3 | 12 | 163223 | 163234 |
|  | 18 | p6 | (TAGTGA)3 | 18 | 167954 | 167971 |
|  | 19 | p2 | (AG)5 | 10 | 168162 | 168171 |
|  | 20 | p4 | (GAAA)3 | 12 | 177120 | 177131 |
|  | 21 | p4 | (TCTT)3 | 12 | 190868 | 190879 |
| Chloroplast genome | 21 | p1 | (A)15 | 15 | 14943 | 14957 |
|  | 22 | p4 | (AAAT)3 | 12 | 15929 | 15940 |
|  | 23 | p1 | (T)11 | 11 | 18114 | 18124 |
|  | 24 | p1 | (A)10 | 10 | 18257 | 18266 |
|  | 25 | p1 | (T)11 | 11 | 18641 | 18651 |
|  | 26 | p2 | (AT)5 | 10 | 19481 | 19490 |
|  | 27 | p1 | (T)12 | 12 | 22159 | 22170 |
|  | 28 | p1 | (T)12 | 12 | 22654 | 22665 |
|  | 29 | p2 | (AT)5 | 10 | 27266 | 27275 |
|  | 30 | p2 | (AT)7 | 14 | 28507 | 28520 |
|  | 31 | p2 | (TA)5 | 10 | 28646 | 28655 |
|  | 32 | p1 | (A)11 | 11 | 30475 | 30485 |
|  | 33 | p1 | (T)12 | 12 | 30937 | 30948 |
|  | 34 | p4 | (TTTA)3 | 12 | 31357 | 31368 |
|  | 35 | p1 | (A)10 | 10 | 31656 | 31665 |
|  | 36 | p1 | (A)14 | 14 | 32283 | 32296 |
|  | 37 | p2 | (TA)6 | 12 | 35666 | 35677 |
|  | 38 | p4 | (TTTA)3 | 12 | 42305 | 42316 |
|  | 39 | p1 | (A)11 | 11 | 44302 | 44312 |
|  | 40 | p4 | (AAGA)3 | 12 | 44428 | 44439 |
|  | 41 | p1 | (A)10 | 10 | 45478 | 45487 |
|  | 42 | p2 | (TA)5 | 10 | 45574 | 45583 |
|  | 43 | p1 | (A)10 | 10 | 45723 | 45732 |
|  | 44 | p1 | (A)10 | 10 | 45790 | 45799 |
|  | 45 | p2 | (AT)6 | 12 | 46192 | 46203 |
|  | 46 | p2 | (TA)7 | 14 | 46864 | 46877 |
|  | 47 | p1 | (A)11 | 11 | 47340 | 47350 |
|  | 48 | p1 | (A)10 | 10 | 47938 | 47947 |
|  | 49 | p1 | (T)11 | 11 | 50278 | 50288 |
|  | 50 | p1 | (A)10 | 10 | 52773 | 52782 |
|  | 51 | p1 | (A)12 | 12 | 57308 | 57319 |
|  | 52 | p1 | (A)11 | 11 | 57847 | 57857 |
|  | 53 | p1 | (A)11 | 11 | 59731 | 59741 |
|  | 54 | p1 | (T)10 | 10 | 60741 | 60750 |
|  | 55 | p1 | (T)12 | 12 | 63142 | 63153 |
|  | 56 | p1 | (T)10 | 10 | 63306 | 63315 |
|  | 57 | p1 | (T)11 | 11 | 63384 | 63394 |
|  | 58 | p4 | (TACT)3 | 12 | 63510 | 63521 |
|  | 59 | p2 | (TA)6 | 12 | 64532 | 64543 |
|  | 60 | p1 | (T)11 | 11 | 65809 | 65819 |
|  | 61 | p1 | (A)13 | 13 | 65904 | 65916 |
|  | 62 | p1 | (A)12 | 12 | 66317 | 66328 |
|  | 63 | p1 | (A)10 | 10 | 66857 | 66866 |
|  | 64 | p2 | (AT)5 | 10 | 67381 | 67390 |
|  | 65 | p1 | (A)11 | 11 | 68009 | 68019 |
|  | 66 | p1 | (T)11 | 11 | 68125 | 68135 |
|  | 67 | p1 | (T)10 | 10 | 69161 | 69170 |
|  | 68 | p1 | (A)11 | 11 | 70193 | 70203 |
|  | 69 | p1 | (A)13 | 13 | 71128 | 71140 |
|  | 70 | p4 | (AATA)3 | 12 | 71689 | 71700 |
|  | 71 | p1 | (T)10 | 10 | 73573 | 73582 |
|  | 72 | p1 | (T)10 | 10 | 77589 | 77598 |
|  | 73 | p1 | (T)12 | 12 | 77642 | 77653 |
|  | 74 | p1 | (T)10 | 10 | 78916 | 78925 |
|  | 75 | p1 | (T)10 | 10 | 80450 | 80459 |
|  | 76 | p2 | (TA)5 | 10 | 81193 | 81202 |
|  | 77 | p1 | (T)12 | 12 | 81580 | 81591 |
|  | 78 | p1 | (A)10 | 10 | 82077 | 82086 |
|  | 79 | p6 | (TTTCCG)3 | 18 | 88418 | 88435 |
|  | 80 | p1 | (T)11 | 11 | 90333 | 90343 |
|  | 81 | p2 | (TA)5 | 10 | 91745 | 91754 |
|  | 82 | p2 | (TA)6 | 12 | 91760 | 91771 |
|  | 83 | p1 | (T)11 | 11 | 93063 | 93073 |
|  | 84 | p1 | (A)10 | 10 | 107169 | 107178 |
|  | 85 | p1 | (T)10 | 10 | 108206 | 108215 |
|  | 86 | p4 | (AATA)3 | 12 | 109011 | 109022 |
|  | 87 | p1 | (A)16 | 16 | 111003 | 111018 |
|  | 88 | p1 | (T)11 | 11 | 111650 | 111660 |
|  | 89 | p1 | (T)11 | 11 | 112296 | 112306 |
|  | 90 | p1 | (T)10 | 10 | 112965 | 112974 |
|  | 91 | p1 | (A)10 | 10 | 114103 | 114112 |
|  | 92 | p2 | (TC)5 | 10 | 116303 | 116312 |
|  | 93 | p4 | (AAAT)3 | 12 | 116865 | 116876 |
|  | 94 | p1 | (T)11 | 11 | 117272 | 117282 |
|  | 95 | p1 | (A)14 | 14 | 117313 | 117326 |
|  | 96 | p2 | (AT)5 | 10 | 117814 | 117823 |
|  | 97 | p1 | (T)14 | 14 | 117909 | 117922 |
|  | 98 | p1 | (T)10 | 10 | 120108 | 120117 |
|  | 99 | p1 | (A)11 | 11 | 134213 | 134223 |
|  | 100 | p2 | (AT)6 | 12 | 135514 | 135525 |
|  | 101 | p2 | (AT)5 | 10 | 135531 | 135540 |
|  | 102 | p1 | (A)11 | 11 | 136943 | 136953 |
|  | 103 | p6 | (ACGGAA)3 | 18 | 138850 | 138867 |

**Supplementary Table S3** Tandem repeat sequences in the mitochondrial and chloroplast genome of *Z. caespitosa*.

| **Genome type** | **Indices** | **Period** **size** | **Copy**  **number** | **Consensus**  **size** | **Percent**  **matches** | **Percent**  **indels** | **Score** | **A** | **C** | **G** | **T** | **Entropy(0-2)** |
| --- | --- | --- | --- | --- | --- | --- | --- | --- | --- | --- | --- | --- |
| Mitochondrial genome | 1481--1544 | 30 | 2.1 | 30 | 100 | 0 | 128 | 21 | 37 | 6 | 34 | 1.79 |
|  | 4051--4120 | 30 | 2.4 | 30 | 97 | 2 | 133 | 20 | 38 | 7 | 34 | 1.8 |
|  | 4135--4514 | 89 | 4.3 | 89 | 100 | 0 | 760 | 23 | 35 | 8 | 32 | 1.86 |
|  | 4135--4514 | 59 | 6.4 | 60 | 92 | 1 | 624 | 23 | 35 | 8 | 32 | 1.86 |
|  | 8586--8644 | 28 | 2.1 | 28 | 96 | 0 | 109 | 23 | 20 | 33 | 22 | 1.97 |
|  | 9109--10242 | 64 | 17.8 | 64 | 94 | 1 | 1612 | 24 | 23 | 28 | 23 | 2 |
|  | 11623--11720 | 45 | 2.2 | 45 | 85 | 3 | 126 | 23 | 17 | 29 | 29 | 1.97 |
|  | 13293--13325 | 1 | 33 | 1 | 100 | 0 | 66 | 0 | 0 | 0 | 100 | 0 |
|  | 13435--13481 | 20 | 2.3 | 20 | 85 | 3 | 58 | 25 | 19 | 31 | 23 | 1.98 |
|  | 13449--13490 | 20 | 2.1 | 20 | 100 | 0 | 84 | 21 | 23 | 30 | 23 | 1.99 |
|  | 15688--15797 | 37 | 3 | 37 | 95 | 0 | 202 | 31 | 10 | 38 | 20 | 1.85 |
|  | 16218--16626 | 33 | 12.4 | 33 | 99 | 0 | 809 | 33 | 11 | 36 | 18 | 1.87 |
|  | 22442--22494 | 16 | 3.3 | 16 | 91 | 0 | 88 | 35 | 41 | 3 | 18 | 1.69 |
|  | 24853--24935 | 37 | 2.3 | 37 | 93 | 2 | 141 | 36 | 18 | 38 | 7 | 1.78 |
|  | 24936--24989 | 24 | 2.3 | 23 | 87 | 9 | 74 | 29 | 18 | 35 | 16 | 1.93 |
|  | 25531--25568 | 18 | 2.1 | 18 | 100 | 0 | 76 | 18 | 36 | 23 | 21 | 1.95 |
|  | 34251--34279 | 15 | 1.9 | 15 | 100 | 0 | 58 | 13 | 34 | 13 | 37 | 1.85 |
|  | 34952--34980 | 15 | 1.9 | 15 | 100 | 0 | 58 | 13 | 34 | 13 | 37 | 1.85 |
|  | 37781--37914 | 64 | 2.1 | 64 | 92 | 4 | 225 | 21 | 36 | 8 | 33 | 1.83 |
|  | 37874--37928 | 18 | 3.1 | 18 | 67 | 0 | 56 | 18 | 38 | 10 | 32 | 1.85 |
|  | 37957--37983 | 12 | 2.2 | 12 | 100 | 0 | 54 | 14 | 37 | 14 | 33 | 1.88 |
|  | 43429--43492 | 30 | 2.1 | 30 | 100 | 0 | 128 | 21 | 37 | 6 | 34 | 1.79 |
|  | 45997--46066 | 30 | 2.4 | 30 | 97 | 2 | 133 | 20 | 38 | 7 | 34 | 1.8 |
|  | 46081--46156 | 30 | 2.5 | 30 | 97 | 0 | 143 | 18 | 36 | 10 | 34 | 1.85 |
|  | 48649--48677 | 15 | 1.9 | 15 | 100 | 0 | 58 | 37 | 13 | 34 | 13 | 1.85 |
|  | 49788--49822 | 18 | 1.9 | 18 | 100 | 0 | 70 | 40 | 17 | 20 | 22 | 1.92 |
|  | 50478--50513 | 19 | 1.9 | 19 | 88 | 5 | 56 | 13 | 27 | 27 | 30 | 1.94 |
|  | 51620--51725 | 54 | 2 | 54 | 94 | 0 | 185 | 16 | 37 | 12 | 33 | 1.85 |
|  | 51710--51740 | 15 | 2.1 | 15 | 100 | 0 | 62 | 12 | 35 | 12 | 38 | 1.82 |
|  | 52703--52729 | 12 | 2.2 | 12 | 100 | 0 | 54 | 14 | 37 | 14 | 33 | 1.88 |
|  | 52799--52851 | 16 | 3.4 | 16 | 79 | 10 | 74 | 11 | 37 | 15 | 35 | 1.83 |
|  | 52972--52998 | 12 | 2.2 | 12 | 100 | 0 | 54 | 14 | 37 | 14 | 33 | 1.88 |
|  | 53009--53080 | 35 | 2.1 | 35 | 100 | 0 | 144 | 11 | 41 | 13 | 33 | 1.8 |
|  | 53186--53212 | 12 | 2.2 | 12 | 100 | 0 | 54 | 14 | 37 | 14 | 33 | 1.88 |
|  | 55126--55154 | 15 | 1.9 | 15 | 100 | 0 | 58 | 13 | 34 | 13 | 37 | 1.85 |
|  | 57582--57634 | 16 | 3.4 | 16 | 79 | 10 | 74 | 11 | 37 | 15 | 35 | 1.83 |
|  | 57584--57660 | 29 | 2.6 | 30 | 87 | 6 | 104 | 12 | 37 | 14 | 35 | 1.84 |
|  | 69887--69963 | 29 | 2.6 | 30 | 87 | 6 | 104 | 35 | 14 | 37 | 12 | 1.84 |
|  | 69913--69965 | 16 | 3.4 | 16 | 79 | 10 | 74 | 35 | 15 | 37 | 11 | 1.83 |
|  | 71933--71962 | 13 | 2.2 | 14 | 94 | 5 | 53 | 26 | 20 | 33 | 20 | 1.97 |
|  | 72199--72225 | 12 | 2.2 | 12 | 100 | 0 | 54 | 33 | 14 | 37 | 14 | 1.88 |
|  | 75746--75783 | 20 | 1.9 | 20 | 88 | 0 | 58 | 15 | 39 | 0 | 44 | 1.47 |
|  | 75871--75900 | 15 | 2 | 15 | 93 | 0 | 51 | 16 | 33 | 13 | 36 | 1.88 |
|  | 77641--77824 | 30 | 6 | 30 | 63 | 15 | 79 | 35 | 8 | 37 | 17 | 1.81 |
|  | 77670--77851 | 62 | 2.9 | 62 | 100 | 0 | 364 | 34 | 9 | 37 | 18 | 1.84 |
|  | 77853--77882 | 15 | 2 | 15 | 93 | 0 | 51 | 36 | 13 | 33 | 16 | 1.88 |
|  | 79648--79702 | 18 | 3.1 | 18 | 70 | 0 | 56 | 34 | 10 | 36 | 18 | 1.86 |
|  | 79566--79680 | 60 | 1.9 | 60 | 81 | 0 | 140 | 27 | 8 | 39 | 24 | 1.85 |
|  | 83589--83879 | 30 | 9.8 | 30 | 90 | 2 | 480 | 23 | 35 | 8 | 32 | 1.86 |
|  | 83589--83900 | 59 | 5.3 | 59 | 93 | 1 | 509 | 23 | 34 | 8 | 32 | 1.86 |
|  | 83589--83879 | 89 | 3.3 | 89 | 100 | 0 | 582 | 23 | 35 | 8 | 32 | 1.86 |
|  | 83957--83986 | 15 | 2 | 15 | 93 | 0 | 51 | 16 | 33 | 13 | 36 | 1.88 |
|  | 84296--84322 | 12 | 2.2 | 12 | 100 | 0 | 54 | 14 | 37 | 14 | 33 | 1.88 |
|  | 85058--85092 | 15 | 2.3 | 15 | 100 | 0 | 70 | 25 | 11 | 42 | 20 | 1.85 |
|  | 87341--87405 | 30 | 2.1 | 33 | 85 | 8 | 91 | 41 | 7 | 40 | 10 | 1.69 |
|  | 87360--87406 | 15 | 3.1 | 15 | 73 | 11 | 51 | 40 | 6 | 38 | 14 | 1.72 |
|  | 88205--88258 | 27 | 2 | 27 | 92 | 0 | 90 | 40 | 11 | 37 | 11 | 1.76 |
|  | 95583--95665 | 37 | 2.3 | 37 | 93 | 2 | 141 | 36 | 18 | 38 | 7 | 1.78 |
|  | 95666--95719 | 24 | 2.3 | 23 | 87 | 9 | 74 | 29 | 18 | 35 | 16 | 1.93 |
|  | 96261--96298 | 18 | 2.1 | 18 | 100 | 0 | 76 | 18 | 36 | 23 | 21 | 1.95 |
|  | 108839--108865 | 12 | 2.2 | 12 | 100 | 0 | 54 | 14 | 37 | 14 | 33 | 1.88 |
|  | 115457--115494 | 18 | 2.1 | 18 | 100 | 0 | 76 | 21 | 23 | 36 | 18 | 1.95 |
|  | 116090--116172 | 37 | 2.3 | 37 | 93 | 2 | 141 | 7 | 38 | 18 | 36 | 1.78 |
|  | 118532--118583 | 16 | 3.2 | 16 | 94 | 0 | 95 | 19 | 1 | 42 | 36 | 1.62 |
|  | 118813--118922 | 60 | 1.9 | 59 | 83 | 7 | 145 | 31 | 17 | 42 | 8 | 1.78 |
|  | 128916--128964 | 23 | 2.1 | 23 | 100 | 0 | 98 | 44 | 8 | 18 | 28 | 1.78 |
|  | 133052--133101 | 13 | 3.8 | 13 | 77 | 20 | 50 | 44 | 0 | 12 | 44 | 1.41 |
|  | 133041--133085 | 23 | 1.9 | 24 | 90 | 4 | 74 | 40 | 0 | 11 | 48 | 1.39 |
|  | 136896--136925 | 15 | 2 | 15 | 93 | 0 | 51 | 16 | 33 | 13 | 36 | 1.88 |
|  | 136980--137120 | 32 | 4.5 | 32 | 72 | 12 | 121 | 31 | 30 | 10 | 26 | 1.9 |
|  | 137116--137145 | 11 | 2.6 | 12 | 94 | 5 | 53 | 16 | 33 | 13 | 36 | 1.88 |
|  | 138623--138695 | 36 | 2 | 36 | 94 | 5 | 130 | 35 | 20 | 35 | 8 | 1.83 |
|  | 138742--138863 | 24 | 4.7 | 24 | 72 | 23 | 113 | 29 | 17 | 34 | 18 | 1.94 |
|  | 138729--138839 | 57 | 1.9 | 57 | 100 | 0 | 222 | 29 | 17 | 34 | 18 | 1.94 |
|  | 138742--138925 | 81 | 2.2 | 83 | 95 | 4 | 327 | 29 | 17 | 34 | 18 | 1.94 |
|  | 138823--138960 | 62 | 2.3 | 59 | 93 | 6 | 235 | 29 | 17 | 35 | 17 | 1.93 |
|  | 138964--138988 | 12 | 2.1 | 12 | 100 | 0 | 50 | 52 | 0 | 40 | 8 | 1.31 |
|  | 139831--139939 | 33 | 3.3 | 33 | 97 | 0 | 209 | 23 | 20 | 38 | 17 | 1.93 |
|  | 140049--140086 | 9 | 4.2 | 9 | 93 | 0 | 67 | 13 | 2 | 44 | 39 | 1.57 |
|  | 144310--144338 | 14 | 2.1 | 14 | 100 | 0 | 58 | 20 | 13 | 34 | 31 | 1.92 |
|  | 148737--148879 | 63 | 2.3 | 63 | 100 | 0 | 286 | 27 | 34 | 8 | 30 | 1.86 |
|  | 155657--155825 | 32 | 5.3 | 32 | 100 | 0 | 338 | 8 | 44 | 17 | 28 | 1.79 |
|  | 156587--156663 | 30 | 2.6 | 30 | 100 | 0 | 154 | 12 | 37 | 14 | 35 | 1.84 |
|  | 156569--156671 | 15 | 6.6 | 15 | 76 | 13 | 93 | 13 | 37 | 14 | 33 | 1.86 |
|  | 161427--161459 | 17 | 1.9 | 17 | 93 | 0 | 57 | 27 | 24 | 24 | 24 | 2 |
|  | 162990--163044 | 18 | 3.1 | 18 | 75 | 0 | 65 | 32 | 10 | 38 | 18 | 1.85 |
|  | 164671--164792 | 32 | 3.9 | 32 | 70 | 12 | 108 | 27 | 11 | 29 | 31 | 1.92 |
|  | 171329--171365 | 17 | 2.2 | 17 | 95 | 0 | 65 | 37 | 35 | 27 | 0 | 1.57 |
|  | 171365--171399 | 17 | 2.1 | 17 | 100 | 0 | 70 | 42 | 11 | 28 | 17 | 1.83 |
|  | 172597--172626 | 15 | 2 | 15 | 93 | 0 | 51 | 36 | 13 | 33 | 16 | 1.88 |
|  | 172714--172751 | 20 | 1.9 | 20 | 88 | 0 | 58 | 44 | 0 | 39 | 15 | 1.47 |
|  | 172764--172837 | 36 | 2.1 | 36 | 94 | 5 | 132 | 35 | 20 | 36 | 8 | 1.82 |
|  | 172845--172915 | 35 | 2 | 35 | 100 | 0 | 142 | 33 | 14 | 40 | 11 | 1.81 |
|  | 172845--172926 | 35 | 2.5 | 33 | 90 | 9 | 107 | 34 | 14 | 41 | 9 | 1.79 |
|  | 172951--172979 | 15 | 1.9 | 15 | 100 | 0 | 58 | 37 | 13 | 34 | 13 | 1.85 |
|  | 177569--177609 | 18 | 2.3 | 18 | 100 | 0 | 82 | 17 | 41 | 9 | 31 | 1.82 |
|  | 177657--177858 | 30 | 6.8 | 30 | 93 | 1 | 336 | 23 | 35 | 8 | 32 | 1.86 |
|  | 177657--177879 | 89 | 2.5 | 89 | 90 | 2 | 367 | 23 | 34 | 8 | 32 | 1.86 |
|  | 177657--177879 | 59 | 3.8 | 59 | 94 | 1 | 401 | 23 | 34 | 8 | 32 | 1.86 |
|  | 177936--177965 | 15 | 2 | 15 | 93 | 0 | 51 | 16 | 33 | 13 | 36 | 1.88 |
|  | 179910--179936 | 12 | 2.2 | 12 | 100 | 0 | 54 | 33 | 14 | 37 | 14 | 1.88 |
|  | 180710--180822 | 30 | 3.8 | 30 | 95 | 1 | 192 | 23 | 35 | 8 | 32 | 1.86 |
|  | 180710--180843 | 59 | 2.3 | 59 | 88 | 2 | 189 | 23 | 34 | 8 | 32 | 1.86 |
|  | 181447--181507 | 18 | 3.4 | 18 | 86 | 8 | 88 | 16 | 39 | 9 | 34 | 1.82 |
|  | 181517--181557 | 21 | 2 | 21 | 100 | 0 | 82 | 21 | 34 | 24 | 19 | 1.97 |
|  | 181537--181566 | 15 | 2 | 15 | 93 | 0 | 51 | 23 | 33 | 23 | 20 | 1.97 |
|  | 181601--181630 | 15 | 2 | 15 | 93 | 0 | 51 | 16 | 33 | 13 | 36 | 1.88 |
|  | 184163--184192 | 15 | 2 | 15 | 93 | 0 | 51 | 16 | 33 | 13 | 36 | 1.88 |
|  | 186497--186527 | 15 | 2.1 | 15 | 93 | 0 | 53 | 16 | 35 | 12 | 35 | 1.87 |
|  | 188065--188110 | 12 | 3.8 | 12 | 81 | 13 | 58 | 30 | 17 | 34 | 17 | 1.93 |

**Supplementary Table S4** Dispersed repeat sequences in the mitochondrial genome of *Z. caespitosa*.

| **ID** | **Length** | **Type** | **Seq_A_id** | **Repeat_A_start** | **Repeat_A_end** | **Seq_B_id** | **Repeat_B_start** | **Repeat_B_end** | **Hamming_Distance** | **E-value** | **Gene_A** | **Gene_B** |
| --- | --- | --- | --- | --- | --- | --- | --- | --- | --- | --- | --- | --- |
| R1 | 4442 | P | *Z. caespitosa* | 21660 | 26101 | *Z. caespitosa* | 114924 | 119365 | 0 | 0.00E+00 | *orf340a* | *atp6;orf540a-1;orf104d;orf148c* |
| R2 | 4209 | P | *Z. caespitosa* | 93304 | 97512 | *Z. caespitosa* | 114243 | 118451 | 0 | 0.00E+00 | *_* | *_* |
| R3 | 3528 | F | *Z. caespitosa* | 22574 | 26101 | *Z. caespitosa* | 93304 | 96831 | 0 | 0.00E+00 | *orf340a* | *orf540a-2;atp6* |
| R4 | 2864 | P | *Z. caespitosa* | 97508 | 100371 | *Z. caespitosa* | 111385 | 114248 | 0 | 0.00E+00 | *cob;orf332a* | *_* |
| R5 | 1794 | F | *Z. caespitosa* | 2395 | 4188 | *Z. caespitosa* | 44341 | 46134 | 0 | 0.00E+00 | *_* | *_* |
| R6 | 1166 | F | *Z. caespitosa* | 833 | 1998 | *Z. caespitosa* | 42781 | 43946 | 0 | 0.00E+00 | *_* | *_* |
| R7 | 1005 | P | *Z. caespitosa* | 105214 | 106218 | *Z. caespitosa* | 170096 | 171100 | 0 | 0.00E+00 | *orf271a* | *orf509a;atp1* |
| R8 | 576 | P | *Z. caespitosa* | 21090 | 21665 | *Z. caespitosa* | 119361 | 119936 | 0 | 0.00E+00 | *_* | *orf104d;nad5;orf399a;orf148c* |
| R9 | 516 | F | *Z. caespitosa* | 4519 | 5034 | *Z. caespitosa* | 46140 | 46655 | 0 | 0.00E+00 | *mttB* | *_* |
| R10 | 430 | F | *Z. caespitosa* | 9163 | 9592 | *Z. caespitosa* | 9227 | 9656 | -3 | 1.92E-240 | *orf397a* | *orf397a* |
| R11 | 408 | F | *Z. caespitosa* | 1993 | 2400 | *Z. caespitosa* | 43940 | 44347 | 0 | 9.51E-236 | *_* | *_* |
| R12 | 398 | F | *Z. caespitosa* | 9165 | 9562 | *Z. caespitosa* | 9421 | 9818 | -3 | 2.81E-221 | *orf397a* | *orf397a* |
| R13 | 384 | F | *Z. caespitosa* | 456 | 839 | *Z. caespitosa* | 42405 | 42788 | 0 | 2.68E-221 | *_* | *_* |
| R14 | 376 | F | *Z. caespitosa* | 16218 | 16593 | *Z. caespitosa* | 16251 | 16626 | -1 | 1.98E-213 | *orf284a* | *orf284a* |
| R15 | 366 | F | *Z. caespitosa* | 9163 | 9528 | *Z. caespitosa* | 9291 | 9656 | -3 | 4.03E-202 | *orf397a* | *orf397a* |
| R16 | 343 | F | *Z. caespitosa* | 4083 | 4425 | *Z. caespitosa* | 83537 | 83879 | 0 | 1.30E-196 | *_* | *_* |
| R17 | 343 | F | *Z. caespitosa* | 16218 | 16560 | *Z. caespitosa* | 16284 | 16626 | -1 | 1.33E-193 | *orf284a* | *orf284a* |
| R18 | 339 | P | *Z. caespitosa* | 87037 | 87375 | *Z. caespitosa* | 109092 | 109430 | 0 | 3.32E-194 | *orf116a;orf122a;nad7* | *orf161a* |
| R19 | 326 | F | *Z. caespitosa* | 4224 | 4549 | *Z. caespitosa* | 83589 | 83914 | 0 | 2.22E-186 | *_* | *_* |
| R20 | 320 | F | *Z. caespitosa* | 83736 | 84055 | *Z. caespitosa* | 177715 | 178034 | 0 | 9.11E-183 | *_* | *_* |
| R21 | 310 | F | *Z. caespitosa* | 16218 | 16527 | *Z. caespitosa* | 16317 | 16626 | -1 | 8.89E-174 | *orf284a* | *orf284a* |
| R22 | 302 | F | *Z. caespitosa* | 9163 | 9464 | *Z. caespitosa* | 9355 | 9656 | -3 | 7.69E-164 | *orf397a* | *orf397a* |
| R23 | 291 | F | *Z. caespitosa* | 4135 | 4425 | *Z. caespitosa* | 4224 | 4514 | 0 | 2.63E-165 | *_* | *_* |
| R24 | 277 | F | *Z. caespitosa* | 9143 | 9419 | *Z. caespitosa* | 9335 | 9611 | -3 | 6.67E-149 | *orf397a* | *orf397a* |
| R25 | 277 | F | *Z. caespitosa* | 9143 | 9419 | *Z. caespitosa* | 9271 | 9547 | -3 | 6.67E-149 | *orf397a* | *orf397a* |
| R26 | 277 | F | *Z. caespitosa* | 9143 | 9419 | *Z. caespitosa* | 9207 | 9483 | -3 | 6.67E-149 | *orf397a* | *orf397a* |
| R27 | 277 | F | *Z. caespitosa* | 16218 | 16494 | *Z. caespitosa* | 16350 | 16626 | -1 | 5.86E-154 | *orf284a* | *orf284a* |
| R28 | 277 | P | *Z. caespitosa* | 75696 | 75972 | *Z. caespitosa* | 172525 | 172801 | 0 | 7.05E-157 | *_* | *orf185a* |
| R29 | 257 | F | *Z. caespitosa* | 9163 | 9419 | *Z. caespitosa* | 9483 | 9739 | -3 | 5.85E-137 | *orf397a* | *orf397a* |
| R30 | 255 | F | *Z. caespitosa* | 9229 | 9483 | *Z. caespitosa* | 9421 | 9675 | -3 | 9.15E-136 | *orf397a* | *orf397a* |
| R31 | 255 | F | *Z. caespitosa* | 9293 | 9547 | *Z. caespitosa* | 9421 | 9675 | -3 | 9.15E-136 | *orf397a* | *orf397a* |
| R32 | 255 | F | *Z. caespitosa* | 9357 | 9611 | *Z. caespitosa* | 9421 | 9675 | -3 | 9.15E-136 | *orf397a* | *orf397a* |
| R33 | 247 | F | *Z. caespitosa* | 9143 | 9389 | *Z. caespitosa* | 9399 | 9645 | -3 | 5.45E-131 | *orf397a* | *orf397a* |
| R34 | 244 | F | *Z. caespitosa* | 16218 | 16461 | *Z. caespitosa* | 16383 | 16626 | -1 | 3.81E-134 | *orf284a* | *orf284a* |
| R35 | 225 | F | *Z. caespitosa* | 9421 | 9645 | *Z. caespitosa* | 9485 | 9709 | -3 | 7.23E-118 | *orf397a* | *orf397a* |
| R36 | 211 | F | *Z. caespitosa* | 16218 | 16428 | *Z. caespitosa* | 16416 | 16626 | -1 | 2.43E-114 | *orf284a* | *orf284a* |
| R37 | 208 | F | *Z. caespitosa* | 9163 | 9370 | *Z. caespitosa* | 9547 | 9754 | -3 | 9.81E-108 | *orf397a* | *orf397a* |
| R38 | 206 | F | *Z. caespitosa* | 9421 | 9626 | *Z. caespitosa* | 9549 | 9754 | -3 | 1.52E-106 | *orf397a* | *orf397a* |
| R39 | 202 | F | *Z. caespitosa* | 4135 | 4336 | *Z. caespitosa* | 83678 | 83879 | 0 | 1.01E-111 | *_* | *_* |
| R40 | 202 | F | *Z. caespitosa* | 4135 | 4336 | *Z. caespitosa* | 4313 | 4514 | 0 | 1.01E-111 | *_* | *_* |
| R41 | 202 | F | *Z. caespitosa* | 83589 | 83790 | *Z. caespitosa* | 83678 | 83879 | 0 | 1.01E-111 | *_* | *_* |
| R42 | 202 | F | *Z. caespitosa* | 4313 | 4514 | *Z. caespitosa* | 83589 | 83790 | 0 | 1.01E-111 | *_* | *_* |
| R43 | 199 | F | *Z. caespitosa* | 9391 | 9589 | *Z. caespitosa* | 9647 | 9845 | -3 | 2.25E-102 | *orf397a* | *orf397a* |
| R44 | 194 | F | *Z. caespitosa* | 9143 | 9336 | *Z. caespitosa* | 9463 | 9656 | -3 | 2.13E-99 | *orf397a* | *orf397a* |
| R45 | 188 | F | *Z. caespitosa* | 9402 | 9589 | *Z. caespitosa* | 9658 | 9845 | -2 | 4.27E-98 | *orf397a* | *orf397a* |
| R46 | 188 | F | *Z. caespitosa* | 9210 | 9397 | *Z. caespitosa* | 9658 | 9845 | -3 | 7.95E-96 | *orf397a* | *orf397a* |
| R47 | 184 | P | *Z. caespitosa* | 71986 | 72169 | *Z. caespitosa* | 108896 | 109079 | -1 | 3.82E-98 | *_* | *orf161a* |
| R48 | 183 | F | *Z. caespitosa* | 7663 | 7845 | *Z. caespitosa* | 13894 | 14076 | -3 | 7.50E-93 | *_* | *_* |
| R49 | 180 | F | *Z. caespitosa* | 9466 | 9645 | *Z. caespitosa* | 9658 | 9837 | -3 | 4.57E-91 | *orf397a* | *orf397a* |
| R50 | 180 | P | *Z. caespitosa* | 123211 | 123390 | *Z. caespitosa* | 148915 | 149094 | -1 | 9.56E-96 | *_* | *_* |
| R51 | 179 | F | *Z. caespitosa* | 4371 | 4549 | *Z. caespitosa* | 177715 | 177893 | 0 | 7.08E-98 | *_* | *_* |
| R52 | 178 | F | *Z. caespitosa* | 16218 | 16395 | *Z. caespitosa* | 16449 | 16626 | -1 | 1.51E-94 | *orf284a* | *orf284a* |
| R53 | 172 | F | *Z. caespitosa* | 9199 | 9370 | *Z. caespitosa* | 9647 | 9818 | -3 | 2.61E-86 | *orf397a* | *orf397a* |
| R54 | 172 | F | *Z. caespitosa* | 9455 | 9626 | *Z. caespitosa* | 9647 | 9818 | -3 | 2.61E-86 | *orf397a* | *orf397a* |
| R55 | 169 | F | *Z. caespitosa* | 9293 | 9461 | *Z. caespitosa* | 9677 | 9845 | -3 | 1.58E-84 | *orf397a* | *orf397a* |
| R56 | 169 | F | *Z. caespitosa* | 9229 | 9397 | *Z. caespitosa* | 9677 | 9845 | -2 | 9.49E-87 | *orf397a* | *orf397a* |
| R57 | 169 | F | *Z. caespitosa* | 9165 | 9333 | *Z. caespitosa* | 9677 | 9845 | -2 | 9.49E-87 | *orf397a* | *orf397a* |
| R58 | 169 | F | *Z. caespitosa* | 9485 | 9653 | *Z. caespitosa* | 9677 | 9845 | -3 | 1.58E-84 | *orf397a* | *orf397a* |
| R59 | 169 | F | *Z. caespitosa* | 9357 | 9525 | *Z. caespitosa* | 9677 | 9845 | -3 | 1.58E-84 | *orf397a* | *orf397a* |
| R60 | 168 | F | *Z. caespitosa* | 77917 | 78084 | *Z. caespitosa* | 85227 | 85394 | 0 | 2.97E-91 | *_* | *orf100a* |
| R61 | 168 | F | *Z. caespitosa* | 9957 | 10124 | *Z. caespitosa* | 10083 | 10250 | -3 | 6.22E-84 | *orf397a* | *orf397a* |
| R62 | 163 | F | *Z. caespitosa* | 55738 | 55900 | *Z. caespitosa* | 160291 | 160453 | -1 | 1.49E-85 | *rrn18;orf124a* | *_* |
| R63 | 161 | F | *Z. caespitosa* | 9338 | 9498 | *Z. caespitosa* | 9658 | 9818 | -3 | 8.97E-80 | *orf397a* | *orf397a* |
| R64 | 161 | F | *Z. caespitosa* | 9274 | 9434 | *Z. caespitosa* | 9658 | 9818 | -3 | 8.97E-80 | *orf397a* | *orf397a* |
| R65 | 161 | F | *Z. caespitosa* | 177609 | 177769 | *Z. caespitosa* | 180662 | 180822 | 0 | 4.87E-87 | *_* | *_* |
| R66 | 161 | F | *Z. caespitosa* | 294 | 454 | *Z. caespitosa* | 141034 | 141194 | -1 | 2.35E-84 | *_* | *matR* |
| R67 | 161 | P | *Z. caespitosa* | 135046 | 135206 | *Z. caespitosa* | 162872 | 163032 | 0 | 4.87E-87 | *_* | *_* |
| R68 | 157 | F | *Z. caespitosa* | 9263 | 9419 | *Z. caespitosa* | 9647 | 9803 | -3 | 2.13E-77 | *orf397a* | *orf397a* |
| R69 | 151 | F | *Z. caespitosa* | 25785 | 25935 | *Z. caespitosa* | 176228 | 176378 | -3 | 7.75E-74 | *orf340a* | *orf110b* |
| R70 | 151 | F | *Z. caespitosa* | 96515 | 96665 | *Z. caespitosa* | 176228 | 176378 | -3 | 7.75E-74 | *orf540a-2* | *orf110b* |
| R71 | 151 | P | *Z. caespitosa* | 115090 | 115240 | *Z. caespitosa* | 176228 | 176378 | -3 | 7.75E-74 | *orf540a-1* | *orf110b* |
| R72 | 149 | F | *Z. caespitosa* | 136847 | 136995 | *Z. caespitosa* | 177887 | 178035 | 0 | 8.16E-80 | *_* | *_* |
| R73 | 149 | F | *Z. caespitosa* | 7705 | 7853 | *Z. caespitosa* | 13936 | 14084 | -3 | 1.19E-72 | *_* | *_* |
| R74 | 148 | F | *Z. caespitosa* | 83908 | 84055 | *Z. caespitosa* | 136847 | 136994 | 0 | 3.27E-79 | *_* | *_* |
| R75 | 145 | F | *Z. caespitosa* | 16218 | 16362 | *Z. caespitosa* | 16482 | 16626 | -1 | 9.09E-75 | *orf284a* | *orf284a* |
| R76 | 144 | F | *Z. caespitosa* | 4193 | 4336 | *Z. caespitosa* | 177715 | 177858 | 0 | 8.36E-77 | *_* | *_* |
| R77 | 144 | F | *Z. caespitosa* | 83647 | 83790 | *Z. caespitosa* | 177715 | 177858 | 0 | 8.36E-77 | *_* | *_* |
| R78 | 144 | F | *Z. caespitosa* | 4282 | 4425 | *Z. caespitosa* | 177715 | 177858 | 0 | 8.36E-77 | *_* | *_* |
| R79 | 144 | F | *Z. caespitosa* | 7520 | 7663 | *Z. caespitosa* | 15887 | 16030 | 0 | 8.36E-77 | *_* | *orf284a* |
| R80 | 144 | F | *Z. caespitosa* | 9163 | 9306 | *Z. caespitosa* | 9611 | 9754 | -3 | 1.10E-69 | *orf397a* | *orf397a* |
| R81 | 144 | P | *Z. caespitosa* | 31924 | 32067 | *Z. caespitosa* | 173969 | 174112 | -1 | 3.61E-74 | *orf127a* | *_* |
| R82 | 142 | F | *Z. caespitosa* | 9421 | 9562 | *Z. caespitosa* | 9613 | 9754 | -3 | 1.69E-68 | *orf397a* | *orf397a* |
| R83 | 142 | P | *Z. caespitosa* | 136989 | 137130 | *Z. caespitosa* | 164661 | 164802 | 0 | 1.34E-75 | *_* | *_* |
| R84 | 140 | F | *Z. caespitosa* | 136845 | 136984 | *Z. caespitosa* | 181550 | 181689 | 0 | 2.14E-74 | *_* | *orf103d* |
| R85 | 140 | P | *Z. caespitosa* | 90078 | 90217 | *Z. caespitosa* | 121738 | 121877 | 0 | 2.14E-74 | *orf118d* | *orf153a* |
| R86 | 138 | F | *Z. caespitosa* | 177887 | 178024 | *Z. caespitosa* | 181552 | 181689 | 0 | 3.42E-73 | *_* | *orf103d* |
| R87 | 138 | F | *Z. caespitosa* | 83908 | 84045 | *Z. caespitosa* | 181552 | 181689 | 0 | 3.42E-73 | *_* | *orf103d* |
| R88 | 137 | F | *Z. caespitosa* | 155657 | 155793 | *Z. caespitosa* | 155689 | 155825 | 0 | 1.37E-72 | *_* | *_* |
| R89 | 136 | F | *Z. caespitosa* | 9989 | 10124 | *Z. caespitosa* | 10115 | 10250 | -2 | 4.53E-67 | *orf397a* | *orf397a* |
| R90 | 135 | F | *Z. caespitosa* | 10311 | 10445 | *Z. caespitosa* | 10752 | 10886 | -3 | 2.37E-64 | *_* | *_* |
| R91 | 135 | F | *Z. caespitosa* | 9647 | 9781 | *Z. caespitosa* | 9711 | 9845 | -3 | 2.37E-64 | *orf397a* | *orf397a* |
| R92 | 134 | F | *Z. caespitosa* | 4165 | 4298 | *Z. caespitosa* | 180710 | 180843 | -1 | 3.52E-68 | *_* | *_* |
| R93 | 134 | F | *Z. caespitosa* | 83619 | 83752 | *Z. caespitosa* | 180710 | 180843 | -1 | 3.52E-68 | *_* | *_* |
| R94 | 134 | F | *Z. caespitosa* | 4254 | 4387 | *Z. caespitosa* | 180710 | 180843 | -1 | 3.52E-68 | *_* | *_* |
| R95 | 134 | F | *Z. caespitosa* | 83708 | 83841 | *Z. caespitosa* | 180710 | 180843 | -1 | 3.52E-68 | *_* | *_* |
| R96 | 134 | F | *Z. caespitosa* | 4343 | 4476 | *Z. caespitosa* | 180710 | 180843 | -1 | 3.52E-68 | *_* | *_* |
| R97 | 130 | F | *Z. caespitosa* | 89958 | 90087 | *Z. caespitosa* | 105624 | 105753 | 0 | 2.24E-68 | *_* | *orf271a* |
| R98 | 130 | F | *Z. caespitosa* | 9143 | 9272 | *Z. caespitosa* | 9527 | 9656 | -3 | 2.17E-61 | *orf397a* | *orf397a* |
| R99 | 130 | P | *Z. caespitosa* | 89958 | 90087 | *Z. caespitosa* | 170561 | 170690 | 0 | 2.24E-68 | *_* | *orf509a;atp1* |
| R100 | 127 | F | *Z. caespitosa* | 9530 | 9656 | *Z. caespitosa* | 9658 | 9784 | -3 | 1.29E-59 | *orf397a* | *orf397a* |
| R101 | 127 | F | *Z. caespitosa* | 9519 | 9645 | *Z. caespitosa* | 9647 | 9773 | -3 | 1.29E-59 | *orf397a* | *orf397a* |
| R102 | 127 | F | *Z. caespitosa* | 9549 | 9675 | *Z. caespitosa* | 9677 | 9803 | -3 | 1.29E-59 | *orf397a* | *orf397a* |
| R103 | 126 | F | *Z. caespitosa* | 10325 | 10450 | *Z. caespitosa* | 10766 | 10891 | -3 | 5.05E-59 | *_* | *_* |
| R104 | 125 | F | *Z. caespitosa* | 1480 | 1604 | *Z. caespitosa* | 46002 | 46126 | -1 | 8.62E-63 | *_* | *_* |
| R105 | 125 | F | *Z. caespitosa* | 1480 | 1604 | *Z. caespitosa* | 4056 | 4180 | -1 | 8.62E-63 | *_* | *_* |
| R106 | 125 | F | *Z. caespitosa* | 43428 | 43552 | *Z. caespitosa* | 46002 | 46126 | -1 | 8.62E-63 | *_* | *_* |
| R107 | 125 | F | *Z. caespitosa* | 4056 | 4180 | *Z. caespitosa* | 43428 | 43552 | -1 | 8.62E-63 | *_* | *_* |
| R108 | 124 | F | *Z. caespitosa* | 9658 | 9781 | *Z. caespitosa* | 9722 | 9845 | -2 | 6.31E-60 | *orf397a* | *orf397a* |
| R109 | 123 | F | *Z. caespitosa* | 7736 | 7858 | *Z. caespitosa* | 13967 | 14089 | -3 | 3.00E-57 | *_* | *_* |
| R110 | 122 | F | *Z. caespitosa* | 99439 | 99560 | *Z. caespitosa* | 166716 | 166837 | 0 | 1.47E-63 | *orf113c-2* | *_* |
| R111 | 122 | P | *Z. caespitosa* | 112196 | 112317 | *Z. caespitosa* | 166716 | 166837 | 0 | 1.47E-63 | *orf113c-1* | *_* |
| R112 | 120 | F | *Z. caespitosa* | 77670 | 77789 | *Z. caespitosa* | 77732 | 77851 | 0 | 2.35E-62 | *_* | *_* |
| R113 | 120 | F | *Z. caespitosa* | 37880 | 37999 | *Z. caespitosa* | 108762 | 108881 | 0 | 2.35E-62 | *_* | *orf161a* |
| R114 | 119 | F | *Z. caespitosa* | 114249 | 114367 | *Z. caespitosa* | 185008 | 185126 | -1 | 3.36E-59 | *atp6;orf540a-1* | *orf131c* |
| R115 | 119 | F | *Z. caespitosa* | 26098 | 26216 | *Z. caespitosa* | 96829 | 96947 | 0 | 9.41E-62 | *orf340a* | *orf540a-2;atp6* |
| R116 | 119 | F | *Z. caespitosa* | 8172 | 8290 | *Z. caespitosa* | 14403 | 14521 | -3 | 6.96E-55 | *_* | *orf113a* |
| R117 | 119 | P | *Z. caespitosa* | 97388 | 97506 | *Z. caespitosa* | 185008 | 185126 | -1 | 3.36E-59 | *orf540a-2;atp6* | *orf131c* |
| R118 | 119 | P | *Z. caespitosa* | 26098 | 26216 | *Z. caespitosa* | 114808 | 114926 | 0 | 9.41E-62 | *orf340a* | *atp6;orf540a-1* |
| R119 | 119 | P | *Z. caespitosa* | 53107 | 53225 | *Z. caespitosa* | 72186 | 72304 | -2 | 5.95E-57 | *_* | *_* |
| R120 | 116 | F | *Z. caespitosa* | 16095 | 16210 | *Z. caespitosa* | 162728 | 162843 | 0 | 6.02E-60 | *orf284a* | *_* |
| R121 | 116 | P | *Z. caespitosa* | 172512 | 172627 | *Z. caespitosa* | 184162 | 184277 | 0 | 6.02E-60 | *orf185a* | *orf130b* |
| R122 | 115 | F | *Z. caespitosa* | 9155 | 9269 | *Z. caespitosa* | 9731 | 9845 | -3 | 1.61E-52 | *orf397a* | *orf397a* |
| R123 | 113 | F | *Z. caespitosa* | 4135 | 4247 | *Z. caespitosa* | 177746 | 177858 | 0 | 3.86E-58 | *_* | *_* |
| R124 | 113 | F | *Z. caespitosa* | 4135 | 4247 | *Z. caespitosa* | 83767 | 83879 | 0 | 3.86E-58 | *_* | *_* |
| R125 | 113 | F | *Z. caespitosa* | 4135 | 4247 | *Z. caespitosa* | 4402 | 4514 | 0 | 3.86E-58 | *_* | *_* |
| R126 | 113 | F | *Z. caespitosa* | 83589 | 83701 | *Z. caespitosa* | 177746 | 177858 | 0 | 3.86E-58 | *_* | *_* |
| R127 | 113 | F | *Z. caespitosa* | 83589 | 83701 | *Z. caespitosa* | 83767 | 83879 | 0 | 3.86E-58 | *_* | *_* |
| R128 | 113 | F | *Z. caespitosa* | 4402 | 4514 | *Z. caespitosa* | 83589 | 83701 | 0 | 3.86E-58 | *_* | *_* |
| R129 | 113 | F | *Z. caespitosa* | 4165 | 4277 | *Z. caespitosa* | 177657 | 177769 | 0 | 3.86E-58 | *_* | *_* |
| R130 | 113 | F | *Z. caespitosa* | 83619 | 83731 | *Z. caespitosa* | 177657 | 177769 | 0 | 3.86E-58 | *_* | *_* |
| R131 | 113 | F | *Z. caespitosa* | 4254 | 4366 | *Z. caespitosa* | 177657 | 177769 | 0 | 3.86E-58 | *_* | *_* |
| R132 | 113 | F | *Z. caespitosa* | 83708 | 83820 | *Z. caespitosa* | 177657 | 177769 | 0 | 3.86E-58 | *_* | *_* |
| R133 | 113 | F | *Z. caespitosa* | 4343 | 4455 | *Z. caespitosa* | 177657 | 177769 | 0 | 3.86E-58 | *_* | *_* |
| R134 | 112 | F | *Z. caespitosa* | 16218 | 16329 | *Z. caespitosa* | 16515 | 16626 | -1 | 5.18E-55 | *orf284a* | *orf284a* |
| R135 | 111 | F | *Z. caespitosa* | 25825 | 25935 | *Z. caespitosa* | 176268 | 176378 | 0 | 6.17E-57 | *orf340a* | *orf110b* |
| R136 | 111 | F | *Z. caespitosa* | 96555 | 96665 | *Z. caespitosa* | 176268 | 176378 | 0 | 6.17E-57 | *orf540a-2* | *orf110b* |
| R137 | 109 | F | *Z. caespitosa* | 8182 | 8290 | *Z. caespitosa* | 14413 | 14521 | -2 | 5.23E-51 | *_* | *orf113a* |
| R138 | 109 | F | *Z. caespitosa* | 56514 | 56622 | *Z. caespitosa* | 73862 | 73970 | -3 | 5.59E-49 | *rrn18* | *_* |
| R139 | 109 | P | *Z. caespitosa* | 77775 | 77883 | *Z. caespitosa* | 136895 | 137003 | -2 | 5.23E-51 | *_* | *_* |
| R140 | 108 | F | *Z. caespitosa* | 9327 | 9434 | *Z. caespitosa* | 9647 | 9754 | -3 | 2.18E-48 | *orf397a* | *orf397a* |
| R141 | 107 | P | *Z. caespitosa* | 72182 | 72288 | *Z. caespitosa* | 84233 | 84339 | -2 | 8.06E-50 | *_* | *_* |
| R142 | 106 | F | *Z. caespitosa* | 177715 | 177820 | *Z. caespitosa* | 180738 | 180843 | -1 | 2.01E-51 | *_* | *_* |
| R143 | 106 | F | *Z. caespitosa* | 46029 | 46134 | *Z. caespitosa* | 83537 | 83642 | 0 | 6.32E-54 | *_* | *_* |
| R144 | 106 | P | *Z. caespitosa* | 148692 | 148797 | *Z. caespitosa* | 163006 | 163111 | -1 | 2.01E-51 | *_* | *_* |
| R145 | 105 | F | *Z. caespitosa* | 155657 | 155761 | *Z. caespitosa* | 155721 | 155825 | 0 | 2.53E-53 | *_* | *_* |
| R146 | 105 | F | *Z. caespitosa* | 9421 | 9525 | *Z. caespitosa* | 9741 | 9845 | -2 | 1.24E-48 | *orf397a* | *orf397a* |
| R147 | 105 | F | *Z. caespitosa* | 9677 | 9781 | *Z. caespitosa* | 9741 | 9845 | -1 | 7.96E-51 | *orf397a* | *orf397a* |
| R148 | 105 | P | *Z. caespitosa* | 52882 | 52986 | *Z. caespitosa* | 101709 | 101813 | 0 | 2.53E-53 | *orf172a* | *_* |
| R149 | 104 | F | *Z. caespitosa* | 52765 | 52868 | *Z. caespitosa* | 57548 | 57651 | 0 | 1.01E-52 | *orf172a* | *orf117a* |
| R150 | 104 | P | *Z. caespitosa* | 123287 | 123390 | *Z. caespitosa* | 148915 | 149018 | 0 | 1.01E-52 | *_* | *_* |
| R151 | 103 | F | *Z. caespitosa* | 75870 | 75972 | *Z. caespitosa* | 184162 | 184264 | 0 | 4.04E-52 | *_* | *orf130b* |
| R152 | 103 | F | *Z. caespitosa* | 53123 | 53225 | *Z. caespitosa* | 84233 | 84335 | 0 | 4.04E-52 | *_* | *_* |
| R153 | 102 | F | *Z. caespitosa* | 181600 | 181701 | *Z. caespitosa* | 184162 | 184263 | 0 | 1.62E-51 | *orf103d* | *orf130b* |
| R154 | 102 | F | *Z. caespitosa* | 75870 | 75971 | *Z. caespitosa* | 181600 | 181701 | 0 | 1.62E-51 | *_* | *orf103d* |
| R155 | 102 | P | *Z. caespitosa* | 172526 | 172627 | *Z. caespitosa* | 181600 | 181701 | 0 | 1.62E-51 | *orf185a* | *orf103d* |
| R156 | 102 | P | *Z. caespitosa* | 77782 | 77883 | *Z. caespitosa* | 177935 | 178036 | 0 | 1.62E-51 | *_* | *_* |
| R157 | 102 | P | *Z. caespitosa* | 57565 | 57666 | *Z. caespitosa* | 69881 | 69982 | 0 | 1.62E-51 | *orf117a* | *_* |
| R158 | 100 | F | *Z. caespitosa* | 9143 | 9242 | *Z. caespitosa* | 9719 | 9818 | -3 | 1.13E-43 | *orf397a* | *orf397a* |
| R159 | 100 | P | *Z. caespitosa* | 77784 | 77883 | *Z. caespitosa* | 83956 | 84055 | 0 | 2.59E-50 | *_* | *_* |
| R160 | 98 | F | *Z. caespitosa* | 1507 | 1604 | *Z. caespitosa* | 83537 | 83634 | -1 | 1.22E-46 | *_* | *_* |
| R161 | 98 | F | *Z. caespitosa* | 43455 | 43552 | *Z. caespitosa* | 83537 | 83634 | -1 | 1.22E-46 | *_* | *_* |
| R162 | 97 | F | *Z. caespitosa* | 138623 | 138719 | *Z. caespitosa* | 172765 | 172861 | -1 | 4.82E-46 | *_* | *orf185a* |
| R163 | 97 | F | *Z. caespitosa* | 9146 | 9242 | *Z. caespitosa* | 9658 | 9754 | -3 | 6.59E-42 | *orf397a* | *orf397a* |
| R164 | 97 | P | *Z. caespitosa* | 52646 | 52742 | *Z. caespitosa* | 188071 | 188167 | 0 | 1.66E-48 | *orf172a* | *_* |
| R165 | 97 | P | *Z. caespitosa* | 72162 | 72258 | *Z. caespitosa* | 108806 | 108902 | -1 | 4.82E-46 | *_* | *orf161a* |
| R166 | 96 | P | *Z. caespitosa* | 108715 | 108810 | *Z. caespitosa* | 162992 | 163087 | -2 | 2.72E-43 | *orf161a* | *_* |
| R167 | 95 | F | *Z. caespitosa* | 79619 | 79713 | *Z. caespitosa* | 162961 | 163055 | -1 | 7.55E-45 | *_* | *_* |
| R168 | 95 | P | *Z. caespitosa* | 83529 | 83623 | *Z. caespitosa* | 188179 | 188273 | 0 | 2.65E-47 | *_* | *_* |
| R169 | 94 | F | *Z. caespitosa* | 83519 | 83612 | *Z. caespitosa* | 177587 | 177680 | 0 | 1.06E-46 | *_* | *_* |
| R170 | 94 | F | *Z. caespitosa* | 124633 | 124726 | *Z. caespitosa* | 159217 | 159310 | -2 | 4.17E-42 | *_* | *rrn26* |
| R171 | 94 | P | *Z. caespitosa* | 52918 | 53011 | *Z. caespitosa* | 179897 | 179990 | 0 | 1.06E-46 | *orf172a* | *_* |
| R172 | 93 | F | *Z. caespitosa* | 56540 | 56632 | *Z. caespitosa* | 73888 | 73980 | -2 | 1.63E-41 | *rrn18* | *_* |
| R173 | 93 | P | *Z. caespitosa* | 111299 | 111391 | *Z. caespitosa* | 161804 | 161896 | 0 | 4.24E-46 | *_* | *cox3* |
| R174 | 93 | P | *Z. caespitosa* | 72196 | 72288 | *Z. caespitosa* | 84233 | 84325 | -1 | 1.18E-43 | *_* | *_* |
| R175 | 92 | F | *Z. caespitosa* | 8199 | 8290 | *Z. caespitosa* | 14430 | 14521 | 0 | 1.70E-45 | *_* | *orf113a* |
| R176 | 92 | F | *Z. caespitosa* | 10359 | 10450 | *Z. caespitosa* | 10800 | 10891 | -2 | 6.39E-41 | *_* | *_* |
| R177 | 91 | F | *Z. caespitosa* | 115892 | 115982 | *Z. caespitosa* | 122173 | 122263 | 0 | 6.78E-45 | *orf540a-1* | *nad5* |
| R178 | 91 | P | *Z. caespitosa* | 25043 | 25133 | *Z. caespitosa* | 122173 | 122263 | 0 | 6.78E-45 | *orf340a* | *nad5* |
| R179 | 91 | P | *Z. caespitosa* | 95773 | 95863 | *Z. caespitosa* | 122173 | 122263 | 0 | 6.78E-45 | *orf540a-2* | *nad5* |
| R180 | 90 | F | *Z. caespitosa* | 136895 | 136984 | *Z. caespitosa* | 184162 | 184251 | 0 | 2.71E-44 | *_* | *orf130b* |
| R181 | 90 | F | *Z. caespitosa* | 177935 | 178024 | *Z. caespitosa* | 184162 | 184251 | 0 | 2.71E-44 | *_* | *orf130b* |
| R182 | 90 | F | *Z. caespitosa* | 83956 | 84045 | *Z. caespitosa* | 184162 | 184251 | 0 | 2.71E-44 | *_* | *orf130b* |
| R183 | 90 | F | *Z. caespitosa* | 75870 | 75959 | *Z. caespitosa* | 136895 | 136984 | 0 | 2.71E-44 | *_* | *_* |
| R184 | 90 | F | *Z. caespitosa* | 75870 | 75959 | *Z. caespitosa* | 177935 | 178024 | 0 | 2.71E-44 | *_* | *_* |
| R185 | 90 | F | *Z. caespitosa* | 75870 | 75959 | *Z. caespitosa* | 83956 | 84045 | 0 | 2.71E-44 | *_* | *_* |
| R186 | 90 | F | *Z. caespitosa* | 24949 | 25038 | *Z. caespitosa* | 138885 | 138974 | 0 | 2.71E-44 | *_* | *_* |
| R187 | 90 | F | *Z. caespitosa* | 95679 | 95768 | *Z. caespitosa* | 138885 | 138974 | 0 | 2.71E-44 | *_* | *_* |
| R188 | 90 | F | *Z. caespitosa* | 77794 | 77883 | *Z. caespitosa* | 172538 | 172627 | 0 | 2.71E-44 | *_* | *orf185a* |
| R189 | 90 | F | *Z. caespitosa* | 9692 | 9781 | *Z. caespitosa* | 9756 | 9845 | 0 | 2.71E-44 | *orf397a* | *orf397a* |
| R190 | 90 | F | *Z. caespitosa* | 9308 | 9397 | *Z. caespitosa* | 9756 | 9845 | -1 | 7.33E-42 | *orf397a* | *orf397a* |
| R191 | 90 | F | *Z. caespitosa* | 9244 | 9333 | *Z. caespitosa* | 9756 | 9845 | -1 | 7.33E-42 | *orf397a* | *orf397a* |
| R192 | 90 | F | *Z. caespitosa* | 9180 | 9269 | *Z. caespitosa* | 9756 | 9845 | -1 | 7.33E-42 | *orf397a* | *orf397a* |
| R193 | 90 | F | *Z. caespitosa* | 9436 | 9525 | *Z. caespitosa* | 9756 | 9845 | -1 | 7.33E-42 | *orf397a* | *orf397a* |
| R194 | 90 | F | *Z. caespitosa* | 9500 | 9589 | *Z. caespitosa* | 9756 | 9845 | -1 | 7.33E-42 | *orf397a* | *orf397a* |
| R195 | 90 | F | *Z. caespitosa* | 9564 | 9653 | *Z. caespitosa* | 9756 | 9845 | -2 | 9.78E-40 | *orf397a* | *orf397a* |
| R196 | 90 | F | *Z. caespitosa* | 9372 | 9461 | *Z. caespitosa* | 9756 | 9845 | -2 | 9.78E-40 | *orf397a* | *orf397a* |
| R197 | 90 | P | *Z. caespitosa* | 77794 | 77883 | *Z. caespitosa* | 184162 | 184251 | 0 | 2.71E-44 | *_* | *orf130b* |
| R198 | 90 | P | *Z. caespitosa* | 77794 | 77883 | *Z. caespitosa* | 181600 | 181689 | 0 | 2.71E-44 | *_* | *orf103d* |
| R199 | 90 | P | *Z. caespitosa* | 172538 | 172627 | *Z. caespitosa* | 177935 | 178024 | 0 | 2.71E-44 | *orf185a* | *_* |
| R200 | 90 | P | *Z. caespitosa* | 136895 | 136984 | *Z. caespitosa* | 172538 | 172627 | 0 | 2.71E-44 | *_* | *orf185a* |
| R201 | 90 | P | *Z. caespitosa* | 83956 | 84045 | *Z. caespitosa* | 172538 | 172627 | 0 | 2.71E-44 | *_* | *orf185a* |
| R202 | 90 | P | *Z. caespitosa* | 115987 | 116076 | *Z. caespitosa* | 138885 | 138974 | 0 | 2.71E-44 | *_* | *_* |
| R203 | 90 | P | *Z. caespitosa* | 75870 | 75959 | *Z. caespitosa* | 77794 | 77883 | 0 | 2.71E-44 | *_* | *_* |
| R204 | 88 | P | *Z. caespitosa* | 97419 | 97506 | *Z. caespitosa* | 185008 | 185095 | 0 | 4.34E-43 | *orf540a-2;atp6* | *orf131c* |
| R205 | 87 | F | *Z. caespitosa* | 72211 | 72297 | *Z. caespitosa* | 162893 | 162979 | -1 | 4.53E-40 | *_* | *_* |
| R206 | 87 | F | *Z. caespitosa* | 53114 | 53200 | *Z. caespitosa* | 135099 | 135185 | 0 | 1.74E-42 | *_* | *_* |
| R207 | 87 | P | *Z. caespitosa* | 1507 | 1593 | *Z. caespitosa* | 188179 | 188265 | 0 | 1.74E-42 | *_* | *_* |
| R208 | 87 | P | *Z. caespitosa* | 43455 | 43541 | *Z. caespitosa* | 188179 | 188265 | 0 | 1.74E-42 | *_* | *_* |
| R209 | 87 | P | *Z. caespitosa* | 46029 | 46115 | *Z. caespitosa* | 188179 | 188265 | 0 | 1.74E-42 | *_* | *_* |
| R210 | 87 | P | *Z. caespitosa* | 4083 | 4169 | *Z. caespitosa* | 188179 | 188265 | 0 | 1.74E-42 | *_* | *_* |
| R211 | 87 | P | Z. caespitosa | 37912 | 37998 | *Z. caespitosa* | 188069 | 188155 | 0 | 1.74E-42 | *_* | *_* |
| R212 | 87 | P | *Z. caespitosa* | 108794 | 108880 | *Z. caespitosa* | 188069 | 188155 | 0 | 1.74E-42 | *orf161a* | *_* |
| R213 | 87 | P | *Z. caespitosa* | 53114 | 53200 | *Z. caespitosa* | 162893 | 162979 | 0 | 1.74E-42 | *_* | *_* |
| R214 | 87 | P | *Z. caespitosa* | 72211 | 72297 | *Z. caespitosa* | 135099 | 135185 | -1 | 4.53E-40 | *_* | *_* |
| R215 | 87 | P | *Z. caespitosa* | 52782 | 52868 | *Z. caespitosa* | 69896 | 69982 | 0 | 1.74E-42 | *orf172a* | *_* |
| R216 | 86 | F | *Z. caespitosa* | 37911 | 37996 | *Z. caespitosa* | 52926 | 53011 | 0 | 6.95E-42 | *_* | *orf172a* |
| R217 | 86 | F | *Z. caespitosa* | 52926 | 53011 | *Z. caespitosa* | 108793 | 108878 | 0 | 6.95E-42 | *orf172a* | *orf161a* |
| R218 | 86 | F | *Z. caespitosa* | 9913 | 9998 | *Z. caespitosa* | 10165 | 10250 | -3 | 1.92E-35 | *orf397a* | *orf397a* |
| R219 | 86 | F | *Z. caespitosa* | 72599 | 72684 | *Z. caespitosa* | 177953 | 178038 | 0 | 6.95E-42 | *_* | *_* |
| R220 | 86 | F | *Z. caespitosa* | 52658 | 52743 | *Z. caespitosa* | 52927 | 53012 | 0 | 6.95E-42 | *orf172a* | *orf172a* |
| R221 | 86 | P | *Z. caespitosa* | 37911 | 37996 | *Z. caespitosa* | 179897 | 179982 | 0 | 6.95E-42 | *_* | *_* |
| R222 | 86 | P | *Z. caespitosa* | 108793 | 108878 | *Z. caespitosa* | 179897 | 179982 | 0 | 6.95E-42 | *orf161a* | *_* |
| R223 | 86 | P | *Z. caespitosa* | 57632 | 57717 | *Z. caespitosa* | 172799 | 172884 | 0 | 6.95E-42 | *orf117a* | *orf185a* |
| R224 | 85 | F | *Z. caespitosa* | 177685 | 177769 | *Z. caespitosa* | 177715 | 177799 | 0 | 2.78E-41 | *_* | *_* |
| R225 | 85 | F | *Z. caespitosa* | 177804 | 177888 | *Z. caespitosa* | 180768 | 180852 | 0 | 2.78E-41 | *_* | *_* |
| R226 | 85 | F | *Z. caespitosa* | 83825 | 83909 | *Z. caespitosa* | 180768 | 180852 | 0 | 2.78E-41 | *_* | *_* |
| R227 | 85 | F | *Z. caespitosa* | 4460 | 4544 | *Z. caespitosa* | 180768 | 180852 | 0 | 2.78E-41 | *_* | *_* |
| R228 | 85 | F | *Z. caespitosa* | 14796 | 14880 | *Z. caespitosa* | 25221 | 25305 | -2 | 8.93E-37 | *orf295a;cox2* | *orf340a* |
| R229 | 85 | F | *Z. caespitosa* | 14796 | 14880 | *Z. caespitosa* | 95951 | 96035 | -2 | 8.93E-37 | *orf295a;cox2* | *orf540a-2* |
| R230 | 85 | F | *Z. caespitosa* | 179897 | 179981 | *Z. caespitosa* | 188071 | 188155 | 0 | 2.78E-41 | *_* | *_* |
| R231 | 85 | F | *Z. caespitosa* | 14522 | 14606 | *Z. caespitosa* | 16026 | 16110 | 0 | 2.78E-41 | *orf113a* | *orf284a* |
| R232 | 85 | F | *Z. caespitosa* | 37912 | 37996 | *Z. caespitosa* | 52658 | 52742 | 0 | 2.78E-41 | *_* | *orf172a* |
| R233 | 85 | F | *Z. caespitosa* | 52658 | 52742 | *Z. caespitosa* | 108794 | 108878 | 0 | 2.78E-41 | *orf172a* | *orf161a* |
| R234 | 85 | P | *Z. caespitosa* | 52927 | 53011 | *Z. caespitosa* | 188071 | 188155 | 0 | 2.78E-41 | *orf172a* | *_* |
| R235 | 85 | P | *Z. caespitosa* | 52658 | 52742 | *Z. caespitosa* | 179897 | 179981 | 0 | 2.78E-41 | *orf172a* | *_* |
| R236 | 85 | P | *Z. caespitosa* | 14796 | 14880 | *Z. caespitosa* | 115720 | 115804 | -2 | 8.93E-37 | *orf295a;cox2* | *orf540a-1* |
| R237 | 84 | F | *Z. caespitosa* | 57633 | 57716 | *Z. caespitosa* | 75650 | 75733 | -3 | 2.86E-34 | *orf117a* | *_* |
| R238 | 84 | F | *Z. caespitosa* | 75729 | 75812 | *Z. caespitosa* | 148669 | 148752 | -1 | 2.80E-38 | *_* | *_* |
| R239 | 84 | P | *Z. caespitosa* | 177597 | 177680 | *Z. caespitosa* | 188190 | 188273 | 0 | 1.11E-40 | *_* | *_* |
| R240 | 84 | P | *Z. caespitosa* | 75650 | 75733 | *Z. caespitosa* | 172800 | 172883 | -3 | 2.86E-34 | *_* | *orf185a* |
| R241 | 84 | P | *Z. caespitosa* | 148669 | 148752 | *Z. caespitosa* | 172685 | 172768 | -1 | 2.80E-38 | *_* | *orf185a* |
| R242 | 84 | P | *Z. caespitosa* | 72599 | 72682 | *Z. caespitosa* | 77782 | 77865 | 0 | 1.11E-40 | *_* | *_* |
| R243 | 83 | F | *Z. caespitosa* | 177657 | 177739 | *Z. caespitosa* | 177776 | 177858 | 0 | 4.45E-40 | *_* | *_* |
| R244 | 83 | F | *Z. caespitosa* | 83797 | 83879 | *Z. caespitosa* | 177657 | 177739 | 0 | 4.45E-40 | *_* | *_* |
| R245 | 83 | F | *Z. caespitosa* | 4432 | 4514 | *Z. caespitosa* | 177657 | 177739 | 0 | 4.45E-40 | *_* | *_* |
| R246 | 83 | F | *Z. caespitosa* | 177776 | 177858 | *Z. caespitosa* | 180710 | 180792 | 0 | 4.45E-40 | *_* | *_* |
| R247 | 83 | F | *Z. caespitosa* | 83797 | 83879 | *Z. caespitosa* | 180710 | 180792 | 0 | 4.45E-40 | *_* | *_* |
| R248 | 83 | F | *Z. caespitosa* | 4432 | 4514 | *Z. caespitosa* | 180710 | 180792 | 0 | 4.45E-40 | *_* | *_* |
| R249 | 83 | F | *Z. caespitosa* | 77785 | 77867 | *Z. caespitosa* | 79443 | 79525 | -2 | 1.36E-35 | *_* | *_* |
| R250 | 83 | F | *Z. caespitosa* | 72599 | 72681 | *Z. caespitosa* | 136913 | 136995 | 0 | 4.45E-40 | *_* | *_* |
| R251 | 83 | P | *Z. caespitosa* | 79443 | 79525 | *Z. caespitosa* | 177951 | 178033 | -2 | 1.36E-35 | *_* | *_* |
| R252 | 83 | P | *Z. caespitosa* | 79443 | 79525 | *Z. caespitosa* | 136911 | 136993 | -2 | 1.36E-35 | *_* | *_* |
| R253 | 83 | P | *Z. caespitosa* | 79443 | 79525 | *Z. caespitosa* | 83972 | 84054 | -2 | 1.36E-35 | *_* | *_* |
| R254 | 82 | F | *Z. caespitosa* | 124645 | 124726 | *Z. caespitosa* | 159229 | 159310 | -1 | 4.37E-37 | *_* | *rrn26* |
| R255 | 82 | F | *Z. caespitosa* | 9594 | 9675 | *Z. caespitosa* | 9658 | 9739 | -3 | 4.25E-33 | *orf397a* | *orf397a* |
| R256 | 82 | F | *Z. caespitosa* | 24877 | 24958 | *Z. caespitosa* | 162642 | 162723 | -1 | 4.37E-37 | *_* | *_* |
| R257 | 82 | F | *Z. caespitosa* | 95607 | 95688 | *Z. caespitosa* | 162642 | 162723 | -1 | 4.37E-37 | *_* | *_* |
| R258 | 82 | F | *Z. caespitosa* | 69672 | 69753 | *Z. caespitosa* | 127476 | 127557 | -3 | 4.25E-33 | *_* | *orf149b* |
| R259 | 82 | F | *Z. caespitosa* | 108715 | 108796 | *Z. caespitosa* | 148716 | 148797 | -2 | 5.31E-35 | *orf161a* | *_* |
| R260 | 82 | F | *Z. caespitosa* | 9866 | 9947 | *Z. caespitosa* | 9929 | 10010 | -3 | 4.25E-33 | *orf397a* | *orf397a* |
| R261 | 82 | F | *Z. caespitosa* | 69686 | 69767 | *Z. caespitosa* | 127490 | 127571 | -3 | 4.25E-33 | *_* | *orf149b* |
| R262 | 82 | F | *Z. caespitosa* | 72599 | 72680 | *Z. caespitosa* | 83974 | 84055 | 0 | 1.78E-39 | *_* | *_* |
| R263 | 82 | P | *Z. caespitosa* | 116067 | 116148 | *Z. caespitosa* | 162642 | 162723 | -1 | 4.37E-37 | *_* | *_* |
| R264 | 81 | F | *Z. caespitosa* | 1559 | 1639 | *Z. caespitosa* | 46111 | 46191 | 0 | 7.11E-39 | *_* | *_* |
| R265 | 81 | F | *Z. caespitosa* | 43507 | 43587 | *Z. caespitosa* | 46111 | 46191 | 0 | 7.11E-39 | *_* | *_* |
| R266 | 81 | F | *Z. caespitosa* | 34239 | 34319 | *Z. caespitosa* | 55114 | 55194 | 0 | 7.11E-39 | *_* | *_* |
| R267 | 81 | P | *Z. caespitosa* | 72599 | 72679 | *Z. caespitosa* | 79443 | 79523 | -2 | 2.07E-34 | *_* | *_* |
| R268 | 81 | P | *Z. caespitosa* | 53145 | 53225 | *Z. caespitosa* | 72186 | 72266 | -1 | 1.73E-36 | *_* | *_* |
| R269 | 80 | F | *Z. caespitosa* | 148737 | 148816 | *Z. caespitosa* | 148800 | 148879 | 0 | 2.84E-38 | *_* | *_* |
| R270 | 79 | F | *Z. caespitosa* | 138739 | 138817 | *Z. caespitosa* | 138882 | 138960 | 0 | 1.14E-37 | *_* | *_* |
| R271 | 79 | F | *Z. caespitosa* | 7860 | 7938 | *Z. caespitosa* | 14091 | 14169 | -3 | 2.43E-31 | *_* | *_* |
| R272 | 79 | F | *Z. caespitosa* | 7788 | 7866 | *Z. caespitosa* | 14019 | 14097 | -3 | 2.43E-31 | *_* | *_* |
| R273 | 79 | F | *Z. caespitosa* | 55947 | 56025 | *Z. caespitosa* | 73292 | 73370 | 0 | 1.14E-37 | *rrn18* | *_* |
| R274 | 79 | F | *Z. caespitosa* | 16218 | 16296 | *Z. caespitosa* | 16548 | 16626 | -1 | 2.70E-35 | *orf284a* | *orf284a* |
| R275 | 78 | F | *Z. caespitosa* | 9613 | 9690 | *Z. caespitosa* | 9677 | 9754 | -3 | 9.35E-31 | *orf397a* | *orf397a* |
| R276 | 78 | F | *Z. caespitosa* | 84233 | 84310 | *Z. caespitosa* | 135108 | 135185 | 0 | 4.55E-37 | *_* | *_* |
| R277 | 78 | P | *Z. caespitosa* | 79715 | 79792 | *Z. caespitosa* | 186382 | 186459 | 0 | 4.55E-37 | *_* | *orf100b* |
| R278 | 78 | P | *Z. caespitosa* | 84233 | 84310 | *Z. caespitosa* | 162893 | 162970 | 0 | 4.55E-37 | *_* | *_* |
| R279 | 77 | F | *Z. caespitosa* | 74594 | 74670 | *Z. caespitosa* | 101749 | 101825 | 0 | 1.82E-36 | *_* | *_* |
| R280 | 77 | F | *Z. caespitosa* | 84263 | 84339 | *Z. caespitosa* | 108806 | 108882 | 0 | 1.82E-36 | *_* | *orf161a* |
| R281 | 77 | P | *Z. caespitosa* | 77713 | 77789 | *Z. caespitosa* | 136927 | 137003 | -2 | 4.79E-32 | *_* | *_* |
| R282 | 76 | F | *Z. caespitosa* | 4223 | 4298 | *Z. caespitosa* | 177804 | 177879 | -1 | 1.66E-33 | *_* | *_* |
| R283 | 76 | F | *Z. caespitosa* | 83677 | 83752 | *Z. caespitosa* | 177804 | 177879 | -1 | 1.66E-33 | *_* | *_* |
| R284 | 76 | F | *Z. caespitosa* | 4312 | 4387 | *Z. caespitosa* | 177804 | 177879 | -1 | 1.66E-33 | *_* | *_* |
| R285 | 76 | F | *Z. caespitosa* | 177745 | 177820 | *Z. caespitosa* | 177804 | 177879 | -1 | 1.66E-33 | *_* | *_* |
| R286 | 76 | F | *Z. caespitosa* | 83766 | 83841 | *Z. caespitosa* | 177804 | 177879 | -1 | 1.66E-33 | *_* | *_* |
| R287 | 76 | F | *Z. caespitosa* | 4401 | 4476 | *Z. caespitosa* | 177804 | 177879 | -1 | 1.66E-33 | *_* | *_* |
| R288 | 76 | F | *Z. caespitosa* | 4223 | 4298 | *Z. caespitosa* | 83825 | 83900 | -1 | 1.66E-33 | *_* | *_* |
| R289 | 76 | F | *Z. caespitosa* | 83677 | 83752 | *Z. caespitosa* | 83825 | 83900 | -1 | 1.66E-33 | *_* | *_* |
| R290 | 76 | F | *Z. caespitosa* | 4312 | 4387 | *Z. caespitosa* | 83825 | 83900 | -1 | 1.66E-33 | *_* | *_* |
| R291 | 76 | F | *Z. caespitosa* | 83825 | 83900 | *Z. caespitosa* | 177745 | 177820 | -1 | 1.66E-33 | *_* | *_* |
| R292 | 76 | F | *Z. caespitosa* | 83766 | 83841 | *Z. caespitosa* | 83825 | 83900 | -1 | 1.66E-33 | *_* | *_* |
| R293 | 76 | F | *Z. caespitosa* | 4401 | 4476 | *Z. caespitosa* | 83825 | 83900 | -1 | 1.66E-33 | *_* | *_* |
| R294 | 76 | F | *Z. caespitosa* | 4223 | 4298 | *Z. caespitosa* | 4460 | 4535 | -1 | 1.66E-33 | *_* | *_* |
| R295 | 76 | F | *Z. caespitosa* | 4460 | 4535 | *Z. caespitosa* | 83677 | 83752 | -1 | 1.66E-33 | *_* | *_* |
| R296 | 76 | F | *Z. caespitosa* | 4312 | 4387 | *Z. caespitosa* | 4460 | 4535 | -1 | 1.66E-33 | *_* | *_* |
| R297 | 76 | F | *Z. caespitosa* | 4460 | 4535 | *Z. caespitosa* | 177745 | 177820 | -1 | 1.66E-33 | *_* | *_* |
| R298 | 76 | F | *Z. caespitosa* | 4460 | 4535 | *Z. caespitosa* | 83766 | 83841 | -1 | 1.66E-33 | *_* | *_* |
| R299 | 76 | F | *Z. caespitosa* | 4401 | 4476 | *Z. caespitosa* | 4460 | 4535 | -1 | 1.66E-33 | *_* | *_* |
| R300 | 76 | F | *Z. caespitosa* | 140105 | 140180 | *Z. caespitosa* | 172536 | 172611 | -1 | 1.66E-33 | *matR* | *orf185a* |
| R301 | 76 | F | *Z. caespitosa* | 46081 | 46156 | *Z. caespitosa* | 177686 | 177761 | -1 | 1.66E-33 | *_* | *_* |
| R302 | 76 | F | *Z. caespitosa* | 46081 | 46156 | *Z. caespitosa* | 180739 | 180814 | -1 | 1.66E-33 | *_* | *_* |
| R303 | 76 | F | *Z. caespitosa* | 4194 | 4269 | *Z. caespitosa* | 46081 | 46156 | -1 | 1.66E-33 | *_* | *_* |
| R304 | 76 | F | *Z. caespitosa* | 46081 | 46156 | *Z. caespitosa* | 83648 | 83723 | -1 | 1.66E-33 | *_* | *_* |
| R305 | 76 | F | *Z. caespitosa* | 4283 | 4358 | *Z. caespitosa* | 46081 | 46156 | -1 | 1.66E-33 | *_* | *_* |
| R306 | 76 | F | *Z. caespitosa* | 46081 | 46156 | *Z. caespitosa* | 177716 | 177791 | -1 | 1.66E-33 | *_* | *_* |
| R307 | 76 | F | *Z. caespitosa* | 46081 | 46156 | *Z. caespitosa* | 83737 | 83812 | -1 | 1.66E-33 | *_* | *_* |
| R308 | 76 | F | *Z. caespitosa* | 4372 | 4447 | *Z. caespitosa* | 46081 | 46156 | -1 | 1.66E-33 | *_* | *_* |
| R309 | 76 | F | *Z. caespitosa* | 1507 | 1582 | *Z. caespitosa* | 177605 | 177680 | 0 | 7.28E-36 | *_* | *_* |
| R310 | 76 | F | *Z. caespitosa* | 43455 | 43530 | *Z. caespitosa* | 177605 | 177680 | 0 | 7.28E-36 | *_* | *_* |
| R311 | 76 | F | *Z. caespitosa* | 46029 | 46104 | *Z. caespitosa* | 177605 | 177680 | 0 | 7.28E-36 | *_* | *_* |
| R312 | 76 | F | *Z. caespitosa* | 4083 | 4158 | *Z. caespitosa* | 177605 | 177680 | 0 | 7.28E-36 | *_* | *_* |
| R313 | 76 | F | *Z. caespitosa* | 37924 | 37999 | *Z. caespitosa* | 84263 | 84338 | 0 | 7.28E-36 | *_* | *_* |
| R314 | 76 | F | *Z. caespitosa* | 139831 | 139906 | *Z. caespitosa* | 139864 | 139939 | -2 | 1.87E-31 | *matR* | *matR* |
| R315 | 76 | F | *Z. caespitosa* | 24892 | 24967 | *Z. caespitosa* | 172794 | 172869 | 0 | 7.28E-36 | *_* | *orf185a* |
| R316 | 76 | F | *Z. caespitosa* | 95622 | 95697 | *Z. caespitosa* | 172794 | 172869 | 0 | 7.28E-36 | *_* | *orf185a* |
| R317 | 76 | F | *Z. caespitosa* | 24949 | 25024 | *Z. caespitosa* | 138742 | 138817 | 0 | 7.28E-36 | *_* | *_* |
| R318 | 76 | F | *Z. caespitosa* | 95679 | 95754 | *Z. caespitosa* | 138742 | 138817 | 0 | 7.28E-36 | *_* | *_* |
| R319 | 76 | P | *Z. caespitosa* | 140105 | 140180 | *Z. caespitosa* | 184178 | 184253 | -1 | 1.66E-33 | *matR* | *orf130b* |
| R320 | 76 | P | *Z. caespitosa* | 140105 | 140180 | *Z. caespitosa* | 181616 | 181691 | -1 | 1.66E-33 | *matR* | *orf103d* |
| R321 | 76 | P | *Z. caespitosa* | 116058 | 116133 | *Z. caespitosa* | 172794 | 172869 | 0 | 7.28E-36 | *_* | *orf185a* |
| R322 | 76 | P | *Z. caespitosa* | 75886 | 75961 | *Z. caespitosa* | 140105 | 140180 | -1 | 1.66E-33 | *_* | *matR* |
| R323 | 76 | P | *Z. caespitosa* | 116001 | 116076 | *Z. caespitosa* | 138742 | 138817 | 0 | 7.28E-36 | *_* | *_* |
| R324 | 76 | P | *Z. caespitosa* | 37924 | 37999 | *Z. caespitosa* | 72183 | 72258 | -1 | 1.66E-33 | *_* | *_* |
| R325 | 75 | F | *Z. caespitosa* | 153261 | 153335 | *Z. caespitosa* | 160726 | 160800 | -2 | 7.28E-31 | *_* | *atp9* |
| R326 | 75 | F | *Z. caespitosa* | 4135 | 4209 | *Z. caespitosa* | 177805 | 177879 | -1 | 6.55E-33 | *_* | *_* |
| R327 | 75 | F | *Z. caespitosa* | 83589 | 83663 | *Z. caespitosa* | 177805 | 177879 | -1 | 6.55E-33 | *_* | *_* |
| R328 | 75 | F | *Z. caespitosa* | 4135 | 4209 | *Z. caespitosa* | 83826 | 83900 | -1 | 6.55E-33 | *_* | *_* |
| R329 | 75 | F | *Z. caespitosa* | 83589 | 83663 | *Z. caespitosa* | 83826 | 83900 | -1 | 6.55E-33 | *_* | *_* |
| R330 | 75 | F | *Z. caespitosa* | 4135 | 4209 | *Z. caespitosa* | 4461 | 4535 | -1 | 6.55E-33 | *_* | *_* |
| R331 | 75 | F | *Z. caespitosa* | 4461 | 4535 | *Z. caespitosa* | 83589 | 83663 | -1 | 6.55E-33 | *_* | *_* |
| R332 | 75 | F | *Z. caespitosa* | 4135 | 4209 | *Z. caespitosa* | 180769 | 180843 | -1 | 6.55E-33 | *_* | *_* |
| R333 | 75 | F | *Z. caespitosa* | 83589 | 83663 | *Z. caespitosa* | 180769 | 180843 | -1 | 6.55E-33 | *_* | *_* |
| R334 | 75 | F | *Z. caespitosa* | 16143 | 16217 | *Z. caespitosa* | 69915 | 69989 | 0 | 2.91E-35 | *orf284a* | *_* |
| R335 | 75 | F | *Z. caespitosa* | 72184 | 72258 | *Z. caespitosa* | 188069 | 188143 | -1 | 6.55E-33 | *_* | *_* |
| R336 | 75 | F | *Z. caespitosa* | 79452 | 79526 | *Z. caespitosa* | 140107 | 140181 | -1 | 6.55E-33 | *_* | *matR* |
| R337 | 75 | P | *Z. caespitosa* | 84263 | 84337 | *Z. caespitosa* | 188069 | 188143 | 0 | 2.91E-35 | *_* | *_* |
| R338 | 75 | P | *Z. caespitosa* | 123128 | 123202 | *Z. caespitosa* | 149095 | 149169 | -1 | 6.55E-33 | *orf103c* | *_* |
| R339 | 74 | F | *Z. caespitosa* | 9583 | 9656 | *Z. caespitosa* | 9647 | 9720 | -3 | 2.04E-28 | *orf397a* | *orf397a* |
| R340 | 74 | F | *Z. caespitosa* | 77794 | 77867 | *Z. caespitosa* | 140107 | 140180 | -1 | 2.59E-32 | *_* | *matR* |
| R341 | 74 | F | *Z. caespitosa* | 79452 | 79525 | *Z. caespitosa* | 172538 | 172611 | -2 | 2.83E-30 | *_* | *orf185a* |
| R342 | 74 | F | *Z. caespitosa* | 9109 | 9182 | *Z. caespitosa* | 9812 | 9885 | -3 | 2.04E-28 | *orf397a* | *orf397a* |
| R343 | 74 | P | *Z. caespitosa* | 79452 | 79525 | *Z. caespitosa* | 184178 | 184251 | -2 | 2.83E-30 | *_* | *orf130b* |
| R344 | 74 | P | *Z. caespitosa* | 79452 | 79525 | *Z. caespitosa* | 181616 | 181689 | -2 | 2.83E-30 | *_* | *orf103d* |
| R345 | 74 | P | *Z. caespitosa* | 140107 | 140180 | *Z. caespitosa* | 177951 | 178024 | -1 | 2.59E-32 | *matR* | *_* |
| R346 | 74 | P | *Z. caespitosa* | 136911 | 136984 | *Z. caespitosa* | 140107 | 140180 | -1 | 2.59E-32 | *_* | *matR* |
| R347 | 74 | P | *Z. caespitosa* | 83972 | 84045 | *Z. caespitosa* | 140107 | 140180 | -1 | 2.59E-32 | *_* | *matR* |
| R348 | 74 | P | *Z. caespitosa* | 75886 | 75959 | *Z. caespitosa* | 79452 | 79525 | -2 | 2.83E-30 | *_* | *_* |
| R349 | 73 | F | *Z. caespitosa* | 101626 | 101698 | *Z. caespitosa* | 172780 | 172852 | -3 | 7.83E-28 | *_* | *orf185a* |
| R350 | 73 | F | *Z. caespitosa* | 101626 | 101698 | *Z. caespitosa* | 138638 | 138710 | -3 | 7.83E-28 | *_* | *_* |
| R351 | 73 | F | *Z. caespitosa* | 72186 | 72258 | *Z. caespitosa* | 179897 | 179969 | -1 | 1.02E-31 | *_* | *_* |
| R352 | 73 | F | *Z. caespitosa* | 155657 | 155729 | *Z. caespitosa* | 155753 | 155825 | 0 | 4.66E-34 | *_* | *_* |
| R353 | 73 | F | *Z. caespitosa* | 52670 | 52742 | *Z. caespitosa* | 84263 | 84335 | 0 | 4.66E-34 | *orf172a* | *_* |
| R354 | 73 | F | *Z. caespitosa* | 52939 | 53011 | *Z. caespitosa* | 84263 | 84335 | 0 | 4.66E-34 | *orf172a* | *_* |
| R355 | 73 | F | *Z. caespitosa* | 37924 | 37996 | *Z. caespitosa* | 53153 | 53225 | 0 | 4.66E-34 | *_* | *_* |
| R356 | 73 | F | *Z. caespitosa* | 53153 | 53225 | *Z. caespitosa* | 108806 | 108878 | 0 | 4.66E-34 | *_* | *orf161a* |
| R357 | 73 | F | *Z. caespitosa* | 52670 | 52742 | *Z. caespitosa* | 53153 | 53225 | 0 | 4.66E-34 | *orf172a* | *_* |
| R358 | 73 | F | *Z. caespitosa* | 52939 | 53011 | *Z. caespitosa* | 53153 | 53225 | 0 | 4.66E-34 | *orf172a* | *_* |
| R359 | 73 | F | *Z. caespitosa* | 68348 | 68420 | *Z. caespitosa* | 172794 | 172866 | 0 | 4.66E-34 | *_* | *orf185a* |
| R360 | 73 | F | *Z. caespitosa* | 24892 | 24964 | *Z. caespitosa* | 68348 | 68420 | 0 | 4.66E-34 | *_* | *_* |
| R361 | 73 | F | *Z. caespitosa* | 68348 | 68420 | *Z. caespitosa* | 95622 | 95694 | 0 | 4.66E-34 | *_* | *_* |
| R362 | 73 | F | *Z. caespitosa* | 15688 | 15760 | *Z. caespitosa* | 15725 | 15797 | -3 | 7.83E-28 | *_* | *_* |
| R363 | 73 | P | *Z. caespitosa* | 53153 | 53225 | *Z. caespitosa* | 188071 | 188143 | 0 | 4.66E-34 | *_* | *_* |
| R364 | 73 | P | *Z. caespitosa* | 84263 | 84335 | *Z. caespitosa* | 179897 | 179969 | 0 | 4.66E-34 | *_* | *_* |
| R365 | 73 | P | *Z. caespitosa* | 53153 | 53225 | *Z. caespitosa* | 179897 | 179969 | 0 | 4.66E-34 | *_* | *_* |
| R366 | 73 | P | *Z. caespitosa* | 27032 | 27104 | *Z. caespitosa* | 136779 | 136851 | -1 | 1.02E-31 | *_* | *_* |
| R367 | 73 | P | *Z. caespitosa* | 68348 | 68420 | *Z. caespitosa* | 116061 | 116133 | 0 | 4.66E-34 | *_* | *_* |
| R368 | 73 | P | *Z. caespitosa* | 37718 | 37790 | *Z. caespitosa* | 87373 | 87445 | -1 | 1.02E-31 | *_* | *orf122a* |
| R369 | 73 | P | *Z. caespitosa* | 52670 | 52742 | *Z. caespitosa* | 72186 | 72258 | -1 | 1.02E-31 | *orf172a* | *_* |
| R370 | 73 | P | *Z. caespitosa* | 52939 | 53011 | *Z. caespitosa* | 72186 | 72258 | -1 | 1.02E-31 | *orf172a* | *_* |
| R371 | 72 | F | *Z. caespitosa* | 1511 | 1582 | *Z. caespitosa* | 180662 | 180733 | 0 | 1.86E-33 | *_* | *_* |
| R372 | 72 | F | *Z. caespitosa* | 43459 | 43530 | *Z. caespitosa* | 180662 | 180733 | 0 | 1.86E-33 | *_* | *_* |
| R373 | 72 | F | *Z. caespitosa* | 46033 | 46104 | *Z. caespitosa* | 180662 | 180733 | 0 | 1.86E-33 | *_* | *_* |
| R374 | 72 | F | *Z. caespitosa* | 4087 | 4158 | *Z. caespitosa* | 180662 | 180733 | 0 | 1.86E-33 | *_* | *_* |
| R375 | 72 | F | *Z. caespitosa* | 83541 | 83612 | *Z. caespitosa* | 180662 | 180733 | 0 | 1.86E-33 | *_* | *_* |
| R376 | 72 | F | *Z. caespitosa* | 72599 | 72670 | *Z. caespitosa* | 181618 | 181689 | 0 | 1.86E-33 | *_* | *orf103d* |
| R377 | 72 | F | *Z. caespitosa* | 72599 | 72670 | *Z. caespitosa* | 75888 | 75959 | 0 | 1.86E-33 | *_* | *_* |
| R378 | 72 | F | *Z. caespitosa* | 72599 | 72670 | *Z. caespitosa* | 184180 | 184251 | 0 | 1.86E-33 | *_* | *orf130b* |
| R379 | 72 | F | *Z. caespitosa* | 9852 | 9923 | *Z. caespitosa* | 9915 | 9986 | -3 | 3.00E-27 | *orf397a* | *orf397a* |
| R380 | 72 | P | *Z. caespitosa* | 180662 | 180733 | *Z. caespitosa* | 188190 | 188261 | 0 | 1.86E-33 | *_* | *_* |
| R381 | 72 | P | *Z. caespitosa* | 172966 | 173037 | *Z. caespitosa* | 180546 | 180617 | -3 | 3.00E-27 | *orf185a* | *_* |
| R382 | 72 | P | *Z. caespitosa* | 149165 | 149236 | *Z. caespitosa* | 175069 | 175140 | -2 | 4.29E-29 | *_* | *_* |
| R383 | 72 | P | *Z. caespitosa* | 72599 | 72670 | *Z. caespitosa* | 172538 | 172609 | 0 | 1.86E-33 | *_* | *orf185a* |
| R384 | 72 | P | *Z. caespitosa* | 72599 | 72670 | *Z. caespitosa* | 140107 | 140178 | -1 | 4.03E-31 | *_* | *matR* |
| R385 | 72 | P | *Z. caespitosa* | 79619 | 79690 | *Z. caespitosa* | 135046 | 135117 | -1 | 4.03E-31 | *_* | *_* |
| R386 | 71 | F | *Z. caespitosa* | 48647 | 48717 | *Z. caespitosa* | 172949 | 173019 | 0 | 7.46E-33 | *_* | *orf185a* |
| R387 | 71 | F | *Z. caespitosa* | 9647 | 9717 | *Z. caespitosa* | 9775 | 9845 | -3 | 1.15E-26 | *orf397a* | *orf397a* |
| R388 | 71 | F | *Z. caespitosa* | 57647 | 57717 | *Z. caespitosa* | 116058 | 116128 | 0 | 7.46E-33 | *orf117a* | *_* |
| R389 | 71 | P | *Z. caespitosa* | 57562 | 57632 | *Z. caespitosa* | 162776 | 162846 | 0 | 7.46E-33 | *orf117a* | *_* |
| R390 | 71 | P | *Z. caespitosa* | 52779 | 52849 | *Z. caespitosa* | 162776 | 162846 | 0 | 7.46E-33 | *orf172a* | *_* |
| R391 | 71 | P | *Z. caespitosa* | 57647 | 57717 | *Z. caespitosa* | 95627 | 95697 | 0 | 7.46E-33 | *orf117a* | *_* |
| R392 | 71 | P | *Z. caespitosa* | 24897 | 24967 | *Z. caespitosa* | 57647 | 57717 | 0 | 7.46E-33 | *_* | *orf117a* |
| R393 | 71 | P | *Z. caespitosa* | 34983 | 35053 | *Z. caespitosa* | 36790 | 36860 | 0 | 7.46E-33 | *_* | *orf134a;nad4* |
| R394 | 70 | F | *Z. caespitosa* | 22058 | 22127 | *Z. caespitosa* | 180548 | 180617 | -1 | 6.26E-30 | *_* | *_* |
| R395 | 70 | F | *Z. caespitosa* | 33021 | 33090 | *Z. caespitosa* | 53051 | 53120 | 0 | 2.98E-32 | *_* | *_* |
| R396 | 70 | F | *Z. caespitosa* | 118898 | 118967 | *Z. caespitosa* | 172966 | 173035 | -2 | 6.48E-28 | *_* | *orf185a* |
| R397 | 70 | F | *Z. caespitosa* | 75664 | 75733 | *Z. caespitosa* | 116058 | 116127 | -3 | 4.41E-26 | *_* | *_* |
| R398 | 70 | F | *Z. caespitosa* | 14811 | 14880 | *Z. caespitosa* | 25236 | 25305 | -1 | 6.26E-30 | *orf295a;cox2* | *orf340a* |
| R399 | 70 | F | *Z. caespitosa* | 14811 | 14880 | *Z. caespitosa* | 95966 | 96035 | -1 | 6.26E-30 | *orf295a;cox2* | *orf540a-2* |
| R400 | 70 | P | *Z. caespitosa* | 118898 | 118967 | *Z. caespitosa* | 180548 | 180617 | -1 | 6.26E-30 | *_* | *_* |
| R401 | 70 | P | *Z. caespitosa* | 77720 | 77789 | *Z. caespitosa* | 177967 | 178036 | 0 | 2.98E-32 | *_* | *_* |
| R402 | 70 | P | *Z. caespitosa* | 22058 | 22127 | *Z. caespitosa* | 172966 | 173035 | -2 | 6.48E-28 | *_* | *orf185a* |
| R403 | 70 | P | *Z. caespitosa* | 14811 | 14880 | *Z. caespitosa* | 115720 | 115789 | -1 | 6.26E-30 | *orf295a;cox2* | *orf540a-1* |
| R404 | 70 | P | *Z. caespitosa* | 75664 | 75733 | *Z. caespitosa* | 95628 | 95697 | -3 | 4.41E-26 | *_* | *_* |
| R405 | 70 | P | *Z. caespitosa* | 72613 | 72682 | *Z. caespitosa* | 77720 | 77789 | 0 | 2.98E-32 | *_* | *_* |
| R406 | 70 | P | *Z. caespitosa* | 24898 | 24967 | *Z. caespitosa* | 75664 | 75733 | -3 | 4.41E-26 | *_* | *_* |
| R407 | 69 | F | *Z. caespitosa* | 101709 | 101777 | *Z. caespitosa* | 179922 | 179990 | 0 | 1.19E-31 | *_* | *_* |
| R408 | 69 | P | *Z. caespitosa* | 74665 | 74733 | *Z. caespitosa* | 181634 | 181702 | -3 | 1.69E-25 | *_* | *orf103d* |
| R409 | 69 | P | *Z. caespitosa* | 53232 | 53300 | *Z. caespitosa* | 169755 | 169823 | 0 | 1.19E-31 | *_* | *orf509a;atp1* |
| R410 | 69 | P | *Z. caespitosa* | 52582 | 52650 | *Z. caespitosa* | 79709 | 79777 | -1 | 2.47E-29 | *orf172a* | *_* |
| R411 | 68 | F | *Z. caespitosa* | 16606 | 16673 | *Z. caespitosa* | 74721 | 74788 | -3 | 6.46E-25 | *orf284a* | *_* |
| R412 | 68 | F | *Z. caespitosa* | 9869 | 9936 | *Z. caespitosa* | 10121 | 10188 | -3 | 6.46E-25 | *orf397a* | *orf397a* |
| R413 | 68 | F | *Z. caespitosa* | 9869 | 9936 | *Z. caespitosa* | 9995 | 10062 | -3 | 6.46E-25 | *orf397a* | *orf397a* |
| R414 | 68 | F | *Z. caespitosa* | 69915 | 69982 | *Z. caespitosa* | 162776 | 162843 | 0 | 4.77E-31 | *_* | *_* |
| R415 | 68 | F | *Z. caespitosa* | 68348 | 68415 | *Z. caespitosa* | 138652 | 138719 | -1 | 9.74E-29 | *_* | *_* |
| R416 | 68 | F | *Z. caespitosa* | 24892 | 24959 | *Z. caespitosa* | 138652 | 138719 | -1 | 9.74E-29 | *_* | *_* |
| R417 | 68 | F | *Z. caespitosa* | 95622 | 95689 | *Z. caespitosa* | 138652 | 138719 | -1 | 9.74E-29 | *_* | *_* |
| R418 | 68 | F | *Z. caespitosa* | 74666 | 74733 | *Z. caespitosa* | 172526 | 172593 | -3 | 6.46E-25 | *_* | *_* |
| R419 | 68 | F | *Z. caespitosa* | 37812 | 37879 | *Z. caespitosa* | 148822 | 148889 | -1 | 9.74E-29 | *_* | *_* |
| R420 | 68 | P | *Z. caespitosa* | 74666 | 74733 | *Z. caespitosa* | 184196 | 184263 | -3 | 6.46E-25 | *_* | *orf130b* |
| R421 | 68 | P | *Z. caespitosa* | 116066 | 116133 | *Z. caespitosa* | 138652 | 138719 | -1 | 9.74E-29 | *_* | *_* |
| R422 | 68 | P | *Z. caespitosa* | 77722 | 77789 | *Z. caespitosa* | 83988 | 84055 | 0 | 4.77E-31 | *_* | *_* |
| R423 | 68 | P | *Z. caespitosa* | 74666 | 74733 | *Z. caespitosa* | 75904 | 75971 | -3 | 6.46E-25 | *_* | *_* |
| R424 | 68 | P | *Z. caespitosa* | 57650 | 57717 | *Z. caespitosa* | 68353 | 68420 | 0 | 4.77E-31 | *orf117a* | *_* |
| R425 | 68 | P | *Z. caespitosa* | 16143 | 16210 | *Z. caespitosa* | 57565 | 57632 | 0 | 4.77E-31 | *orf284a* | *orf117a* |
| R426 | 68 | P | *Z. caespitosa* | 16143 | 16210 | *Z. caespitosa* | 52782 | 52849 | 0 | 4.77E-31 | *orf284a* | *orf172a* |
| R427 | 67 | F | *Z. caespitosa* | 68348 | 68414 | *Z. caespitosa* | 162657 | 162723 | -1 | 3.84E-28 | *_* | *_* |
| R428 | 67 | F | *Z. caespitosa* | 162657 | 162723 | *Z. caespitosa* | 172794 | 172860 | -1 | 3.84E-28 | *_* | *orf185a* |
| R429 | 67 | F | *Z. caespitosa* | 138652 | 138718 | *Z. caespitosa* | 162657 | 162723 | -2 | 3.80E-26 | *_* | *_* |
| R430 | 67 | F | *Z. caespitosa* | 74667 | 74733 | *Z. caespitosa* | 77659 | 77725 | -1 | 3.84E-28 | *_* | *_* |
| R431 | 67 | F | *Z. caespitosa* | 77723 | 77789 | *Z. caespitosa* | 79443 | 79509 | -1 | 3.84E-28 | *_* | *_* |
| R432 | 67 | P | *Z. caespitosa* | 87366 | 87432 | *Z. caespitosa* | 156567 | 156633 | -3 | 2.47E-24 | *orf122a* | *_* |
| R433 | 67 | P | *Z. caespitosa* | 72613 | 72679 | *Z. caespitosa* | 79443 | 79509 | -1 | 3.84E-28 | *_* | *_* |
| R434 | 67 | P | *Z. caespitosa* | 68354 | 68420 | *Z. caespitosa* | 75667 | 75733 | -3 | 2.47E-24 | *_* | *_* |
| R435 | 67 | P | *Z. caespitosa* | 29893 | 29959 | *Z. caespitosa* | 40254 | 40320 | -1 | 3.84E-28 | *_* | *orf309a* |
| R436 | 66 | F | *Z. caespitosa* | 51038 | 51103 | *Z. caespitosa* | 109978 | 110043 | -3 | 9.44E-24 | *ccmFn* | *orf165a* |
| R437 | 66 | F | *Z. caespitosa* | 9143 | 9208 | *Z. caespitosa* | 9591 | 9656 | -3 | 9.44E-24 | *orf397a* | *orf397a* |
| R438 | 66 | F | *Z. caespitosa* | 74676 | 74741 | *Z. caespitosa* | 140105 | 140170 | -2 | 1.47E-25 | *_* | *matR* |
| R439 | 65 | F | *Z. caespitosa* | 9891 | 9955 | *Z. caespitosa* | 10143 | 10207 | -3 | 3.60E-23 | *orf397a* | *orf397a* |
| R440 | 65 | F | *Z. caespitosa* | 67532 | 67596 | *Z. caespitosa* | 160233 | 160297 | 0 | 3.05E-29 | *orf130a* | *_* |
| R441 | 65 | F | *Z. caespitosa* | 181566 | 181630 | *Z. caespitosa* | 186462 | 186526 | 0 | 3.05E-29 | *orf103d* | *orf100b* |
| R442 | 65 | F | *Z. caespitosa* | 136861 | 136925 | *Z. caespitosa* | 186462 | 186526 | 0 | 3.05E-29 | *_* | *orf100b* |
| R443 | 65 | F | *Z. caespitosa* | 177901 | 177965 | *Z. caespitosa* | 186462 | 186526 | 0 | 3.05E-29 | *_* | *orf100b* |
| R444 | 65 | F | *Z. caespitosa* | 83922 | 83986 | *Z. caespitosa* | 186462 | 186526 | 0 | 3.05E-29 | *_* | *orf100b* |
| R445 | 65 | P | *Z. caespitosa* | 55115 | 55179 | *Z. caespitosa* | 172926 | 172990 | 0 | 3.05E-29 | *_* | *orf185a* |
| R446 | 65 | P | *Z. caespitosa* | 34240 | 34304 | *Z. caespitosa* | 172926 | 172990 | 0 | 3.05E-29 | *_* | *orf185a* |
| R447 | 65 | P | *Z. caespitosa* | 53016 | 53080 | *Z. caespitosa* | 172851 | 172915 | 0 | 3.05E-29 | *_* | *orf185a* |
| R448 | 65 | P | *Z. caespitosa* | 49992 | 50056 | *Z. caespitosa* | 74746 | 74810 | -1 | 5.96E-27 | *_* | *_* |
| R449 | 65 | P | *Z. caespitosa* | 52882 | 52946 | *Z. caespitosa* | 74594 | 74658 | 0 | 3.05E-29 | *orf172a* | *_* |
| R450 | 65 | P | *Z. caespitosa* | 15838 | 15902 | *Z. caespitosa* | 43536 | 43600 | -3 | 3.60E-23 | *_* | *_* |
| R451 | 65 | P | *Z. caespitosa* | 1588 | 1652 | *Z. caespitosa* | 15838 | 15902 | -3 | 3.60E-23 | *_* | *_* |
| R452 | 64 | P | *Z. caespitosa* | 101600 | 101663 | *Z. caespitosa* | 184102 | 184165 | 0 | 1.22E-28 | *_* | *orf130b* |
| R453 | 64 | P | *Z. caespitosa* | 79650 | 79713 | *Z. caespitosa* | 108747 | 108810 | -1 | 2.35E-26 | *_* | *orf161a* |
| R454 | 64 | P | *Z. caespitosa* | 6565 | 6628 | *Z. caespitosa* | 59792 | 59855 | 0 | 1.22E-28 | *_* | *orf426a* |
| R455 | 63 | F | *Z. caespitosa* | 37720 | 37782 | *Z. caespitosa* | 156556 | 156618 | -2 | 8.59E-24 | *_* | *_* |
| R456 | 63 | F | *Z. caespitosa* | 79729 | 79791 | *Z. caespitosa* | 135916 | 135978 | 0 | 4.89E-28 | *_* | *_* |
| R457 | 63 | F | *Z. caespitosa* | 9143 | 9205 | *Z. caespitosa* | 9783 | 9845 | -3 | 5.24E-22 | *orf397a* | *orf397a* |
| R458 | 63 | F | *Z. caespitosa* | 72196 | 72258 | *Z. caespitosa* | 188081 | 188143 | 0 | 4.89E-28 | *_* | *_* |
| R459 | 63 | F | *Z. caespitosa* | 72196 | 72258 | *Z. caespitosa* | 179907 | 179969 | 0 | 4.89E-28 | *_* | *_* |
| R460 | 63 | F | *Z. caespitosa* | 9628 | 9690 | *Z. caespitosa* | 9756 | 9818 | -3 | 5.24E-22 | *orf397a* | *orf397a* |
| R461 | 63 | F | *Z. caespitosa* | 52582 | 52644 | *Z. caespitosa* | 186397 | 186459 | -1 | 9.24E-26 | *orf172a* | *orf100b* |
| R462 | 63 | P | *Z. caespitosa* | 135916 | 135978 | *Z. caespitosa* | 186383 | 186445 | 0 | 4.89E-28 | *_* | *orf100b* |
| R463 | 63 | P | *Z. caespitosa* | 87381 | 87443 | *Z. caespitosa* | 156556 | 156618 | -1 | 9.24E-26 | *orf122a* | *_* |
| R464 | 63 | P | *Z. caespitosa* | 57655 | 57717 | *Z. caespitosa* | 138657 | 138719 | -1 | 9.24E-26 | *orf117a* | *_* |
| R465 | 63 | P | *Z. caespitosa* | 72196 | 72258 | *Z. caespitosa* | 108806 | 108868 | 0 | 4.89E-28 | *_* | *orf161a* |
| R466 | 62 | F | *Z. caespitosa* | 9886 | 9947 | *Z. caespitosa* | 10012 | 10073 | -3 | 2.00E-21 | *orf397a* | *orf397a* |
| R467 | 62 | F | *Z. caespitosa* | 77666 | 77727 | *Z. caespitosa* | 172534 | 172595 | 0 | 1.96E-27 | *_* | *_* |
| R468 | 62 | F | *Z. caespitosa* | 74680 | 74741 | *Z. caespitosa* | 77796 | 77857 | -2 | 3.33E-23 | *_* | *_* |
| R469 | 62 | F | *Z. caespitosa* | 74680 | 74741 | *Z. caespitosa* | 79454 | 79515 | -2 | 3.33E-23 | *_* | *_* |
| R470 | 62 | P | *Z. caespitosa* | 77666 | 77727 | *Z. caespitosa* | 184194 | 184255 | 0 | 1.96E-27 | *_* | *orf130b* |
| R471 | 62 | P | *Z. caespitosa* | 77666 | 77727 | *Z. caespitosa* | 181632 | 181693 | 0 | 1.96E-27 | *_* | *orf103d* |
| R472 | 62 | P | *Z. caespitosa* | 74680 | 74741 | *Z. caespitosa* | 177961 | 178022 | -2 | 3.33E-23 | *_* | *_* |
| R473 | 62 | P | *Z. caespitosa* | 57656 | 57717 | *Z. caespitosa* | 162662 | 162723 | -1 | 3.64E-25 | *orf117a* | *_* |
| R474 | 62 | P | *Z. caespitosa* | 74680 | 74741 | *Z. caespitosa* | 136921 | 136982 | -2 | 3.33E-23 | *_* | *_* |
| R475 | 62 | P | *Z. caespitosa* | 74680 | 74741 | *Z. caespitosa* | 83982 | 84043 | -2 | 3.33E-23 | *_* | *_* |
| R476 | 62 | P | *Z. caespitosa* | 75902 | 75963 | *Z. caespitosa* | 77666 | 77727 | 0 | 1.96E-27 | *_* | *_* |
| R477 | 62 | P | *Z. caespitosa* | 72607 | 72668 | *Z. caespitosa* | 74680 | 74741 | -2 | 3.33E-23 | *_* | *_* |
| R478 | 61 | F | *Z. caespitosa* | 8083 | 8143 | *Z. caespitosa* | 14315 | 14375 | -3 | 7.60E-21 | *_* | *orf113a* |
| R479 | 61 | F | *Z. caespitosa* | 101696 | 101756 | *Z. caespitosa* | 162880 | 162940 | 0 | 7.82E-27 | *_* | *_* |
| R480 | 61 | F | *Z. caespitosa* | 108736 | 108796 | *Z. caespitosa* | 148800 | 148860 | -2 | 1.29E-22 | *orf161a* | *_* |
| R481 | 61 | F | *Z. caespitosa* | 163051 | 163111 | *Z. caespitosa* | 172685 | 172745 | 0 | 7.82E-27 | *_* | *orf185a* |
| R482 | 61 | F | *Z. caespitosa* | 9938 | 9998 | *Z. caespitosa* | 10190 | 10250 | -2 | 1.29E-22 | *orf397a* | *orf397a* |
| R483 | 61 | P | *Z. caespitosa* | 75752 | 75812 | *Z. caespitosa* | 163051 | 163111 | 0 | 7.82E-27 | *_* | *_* |
| R484 | 61 | P | *Z. caespitosa* | 148800 | 148860 | *Z. caespitosa* | 163006 | 163066 | 0 | 7.82E-27 | *_* | *_* |
| R485 | 61 | P | *Z. caespitosa* | 1444 | 1504 | *Z. caespitosa* | 162557 | 162617 | 0 | 7.82E-27 | *_* | *_* |
| R486 | 61 | P | *Z. caespitosa* | 43392 | 43452 | *Z. caespitosa* | 162557 | 162617 | 0 | 7.82E-27 | *_* | *_* |
| R487 | 61 | P | *Z. caespitosa* | 101696 | 101756 | *Z. caespitosa* | 135138 | 135198 | 0 | 7.82E-27 | *_* | *_* |
| R488 | 61 | P | *Z. caespitosa* | 101709 | 101769 | *Z. caespitosa* | 108793 | 108853 | 0 | 7.82E-27 | *_* | *orf161a* |
| R489 | 61 | P | *Z. caespitosa* | 37911 | 37971 | *Z. caespitosa* | 101709 | 101769 | 0 | 7.82E-27 | *_* | *_* |
| R490 | 60 | F | *Z. caespitosa* | 77668 | 77727 | *Z. caespitosa* | 140105 | 140164 | 0 | 3.13E-26 | *_* | *matR* |
| R491 | 60 | F | *Z. caespitosa* | 9658 | 9717 | *Z. caespitosa* | 9786 | 9845 | -2 | 4.98E-22 | *orf397a* | *orf397a* |
| R492 | 60 | F | *Z. caespitosa* | 101709 | 101768 | *Z. caespitosa* | 188096 | 188155 | 0 | 3.13E-26 | *_* | *_* |
| R493 | 60 | F | *Z. caespitosa* | 75975 | 76034 | *Z. caespitosa* | 100033 | 100092 | -2 | 4.98E-22 | *_* | *_* |
| R494 | 60 | F | *Z. caespitosa* | 53096 | 53155 | *Z. caespitosa* | 186453 | 186512 | -3 | 2.89E-20 | *_* | *orf100b* |
| R495 | 60 | F | *Z. caespitosa* | 16186 | 16245 | *Z. caespitosa* | 74634 | 74693 | -1 | 5.63E-24 | *orf284a* | *_* |
| R496 | 60 | F | *Z. caespitosa* | 116036 | 116095 | *Z. caespitosa* | 180606 | 180665 | 0 | 3.13E-26 | *_* | *_* |
| R497 | 60 | F | *Z. caespitosa* | 57027 | 57086 | *Z. caespitosa* | 74138 | 74197 | -3 | 2.89E-20 | *rrn18;orf152a* | *_* |
| R498 | 60 | P | *Z. caespitosa* | 95660 | 95719 | *Z. caespitosa* | 180606 | 180665 | 0 | 3.13E-26 | *_* | *_* |
| R499 | 60 | P | *Z. caespitosa* | 24930 | 24989 | *Z. caespitosa* | 180606 | 180665 | 0 | 3.13E-26 | *_* | *_* |
| R500 | 60 | P | *Z. caespitosa* | 75902 | 75961 | *Z. caespitosa* | 140105 | 140164 | 0 | 3.13E-26 | *_* | *matR* |
| R501 | 60 | P | *Z. caespitosa* | 75975 | 76034 | *Z. caespitosa* | 111664 | 111723 | -2 | 4.98E-22 | *_* | *_* |
| R502 | 60 | P | *Z. caespitosa* | 52658 | 52717 | *Z. caespitosa* | 101709 | 101768 | 0 | 3.13E-26 | *orf172a* | *_* |
| R503 | 59 | F | *Z. caespitosa* | 41418 | 41476 | *Z. caespitosa* | 68890 | 68948 | -3 | 1.10E-19 | *orf120b* | *_* |
| R504 | 59 | F | *Z. caespitosa* | 68348 | 68406 | *Z. caespitosa* | 101640 | 101698 | -3 | 1.10E-19 | *_* | *_* |
| R505 | 59 | F | *Z. caespitosa* | 101640 | 101698 | *Z. caespitosa* | 162657 | 162715 | -3 | 1.10E-19 | *_* | *_* |
| R506 | 59 | F | *Z. caespitosa* | 24892 | 24950 | *Z. caespitosa* | 101640 | 101698 | -3 | 1.10E-19 | *_* | *_* |
| R507 | 59 | F | *Z. caespitosa* | 95622 | 95680 | *Z. caespitosa* | 101640 | 101698 | -3 | 1.10E-19 | *_* | *_* |
| R508 | 59 | F | *Z. caespitosa* | 69709 | 69767 | *Z. caespitosa* | 127513 | 127571 | -2 | 1.93E-21 | *_* | *orf149b* |
| R509 | 59 | F | *Z. caespitosa* | 9894 | 9952 | *Z. caespitosa* | 10083 | 10141 | -3 | 1.10E-19 | *orf397a* | *orf397a* |
| R510 | 59 | F | *Z. caespitosa* | 123180 | 123238 | *Z. caespitosa* | 157551 | 157609 | -3 | 1.10E-19 | *_* | *rrn26* |
| R511 | 59 | F | *Z. caespitosa* | 83353 | 83411 | *Z. caespitosa* | 118804 | 118862 | -2 | 1.93E-21 | *_* | *_* |
| R512 | 59 | P | *Z. caespitosa* | 101640 | 101698 | *Z. caespitosa* | 116075 | 116133 | -3 | 1.10E-19 | *_* | *_* |
| R513 | 59 | P | *Z. caespitosa* | 22163 | 22221 | *Z. caespitosa* | 83353 | 83411 | -2 | 1.93E-21 | *_* | *_* |
| R514 | 59 | P | *Z. caespitosa* | 52592 | 52650 | *Z. caespitosa* | 79709 | 79767 | 0 | 1.25E-25 | *orf172a* | *_* |
| R515 | 58 | F | *Z. caespitosa* | 155791 | 155848 | *Z. caespitosa* | 184273 | 184330 | 0 | 5.00E-25 | *_* | *_* |
| R516 | 58 | F | *Z. caespitosa* | 9866 | 9923 | *Z. caespitosa* | 10181 | 10238 | -3 | 4.17E-19 | *orf397a* | *orf397a* |
| R517 | 58 | F | *Z. caespitosa* | 8093 | 8150 | *Z. caespitosa* | 14325 | 14382 | -3 | 4.17E-19 | *_* | *orf113a* |
| R518 | 58 | F | *Z. caespitosa* | 77670 | 77727 | *Z. caespitosa* | 77794 | 77851 | 0 | 5.00E-25 | *_* | *_* |
| R519 | 58 | F | *Z. caespitosa* | 77732 | 77789 | *Z. caespitosa* | 172538 | 172595 | 0 | 5.00E-25 | *_* | *_* |
| R520 | 58 | F | *Z. caespitosa* | 77732 | 77789 | *Z. caespitosa* | 140107 | 140164 | 0 | 5.00E-25 | *_* | *matR* |
| R521 | 58 | F | *Z. caespitosa* | 77670 | 77727 | *Z. caespitosa* | 79452 | 79509 | -1 | 8.71E-23 | *_* | *_* |
| R522 | 58 | F | *Z. caespitosa* | 15703 | 15760 | *Z. caespitosa* | 15740 | 15797 | -2 | 7.45E-21 | *_* | *_* |
| R523 | 58 | F | *Z. caespitosa* | 37812 | 37869 | *Z. caespitosa* | 148759 | 148816 | -1 | 8.71E-23 | *_* | *_* |
| R524 | 58 | P | *Z. caespitosa* | 77732 | 77789 | *Z. caespitosa* | 184194 | 184251 | 0 | 5.00E-25 | *_* | *orf130b* |
| R525 | 58 | P | *Z. caespitosa* | 77732 | 77789 | *Z. caespitosa* | 181632 | 181689 | 0 | 5.00E-25 | *_* | *orf103d* |
| R526 | 58 | P | *Z. caespitosa* | 35840 | 35897 | *Z. caespitosa* | 180898 | 180955 | 0 | 5.00E-25 | *_* | *_* |
| R527 | 58 | P | *Z. caespitosa* | 77670 | 77727 | *Z. caespitosa* | 177967 | 178024 | 0 | 5.00E-25 | *_* | *_* |
| R528 | 58 | P | *Z. caespitosa* | 1624 | 1681 | *Z. caespitosa* | 162589 | 162646 | -2 | 7.45E-21 | *_* | *_* |
| R529 | 58 | P | *Z. caespitosa* | 43572 | 43629 | *Z. caespitosa* | 162589 | 162646 | -2 | 7.45E-21 | *_* | *_* |
| R530 | 58 | P | *Z. caespitosa* | 136927 | 136984 | *Z. caespitosa* | 140107 | 140164 | 0 | 5.00E-25 | *_* | *matR* |
| R531 | 58 | P | *Z. caespitosa* | 72613 | 72670 | *Z. caespitosa* | 140107 | 140164 | 0 | 5.00E-25 | *_* | *matR* |
| R532 | 58 | P | *Z. caespitosa* | 83988 | 84045 | *Z. caespitosa* | 140107 | 140164 | 0 | 5.00E-25 | *_* | *matR* |
| R533 | 58 | P | *Z. caespitosa* | 77670 | 77727 | *Z. caespitosa* | 136927 | 136984 | 0 | 5.00E-25 | *_* | *_* |
| R534 | 58 | P | *Z. caespitosa* | 77670 | 77727 | *Z. caespitosa* | 83988 | 84045 | 0 | 5.00E-25 | *_* | *_* |
| R535 | 58 | P | *Z. caespitosa* | 51668 | 51725 | *Z. caespitosa* | 79510 | 79567 | -2 | 7.45E-21 | *orf106b;ccmFn* | *_* |
| R536 | 58 | P | *Z. caespitosa* | 75902 | 75959 | *Z. caespitosa* | 79452 | 79509 | -1 | 8.71E-23 | *_* | *_* |
| R537 | 58 | P | *Z. caespitosa* | 75902 | 75959 | *Z. caespitosa* | 77732 | 77789 | 0 | 5.00E-25 | *_* | *_* |
| R538 | 58 | P | *Z. caespitosa* | 72613 | 72670 | *Z. caespitosa* | 77670 | 77727 | 0 | 5.00E-25 | *_* | *_* |
| R539 | 58 | P | *Z. caespitosa* | 16060 | 16117 | *Z. caespitosa* | 53044 | 53101 | -1 | 8.71E-23 | *orf284a* | *_* |
| R540 | 57 | F | *Z. caespitosa* | 37750 | 37806 | *Z. caespitosa* | 136942 | 136998 | -1 | 3.42E-22 | *_* | *_* |
| R541 | 57 | F | *Z. caespitosa* | 14626 | 14682 | *Z. caespitosa* | 25060 | 25116 | -3 | 1.58E-18 | *orf113a;orf295a;cox2* | *orf340a* |
| R542 | 57 | F | *Z. caespitosa* | 14626 | 14682 | *Z. caespitosa* | 95790 | 95846 | -3 | 1.58E-18 | *orf113a;orf295a;cox2* | *orf540a-2* |
| R543 | 57 | F | *Z. caespitosa* | 84184 | 84240 | *Z. caespitosa* | 137052 | 137108 | -1 | 3.42E-22 | *_* | *_* |
| R544 | 57 | F | *Z. caespitosa* | 84195 | 84251 | *Z. caespitosa* | 137001 | 137057 | 0 | 2.00E-24 | *_* | *_* |
| R545 | 57 | P | *Z. caespitosa* | 83366 | 83422 | *Z. caespitosa* | 175058 | 175114 | -1 | 3.42E-22 | *_* | *_* |
| R546 | 57 | P | *Z. caespitosa* | 84195 | 84251 | *Z. caespitosa* | 164734 | 164790 | 0 | 2.00E-24 | *_* | *_* |
| R547 | 57 | P | *Z. caespitosa* | 84184 | 84240 | *Z. caespitosa* | 164683 | 164739 | -1 | 3.42E-22 | *_* | *_* |
| R548 | 57 | P | *Z. caespitosa* | 14626 | 14682 | *Z. caespitosa* | 122190 | 122246 | -3 | 1.58E-18 | *orf113a;orf295a;cox2* | *nad5* |
| R549 | 57 | P | *Z. caespitosa* | 14626 | 14682 | *Z. caespitosa* | 115909 | 115965 | -3 | 1.58E-18 | *orf113a;orf295a;cox2* | *orf540a-1* |
| R550 | 57 | P | *Z. caespitosa* | 37734 | 37790 | *Z. caespitosa* | 87373 | 87429 | 0 | 2.00E-24 | *_* | *orf122a* |
| R551 | 57 | P | *Z. caespitosa* | 33021 | 33077 | *Z. caespitosa* | 83355 | 83411 | 0 | 2.00E-24 | *_* | *_* |
| R552 | 57 | P | *Z. caespitosa* | 53051 | 53107 | *Z. caespitosa* | 83355 | 83411 | 0 | 2.00E-24 | *_* | *_* |
| R553 | 57 | P | *Z. caespitosa* | 72623 | 72679 | *Z. caespitosa* | 79443 | 79499 | 0 | 2.00E-24 | *_* | *_* |
| R554 | 56 | F | *Z. caespitosa* | 75757 | 75812 | *Z. caespitosa* | 148697 | 148752 | 0 | 8.01E-24 | *_* | *_* |
| R555 | 56 | F | *Z. caespitosa* | 48664 | 48719 | *Z. caespitosa* | 118898 | 118953 | 0 | 8.01E-24 | *_* | *_* |
| R556 | 56 | F | *Z. caespitosa* | 218 | 273 | *Z. caespitosa* | 112135 | 112190 | -2 | 1.11E-19 | *_* | *orf113c-1* |
| R557 | 56 | F | *Z. caespitosa* | 87358 | 87413 | *Z. caespitosa* | 172525 | 172580 | -3 | 5.99E-18 | *orf122a* | *_* |
| R558 | 56 | F | *Z. caespitosa* | 69805 | 69860 | *Z. caespitosa* | 127609 | 127664 | -3 | 5.99E-18 | *_* | *orf149b* |
| R559 | 56 | P | *Z. caespitosa* | 87358 | 87413 | *Z. caespitosa* | 184209 | 184264 | -3 | 5.99E-18 | *orf122a* | *orf130b* |
| R560 | 56 | P | *Z. caespitosa* | 69856 | 69911 | *Z. caespitosa* | 180593 | 180648 | -2 | 1.11E-19 | *_* | *_* |
| R561 | 56 | P | *Z. caespitosa* | 48664 | 48719 | *Z. caespitosa* | 180562 | 180617 | 0 | 8.01E-24 | *_* | *_* |
| R562 | 56 | P | *Z. caespitosa* | 148697 | 148752 | *Z. caespitosa* | 172685 | 172740 | 0 | 8.01E-24 | *_* | *orf185a* |
| R563 | 56 | P | *Z. caespitosa* | 123147 | 123202 | *Z. caespitosa* | 149095 | 149150 | 0 | 8.01E-24 | *orf103c* | *_* |
| R564 | 56 | P | *Z. caespitosa* | 75678 | 75733 | *Z. caespitosa* | 138658 | 138713 | -3 | 5.99E-18 | *_* | *_* |
| R565 | 56 | P | *Z. caespitosa* | 218 | 273 | *Z. caespitosa* | 99566 | 99621 | -2 | 1.11E-19 | *_* | *orf113c-2* |
| R566 | 56 | P | *Z. caespitosa* | 75917 | 75972 | *Z. caespitosa* | 87358 | 87413 | -3 | 5.99E-18 | *_* | *orf122a* |
| R567 | 56 | P | *Z. caespitosa* | 22072 | 22127 | *Z. caespitosa* | 48664 | 48719 | 0 | 8.01E-24 | *_* | *_* |
| R568 | 56 | P | *Z. caespitosa* | 29904 | 29959 | *Z. caespitosa* | 40254 | 40309 | 0 | 8.01E-24 | *_* | *orf309a* |
| R569 | 55 | F | *Z. caespitosa* | 56670 | 56724 | *Z. caespitosa* | 74027 | 74081 | -3 | 2.27E-17 | *rrn18* | *_* |
| R570 | 55 | F | *Z. caespitosa* | 177685 | 177739 | *Z. caespitosa* | 180768 | 180822 | 0 | 3.20E-23 | *_* | *_* |
| R571 | 55 | F | *Z. caespitosa* | 4223 | 4277 | *Z. caespitosa* | 177685 | 177739 | 0 | 3.20E-23 | *_* | *_* |
| R572 | 55 | F | *Z. caespitosa* | 83677 | 83731 | *Z. caespitosa* | 177685 | 177739 | 0 | 3.20E-23 | *_* | *_* |
| R573 | 55 | F | *Z. caespitosa* | 4312 | 4366 | *Z. caespitosa* | 177685 | 177739 | 0 | 3.20E-23 | *_* | *_* |
| R574 | 55 | F | *Z. caespitosa* | 177685 | 177739 | *Z. caespitosa* | 177745 | 177799 | 0 | 3.20E-23 | *_* | *_* |
| R575 | 55 | F | *Z. caespitosa* | 83766 | 83820 | *Z. caespitosa* | 177685 | 177739 | 0 | 3.20E-23 | *_* | *_* |
| R576 | 55 | F | *Z. caespitosa* | 4401 | 4455 | *Z. caespitosa* | 177685 | 177739 | 0 | 3.20E-23 | *_* | *_* |
| R577 | 55 | F | *Z. caespitosa* | 180738 | 180792 | *Z. caespitosa* | 180768 | 180822 | 0 | 3.20E-23 | *_* | *_* |
| R578 | 55 | F | *Z. caespitosa* | 4223 | 4277 | *Z. caespitosa* | 180738 | 180792 | 0 | 3.20E-23 | *_* | *_* |
| R579 | 55 | F | *Z. caespitosa* | 83677 | 83731 | *Z. caespitosa* | 180738 | 180792 | 0 | 3.20E-23 | *_* | *_* |
| R580 | 55 | F | *Z. caespitosa* | 4312 | 4366 | *Z. caespitosa* | 180738 | 180792 | 0 | 3.20E-23 | *_* | *_* |
| R581 | 55 | F | *Z. caespitosa* | 177745 | 177799 | *Z. caespitosa* | 180738 | 180792 | 0 | 3.20E-23 | *_* | *_* |
| R582 | 55 | F | *Z. caespitosa* | 83766 | 83820 | *Z. caespitosa* | 180738 | 180792 | 0 | 3.20E-23 | *_* | *_* |
| R583 | 55 | F | *Z. caespitosa* | 4401 | 4455 | *Z. caespitosa* | 180738 | 180792 | 0 | 3.20E-23 | *_* | *_* |
| R584 | 55 | F | *Z. caespitosa* | 4193 | 4247 | *Z. caespitosa* | 180768 | 180822 | 0 | 3.20E-23 | *_* | *_* |
| R585 | 55 | F | *Z. caespitosa* | 4193 | 4247 | *Z. caespitosa* | 4223 | 4277 | 0 | 3.20E-23 | *_* | *_* |
| R586 | 55 | F | *Z. caespitosa* | 4193 | 4247 | *Z. caespitosa* | 83677 | 83731 | 0 | 3.20E-23 | *_* | *_* |
| R587 | 55 | F | *Z. caespitosa* | 4193 | 4247 | *Z. caespitosa* | 4312 | 4366 | 0 | 3.20E-23 | *_* | *_* |
| R588 | 55 | F | *Z. caespitosa* | 4193 | 4247 | *Z. caespitosa* | 177745 | 177799 | 0 | 3.20E-23 | *_* | *_* |
| R589 | 55 | F | *Z. caespitosa* | 4193 | 4247 | *Z. caespitosa* | 83766 | 83820 | 0 | 3.20E-23 | *_* | *_* |
| R590 | 55 | F | *Z. caespitosa* | 4193 | 4247 | *Z. caespitosa* | 4401 | 4455 | 0 | 3.20E-23 | *_* | *_* |
| R591 | 55 | F | *Z. caespitosa* | 83647 | 83701 | *Z. caespitosa* | 180768 | 180822 | 0 | 3.20E-23 | *_* | *_* |
| R592 | 55 | F | *Z. caespitosa* | 4223 | 4277 | *Z. caespitosa* | 83647 | 83701 | 0 | 3.20E-23 | *_* | *_* |
| R593 | 55 | F | *Z. caespitosa* | 83647 | 83701 | *Z. caespitosa* | 83677 | 83731 | 0 | 3.20E-23 | *_* | *_* |
| R594 | 55 | F | *Z. caespitosa* | 4312 | 4366 | *Z. caespitosa* | 83647 | 83701 | 0 | 3.20E-23 | *_* | *_* |
| R595 | 55 | F | *Z. caespitosa* | 83647 | 83701 | *Z. caespitosa* | 177745 | 177799 | 0 | 3.20E-23 | *_* | *_* |
| R596 | 55 | F | *Z. caespitosa* | 83647 | 83701 | *Z. caespitosa* | 83766 | 83820 | 0 | 3.20E-23 | *_* | *_* |
| R597 | 55 | F | *Z. caespitosa* | 4401 | 4455 | *Z. caespitosa* | 83647 | 83701 | 0 | 3.20E-23 | *_* | *_* |
| R598 | 55 | F | *Z. caespitosa* | 4282 | 4336 | *Z. caespitosa* | 180768 | 180822 | 0 | 3.20E-23 | *_* | *_* |
| R599 | 55 | F | *Z. caespitosa* | 4223 | 4277 | *Z. caespitosa* | 4282 | 4336 | 0 | 3.20E-23 | *_* | *_* |
| R600 | 55 | F | *Z. caespitosa* | 4282 | 4336 | *Z. caespitosa* | 83677 | 83731 | 0 | 3.20E-23 | *_* | *_* |
| R601 | 55 | F | *Z. caespitosa* | 4282 | 4336 | *Z. caespitosa* | 4312 | 4366 | 0 | 3.20E-23 | *_* | *_* |
| R602 | 55 | F | *Z. caespitosa* | 4282 | 4336 | *Z. caespitosa* | 177745 | 177799 | 0 | 3.20E-23 | *_* | *_* |
| R603 | 55 | F | *Z. caespitosa* | 4282 | 4336 | *Z. caespitosa* | 83766 | 83820 | 0 | 3.20E-23 | *_* | *_* |
| R604 | 55 | F | *Z. caespitosa* | 4282 | 4336 | *Z. caespitosa* | 4401 | 4455 | 0 | 3.20E-23 | *_* | *_* |
| R605 | 55 | F | *Z. caespitosa* | 83736 | 83790 | *Z. caespitosa* | 180768 | 180822 | 0 | 3.20E-23 | *_* | *_* |
| R606 | 55 | F | *Z. caespitosa* | 4223 | 4277 | *Z. caespitosa* | 83736 | 83790 | 0 | 3.20E-23 | *_* | *_* |
| R607 | 55 | F | *Z. caespitosa* | 83677 | 83731 | *Z. caespitosa* | 83736 | 83790 | 0 | 3.20E-23 | *_* | *_* |
| R608 | 55 | F | *Z. caespitosa* | 4312 | 4366 | *Z. caespitosa* | 83736 | 83790 | 0 | 3.20E-23 | *_* | *_* |
| R609 | 55 | F | *Z. caespitosa* | 83736 | 83790 | *Z. caespitosa* | 177745 | 177799 | 0 | 3.20E-23 | *_* | *_* |
| R610 | 55 | F | *Z. caespitosa* | 83736 | 83790 | *Z. caespitosa* | 83766 | 83820 | 0 | 3.20E-23 | *_* | *_* |
| R611 | 55 | F | *Z. caespitosa* | 4401 | 4455 | *Z. caespitosa* | 83736 | 83790 | 0 | 3.20E-23 | *_* | *_* |
| R612 | 55 | F | *Z. caespitosa* | 4371 | 4425 | *Z. caespitosa* | 180768 | 180822 | 0 | 3.20E-23 | *_* | *_* |
| R613 | 55 | F | *Z. caespitosa* | 4223 | 4277 | *Z. caespitosa* | 4371 | 4425 | 0 | 3.20E-23 | *_* | *_* |
| R614 | 55 | F | *Z. caespitosa* | 4371 | 4425 | *Z. caespitosa* | 83677 | 83731 | 0 | 3.20E-23 | *_* | *_* |
| R615 | 55 | F | *Z. caespitosa* | 4312 | 4366 | *Z. caespitosa* | 4371 | 4425 | 0 | 3.20E-23 | *_* | *_* |
| R616 | 55 | F | *Z. caespitosa* | 4371 | 4425 | *Z. caespitosa* | 177745 | 177799 | 0 | 3.20E-23 | *_* | *_* |
| R617 | 55 | F | *Z. caespitosa* | 4371 | 4425 | *Z. caespitosa* | 83766 | 83820 | 0 | 3.20E-23 | *_* | *_* |
| R618 | 55 | F | *Z. caespitosa* | 4371 | 4425 | *Z. caespitosa* | 4401 | 4455 | 0 | 3.20E-23 | *_* | *_* |
| R619 | 55 | F | *Z. caespitosa* | 177715 | 177769 | *Z. caespitosa* | 177804 | 177858 | 0 | 3.20E-23 | *_* | *_* |
| R620 | 55 | F | *Z. caespitosa* | 83825 | 83879 | *Z. caespitosa* | 177715 | 177769 | 0 | 3.20E-23 | *_* | *_* |
| R621 | 55 | F | *Z. caespitosa* | 4460 | 4514 | *Z. caespitosa* | 177715 | 177769 | 0 | 3.20E-23 | *_* | *_* |
| R622 | 55 | F | *Z. caespitosa* | 24948 | 25002 | *Z. caespitosa* | 138822 | 138876 | 0 | 3.20E-23 | *_* | *_* |
| R623 | 55 | F | *Z. caespitosa* | 95678 | 95732 | *Z. caespitosa* | 138822 | 138876 | 0 | 3.20E-23 | *_* | *_* |
| R624 | 55 | F | *Z. caespitosa* | 25496 | 25550 | *Z. caespitosa* | 176027 | 176081 | -1 | 5.29E-21 | *orf340a* | *_* |
| R625 | 55 | F | *Z. caespitosa* | 96226 | 96280 | *Z. caespitosa* | 176027 | 176081 | -1 | 5.29E-21 | *orf540a-2* | *_* |
| R626 | 55 | F | *Z. caespitosa* | 9932 | 9986 | *Z. caespitosa* | 10121 | 10175 | -3 | 2.27E-17 | *orf397a* | *orf397a* |
| R627 | 55 | F | *Z. caespitosa* | 9932 | 9986 | *Z. caespitosa* | 9995 | 10049 | -3 | 2.27E-17 | *orf397a* | *orf397a* |
| R628 | 55 | F | *Z. caespitosa* | 35381 | 35435 | *Z. caespitosa* | 84949 | 85003 | 0 | 3.20E-23 | *_* | *_* |
| R629 | 55 | F | *Z. caespitosa* | 74666 | 74720 | *Z. caespitosa* | 87359 | 87413 | -1 | 5.29E-21 | *_* | *orf122a* |
| R630 | 55 | P | *Z. caespitosa* | 87359 | 87413 | *Z. caespitosa* | 181647 | 181701 | -3 | 2.27E-17 | *orf122a* | *orf103d* |
| R631 | 55 | P | *Z. caespitosa* | 115475 | 115529 | *Z. caespitosa* | 176027 | 176081 | -1 | 5.29E-21 | *orf540a-1* | *_* |
| R632 | 55 | P | *Z. caespitosa* | 116023 | 116077 | *Z. caespitosa* | 138822 | 138876 | 0 | 3.20E-23 | *_* | *_* |
| R633 | 54 | F | *Z. caespitosa* | 37750 | 37803 | *Z. caespitosa* | 72628 | 72681 | -1 | 2.08E-20 | *_* | *_* |
| R634 | 54 | F | *Z. caespitosa* | 37750 | 37803 | *Z. caespitosa* | 177982 | 178035 | -1 | 2.08E-20 | *_* | *_* |
| R635 | 54 | F | *Z. caespitosa* | 156587 | 156640 | *Z. caespitosa* | 156617 | 156670 | -2 | 1.65E-18 | *_* | *_* |
| R636 | 54 | F | *Z. caespitosa* | 4135 | 4188 | *Z. caespitosa* | 177686 | 177739 | 0 | 1.28E-22 | *_* | *_* |
| R637 | 54 | F | *Z. caespitosa* | 83589 | 83642 | *Z. caespitosa* | 177686 | 177739 | 0 | 1.28E-22 | *_* | *_* |
| R638 | 54 | F | *Z. caespitosa* | 4135 | 4188 | *Z. caespitosa* | 180739 | 180792 | 0 | 1.28E-22 | *_* | *_* |
| R639 | 54 | F | *Z. caespitosa* | 83589 | 83642 | *Z. caespitosa* | 180739 | 180792 | 0 | 1.28E-22 | *_* | *_* |
| R640 | 54 | F | *Z. caespitosa* | 4135 | 4188 | *Z. caespitosa* | 4194 | 4247 | 0 | 1.28E-22 | *_* | *_* |
| R641 | 54 | F | *Z. caespitosa* | 4194 | 4247 | *Z. caespitosa* | 83589 | 83642 | 0 | 1.28E-22 | *_* | *_* |
| R642 | 54 | F | *Z. caespitosa* | 4135 | 4188 | *Z. caespitosa* | 83648 | 83701 | 0 | 1.28E-22 | *_* | *_* |
| R643 | 54 | F | *Z. caespitosa* | 83589 | 83642 | *Z. caespitosa* | 83648 | 83701 | 0 | 1.28E-22 | *_* | *_* |
| R644 | 54 | F | *Z. caespitosa* | 4135 | 4188 | *Z. caespitosa* | 4283 | 4336 | 0 | 1.28E-22 | *_* | *_* |
| R645 | 54 | F | *Z. caespitosa* | 4283 | 4336 | *Z. caespitosa* | 83589 | 83642 | 0 | 1.28E-22 | *_* | *_* |
| R646 | 54 | F | *Z. caespitosa* | 4135 | 4188 | *Z. caespitosa* | 177716 | 177769 | 0 | 1.28E-22 | *_* | *_* |
| R647 | 54 | F | *Z. caespitosa* | 83589 | 83642 | *Z. caespitosa* | 177716 | 177769 | 0 | 1.28E-22 | *_* | *_* |
| R648 | 54 | F | *Z. caespitosa* | 4135 | 4188 | *Z. caespitosa* | 83737 | 83790 | 0 | 1.28E-22 | *_* | *_* |
| R649 | 54 | F | *Z. caespitosa* | 83589 | 83642 | *Z. caespitosa* | 83737 | 83790 | 0 | 1.28E-22 | *_* | *_* |
| R650 | 54 | F | *Z. caespitosa* | 4135 | 4188 | *Z. caespitosa* | 4372 | 4425 | 0 | 1.28E-22 | *_* | *_* |
| R651 | 54 | F | *Z. caespitosa* | 4372 | 4425 | *Z. caespitosa* | 83589 | 83642 | 0 | 1.28E-22 | *_* | *_* |
| R652 | 54 | F | *Z. caespitosa* | 46081 | 46134 | *Z. caespitosa* | 177805 | 177858 | 0 | 1.28E-22 | *_* | *_* |
| R653 | 54 | F | *Z. caespitosa* | 46081 | 46134 | *Z. caespitosa* | 83826 | 83879 | 0 | 1.28E-22 | *_* | *_* |
| R654 | 54 | F | *Z. caespitosa* | 4461 | 4514 | *Z. caespitosa* | 46081 | 46134 | 0 | 1.28E-22 | *_* | *_* |
| R655 | 54 | F | *Z. caespitosa* | 46081 | 46134 | *Z. caespitosa* | 180769 | 180822 | 0 | 1.28E-22 | *_* | *_* |
| R656 | 54 | F | *Z. caespitosa* | 4224 | 4277 | *Z. caespitosa* | 46081 | 46134 | 0 | 1.28E-22 | *_* | *_* |
| R657 | 54 | F | *Z. caespitosa* | 46081 | 46134 | *Z. caespitosa* | 83678 | 83731 | 0 | 1.28E-22 | *_* | *_* |
| R658 | 54 | F | *Z. caespitosa* | 4313 | 4366 | *Z. caespitosa* | 46081 | 46134 | 0 | 1.28E-22 | *_* | *_* |
| R659 | 54 | F | *Z. caespitosa* | 46081 | 46134 | *Z. caespitosa* | 177746 | 177799 | 0 | 1.28E-22 | *_* | *_* |
| R660 | 54 | F | *Z. caespitosa* | 46081 | 46134 | *Z. caespitosa* | 83767 | 83820 | 0 | 1.28E-22 | *_* | *_* |
| R661 | 54 | F | *Z. caespitosa* | 4402 | 4455 | *Z. caespitosa* | 46081 | 46134 | 0 | 1.28E-22 | *_* | *_* |
| R662 | 54 | F | *Z. caespitosa* | 138729 | 138782 | *Z. caespitosa* | 138786 | 138839 | 0 | 1.28E-22 | *_* | *_* |
| R663 | 54 | F | *Z. caespitosa* | 138742 | 138795 | *Z. caespitosa* | 138823 | 138876 | 0 | 1.28E-22 | *_* | *_* |
| R664 | 54 | F | *Z. caespitosa* | 138823 | 138876 | *Z. caespitosa* | 138885 | 138938 | 0 | 1.28E-22 | *_* | *_* |
| R665 | 54 | F | *Z. caespitosa* | 15950 | 16003 | *Z. caespitosa* | 23213 | 23266 | -1 | 2.08E-20 | *orf284a* | *_* |
| R666 | 54 | F | *Z. caespitosa* | 7583 | 7636 | *Z. caespitosa* | 23213 | 23266 | -1 | 2.08E-20 | *_* | *_* |
| R667 | 54 | F | *Z. caespitosa* | 15950 | 16003 | *Z. caespitosa* | 93943 | 93996 | -1 | 2.08E-20 | *orf284a* | *_* |
| R668 | 54 | F | *Z. caespitosa* | 7583 | 7636 | *Z. caespitosa* | 93943 | 93996 | -1 | 2.08E-20 | *_* | *_* |
| R669 | 54 | F | *Z. caespitosa* | 74680 | 74733 | *Z. caespitosa* | 77734 | 77787 | 0 | 1.28E-22 | *_* | *_* |
| R670 | 54 | F | *Z. caespitosa* | 74680 | 74733 | *Z. caespitosa* | 77672 | 77725 | 0 | 1.28E-22 | *_* | *_* |
| R671 | 54 | F | *Z. caespitosa* | 77659 | 77712 | *Z. caespitosa* | 87360 | 87413 | -2 | 1.65E-18 | *_* | *orf122a* |
| R672 | 54 | P | *Z. caespitosa* | 22074 | 22127 | *Z. caespitosa* | 172966 | 173019 | 0 | 1.28E-22 | *_* | *orf185a* |
| R673 | 54 | P | *Z. caespitosa* | 15950 | 16003 | *Z. caespitosa* | 117759 | 117812 | -1 | 2.08E-20 | *orf284a* | *_* |
| R674 | 54 | P | *Z. caespitosa* | 7583 | 7636 | *Z. caespitosa* | 117759 | 117812 | -1 | 2.08E-20 | *_* | *_* |
| R675 | 54 | P | *Z. caespitosa* | 57664 | 57717 | *Z. caespitosa* | 101645 | 101698 | -3 | 8.58E-17 | *orf117a* | *_* |
| R676 | 54 | P | *Z. caespitosa* | 37750 | 37803 | *Z. caespitosa* | 77783 | 77836 | -1 | 2.08E-20 | *_* | *_* |
| R677 | 54 | P | *Z. caespitosa* | 37750 | 37803 | *Z. caespitosa* | 77721 | 77774 | -1 | 2.08E-20 | *_* | *_* |
| R678 | 54 | P | *Z. caespitosa* | 14556 | 14609 | *Z. caespitosa* | 33018 | 33071 | -1 | 2.08E-20 | *orf113a* | *_* |
| R679 | 53 | F | *Z. caespitosa* | 71945 | 71997 | *Z. caespitosa* | 79523 | 79575 | 0 | 5.12E-22 | *_* | *_* |
| R680 | 53 | F | *Z. caespitosa* | 37750 | 37802 | *Z. caespitosa* | 84003 | 84055 | -1 | 8.15E-20 | *_* | *_* |
| R681 | 53 | F | *Z. caespitosa* | 51695 | 51747 | *Z. caespitosa* | 184148 | 184200 | -3 | 3.24E-16 | *orf106b;ccmFn* | *orf130b* |
| R682 | 53 | F | *Z. caespitosa* | 9832 | 9884 | *Z. caespitosa* | 9958 | 10010 | -3 | 3.24E-16 | *orf397a* | *orf397a* |
| R683 | 53 | F | *Z. caespitosa* | 14561 | 14613 | *Z. caespitosa* | 15972 | 16024 | -3 | 3.24E-16 | *orf113a* | *orf284a* |
| R684 | 53 | F | *Z. caespitosa* | 7605 | 7657 | *Z. caespitosa* | 14561 | 14613 | -3 | 3.24E-16 | *_* | *orf113a* |
| R685 | 53 | F | *Z. caespitosa* | 16065 | 16117 | *Z. caespitosa* | 149191 | 149243 | -3 | 3.24E-16 | *orf284a* | *_* |
| R686 | 53 | F | *Z. caespitosa* | 153283 | 153335 | *Z. caespitosa* | 160748 | 160800 | -1 | 8.15E-20 | *_* | *atp9* |
| R687 | 53 | F | *Z. caespitosa* | 52592 | 52644 | *Z. caespitosa* | 186407 | 186459 | 0 | 5.12E-22 | *orf172a* | *orf100b* |
| R688 | 53 | F | *Z. caespitosa* | 22162 | 22214 | *Z. caespitosa* | 33020 | 33072 | 0 | 5.12E-22 | *_* | *_* |
| R689 | 53 | P | *Z. caespitosa* | 37876 | 37928 | *Z. caespitosa* | 162992 | 163044 | -1 | 8.15E-20 | *_* | *_* |
| R690 | 53 | P | *Z. caespitosa* | 156587 | 156639 | *Z. caespitosa* | 162771 | 162823 | 0 | 5.12E-22 | *_* | *_* |
| R691 | 53 | P | *Z. caespitosa* | 75681 | 75733 | *Z. caespitosa* | 162663 | 162715 | -3 | 3.24E-16 | *_* | *_* |
| R692 | 53 | P | *Z. caespitosa* | 16138 | 16190 | *Z. caespitosa* | 156587 | 156639 | 0 | 5.12E-22 | *orf284a* | *_* |
| R693 | 53 | P | *Z. caespitosa* | 53044 | 53096 | *Z. caespitosa* | 149191 | 149243 | -3 | 3.24E-16 | *_* | *_* |
| R694 | 53 | P | *Z. caespitosa* | 33020 | 33072 | *Z. caespitosa* | 118811 | 118863 | 0 | 5.12E-22 | *_* | *_* |
| R695 | 53 | P | *Z. caespitosa* | 75681 | 75733 | *Z. caespitosa* | 101646 | 101698 | 0 | 5.12E-22 | *_* | *_* |
| R696 | 53 | P | *Z. caespitosa* | 37876 | 37928 | *Z. caespitosa* | 79650 | 79702 | 0 | 5.12E-22 | *_* | *_* |
| R697 | 53 | P | *Z. caespitosa* | 69881 | 69933 | *Z. caespitosa* | 75631 | 75683 | -3 | 3.24E-16 | *_* | *_* |
| R698 | 53 | P | *Z. caespitosa* | 4514 | 4566 | *Z. caespitosa* | 15855 | 15907 | 0 | 5.12E-22 | *_* | *_* |
| R699 | 52 | F | *Z. caespitosa* | 14574 | 14625 | *Z. caespitosa* | 25003 | 25054 | -1 | 3.20E-19 | *orf113a* | *_* |
| R700 | 52 | F | *Z. caespitosa* | 14574 | 14625 | *Z. caespitosa* | 95733 | 95784 | -1 | 3.20E-19 | *orf113a* | *_* |
| R701 | 52 | F | *Z. caespitosa* | 1588 | 1639 | *Z. caespitosa* | 4519 | 4570 | 0 | 2.05E-21 | *_* | *_* |
| R702 | 52 | F | *Z. caespitosa* | 4519 | 4570 | *Z. caespitosa* | 43536 | 43587 | 0 | 2.05E-21 | *_* | *_* |
| R703 | 52 | F | *Z. caespitosa* | 25620 | 25671 | *Z. caespitosa* | 176207 | 176258 | -3 | 1.22E-15 | *orf340a* | *orf110b* |
| R704 | 52 | F | *Z. caespitosa* | 96350 | 96401 | *Z. caespitosa* | 176207 | 176258 | -3 | 1.22E-15 | *orf540a-2* | *orf110b* |
| R705 | 52 | F | *Z. caespitosa* | 14556 | 14607 | *Z. caespitosa* | 118812 | 118863 | -1 | 3.20E-19 | *orf113a* | *_* |
| R706 | 52 | F | *Z. caespitosa* | 22163 | 22214 | *Z. caespitosa* | 53051 | 53102 | 0 | 2.05E-21 | *_* | *_* |
| R707 | 52 | F | *Z. caespitosa* | 51620 | 51671 | *Z. caespitosa* | 51674 | 51725 | -3 | 1.22E-15 | *orf106b;ccmFn* | *orf106b;ccmFn* |
| R708 | 52 | F | *Z. caespitosa* | 15966 | 16017 | *Z. caespitosa* | 149185 | 149236 | -1 | 3.20E-19 | *orf284a* | *_* |
| R709 | 52 | F | *Z. caespitosa* | 7599 | 7650 | *Z. caespitosa* | 149185 | 149236 | -1 | 3.20E-19 | *_* | *_* |
| R710 | 52 | F | *Z. caespitosa* | 14717 | 14768 | *Z. caespitosa* | 25151 | 25202 | -2 | 2.45E-17 | *orf295a;cox2* | *orf340a* |
| R711 | 52 | F | *Z. caespitosa* | 14717 | 14768 | *Z. caespitosa* | 95881 | 95932 | -2 | 2.45E-17 | *orf295a;cox2* | *orf540a-2* |
| R712 | 52 | P | *Z. caespitosa* | 72256 | 72307 | *Z. caespitosa* | 186461 | 186512 | 0 | 2.05E-21 | *_* | *orf100b* |
| R713 | 52 | P | *Z. caespitosa* | 115354 | 115405 | *Z. caespitosa* | 176207 | 176258 | -3 | 1.22E-15 | *orf540a-1* | *orf110b* |
| R714 | 52 | P | *Z. caespitosa* | 15966 | 16017 | *Z. caespitosa* | 175069 | 175120 | -3 | 1.22E-15 | *orf284a* | *_* |
| R715 | 52 | P | *Z. caespitosa* | 7599 | 7650 | *Z. caespitosa* | 175069 | 175120 | -3 | 1.22E-15 | *_* | *_* |
| R716 | 52 | P | *Z. caespitosa* | 48750 | 48801 | *Z. caespitosa* | 155611 | 155662 | 0 | 2.05E-21 | *_* | *_* |
| R717 | 52 | P | *Z. caespitosa* | 53051 | 53102 | *Z. caespitosa* | 118811 | 118862 | 0 | 2.05E-21 | *_* | *_* |
| R718 | 52 | P | *Z. caespitosa* | 14574 | 14625 | *Z. caespitosa* | 115971 | 116022 | -1 | 3.20E-19 | *orf113a* | *_* |
| R719 | 52 | P | *Z. caespitosa* | 14717 | 14768 | *Z. caespitosa* | 115823 | 115874 | -2 | 2.45E-17 | *orf295a;cox2* | *orf540a-1* |
| R720 | 52 | P | *Z. caespitosa* | 37750 | 37801 | *Z. caespitosa* | 79443 | 79494 | -1 | 3.20E-19 | *_* | *_* |
| R721 | 52 | P | *Z. caespitosa* | 14556 | 14607 | *Z. caespitosa* | 22162 | 22213 | -1 | 3.20E-19 | *orf113a* | *_* |
| R722 | 51 | F | *Z. caespitosa* | 25716 | 25766 | *Z. caespitosa* | 176055 | 176105 | -1 | 1.25E-18 | *orf340a* | *orf110b* |
| R723 | 51 | F | *Z. caespitosa* | 96446 | 96496 | *Z. caespitosa* | 176055 | 176105 | -1 | 1.25E-18 | *orf540a-2* | *orf110b* |
| R724 | 51 | F | *Z. caespitosa* | 40431 | 40481 | *Z. caespitosa* | 126677 | 126727 | -3 | 4.61E-15 | *orf309a* | *orf242a* |
| R725 | 51 | F | *Z. caespitosa* | 16060 | 16110 | *Z. caespitosa* | 118812 | 118862 | -1 | 1.25E-18 | *orf284a* | *_* |
| R726 | 51 | F | *Z. caespitosa* | 14556 | 14606 | *Z. caespitosa* | 83361 | 83411 | -1 | 1.25E-18 | *orf113a* | *_* |
| R727 | 51 | F | *Z. caespitosa* | 16060 | 16110 | *Z. caespitosa* | 83361 | 83411 | -1 | 1.25E-18 | *orf284a* | *_* |
| R728 | 51 | F | *Z. caespitosa* | 108747 | 108797 | *Z. caespitosa* | 181555 | 181605 | -1 | 1.25E-18 | *orf161a* | *orf103d* |
| R729 | 51 | F | *Z. caespitosa* | 108747 | 108797 | *Z. caespitosa* | 136850 | 136900 | -1 | 1.25E-18 | *orf161a* | *_* |
| R730 | 51 | F | *Z. caespitosa* | 108747 | 108797 | *Z. caespitosa* | 177890 | 177940 | -1 | 1.25E-18 | *orf161a* | *_* |
| R731 | 51 | F | *Z. caespitosa* | 83911 | 83961 | *Z. caespitosa* | 108747 | 108797 | -1 | 1.25E-18 | *_* | *orf161a* |
| R732 | 51 | F | *Z. caespitosa* | 41430 | 41480 | *Z. caespitosa* | 68902 | 68952 | -1 | 1.25E-18 | *orf120b* | *_* |
| R733 | 51 | F | *Z. caespitosa* | 9155 | 9205 | *Z. caespitosa* | 9795 | 9845 | -2 | 9.41E-17 | *orf397a* | *orf397a* |
| R734 | 51 | F | *Z. caespitosa* | 57238 | 57288 | *Z. caespitosa* | 74351 | 74401 | -1 | 1.25E-18 | *rrn18;orf152a* | *_* |
| R735 | 51 | F | *Z. caespitosa* | 118833 | 118883 | *Z. caespitosa* | 172886 | 172936 | 0 | 8.20E-21 | *_* | *orf185a* |
| R736 | 51 | P | *Z. caespitosa* | 72256 | 72306 | *Z. caespitosa* | 181566 | 181616 | 0 | 8.20E-21 | *_* | *orf103d* |
| R737 | 51 | P | *Z. caespitosa* | 79663 | 79713 | *Z. caespitosa* | 181555 | 181605 | 0 | 8.20E-21 | *_* | *orf103d* |
| R738 | 51 | P | *Z. caespitosa* | 163005 | 163055 | *Z. caespitosa* | 181555 | 181605 | -1 | 1.25E-18 | *_* | *orf103d* |
| R739 | 51 | P | *Z. caespitosa* | 72256 | 72306 | *Z. caespitosa* | 177901 | 177951 | 0 | 8.20E-21 | *_* | *_* |
| R740 | 51 | P | *Z. caespitosa* | 79663 | 79713 | *Z. caespitosa* | 177890 | 177940 | 0 | 8.20E-21 | *_* | *_* |
| R741 | 51 | P | *Z. caespitosa* | 163005 | 163055 | *Z. caespitosa* | 177890 | 177940 | -1 | 1.25E-18 | *_* | *_* |
| R742 | 51 | P | *Z. caespitosa* | 115259 | 115309 | *Z. caespitosa* | 176055 | 176105 | -1 | 1.25E-18 | *orf540a-1* | *orf110b* |
| R743 | 51 | P | *Z. caespitosa* | 22142 | 22192 | *Z. caespitosa* | 172886 | 172936 | 0 | 8.20E-21 | *_* | *orf185a* |
| R744 | 51 | P | *Z. caespitosa* | 136850 | 136900 | *Z. caespitosa* | 163005 | 163055 | -1 | 1.25E-18 | *_* | *_* |
| R745 | 51 | P | *Z. caespitosa* | 83911 | 83961 | *Z. caespitosa* | 163005 | 163055 | -1 | 1.25E-18 | *_* | *_* |
| R746 | 51 | P | *Z. caespitosa* | 72256 | 72306 | *Z. caespitosa* | 136861 | 136911 | 0 | 8.20E-21 | *_* | *_* |
| R747 | 51 | P | *Z. caespitosa* | 79663 | 79713 | *Z. caespitosa* | 136850 | 136900 | 0 | 8.20E-21 | *_* | *_* |
| R748 | 51 | P | *Z. caespitosa* | 72256 | 72306 | *Z. caespitosa* | 83922 | 83972 | 0 | 8.20E-21 | *_* | *_* |
| R749 | 51 | P | *Z. caespitosa* | 79663 | 79713 | *Z. caespitosa* | 83911 | 83961 | 0 | 8.20E-21 | *_* | *_* |
| R750 | 51 | P | *Z. caespitosa* | 34915 | 34965 | *Z. caespitosa* | 77623 | 77673 | -3 | 4.61E-15 | *_* | *_* |
| R751 | 51 | P | *Z. caespitosa* | 14556 | 14606 | *Z. caespitosa* | 53051 | 53101 | -1 | 1.25E-18 | *orf113a* | *_* |
| R752 | 51 | P | *Z. caespitosa* | 16060 | 16110 | *Z. caespitosa* | 33021 | 33071 | -1 | 1.25E-18 | *orf284a* | *_* |
| R753 | 51 | P | *Z. caespitosa* | 16060 | 16110 | *Z. caespitosa* | 22163 | 22213 | -1 | 1.25E-18 | *orf284a* | *_* |
| R754 | 50 | F | *Z. caespitosa* | 123196 | 123245 | *Z. caespitosa* | 157567 | 157616 | -1 | 4.92E-18 | *_* | *rrn26* |
| R755 | 50 | F | *Z. caespitosa* | 131511 | 131560 | *Z. caespitosa* | 182584 | 182633 | -3 | 1.74E-14 | *orf131b* | *orf117c* |
| R756 | 50 | F | *Z. caespitosa* | 148748 | 148797 | *Z. caespitosa* | 181555 | 181604 | -1 | 4.92E-18 | *_* | *orf103d* |
| R757 | 50 | F | *Z. caespitosa* | 136850 | 136899 | *Z. caespitosa* | 148748 | 148797 | -1 | 4.92E-18 | *_* | *_* |
| R758 | 50 | F | *Z. caespitosa* | 148748 | 148797 | *Z. caespitosa* | 177890 | 177939 | -1 | 4.92E-18 | *_* | *_* |
| R759 | 50 | F | *Z. caespitosa* | 83911 | 83960 | *Z. caespitosa* | 148748 | 148797 | -1 | 4.92E-18 | *_* | *_* |
| R760 | 50 | F | *Z. caespitosa* | 148811 | 148860 | *Z. caespitosa* | 181555 | 181604 | -1 | 4.92E-18 | *_* | *orf103d* |
| R761 | 50 | F | *Z. caespitosa* | 136850 | 136899 | *Z. caespitosa* | 148811 | 148860 | -1 | 4.92E-18 | *_* | *_* |
| R762 | 50 | F | *Z. caespitosa* | 148811 | 148860 | *Z. caespitosa* | 177890 | 177939 | -1 | 4.92E-18 | *_* | *_* |
| R763 | 50 | F | *Z. caespitosa* | 83911 | 83960 | *Z. caespitosa* | 148811 | 148860 | -1 | 4.92E-18 | *_* | *_* |
| R764 | 50 | F | *Z. caespitosa* | 9129 | 9178 | *Z. caespitosa* | 9958 | 10007 | -3 | 1.74E-14 | *orf397a* | *orf397a* |
| R765 | 50 | F | *Z. caespitosa* | 581 | 630 | *Z. caespitosa* | 1242 | 1291 | -3 | 1.74E-14 | *_* | *_* |
| R766 | 50 | F | *Z. caespitosa* | 581 | 630 | *Z. caespitosa* | 43190 | 43239 | -3 | 1.74E-14 | *_* | *_* |
| R767 | 50 | F | *Z. caespitosa* | 1242 | 1291 | *Z. caespitosa* | 42530 | 42579 | -3 | 1.74E-14 | *_* | *_* |
| R768 | 50 | F | *Z. caespitosa* | 42530 | 42579 | *Z. caespitosa* | 43190 | 43239 | -3 | 1.74E-14 | *_* | *_* |
| R769 | 50 | F | *Z. caespitosa* | 117765 | 117814 | *Z. caespitosa* | 136806 | 136855 | 0 | 3.28E-20 | *_* | *_* |
| R770 | 50 | F | *Z. caespitosa* | 9949 | 9998 | *Z. caespitosa* | 10201 | 10250 | -1 | 4.92E-18 | *orf397a* | *orf397a* |
| R771 | 50 | F | *Z. caespitosa* | 9938 | 9987 | *Z. caespitosa* | 10064 | 10113 | -3 | 1.74E-14 | *orf397a* | *orf397a* |
| R772 | 50 | P | *Z. caespitosa* | 111073 | 111122 | *Z. caespitosa* | 166645 | 166694 | 0 | 3.28E-20 | *nad1* | *_* |
| R773 | 50 | P | *Z. caespitosa* | 79664 | 79713 | *Z. caespitosa* | 148811 | 148860 | -1 | 4.92E-18 | *_* | *_* |
| R774 | 50 | P | *Z. caespitosa* | 79664 | 79713 | *Z. caespitosa* | 148748 | 148797 | -1 | 4.92E-18 | *_* | *_* |
| R775 | 50 | P | *Z. caespitosa* | 23211 | 23260 | *Z. caespitosa* | 136806 | 136855 | 0 | 3.28E-20 | *_* | *_* |
| R776 | 50 | P | *Z. caespitosa* | 93941 | 93990 | *Z. caespitosa* | 136806 | 136855 | 0 | 3.28E-20 | *_* | *_* |
| R777 | 49 | F | *Z. caespitosa* | 118813 | 118861 | *Z. caespitosa* | 184962 | 185010 | -1 | 1.93E-17 | *_* | *_* |
| R778 | 49 | F | *Z. caespitosa* | 83362 | 83410 | *Z. caespitosa* | 184962 | 185010 | -1 | 1.93E-17 | *_* | *_* |
| R779 | 49 | F | *Z. caespitosa* | 85239 | 85287 | *Z. caespitosa* | 172922 | 172970 | 0 | 1.31E-19 | *orf100a* | *orf185a* |
| R780 | 49 | F | *Z. caespitosa* | 77929 | 77977 | *Z. caespitosa* | 172922 | 172970 | 0 | 1.31E-19 | *_* | *orf185a* |
| R781 | 49 | F | *Z. caespitosa* | 40145 | 40193 | *Z. caespitosa* | 126391 | 126439 | -3 | 6.53E-14 | *_* | *_* |
| R782 | 49 | F | *Z. caespitosa* | 40244 | 40292 | *Z. caespitosa* | 126490 | 126538 | -2 | 1.39E-15 | *orf309a* | *orf242a* |
| R783 | 49 | F | *Z. caespitosa* | 53107 | 53155 | *Z. caespitosa* | 181568 | 181616 | -1 | 1.93E-17 | *_* | *orf103d* |
| R784 | 49 | F | *Z. caespitosa* | 53107 | 53155 | *Z. caespitosa* | 136863 | 136911 | -1 | 1.93E-17 | *_* | *_* |
| R785 | 49 | F | *Z. caespitosa* | 53107 | 53155 | *Z. caespitosa* | 177903 | 177951 | -1 | 1.93E-17 | *_* | *_* |
| R786 | 49 | F | *Z. caespitosa* | 53107 | 53155 | *Z. caespitosa* | 83924 | 83972 | -1 | 1.93E-17 | *_* | *_* |
| R787 | 49 | F | *Z. caespitosa* | 37734 | 37782 | *Z. caespitosa* | 156570 | 156618 | 0 | 1.31E-19 | *_* | *_* |
| R788 | 49 | F | *Z. caespitosa* | 36844 | 36892 | *Z. caespitosa* | 140204 | 140252 | 0 | 1.31E-19 | *_* | *matR* |
| R789 | 49 | F | *Z. caespitosa* | 7962 | 8010 | *Z. caespitosa* | 14193 | 14241 | -3 | 6.53E-14 | *_* | *_* |
| R790 | 49 | F | *Z. caespitosa* | 10127 | 10175 | *Z. caespitosa* | 10190 | 10238 | -3 | 6.53E-14 | *orf397a* | *orf397a* |
| R791 | 49 | F | *Z. caespitosa* | 10001 | 10049 | *Z. caespitosa* | 10190 | 10238 | -3 | 6.53E-14 | *orf397a* | *orf397a* |
| R792 | 49 | F | *Z. caespitosa* | 10064 | 10112 | *Z. caespitosa* | 10127 | 10175 | -3 | 6.53E-14 | *orf397a* | *orf397a* |
| R793 | 49 | F | *Z. caespitosa* | 10001 | 10049 | *Z. caespitosa* | 10064 | 10112 | -3 | 6.53E-14 | *orf397a* | *orf397a* |
| R794 | 49 | F | *Z. caespitosa* | 9875 | 9923 | *Z. caespitosa* | 10190 | 10238 | -2 | 1.39E-15 | *orf397a* | *orf397a* |
| R795 | 49 | F | *Z. caespitosa* | 9875 | 9923 | *Z. caespitosa* | 10064 | 10112 | -3 | 6.53E-14 | *orf397a* | *orf397a* |
| R796 | 49 | F | *Z. caespitosa* | 8534 | 8582 | *Z. caespitosa* | 8608 | 8656 | -1 | 1.93E-17 | *_* | *_* |
| R797 | 49 | P | *Z. caespitosa* | 22164 | 22212 | *Z. caespitosa* | 184962 | 185010 | -1 | 1.93E-17 | *_* | *_* |
| R798 | 49 | P | *Z. caespitosa* | 53052 | 53100 | *Z. caespitosa* | 184962 | 185010 | -1 | 1.93E-17 | *_* | *_* |
| R799 | 49 | P | *Z. caespitosa* | 33022 | 33070 | *Z. caespitosa* | 184962 | 185010 | -1 | 1.93E-17 | *_* | *_* |
| R800 | 49 | P | *Z. caespitosa* | 164648 | 164696 | *Z. caespitosa* | 177843 | 177891 | -1 | 1.93E-17 | *_* | *_* |
| R801 | 49 | P | *Z. caespitosa* | 4499 | 4547 | *Z. caespitosa* | 164648 | 164696 | -1 | 1.93E-17 | *_* | *_* |
| R802 | 49 | P | *Z. caespitosa* | 83864 | 83912 | *Z. caespitosa* | 164648 | 164696 | -1 | 1.93E-17 | *_* | *_* |
| R803 | 49 | P | *Z. caespitosa* | 108762 | 108810 | *Z. caespitosa* | 162992 | 163040 | -1 | 1.93E-17 | *orf161a* | *_* |
| R804 | 49 | P | *Z. caespitosa* | 43581 | 43629 | *Z. caespitosa* | 162589 | 162637 | -1 | 1.93E-17 | *_* | *_* |
| R805 | 49 | P | *Z. caespitosa* | 1633 | 1681 | *Z. caespitosa* | 162589 | 162637 | -1 | 1.93E-17 | *_* | *_* |
| R806 | 49 | P | *Z. caespitosa* | 93236 | 93284 | *Z. caespitosa* | 161344 | 161392 | 0 | 1.31E-19 | *_* | *_* |
| R807 | 49 | P | *Z. caespitosa* | 52582 | 52630 | *Z. caespitosa* | 135916 | 135964 | -1 | 1.93E-17 | *orf172a* | *_* |
| R808 | 49 | P | *Z. caespitosa* | 15645 | 15693 | *Z. caespitosa* | 57559 | 57607 | -1 | 1.93E-17 | *_* | *orf117a* |
| R809 | 49 | P | *Z. caespitosa* | 15645 | 15693 | *Z. caespitosa* | 52776 | 52824 | -1 | 1.93E-17 | *_* | *orf172a* |
| R810 | 49 | P | *Z. caespitosa* | 30008 | 30056 | *Z. caespitosa* | 40142 | 40190 | -3 | 6.53E-14 | *_* | *_* |
| R811 | 49 | P | *Z. caespitosa* | 15972 | 16020 | *Z. caespitosa* | 33018 | 33066 | -3 | 6.53E-14 | *orf284a* | *_* |
| R812 | 49 | P | *Z. caespitosa* | 7605 | 7653 | *Z. caespitosa* | 33018 | 33066 | -3 | 6.53E-14 | *_* | *_* |
| R813 | 48 | F | *Z. caespitosa* | 139859 | 139906 | *Z. caespitosa* | 139892 | 139939 | -1 | 7.56E-17 | *matR* | *matR* |
| R814 | 48 | F | *Z. caespitosa* | 162893 | 162940 | *Z. caespitosa* | 188096 | 188143 | 0 | 5.25E-19 | *_* | *_* |
| R815 | 48 | F | *Z. caespitosa* | 162893 | 162940 | *Z. caespitosa* | 179922 | 179969 | 0 | 5.25E-19 | *_* | *_* |
| R816 | 48 | F | *Z. caespitosa* | 72211 | 72258 | *Z. caespitosa* | 101709 | 101756 | 0 | 5.25E-19 | *_* | *_* |
| R817 | 48 | F | *Z. caespitosa* | 131563 | 131610 | *Z. caespitosa* | 182636 | 182683 | -2 | 5.33E-15 | *orf131b* | *orf117c* |
| R818 | 48 | F | *Z. caespitosa* | 52802 | 52849 | *Z. caespitosa* | 156587 | 156634 | 0 | 5.25E-19 | *orf172a* | *_* |
| R819 | 48 | F | *Z. caespitosa* | 57585 | 57632 | *Z. caespitosa* | 156587 | 156634 | 0 | 5.25E-19 | *orf117a* | *_* |
| R820 | 48 | F | *Z. caespitosa* | 37924 | 37971 | *Z. caespitosa* | 135138 | 135185 | 0 | 5.25E-19 | *_* | *_* |
| R821 | 48 | F | *Z. caespitosa* | 108806 | 108853 | *Z. caespitosa* | 135138 | 135185 | 0 | 5.25E-19 | *orf161a* | *_* |
| R822 | 48 | F | *Z. caespitosa* | 52670 | 52717 | *Z. caespitosa* | 135138 | 135185 | 0 | 5.25E-19 | *orf172a* | *_* |
| R823 | 48 | F | *Z. caespitosa* | 52939 | 52986 | *Z. caespitosa* | 135138 | 135185 | 0 | 5.25E-19 | *orf172a* | *_* |
| R824 | 48 | F | *Z. caespitosa* | 572 | 619 | *Z. caespitosa* | 1233 | 1280 | -2 | 5.33E-15 | *_* | *_* |
| R825 | 48 | F | *Z. caespitosa* | 572 | 619 | *Z. caespitosa* | 43181 | 43228 | -2 | 5.33E-15 | *_* | *_* |
| R826 | 48 | F | *Z. caespitosa* | 1233 | 1280 | *Z. caespitosa* | 42521 | 42568 | -2 | 5.33E-15 | *_* | *_* |
| R827 | 48 | F | *Z. caespitosa* | 42521 | 42568 | *Z. caespitosa* | 43181 | 43228 | -2 | 5.33E-15 | *_* | *_* |
| R828 | 48 | F | *Z. caespitosa* | 125317 | 125364 | *Z. caespitosa* | 159890 | 159937 | -3 | 2.45E-13 | *_* | *rrn26* |
| R829 | 48 | F | *Z. caespitosa* | 52801 | 52848 | *Z. caespitosa* | 156616 | 156663 | 0 | 5.25E-19 | *orf172a* | *_* |
| R830 | 48 | F | *Z. caespitosa* | 57584 | 57631 | *Z. caespitosa* | 156616 | 156663 | 0 | 5.25E-19 | *orf117a* | *_* |
| R831 | 48 | P | *Z. caespitosa* | 135138 | 135185 | *Z. caespitosa* | 188096 | 188143 | 0 | 5.25E-19 | *_* | *_* |
| R832 | 48 | P | *Z. caespitosa* | 135138 | 135185 | *Z. caespitosa* | 179922 | 179969 | 0 | 5.25E-19 | *_* | *_* |
| R833 | 48 | P | *Z. caespitosa* | 110379 | 110426 | *Z. caespitosa* | 173379 | 173426 | 0 | 5.25E-19 | *orf165a* | *_* |
| R834 | 48 | P | *Z. caespitosa* | 52939 | 52986 | *Z. caespitosa* | 162893 | 162940 | 0 | 5.25E-19 | *orf172a* | *_* |
| R835 | 48 | P | *Z. caespitosa* | 52670 | 52717 | *Z. caespitosa* | 162893 | 162940 | 0 | 5.25E-19 | *orf172a* | *_* |
| R836 | 48 | P | *Z. caespitosa* | 108806 | 108853 | *Z. caespitosa* | 162893 | 162940 | 0 | 5.25E-19 | *orf161a* | *_* |
| R837 | 48 | P | *Z. caespitosa* | 37924 | 37971 | *Z. caespitosa* | 162893 | 162940 | 0 | 5.25E-19 | *_* | *_* |
| R838 | 48 | P | *Z. caespitosa* | 156616 | 156663 | *Z. caespitosa* | 162777 | 162824 | 0 | 5.25E-19 | *_* | *_* |
| R839 | 48 | P | *Z. caespitosa* | 16144 | 16191 | *Z. caespitosa* | 156616 | 156663 | 0 | 5.25E-19 | *orf284a* | *_* |
| R840 | 48 | P | *Z. caespitosa* | 69916 | 69963 | *Z. caespitosa* | 156616 | 156663 | 0 | 5.25E-19 | *_* | *_* |
| R841 | 48 | P | *Z. caespitosa* | 69915 | 69962 | *Z. caespitosa* | 156587 | 156634 | 0 | 5.25E-19 | *_* | *_* |
| R842 | 48 | P | *Z. caespitosa* | 74673 | 74720 | *Z. caespitosa* | 156586 | 156633 | -3 | 2.45E-13 | *_* | *_* |
| R843 | 48 | P | *Z. caespitosa* | 15950 | 15997 | *Z. caespitosa* | 136806 | 136853 | -1 | 7.56E-17 | *orf284a* | *_* |
| R844 | 48 | P | *Z. caespitosa* | 7583 | 7630 | *Z. caespitosa* | 136806 | 136853 | -1 | 7.56E-17 | *_* | *_* |
| R845 | 48 | P | *Z. caespitosa* | 53153 | 53200 | *Z. caespitosa* | 101709 | 101756 | 0 | 5.25E-19 | *_* | *_* |
| R846 | 48 | P | *Z. caespitosa* | 84263 | 84310 | *Z. caespitosa* | 101709 | 101756 | 0 | 5.25E-19 | *_* | *_* |
| R847 | 48 | P | *Z. caespitosa* | 75912 | 75959 | *Z. caespitosa* | 79452 | 79499 | 0 | 5.25E-19 | *_* | *_* |
| R848 | 48 | P | *Z. caespitosa* | 15855 | 15902 | *Z. caespitosa* | 46140 | 46187 | 0 | 5.25E-19 | *_* | *_* |
| R849 | 48 | P | *Z. caespitosa* | 15855 | 15902 | *Z. caespitosa* | 43536 | 43583 | 0 | 5.25E-19 | *_* | *_* |
| R850 | 47 | F | *Z. caespitosa* | 46110 | 46156 | *Z. caespitosa* | 177685 | 177731 | -1 | 2.96E-16 | *_* | *_* |
| R851 | 47 | F | *Z. caespitosa* | 46110 | 46156 | *Z. caespitosa* | 180738 | 180784 | -1 | 2.96E-16 | *_* | *_* |
| R852 | 47 | F | *Z. caespitosa* | 4193 | 4239 | *Z. caespitosa* | 46110 | 46156 | -1 | 2.96E-16 | *_* | *_* |
| R853 | 47 | F | *Z. caespitosa* | 46110 | 46156 | *Z. caespitosa* | 83647 | 83693 | -1 | 2.96E-16 | *_* | *_* |
| R854 | 47 | F | *Z. caespitosa* | 4282 | 4328 | *Z. caespitosa* | 46110 | 46156 | -1 | 2.96E-16 | *_* | *_* |
| R855 | 47 | F | *Z. caespitosa* | 46110 | 46156 | *Z. caespitosa* | 83736 | 83782 | -1 | 2.96E-16 | *_* | *_* |
| R856 | 47 | F | *Z. caespitosa* | 4371 | 4417 | *Z. caespitosa* | 46110 | 46156 | -1 | 2.96E-16 | *_* | *_* |
| R857 | 47 | F | *Z. caespitosa* | 46110 | 46156 | *Z. caespitosa* | 177804 | 177850 | -1 | 2.96E-16 | *_* | *_* |
| R858 | 47 | F | *Z. caespitosa* | 46110 | 46156 | *Z. caespitosa* | 83825 | 83871 | -1 | 2.96E-16 | *_* | *_* |
| R859 | 47 | F | *Z. caespitosa* | 4460 | 4506 | *Z. caespitosa* | 46110 | 46156 | -1 | 2.96E-16 | *_* | *_* |
| R860 | 47 | F | *Z. caespitosa* | 72208 | 72254 | *Z. caespitosa* | 162534 | 162580 | -3 | 9.19E-13 | *_* | *_* |
| R861 | 47 | F | *Z. caespitosa* | 162534 | 162580 | *Z. caespitosa* | 188093 | 188139 | -3 | 9.19E-13 | *_* | *_* |
| R862 | 47 | F | *Z. caespitosa* | 162534 | 162580 | *Z. caespitosa* | 179919 | 179965 | -3 | 9.19E-13 | *_* | *_* |
| R863 | 47 | F | *Z. caespitosa* | 15622 | 15668 | *Z. caespitosa* | 16115 | 16161 | 0 | 2.10E-18 | *_* | *orf284a* |
| R864 | 47 | F | *Z. caespitosa* | 15622 | 15668 | *Z. caespitosa* | 162748 | 162794 | 0 | 2.10E-18 | *_* | *_* |
| R865 | 47 | F | *Z. caespitosa* | 32922 | 32968 | *Z. caespitosa* | 36150 | 36196 | 0 | 2.10E-18 | *_* | *_* |
| R866 | 47 | F | *Z. caespitosa* | 14639 | 14685 | *Z. caespitosa* | 25073 | 25119 | -3 | 9.19E-13 | *orf113a;orf295a;cox2* | *orf340a* |
| R867 | 47 | F | *Z. caespitosa* | 14639 | 14685 | *Z. caespitosa* | 95803 | 95849 | -3 | 9.19E-13 | *orf113a;orf295a;cox2* | *orf540a-2* |
| R868 | 47 | F | *Z. caespitosa* | 55265 | 55311 | *Z. caespitosa* | 156455 | 156501 | -2 | 2.04E-14 | *_* | *_* |
| R869 | 47 | F | *Z. caespitosa* | 15972 | 16018 | *Z. caespitosa* | 118817 | 118863 | -3 | 9.19E-13 | *orf284a* | *_* |
| R870 | 47 | F | *Z. caespitosa* | 7605 | 7651 | *Z. caespitosa* | 118817 | 118863 | -3 | 9.19E-13 | *_* | *_* |
| R871 | 47 | F | *Z. caespitosa* | 69916 | 69962 | *Z. caespitosa* | 87366 | 87412 | -3 | 9.19E-13 | *_* | *orf122a* |
| R872 | 47 | F | *Z. caespitosa* | 16144 | 16190 | *Z. caespitosa* | 87366 | 87412 | -3 | 9.19E-13 | *orf284a* | *orf122a* |
| R873 | 47 | F | *Z. caespitosa* | 87366 | 87412 | *Z. caespitosa* | 162777 | 162823 | -3 | 9.19E-13 | *orf122a* | *_* |
| R874 | 47 | F | *Z. caespitosa* | 69916 | 69962 | *Z. caespitosa* | 74673 | 74719 | -3 | 9.19E-13 | *_* | *_* |
| R875 | 47 | F | *Z. caespitosa* | 16144 | 16190 | *Z. caespitosa* | 74673 | 74719 | -3 | 9.19E-13 | *orf284a* | *_* |
| R876 | 47 | F | *Z. caespitosa* | 74673 | 74719 | *Z. caespitosa* | 162777 | 162823 | -3 | 9.19E-13 | *_* | *_* |
| R877 | 47 | F | *Z. caespitosa* | 37843 | 37889 | *Z. caespitosa* | 148853 | 148899 | -3 | 9.19E-13 | *_* | *_* |
| R878 | 47 | P | *Z. caespitosa* | 53157 | 53203 | *Z. caespitosa* | 162534 | 162580 | -3 | 9.19E-13 | *_* | *_* |
| R879 | 47 | P | *Z. caespitosa* | 52943 | 52989 | *Z. caespitosa* | 162534 | 162580 | -3 | 9.19E-13 | *orf172a* | *_* |
| R880 | 47 | P | *Z. caespitosa* | 52674 | 52720 | *Z. caespitosa* | 162534 | 162580 | -3 | 9.19E-13 | *orf172a* | *_* |
| R881 | 47 | P | *Z. caespitosa* | 84267 | 84313 | *Z. caespitosa* | 162534 | 162580 | -3 | 9.19E-13 | *_* | *_* |
| R882 | 47 | P | *Z. caespitosa* | 108810 | 108856 | *Z. caespitosa* | 162534 | 162580 | -3 | 9.19E-13 | *orf161a* | *_* |
| R883 | 47 | P | *Z. caespitosa* | 37928 | 37974 | *Z. caespitosa* | 162534 | 162580 | -3 | 9.19E-13 | *_* | *_* |
| R884 | 47 | P | *Z. caespitosa* | 74673 | 74719 | *Z. caespitosa* | 156617 | 156663 | -3 | 9.19E-13 | *_* | *_* |
| R885 | 47 | P | *Z. caespitosa* | 87366 | 87412 | *Z. caespitosa* | 156617 | 156663 | -3 | 9.19E-13 | *orf122a* | *_* |
| R886 | 47 | P | *Z. caespitosa* | 14639 | 14685 | *Z. caespitosa* | 122187 | 122233 | -3 | 9.19E-13 | *orf113a;orf295a;cox2* | *nad5* |
| R887 | 47 | P | *Z. caespitosa* | 14639 | 14685 | *Z. caespitosa* | 115906 | 115952 | -3 | 9.19E-13 | *orf113a;orf295a;cox2* | *orf540a-1* |
| R888 | 47 | P | *Z. caespitosa* | 57585 | 57631 | *Z. caespitosa* | 87366 | 87412 | -3 | 9.19E-13 | *orf117a* | *orf122a* |
| R889 | 47 | P | *Z. caespitosa* | 52802 | 52848 | *Z. caespitosa* | 87366 | 87412 | -3 | 9.19E-13 | *orf172a* | *orf122a* |
| R890 | 47 | P | *Z. caespitosa* | 57744 | 57790 | *Z. caespitosa* | 79675 | 79721 | -1 | 2.96E-16 | *orf117a* | *_* |
| R891 | 47 | P | *Z. caespitosa* | 57585 | 57631 | *Z. caespitosa* | 74673 | 74719 | -3 | 9.19E-13 | *orf117a* | *_* |
| R892 | 47 | P | *Z. caespitosa* | 52802 | 52848 | *Z. caespitosa* | 74673 | 74719 | -3 | 9.19E-13 | *orf172a* | *_* |
| R893 | 47 | P | *Z. caespitosa* | 15972 | 16018 | *Z. caespitosa* | 22162 | 22208 | -3 | 9.19E-13 | *orf284a* | *_* |
| R894 | 47 | P | *Z. caespitosa* | 7605 | 7651 | *Z. caespitosa* | 22162 | 22208 | -3 | 9.19E-13 | *_* | *_* |
| R895 | 46 | F | *Z. caespitosa* | 23215 | 23260 | *Z. caespitosa* | 27032 | 27077 | 0 | 8.40E-18 | *_* | *_* |
| R896 | 46 | F | *Z. caespitosa* | 27032 | 27077 | *Z. caespitosa* | 93945 | 93990 | 0 | 8.40E-18 | *_* | *_* |
| R897 | 46 | F | *Z. caespitosa* | 15952 | 15997 | *Z. caespitosa* | 27032 | 27077 | -1 | 1.16E-15 | *orf284a* | *_* |
| R898 | 46 | F | *Z. caespitosa* | 7585 | 7630 | *Z. caespitosa* | 27032 | 27077 | -1 | 1.16E-15 | *_* | *_* |
| R899 | 46 | F | *Z. caespitosa* | 7152 | 7197 | *Z. caespitosa* | 160926 | 160971 | -2 | 7.82E-14 | *_* | *trnfM-CAU* |
| R900 | 46 | F | *Z. caespitosa* | 46081 | 46126 | *Z. caespitosa* | 46111 | 46156 | -1 | 1.16E-15 | *_* | *_* |
| R901 | 46 | F | *Z. caespitosa* | 4135 | 4180 | *Z. caespitosa* | 46111 | 46156 | -1 | 1.16E-15 | *_* | *_* |
| R902 | 46 | F | *Z. caespitosa* | 46111 | 46156 | *Z. caespitosa* | 83589 | 83634 | -1 | 1.16E-15 | *_* | *_* |
| R903 | 46 | F | *Z. caespitosa* | 1559 | 1604 | *Z. caespitosa* | 177686 | 177731 | -1 | 1.16E-15 | *_* | *_* |
| R904 | 46 | F | *Z. caespitosa* | 1559 | 1604 | *Z. caespitosa* | 180739 | 180784 | -1 | 1.16E-15 | *_* | *_* |
| R905 | 46 | F | *Z. caespitosa* | 1559 | 1604 | *Z. caespitosa* | 4194 | 4239 | -1 | 1.16E-15 | *_* | *_* |
| R906 | 46 | F | *Z. caespitosa* | 1559 | 1604 | *Z. caespitosa* | 83648 | 83693 | -1 | 1.16E-15 | *_* | *_* |
| R907 | 46 | F | *Z. caespitosa* | 1559 | 1604 | *Z. caespitosa* | 4283 | 4328 | -1 | 1.16E-15 | *_* | *_* |
| R908 | 46 | F | *Z. caespitosa* | 1559 | 1604 | *Z. caespitosa* | 177716 | 177761 | -1 | 1.16E-15 | *_* | *_* |
| R909 | 46 | F | *Z. caespitosa* | 1559 | 1604 | *Z. caespitosa* | 83737 | 83782 | -1 | 1.16E-15 | *_* | *_* |
| R910 | 46 | F | *Z. caespitosa* | 1559 | 1604 | *Z. caespitosa* | 4372 | 4417 | -1 | 1.16E-15 | *_* | *_* |
| R911 | 46 | F | *Z. caespitosa* | 1559 | 1604 | *Z. caespitosa* | 177805 | 177850 | -1 | 1.16E-15 | *_* | *_* |
| R912 | 46 | F | *Z. caespitosa* | 1559 | 1604 | *Z. caespitosa* | 83826 | 83871 | -1 | 1.16E-15 | *_* | *_* |
| R913 | 46 | F | *Z. caespitosa* | 1559 | 1604 | *Z. caespitosa* | 4461 | 4506 | -1 | 1.16E-15 | *_* | *_* |
| R914 | 46 | F | *Z. caespitosa* | 1559 | 1604 | *Z. caespitosa* | 180769 | 180814 | -1 | 1.16E-15 | *_* | *_* |
| R915 | 46 | F | *Z. caespitosa* | 1559 | 1604 | *Z. caespitosa* | 4224 | 4269 | -1 | 1.16E-15 | *_* | *_* |
| R916 | 46 | F | *Z. caespitosa* | 1559 | 1604 | *Z. caespitosa* | 83678 | 83723 | -1 | 1.16E-15 | *_* | *_* |
| R917 | 46 | F | *Z. caespitosa* | 1559 | 1604 | *Z. caespitosa* | 4313 | 4358 | -1 | 1.16E-15 | *_* | *_* |
| R918 | 46 | F | *Z. caespitosa* | 1559 | 1604 | *Z. caespitosa* | 177746 | 177791 | -1 | 1.16E-15 | *_* | *_* |
| R919 | 46 | F | *Z. caespitosa* | 1559 | 1604 | *Z. caespitosa* | 83767 | 83812 | -1 | 1.16E-15 | *_* | *_* |
| R920 | 46 | F | *Z. caespitosa* | 1559 | 1604 | *Z. caespitosa* | 4402 | 4447 | -1 | 1.16E-15 | *_* | *_* |
| R921 | 46 | F | *Z. caespitosa* | 43507 | 43552 | *Z. caespitosa* | 177686 | 177731 | -1 | 1.16E-15 | *_* | *_* |
| R922 | 46 | F | *Z. caespitosa* | 43507 | 43552 | *Z. caespitosa* | 180739 | 180784 | -1 | 1.16E-15 | *_* | *_* |
| R923 | 46 | F | *Z. caespitosa* | 4194 | 4239 | *Z. caespitosa* | 43507 | 43552 | -1 | 1.16E-15 | *_* | *_* |
| R924 | 46 | F | *Z. caespitosa* | 43507 | 43552 | *Z. caespitosa* | 83648 | 83693 | -1 | 1.16E-15 | *_* | *_* |
| R925 | 46 | F | *Z. caespitosa* | 4283 | 4328 | *Z. caespitosa* | 43507 | 43552 | -1 | 1.16E-15 | *_* | *_* |
| R926 | 46 | F | *Z. caespitosa* | 43507 | 43552 | *Z. caespitosa* | 177716 | 177761 | -1 | 1.16E-15 | *_* | *_* |
| R927 | 46 | F | *Z. caespitosa* | 43507 | 43552 | *Z. caespitosa* | 83737 | 83782 | -1 | 1.16E-15 | *_* | *_* |
| R928 | 46 | F | *Z. caespitosa* | 4372 | 4417 | *Z. caespitosa* | 43507 | 43552 | -1 | 1.16E-15 | *_* | *_* |
| R929 | 46 | F | *Z. caespitosa* | 43507 | 43552 | *Z. caespitosa* | 177805 | 177850 | -1 | 1.16E-15 | *_* | *_* |
| R930 | 46 | F | *Z. caespitosa* | 43507 | 43552 | *Z. caespitosa* | 83826 | 83871 | -1 | 1.16E-15 | *_* | *_* |
| R931 | 46 | F | *Z. caespitosa* | 4461 | 4506 | *Z. caespitosa* | 43507 | 43552 | -1 | 1.16E-15 | *_* | *_* |
| R932 | 46 | F | *Z. caespitosa* | 43507 | 43552 | *Z. caespitosa* | 180769 | 180814 | -1 | 1.16E-15 | *_* | *_* |
| R933 | 46 | F | *Z. caespitosa* | 4224 | 4269 | *Z. caespitosa* | 43507 | 43552 | -1 | 1.16E-15 | *_* | *_* |
| R934 | 46 | F | *Z. caespitosa* | 43507 | 43552 | *Z. caespitosa* | 83678 | 83723 | -1 | 1.16E-15 | *_* | *_* |
| R935 | 46 | F | *Z. caespitosa* | 4313 | 4358 | *Z. caespitosa* | 43507 | 43552 | -1 | 1.16E-15 | *_* | *_* |
| R936 | 46 | F | *Z. caespitosa* | 43507 | 43552 | *Z. caespitosa* | 177746 | 177791 | -1 | 1.16E-15 | *_* | *_* |
| R937 | 46 | F | *Z. caespitosa* | 43507 | 43552 | *Z. caespitosa* | 83767 | 83812 | -1 | 1.16E-15 | *_* | *_* |
| R938 | 46 | F | *Z. caespitosa* | 4402 | 4447 | *Z. caespitosa* | 43507 | 43552 | -1 | 1.16E-15 | *_* | *_* |
| R939 | 46 | F | *Z. caespitosa* | 72261 | 72306 | *Z. caespitosa* | 162999 | 163044 | -3 | 3.44E-12 | *_* | *_* |
| R940 | 46 | F | *Z. caespitosa* | 14560 | 14605 | *Z. caespitosa* | 184965 | 185010 | -1 | 1.16E-15 | *orf113a* | *_* |
| R941 | 46 | F | *Z. caespitosa* | 16064 | 16109 | *Z. caespitosa* | 184965 | 185010 | -1 | 1.16E-15 | *orf284a* | *_* |
| R942 | 46 | F | *Z. caespitosa* | 69381 | 69426 | *Z. caespitosa* | 127196 | 127241 | -3 | 3.44E-12 | *orf118b* | *orf242a* |
| R943 | 46 | F | *Z. caespitosa* | 53051 | 53096 | *Z. caespitosa* | 175069 | 175114 | 0 | 8.40E-18 | *_* | *_* |
| R944 | 46 | F | *Z. caespitosa* | 33021 | 33066 | *Z. caespitosa* | 175069 | 175114 | 0 | 8.40E-18 | *_* | *_* |
| R945 | 46 | F | *Z. caespitosa* | 22163 | 22208 | *Z. caespitosa* | 175069 | 175114 | 0 | 8.40E-18 | *_* | *_* |
| R946 | 46 | F | *Z. caespitosa* | 15972 | 16017 | *Z. caespitosa* | 16065 | 16110 | -3 | 3.44E-12 | *orf284a* | *orf284a* |
| R947 | 46 | F | *Z. caespitosa* | 7605 | 7650 | *Z. caespitosa* | 16065 | 16110 | -3 | 3.44E-12 | *_* | *orf284a* |
| R948 | 46 | F | *Z. caespitosa* | 15972 | 16017 | *Z. caespitosa* | 83366 | 83411 | -3 | 3.44E-12 | *orf284a* | *_* |
| R949 | 46 | F | *Z. caespitosa* | 7605 | 7650 | *Z. caespitosa* | 83366 | 83411 | -3 | 3.44E-12 | *_* | *_* |
| R950 | 46 | F | *Z. caespitosa* | 14561 | 14606 | *Z. caespitosa* | 149191 | 149236 | -2 | 7.82E-14 | *orf113a* | *_* |
| R951 | 46 | F | *Z. caespitosa* | 118817 | 118862 | *Z. caespitosa* | 149191 | 149236 | -2 | 7.82E-14 | *_* | *_* |
| R952 | 46 | F | *Z. caespitosa* | 83366 | 83411 | *Z. caespitosa* | 149191 | 149236 | -2 | 7.82E-14 | *_* | *_* |
| R953 | 46 | F | *Z. caespitosa* | 137001 | 137046 | *Z. caespitosa* | 137063 | 137108 | 0 | 8.40E-18 | *_* | *_* |
| R954 | 46 | F | *Z. caespitosa* | 33193 | 33238 | *Z. caespitosa* | 35321 | 35366 | 0 | 8.40E-18 | *_* | *_* |
| R955 | 46 | F | *Z. caespitosa* | 108758 | 108803 | *Z. caespitosa* | 186462 | 186507 | -3 | 3.44E-12 | *orf161a* | *orf100b* |
| R956 | 46 | F | *Z. caespitosa* | 164683 | 164728 | *Z. caespitosa* | 164745 | 164790 | 0 | 8.40E-18 | *_* | *_* |
| R957 | 46 | F | *Z. caespitosa* | 135157 | 135202 | *Z. caespitosa* | 177850 | 177895 | -2 | 7.82E-14 | *_* | *_* |
| R958 | 46 | F | *Z. caespitosa* | 83871 | 83916 | *Z. caespitosa* | 135157 | 135202 | -2 | 7.82E-14 | *_* | *_* |
| R959 | 46 | F | *Z. caespitosa* | 9372 | 9417 | *Z. caespitosa* | 10629 | 10674 | 0 | 8.40E-18 | *orf397a* | *_* |
| R960 | 46 | F | *Z. caespitosa* | 9308 | 9353 | *Z. caespitosa* | 10629 | 10674 | 0 | 8.40E-18 | *orf397a* | *_* |
| R961 | 46 | F | *Z. caespitosa* | 9244 | 9289 | *Z. caespitosa* | 10629 | 10674 | 0 | 8.40E-18 | *orf397a* | *_* |
| R962 | 46 | F | *Z. caespitosa* | 9180 | 9225 | *Z. caespitosa* | 10629 | 10674 | 0 | 8.40E-18 | *orf397a* | *_* |
| R963 | 46 | F | *Z. caespitosa* | 9436 | 9481 | *Z. caespitosa* | 10629 | 10674 | 0 | 8.40E-18 | *orf397a* | *_* |
| R964 | 46 | F | *Z. caespitosa* | 9500 | 9545 | *Z. caespitosa* | 10629 | 10674 | 0 | 8.40E-18 | *orf397a* | *_* |
| R965 | 46 | F | *Z. caespitosa* | 9564 | 9609 | *Z. caespitosa* | 10629 | 10674 | 0 | 8.40E-18 | *orf397a* | *_* |
| R966 | 46 | F | *Z. caespitosa* | 9692 | 9737 | *Z. caespitosa* | 10629 | 10674 | 0 | 8.40E-18 | *orf397a* | *_* |
| R967 | 46 | F | *Z. caespitosa* | 9756 | 9801 | *Z. caespitosa* | 10629 | 10674 | 0 | 8.40E-18 | *orf397a* | *_* |
| R968 | 46 | F | *Z. caespitosa* | 9628 | 9673 | *Z. caespitosa* | 10629 | 10674 | -2 | 7.82E-14 | *orf397a* | *_* |
| R969 | 46 | F | *Z. caespitosa* | 16218 | 16263 | *Z. caespitosa* | 16581 | 16626 | -1 | 1.16E-15 | *orf284a* | *orf284a* |
| R970 | 46 | P | *Z. caespitosa* | 162999 | 163044 | *Z. caespitosa* | 186462 | 186507 | -3 | 3.44E-12 | *_* | *orf100b* |
| R971 | 46 | P | *Z. caespitosa* | 164651 | 164696 | *Z. caespitosa* | 180807 | 180852 | -1 | 1.16E-15 | *_* | *_* |
| R972 | 46 | P | *Z. caespitosa* | 162876 | 162921 | *Z. caespitosa* | 177850 | 177895 | -2 | 7.82E-14 | *_* | *_* |
| R973 | 46 | P | *Z. caespitosa* | 16065 | 16110 | *Z. caespitosa* | 175069 | 175114 | 0 | 8.40E-18 | *orf284a* | *_* |
| R974 | 46 | P | *Z. caespitosa* | 118817 | 118862 | *Z. caespitosa* | 175069 | 175114 | 0 | 8.40E-18 | *_* | *_* |
| R975 | 46 | P | *Z. caespitosa* | 14561 | 14606 | *Z. caespitosa* | 175069 | 175114 | 0 | 8.40E-18 | *orf113a* | *_* |
| R976 | 46 | P | *Z. caespitosa* | 137063 | 137108 | *Z. caespitosa* | 164745 | 164790 | 0 | 8.40E-18 | *_* | *_* |
| R977 | 46 | P | *Z. caespitosa* | 137001 | 137046 | *Z. caespitosa* | 164683 | 164728 | 0 | 8.40E-18 | *_* | *_* |
| R978 | 46 | P | *Z. caespitosa* | 83871 | 83916 | *Z. caespitosa* | 162876 | 162921 | -2 | 7.82E-14 | *_* | *_* |
| R979 | 46 | P | *Z. caespitosa* | 33021 | 33066 | *Z. caespitosa* | 149191 | 149236 | -2 | 7.82E-14 | *_* | *_* |
| R980 | 46 | P | *Z. caespitosa* | 22163 | 22208 | *Z. caespitosa* | 149191 | 149236 | -2 | 7.82E-14 | *_* | *_* |
| R981 | 46 | P | *Z. caespitosa* | 27032 | 27077 | *Z. caespitosa* | 117765 | 117810 | 0 | 8.40E-18 | *_* | *_* |
| R982 | 46 | P | *Z. caespitosa* | 72261 | 72306 | *Z. caespitosa* | 108758 | 108803 | -3 | 3.44E-12 | *_* | *orf161a* |
| R983 | 46 | P | *Z. caespitosa* | 51770 | 51815 | *Z. caespitosa* | 79504 | 79549 | -2 | 7.82E-14 | *orf106b;ccmFn* | *_* |
| R984 | 46 | P | *Z. caespitosa* | 15972 | 16017 | *Z. caespitosa* | 53051 | 53096 | -3 | 3.44E-12 | *orf284a* | *_* |
| R985 | 46 | P | *Z. caespitosa* | 7605 | 7650 | *Z. caespitosa* | 53051 | 53096 | -3 | 3.44E-12 | *_* | *_* |
| R986 | 46 | P | *Z. caespitosa* | 7519 | 7564 | *Z. caespitosa* | 33143 | 33188 | -1 | 1.16E-15 | *_* | *_* |
| R987 | 46 | P | *Z. caespitosa* | 27066 | 27111 | *Z. caespitosa* | 33109 | 33154 | -1 | 1.16E-15 | *_* | *_* |
| R988 | 45 | F | *Z. caespitosa* | 10080 | 10124 | *Z. caespitosa* | 10206 | 10250 | -1 | 4.53E-15 | *orf397a* | *_* |
| R989 | 45 | F | *Z. caespitosa* | 162536 | 162580 | *Z. caespitosa* | 162892 | 162936 | -2 | 2.99E-13 | *_* | *_* |
| R990 | 45 | F | *Z. caespitosa* | 101708 | 101752 | *Z. caespitosa* | 162536 | 162580 | -2 | 2.99E-13 | *_* | *_* |
| R991 | 45 | F | *Z. caespitosa* | 28766 | 28810 | *Z. caespitosa* | 113344 | 113388 | -3 | 1.29E-11 | *_* | *_* |
| R992 | 45 | F | *Z. caespitosa* | 177657 | 177701 | *Z. caespitosa* | 177835 | 177879 | -1 | 4.53E-15 | *_* | *_* |
| R993 | 45 | F | *Z. caespitosa* | 177835 | 177879 | *Z. caespitosa* | 180710 | 180754 | -1 | 4.53E-15 | *_* | *_* |
| R994 | 45 | F | *Z. caespitosa* | 83856 | 83900 | *Z. caespitosa* | 177657 | 177701 | -1 | 4.53E-15 | *_* | *_* |
| R995 | 45 | F | *Z. caespitosa* | 83856 | 83900 | *Z. caespitosa* | 180710 | 180754 | -1 | 4.53E-15 | *_* | *_* |
| R996 | 45 | F | *Z. caespitosa* | 4491 | 4535 | *Z. caespitosa* | 177657 | 177701 | -1 | 4.53E-15 | *_* | *_* |
| R997 | 45 | F | *Z. caespitosa* | 4491 | 4535 | *Z. caespitosa* | 180710 | 180754 | -1 | 4.53E-15 | *_* | *_* |
| R998 | 45 | F | *Z. caespitosa* | 177657 | 177701 | *Z. caespitosa* | 180799 | 180843 | -1 | 4.53E-15 | *_* | *_* |
| R999 | 45 | F | *Z. caespitosa* | 180710 | 180754 | *Z. caespitosa* | 180799 | 180843 | -1 | 4.53E-15 | *_* | *_* |
| R1000 | 45 | F | *Z. caespitosa* | 8039 | 8083 | *Z. caespitosa* | 14270 | 14314 | -3 | 1.29E-11 | *_* | *orf113a* |
| R1001 | 45 | F | *Z. caespitosa* | 68543 | 68587 | *Z. caespitosa* | 181563 | 181607 | 0 | 3.36E-17 | *_* | *orf103d* |
| R1002 | 45 | F | *Z. caespitosa* | 68543 | 68587 | *Z. caespitosa* | 136858 | 136902 | 0 | 3.36E-17 | *_* | *_* |
| R1003 | 45 | F | *Z. caespitosa* | 68543 | 68587 | *Z. caespitosa* | 177898 | 177942 | 0 | 3.36E-17 | *_* | *_* |
| R1004 | 45 | F | *Z. caespitosa* | 68543 | 68587 | *Z. caespitosa* | 83919 | 83963 | 0 | 3.36E-17 | *_* | *_* |
| R1005 | 45 | F | *Z. caespitosa* | 51586 | 51630 | *Z. caespitosa* | 51725 | 51769 | -1 | 4.53E-15 | *orf106b;ccmFn* | *orf106b;ccmFn* |
| R1006 | 45 | F | *Z. caespitosa* | 149191 | 149235 | *Z. caespitosa* | 184966 | 185010 | -3 | 1.29E-11 | *_* | *_* |
| R1007 | 45 | F | *Z. caespitosa* | 87369 | 87413 | *Z. caespitosa* | 140105 | 140149 | -1 | 4.53E-15 | *orf122a* | *matR* |
| R1008 | 45 | F | *Z. caespitosa* | 98369 | 98413 | *Z. caespitosa* | 131300 | 131344 | -3 | 1.29E-11 | *_* | *orf131b* |
| R1009 | 45 | F | *Z. caespitosa* | 37907 | 37951 | *Z. caespitosa* | 83520 | 83564 | 0 | 3.36E-17 | *_* | *_* |
| R1010 | 45 | F | *Z. caespitosa* | 83520 | 83564 | *Z. caespitosa* | 108789 | 108833 | 0 | 3.36E-17 | *_* | *orf161a* |
| R1011 | 45 | F | *Z. caespitosa* | 37907 | 37951 | *Z. caespitosa* | 177588 | 177632 | 0 | 3.36E-17 | *_* | *_* |
| R1012 | 45 | F | *Z. caespitosa* | 108789 | 108833 | *Z. caespitosa* | 177588 | 177632 | 0 | 3.36E-17 | *orf161a* | *_* |
| R1013 | 45 | F | *Z. caespitosa* | 9886 | 9930 | *Z. caespitosa* | 10201 | 10245 | -3 | 1.29E-11 | *orf397a* | *orf397a* |
| R1014 | 45 | P | *Z. caespitosa* | 175070 | 175114 | *Z. caespitosa* | 184966 | 185010 | -1 | 4.53E-15 | *_* | *_* |
| R1015 | 45 | P | *Z. caespitosa* | 135142 | 135186 | *Z. caespitosa* | 162536 | 162580 | -2 | 2.99E-13 | *_* | *_* |
| R1016 | 45 | P | *Z. caespitosa* | 85126 | 85170 | *Z. caespitosa* | 142103 | 142147 | -3 | 1.29E-11 | *orf100a* | *orf101b* |
| R1017 | 45 | P | *Z. caespitosa* | 113343 | 113387 | *Z. caespitosa* | 131300 | 131344 | -3 | 1.29E-11 | *_* | *orf131b* |
| R1018 | 45 | P | *Z. caespitosa* | 28766 | 28810 | *Z. caespitosa* | 98368 | 98412 | -3 | 1.29E-11 | *_* | *_* |
| R1019 | 45 | P | *Z. caespitosa* | 65070 | 65114 | *Z. caespitosa* | 89733 | 89777 | -1 | 4.53E-15 | *_* | *orf103a* |
| R1020 | 45 | P | *Z. caespitosa* | 51815 | 51859 | *Z. caespitosa* | 87411 | 87455 | -1 | 4.53E-15 | *orf106b;ccmFn* | *orf122a* |
| R1021 | 45 | P | *Z. caespitosa* | 55135 | 55179 | *Z. caespitosa* | 85243 | 85287 | 0 | 3.36E-17 | *_* | *orf100a* |
| R1022 | 45 | P | *Z. caespitosa* | 34260 | 34304 | *Z. caespitosa* | 85243 | 85287 | 0 | 3.36E-17 | *_* | *orf100a* |
| R1023 | 45 | P | *Z. caespitosa* | 55135 | 55179 | *Z. caespitosa* | 77933 | 77977 | 0 | 3.36E-17 | *_* | *_* |
| R1024 | 45 | P | *Z. caespitosa* | 34260 | 34304 | *Z. caespitosa* | 77933 | 77977 | 0 | 3.36E-17 | *_* | *_* |
| R1025 | 45 | P | *Z. caespitosa* | 51668 | 51712 | *Z. caespitosa* | 71945 | 71989 | -1 | 4.53E-15 | *orf106b;ccmFn* | *_* |
| R1026 | 45 | P | *Z. caespitosa* | 16081 | 16125 | *Z. caespitosa* | 53001 | 53045 | 0 | 3.36E-17 | *orf284a* | *_* |
| R1027 | 45 | P | *Z. caespitosa* | 15887 | 15931 | *Z. caespitosa* | 33143 | 33187 | -1 | 4.53E-15 | *_* | *_* |
| R1028 | 44 | F | *Z. caespitosa* | 69724 | 69767 | *Z. caespitosa* | 127528 | 127571 | -1 | 1.77E-14 | *_* | *orf149b* |
| R1029 | 44 | F | *Z. caespitosa* | 138796 | 138839 | *Z. caespitosa* | 138882 | 138925 | 0 | 1.34E-16 | *_* | *_* |
| R1030 | 44 | F | *Z. caespitosa* | 172705 | 172748 | *Z. caespitosa* | 180009 | 180052 | 0 | 1.34E-16 | *orf185a* | *_* |
| R1031 | 44 | F | *Z. caespitosa* | 148872 | 148915 | *Z. caespitosa* | 186435 | 186478 | 0 | 1.34E-16 | *_* | *orf100b* |
| R1032 | 44 | F | *Z. caespitosa* | 57043 | 57086 | *Z. caespitosa* | 74154 | 74197 | -2 | 1.14E-12 | *rrn18;orf152a* | *_* |
| R1033 | 44 | F | *Z. caespitosa* | 15645 | 15688 | *Z. caespitosa* | 162801 | 162844 | 0 | 1.34E-16 | *_* | *_* |
| R1034 | 44 | F | *Z. caespitosa* | 41487 | 41530 | *Z. caespitosa* | 68959 | 69002 | -3 | 4.80E-11 | *orf120b* | *_* |
| R1035 | 44 | F | *Z. caespitosa* | 69817 | 69860 | *Z. caespitosa* | 127621 | 127664 | -1 | 1.77E-14 | *_* | *orf149b* |
| R1036 | 44 | F | *Z. caespitosa* | 40159 | 40202 | *Z. caespitosa* | 126405 | 126448 | -3 | 4.80E-11 | *_* | *_* |
| R1037 | 44 | F | *Z. caespitosa* | 4506 | 4549 | *Z. caespitosa* | 135157 | 135200 | -2 | 1.14E-12 | *_* | *_* |
| R1038 | 44 | F | *Z. caespitosa* | 135911 | 135954 | *Z. caespitosa* | 179984 | 180027 | -3 | 4.80E-11 | *_* | *_* |
| R1039 | 44 | F | *Z. caespitosa* | 34238 | 34281 | *Z. caespitosa* | 34939 | 34982 | 0 | 1.34E-16 | *_* | *_* |
| R1040 | 44 | P | *Z. caespitosa* | 149700 | 149743 | *Z. caespitosa* | 190833 | 190876 | -2 | 1.14E-12 | *_* | *_* |
| R1041 | 44 | P | *Z. caespitosa* | 75749 | 75792 | *Z. caespitosa* | 180009 | 180052 | 0 | 1.34E-16 | *_* | *_* |
| R1042 | 44 | P | *Z. caespitosa* | 148689 | 148732 | *Z. caespitosa* | 180009 | 180052 | -1 | 1.77E-14 | *_* | *_* |
| R1043 | 44 | P | *Z. caespitosa* | 156628 | 156671 | *Z. caespitosa* | 172958 | 173001 | -3 | 4.80E-11 | *_* | *orf185a* |
| R1044 | 44 | P | *Z. caespitosa* | 4506 | 4549 | *Z. caespitosa* | 162878 | 162921 | -2 | 1.14E-12 | *_* | *_* |
| R1045 | 44 | P | *Z. caespitosa* | 48656 | 48699 | *Z. caespitosa* | 156628 | 156671 | -3 | 4.80E-11 | *_* | *_* |
| R1046 | 44 | P | *Z. caespitosa* | 34925 | 34968 | *Z. caespitosa* | 138944 | 138987 | -3 | 4.80E-11 | *_* | *_* |
| R1047 | 44 | P | *Z. caespitosa* | 28805 | 28848 | *Z. caespitosa* | 68818 | 68861 | -3 | 4.80E-11 | *_* | *_* |
| R1048 | 43 | F | *Z. caespitosa* | 56495 | 56537 | *Z. caespitosa* | 73843 | 73885 | -3 | 1.79E-10 | *rrn18* | *_* |
| R1049 | 43 | F | *Z. caespitosa* | 40786 | 40828 | *Z. caespitosa* | 127032 | 127074 | -3 | 1.79E-10 | *orf309a* | *orf242a* |
| R1050 | 43 | F | *Z. caespitosa* | 37750 | 37792 | *Z. caespitosa* | 181647 | 181689 | 0 | 5.37E-16 | *_* | *orf103d* |
| R1051 | 43 | F | *Z. caespitosa* | 37750 | 37792 | *Z. caespitosa* | 75917 | 75959 | 0 | 5.37E-16 | *_* | *_* |
| R1052 | 43 | F | *Z. caespitosa* | 37750 | 37792 | *Z. caespitosa* | 184209 | 184251 | 0 | 5.37E-16 | *_* | *orf130b* |
| R1053 | 43 | F | *Z. caespitosa* | 84308 | 84350 | *Z. caespitosa* | 135171 | 135213 | 0 | 5.37E-16 | *_* | *_* |
| R1054 | 43 | F | *Z. caespitosa* | 25508 | 25550 | *Z. caespitosa* | 176039 | 176081 | 0 | 5.37E-16 | *orf340a* | *_* |
| R1055 | 43 | F | *Z. caespitosa* | 96238 | 96280 | *Z. caespitosa* | 176039 | 176081 | 0 | 5.37E-16 | *orf540a-2* | *_* |
| R1056 | 43 | F | *Z. caespitosa* | 24947 | 24989 | *Z. caespitosa* | 69856 | 69898 | -1 | 6.93E-14 | *_* | *_* |
| R1057 | 43 | F | *Z. caespitosa* | 69856 | 69898 | *Z. caespitosa* | 95677 | 95719 | -1 | 6.93E-14 | *_* | *_* |
| R1058 | 43 | F | *Z. caespitosa* | 139831 | 139873 | *Z. caespitosa* | 139897 | 139939 | 0 | 5.37E-16 | *matR* | *matR* |
| R1059 | 43 | F | *Z. caespitosa* | 101695 | 101737 | *Z. caespitosa* | 164647 | 164689 | -3 | 1.79E-10 | *_* | *_* |
| R1060 | 43 | F | *Z. caespitosa* | 15645 | 15687 | *Z. caespitosa* | 69940 | 69982 | 0 | 5.37E-16 | *_* | *_* |
| R1061 | 43 | F | *Z. caespitosa* | 15645 | 15687 | *Z. caespitosa* | 16168 | 16210 | 0 | 5.37E-16 | *_* | *orf284a* |
| R1062 | 43 | F | *Z. caespitosa* | 27082 | 27124 | *Z. caespitosa* | 77632 | 77674 | -3 | 1.79E-10 | *_* | *_* |
| R1063 | 43 | F | *Z. caespitosa* | 68543 | 68585 | *Z. caespitosa* | 108755 | 108797 | -1 | 6.93E-14 | *_* | *orf161a* |
| R1064 | 43 | F | *Z. caespitosa* | 9913 | 9955 | *Z. caespitosa* | 10039 | 10081 | -3 | 1.79E-10 | *orf397a* | *orf397a* |
| R1065 | 43 | F | *Z. caespitosa* | 34940 | 34982 | *Z. caespitosa* | 55114 | 55156 | 0 | 5.37E-16 | *_* | *_* |
| R1066 | 43 | F | *Z. caespitosa* | 10083 | 10125 | *Z. caespitosa* | 10146 | 10188 | -3 | 1.79E-10 | *orf397a* | *orf397a* |
| R1067 | 43 | F | *Z. caespitosa* | 10020 | 10062 | *Z. caespitosa* | 10083 | 10125 | -3 | 1.79E-10 | *orf397a* | *orf397a* |
| R1068 | 43 | F | *Z. caespitosa* | 36876 | 36918 | *Z. caespitosa* | 85267 | 85309 | 0 | 5.37E-16 | *_* | *orf100a* |
| R1069 | 43 | F | *Z. caespitosa* | 36876 | 36918 | *Z. caespitosa* | 77957 | 77999 | 0 | 5.37E-16 | *_* | *_* |
| R1070 | 43 | F | *Z. caespitosa* | 8155 | 8197 | *Z. caespitosa* | 14386 | 14428 | -3 | 1.79E-10 | *_* | *orf113a* |
| R1071 | 43 | P | *Z. caespitosa* | 138799 | 138841 | *Z. caespitosa* | 180604 | 180646 | 0 | 5.37E-16 | *_* | *_* |
| R1072 | 43 | P | *Z. caespitosa* | 53009 | 53051 | *Z. caespitosa* | 172845 | 172887 | 0 | 5.37E-16 | *_* | *orf185a* |
| R1073 | 43 | P | *Z. caespitosa* | 37750 | 37792 | *Z. caespitosa* | 172538 | 172580 | 0 | 5.37E-16 | *_* | *_* |
| R1074 | 43 | P | *Z. caespitosa* | 68543 | 68585 | *Z. caespitosa* | 163005 | 163047 | -1 | 6.93E-14 | *_* | *_* |
| R1075 | 43 | P | *Z. caespitosa* | 37750 | 37792 | *Z. caespitosa* | 140107 | 140149 | 0 | 5.37E-16 | *_* | *matR* |
| R1076 | 43 | P | *Z. caespitosa* | 85080 | 85122 | *Z. caespitosa* | 136819 | 136861 | -1 | 6.93E-14 | *_* | *_* |
| R1077 | 43 | P | *Z. caespitosa* | 69856 | 69898 | *Z. caespitosa* | 116036 | 116078 | -1 | 6.93E-14 | *_* | *_* |
| R1078 | 43 | P | *Z. caespitosa* | 68543 | 68585 | *Z. caespitosa* | 79663 | 79705 | 0 | 5.37E-16 | *_* | *_* |
| R1079 | 43 | P | *Z. caespitosa* | 37750 | 37792 | *Z. caespitosa* | 77670 | 77712 | 0 | 5.37E-16 | *_* | *_* |
| R1080 | 43 | P | *Z. caespitosa* | 51613 | 51655 | *Z. caespitosa* | 74579 | 74621 | -1 | 6.93E-14 | *orf106b;ccmFn* | *_* |
| R1081 | 43 | P | *Z. caespitosa* | 16631 | 16673 | *Z. caespitosa* | 50014 | 50056 | 0 | 5.37E-16 | *orf284a* | *_* |
| R1082 | 43 | P | *Z. caespitosa* | 34941 | 34983 | *Z. caespitosa* | 48646 | 48688 | 0 | 5.37E-16 | *_* | *_* |
| R1083 | 42 | F | *Z. caespitosa* | 1253 | 1294 | *Z. caespitosa* | 152493 | 152534 | -3 | 6.66E-10 | *_* | *_* |
| R1084 | 42 | F | *Z. caespitosa* | 43201 | 43242 | *Z. caespitosa* | 152493 | 152534 | -3 | 6.66E-10 | *_* | *_* |
| R1085 | 42 | F | *Z. caespitosa* | 16233 | 16274 | *Z. caespitosa* | 87341 | 87382 | -3 | 6.66E-10 | *orf284a* | *orf122a* |
| R1086 | 42 | F | *Z. caespitosa* | 16266 | 16307 | *Z. caespitosa* | 87341 | 87382 | -3 | 6.66E-10 | *orf284a* | *orf122a* |
| R1087 | 42 | F | *Z. caespitosa* | 16299 | 16340 | *Z. caespitosa* | 87341 | 87382 | -3 | 6.66E-10 | *orf284a* | *orf122a* |
| R1088 | 42 | F | *Z. caespitosa* | 16332 | 16373 | *Z. caespitosa* | 87341 | 87382 | -3 | 6.66E-10 | *orf284a* | *orf122a* |
| R1089 | 42 | F | *Z. caespitosa* | 16365 | 16406 | *Z. caespitosa* | 87341 | 87382 | -3 | 6.66E-10 | *orf284a* | *orf122a* |
| R1090 | 42 | F | *Z. caespitosa* | 16398 | 16439 | *Z. caespitosa* | 87341 | 87382 | -3 | 6.66E-10 | *orf284a* | *orf122a* |
| R1091 | 42 | F | *Z. caespitosa* | 16431 | 16472 | *Z. caespitosa* | 87341 | 87382 | -3 | 6.66E-10 | *orf284a* | *orf122a* |
| R1092 | 42 | F | *Z. caespitosa* | 16464 | 16505 | *Z. caespitosa* | 87341 | 87382 | -3 | 6.66E-10 | *orf284a* | *orf122a* |
| R1093 | 42 | F | *Z. caespitosa* | 16497 | 16538 | *Z. caespitosa* | 87341 | 87382 | -3 | 6.66E-10 | *orf284a* | *orf122a* |
| R1094 | 42 | F | *Z. caespitosa* | 16530 | 16571 | *Z. caespitosa* | 87341 | 87382 | -3 | 6.66E-10 | *orf284a* | *orf122a* |
| R1095 | 42 | F | *Z. caespitosa* | 16563 | 16604 | *Z. caespitosa* | 87341 | 87382 | -3 | 6.66E-10 | *orf284a* | *orf122a* |
| R1096 | 42 | F | *Z. caespitosa* | 162880 | 162921 | *Z. caespitosa* | 164648 | 164689 | -3 | 6.66E-10 | *_* | *_* |
| R1097 | 42 | F | *Z. caespitosa* | 135099 | 135140 | *Z. caespitosa* | 186471 | 186512 | -1 | 2.71E-13 | *_* | *orf100b* |
| R1098 | 42 | F | *Z. caespitosa* | 135099 | 135140 | *Z. caespitosa* | 181575 | 181616 | -1 | 2.71E-13 | *_* | *orf103d* |
| R1099 | 42 | F | *Z. caespitosa* | 135099 | 135140 | *Z. caespitosa* | 136870 | 136911 | -1 | 2.71E-13 | *_* | *_* |
| R1100 | 42 | F | *Z. caespitosa* | 135099 | 135140 | *Z. caespitosa* | 177910 | 177951 | -1 | 2.71E-13 | *_* | *_* |
| R1101 | 42 | F | *Z. caespitosa* | 83931 | 83972 | *Z. caespitosa* | 135099 | 135140 | -1 | 2.71E-13 | *_* | *_* |
| R1102 | 42 | F | *Z. caespitosa* | 37874 | 37915 | *Z. caespitosa* | 186460 | 186501 | 0 | 2.15E-15 | *_* | *orf100b* |
| R1103 | 42 | F | *Z. caespitosa* | 37814 | 37855 | *Z. caespitosa* | 53107 | 53148 | -1 | 2.71E-13 | *_* | *_* |
| R1104 | 42 | F | *Z. caespitosa* | 53107 | 53148 | *Z. caespitosa* | 148761 | 148802 | -2 | 1.67E-11 | *_* | *_* |
| R1105 | 42 | F | *Z. caespitosa* | 53107 | 53148 | *Z. caespitosa* | 148824 | 148865 | -2 | 1.67E-11 | *_* | *_* |
| R1106 | 42 | F | *Z. caespitosa* | 68543 | 68584 | *Z. caespitosa* | 148756 | 148797 | -1 | 2.71E-13 | *_* | *_* |
| R1107 | 42 | F | *Z. caespitosa* | 68543 | 68584 | *Z. caespitosa* | 148819 | 148860 | -1 | 2.71E-13 | *_* | *_* |
| R1108 | 42 | F | *Z. caespitosa* | 1496 | 1537 | *Z. caespitosa* | 84252 | 84293 | -3 | 6.66E-10 | *_* | *_* |
| R1109 | 42 | F | *Z. caespitosa* | 1496 | 1537 | *Z. caespitosa* | 53142 | 53183 | -3 | 6.66E-10 | *_* | *_* |
| R1110 | 42 | F | *Z. caespitosa* | 1496 | 1537 | *Z. caespitosa* | 135127 | 135168 | -3 | 6.66E-10 | *_* | *_* |
| R1111 | 42 | F | *Z. caespitosa* | 43444 | 43485 | *Z. caespitosa* | 84252 | 84293 | -3 | 6.66E-10 | *_* | *_* |
| R1112 | 42 | F | *Z. caespitosa* | 43444 | 43485 | *Z. caespitosa* | 53142 | 53183 | -3 | 6.66E-10 | *_* | *_* |
| R1113 | 42 | F | *Z. caespitosa* | 43444 | 43485 | *Z. caespitosa* | 135127 | 135168 | -3 | 6.66E-10 | *_* | *_* |
| R1114 | 42 | F | *Z. caespitosa* | 46018 | 46059 | *Z. caespitosa* | 84252 | 84293 | -3 | 6.66E-10 | *_* | *_* |
| R1115 | 42 | F | *Z. caespitosa* | 46018 | 46059 | *Z. caespitosa* | 53142 | 53183 | -3 | 6.66E-10 | *_* | *_* |
| R1116 | 42 | F | *Z. caespitosa* | 46018 | 46059 | *Z. caespitosa* | 135127 | 135168 | -3 | 6.66E-10 | *_* | *_* |
| R1117 | 42 | F | *Z. caespitosa* | 4072 | 4113 | *Z. caespitosa* | 84252 | 84293 | -3 | 6.66E-10 | *_* | *_* |
| R1118 | 42 | F | *Z. caespitosa* | 4072 | 4113 | *Z. caespitosa* | 53142 | 53183 | -3 | 6.66E-10 | *_* | *_* |
| R1119 | 42 | F | *Z. caespitosa* | 4072 | 4113 | *Z. caespitosa* | 135127 | 135168 | -3 | 6.66E-10 | *_* | *_* |
| R1120 | 42 | F | *Z. caespitosa* | 37788 | 37829 | *Z. caespitosa* | 75797 | 75838 | 0 | 2.15E-15 | *_* | *_* |
| R1121 | 42 | F | *Z. caespitosa* | 15976 | 16017 | *Z. caespitosa* | 149195 | 149236 | 0 | 2.15E-15 | *orf284a* | *_* |
| R1122 | 42 | F | *Z. caespitosa* | 7609 | 7650 | *Z. caespitosa* | 149195 | 149236 | 0 | 2.15E-15 | *_* | *_* |
| R1123 | 42 | F | *Z. caespitosa* | 69361 | 69402 | *Z. caespitosa* | 127176 | 127217 | -3 | 6.66E-10 | *orf118b* | *orf242a* |
| R1124 | 42 | F | *Z. caespitosa* | 125167 | 125208 | *Z. caespitosa* | 159740 | 159781 | -2 | 1.67E-11 | *_* | *rrn26* |
| R1125 | 42 | F | *Z. caespitosa* | 68546 | 68587 | *Z. caespitosa* | 186462 | 186503 | 0 | 2.15E-15 | *_* | *orf100b* |
| R1126 | 42 | F | *Z. caespitosa* | 37935 | 37976 | *Z. caespitosa* | 61320 | 61361 | -3 | 6.66E-10 | *_* | *orf189a* |
| R1127 | 42 | F | *Z. caespitosa* | 61320 | 61361 | *Z. caespitosa* | 108817 | 108858 | -3 | 6.66E-10 | *orf189a* | *orf161a* |
| R1128 | 42 | F | *Z. caespitosa* | 61320 | 61361 | *Z. caespitosa* | 84274 | 84315 | -3 | 6.66E-10 | *orf189a* | *_* |
| R1129 | 42 | F | *Z. caespitosa* | 52681 | 52722 | *Z. caespitosa* | 61320 | 61361 | -3 | 6.66E-10 | *orf172a* | *orf189a* |
| R1130 | 42 | F | *Z. caespitosa* | 52950 | 52991 | *Z. caespitosa* | 61320 | 61361 | -3 | 6.66E-10 | *orf172a* | *orf189a* |
| R1131 | 42 | F | *Z. caespitosa* | 53164 | 53205 | *Z. caespitosa* | 61320 | 61361 | -3 | 6.66E-10 | *_* | *orf189a* |
| R1132 | 42 | P | *Z. caespitosa* | 169354 | 169395 | *Z. caespitosa* | 190832 | 190873 | -2 | 1.67E-11 | *_* | *_* |
| R1133 | 42 | P | *Z. caespitosa* | 61320 | 61361 | *Z. caespitosa* | 188091 | 188132 | -3 | 6.66E-10 | *orf189a* | *_* |
| R1134 | 42 | P | *Z. caespitosa* | 162938 | 162979 | *Z. caespitosa* | 186471 | 186512 | -1 | 2.71E-13 | *_* | *orf100b* |
| R1135 | 42 | P | *Z. caespitosa* | 162938 | 162979 | *Z. caespitosa* | 181575 | 181616 | -1 | 2.71E-13 | *_* | *orf103d* |
| R1136 | 42 | P | *Z. caespitosa* | 138822 | 138863 | *Z. caespitosa* | 180606 | 180647 | 0 | 2.15E-15 | *_* | *_* |
| R1137 | 42 | P | *Z. caespitosa* | 61320 | 61361 | *Z. caespitosa* | 179917 | 179958 | -3 | 6.66E-10 | *orf189a* | *_* |
| R1138 | 42 | P | *Z. caespitosa* | 162938 | 162979 | *Z. caespitosa* | 177910 | 177951 | -1 | 2.71E-13 | *_* | *_* |
| R1139 | 42 | P | *Z. caespitosa* | 101696 | 101737 | *Z. caespitosa* | 177850 | 177891 | -2 | 1.67E-11 | *_* | *_* |
| R1140 | 42 | P | *Z. caespitosa* | 15976 | 16017 | *Z. caespitosa* | 175069 | 175110 | -2 | 1.67E-11 | *orf284a* | *_* |
| R1141 | 42 | P | *Z. caespitosa* | 7609 | 7650 | *Z. caespitosa* | 175069 | 175110 | -2 | 1.67E-11 | *_* | *_* |
| R1142 | 42 | P | *Z. caespitosa* | 34941 | 34982 | *Z. caespitosa* | 172949 | 172990 | 0 | 2.15E-15 | *_* | *orf185a* |
| R1143 | 42 | P | *Z. caespitosa* | 37788 | 37829 | *Z. caespitosa* | 172659 | 172700 | 0 | 2.15E-15 | *_* | *orf185a* |
| R1144 | 42 | P | *Z. caespitosa* | 135157 | 135198 | *Z. caespitosa* | 164648 | 164689 | -3 | 6.66E-10 | *_* | *_* |
| R1145 | 42 | P | *Z. caespitosa* | 83931 | 83972 | *Z. caespitosa* | 162938 | 162979 | -1 | 2.71E-13 | *_* | *_* |
| R1146 | 42 | P | *Z. caespitosa* | 136870 | 136911 | *Z. caespitosa* | 162938 | 162979 | -1 | 2.71E-13 | *_* | *_* |
| R1147 | 42 | P | *Z. caespitosa* | 4072 | 4113 | *Z. caespitosa* | 162910 | 162951 | -3 | 6.66E-10 | *_* | *_* |
| R1148 | 42 | P | *Z. caespitosa* | 46018 | 46059 | *Z. caespitosa* | 162910 | 162951 | -3 | 6.66E-10 | *_* | *_* |
| R1149 | 42 | P | *Z. caespitosa* | 43444 | 43485 | *Z. caespitosa* | 162910 | 162951 | -3 | 6.66E-10 | *_* | *_* |
| R1150 | 42 | P | *Z. caespitosa* | 1496 | 1537 | *Z. caespitosa* | 162910 | 162951 | -3 | 6.66E-10 | *_* | *_* |
| R1151 | 42 | P | *Z. caespitosa* | 31832 | 31873 | *Z. caespitosa* | 134528 | 134569 | -3 | 6.66E-10 | *_* | *orf160a* |
| R1152 | 42 | P | *Z. caespitosa* | 29354 | 29395 | *Z. caespitosa* | 127070 | 127111 | -3 | 6.66E-10 | *orf129a* | *orf242a* |
| R1153 | 42 | P | *Z. caespitosa* | 4506 | 4547 | *Z. caespitosa* | 101696 | 101737 | -2 | 1.67E-11 | *_* | *_* |
| R1154 | 42 | P | *Z. caespitosa* | 83871 | 83912 | *Z. caespitosa* | 101696 | 101737 | -2 | 1.67E-11 | *_* | *_* |
| R1155 | 42 | P | *Z. caespitosa* | 15987 | 16028 | *Z. caespitosa* | 75974 | 76015 | -2 | 1.67E-11 | *orf284a* | *_* |
| R1156 | 42 | P | *Z. caespitosa* | 7620 | 7661 | *Z. caespitosa* | 75974 | 76015 | -2 | 1.67E-11 | *_* | *_* |
| R1157 | 42 | P | *Z. caespitosa* | 68546 | 68587 | *Z. caespitosa* | 72265 | 72306 | 0 | 2.15E-15 | *_* | *_* |
| R1158 | 42 | P | *Z. caespitosa* | 61320 | 61361 | *Z. caespitosa* | 72206 | 72247 | -3 | 6.66E-10 | *orf189a* | *_* |
| R1159 | 42 | P | *Z. caespitosa* | 55482 | 55523 | *Z. caespitosa* | 65275 | 65316 | 0 | 2.15E-15 | *orf124a* | *_* |
| R1160 | 42 | P | *Z. caespitosa* | 48647 | 48688 | *Z. caespitosa* | 55115 | 55156 | 0 | 2.15E-15 | *_* | *_* |
| R1161 | 42 | P | *Z. caespitosa* | 34240 | 34281 | *Z. caespitosa* | 48647 | 48688 | 0 | 2.15E-15 | *_* | *_* |
| R1162 | 42 | P | *Z. caespitosa* | 27082 | 27123 | *Z. caespitosa* | 34915 | 34956 | 0 | 2.15E-15 | *_* | *_* |
| R1163 | 41 | F | *Z. caespitosa* | 163044 | 163084 | *Z. caespitosa* | 164695 | 164735 | 0 | 8.60E-15 | *_* | *_* |
| R1164 | 41 | F | *Z. caespitosa* | 163071 | 163111 | *Z. caespitosa* | 180009 | 180049 | 0 | 8.60E-15 | *_* | *_* |
| R1165 | 41 | F | *Z. caespitosa* | 84103 | 84143 | *Z. caespitosa* | 186560 | 186600 | -2 | 6.35E-11 | *_* | *orf100b* |
| R1166 | 41 | F | *Z. caespitosa* | 155657 | 155697 | *Z. caespitosa* | 155785 | 155825 | 0 | 8.60E-15 | *_* | *_* |
| R1167 | 41 | F | *Z. caespitosa* | 57073 | 57113 | *Z. caespitosa* | 74184 | 74224 | -3 | 2.47E-09 | *rrn18;orf152a* | *_* |
| R1168 | 41 | F | *Z. caespitosa* | 51632 | 51672 | *Z. caespitosa* | 51770 | 51810 | -3 | 2.47E-09 | *orf106b;ccmFn* | *orf106b;ccmFn* |
| R1169 | 41 | F | *Z. caespitosa* | 75994 | 76034 | *Z. caespitosa* | 100052 | 100092 | -1 | 1.06E-12 | *_* | *_* |
| R1170 | 41 | F | *Z. caespitosa* | 77622 | 77662 | *Z. caespitosa* | 138946 | 138986 | -3 | 2.47E-09 | *_* | *_* |
| R1171 | 41 | F | *Z. caespitosa* | 40263 | 40303 | *Z. caespitosa* | 126509 | 126549 | -3 | 2.47E-09 | *orf309a* | *orf242a* |
| R1172 | 41 | F | *Z. caespitosa* | 15987 | 16027 | *Z. caespitosa* | 111683 | 111723 | 0 | 8.60E-15 | *orf284a* | *_* |
| R1173 | 41 | F | *Z. caespitosa* | 7620 | 7660 | *Z. caespitosa* | 111683 | 111723 | 0 | 8.60E-15 | *_* | *_* |
| R1174 | 41 | F | *Z. caespitosa* | 84188 | 84228 | *Z. caespitosa* | 108718 | 108758 | 0 | 8.60E-15 | *_* | *orf161a* |
| R1175 | 41 | F | *Z. caespitosa* | 84188 | 84228 | *Z. caespitosa* | 148719 | 148759 | 0 | 8.60E-15 | *_* | *_* |
| R1176 | 41 | F | *Z. caespitosa* | 108718 | 108758 | *Z. caespitosa* | 137056 | 137096 | 0 | 8.60E-15 | *orf161a* | *_* |
| R1177 | 41 | F | *Z. caespitosa* | 137056 | 137096 | *Z. caespitosa* | 148719 | 148759 | 0 | 8.60E-15 | *_* | *_* |
| R1178 | 41 | F | *Z. caespitosa* | 37888 | 37928 | *Z. caespitosa* | 135046 | 135086 | -1 | 1.06E-12 | *_* | *_* |
| R1179 | 41 | F | *Z. caespitosa* | 108770 | 108810 | *Z. caespitosa* | 135046 | 135086 | -1 | 1.06E-12 | *orf161a* | *_* |
| R1180 | 41 | F | *Z. caespitosa* | 55331 | 55371 | *Z. caespitosa* | 158026 | 158066 | 0 | 8.60E-15 | *_* | *rrn26* |
| R1181 | 41 | F | *Z. caespitosa* | 52926 | 52966 | *Z. caespitosa* | 83524 | 83564 | 0 | 8.60E-15 | *orf172a* | *_* |
| R1182 | 41 | F | *Z. caespitosa* | 52926 | 52966 | *Z. caespitosa* | 177592 | 177632 | 0 | 8.60E-15 | *orf172a* | *_* |
| R1183 | 41 | F | *Z. caespitosa* | 9832 | 9872 | *Z. caespitosa* | 10210 | 10250 | -3 | 2.47E-09 | *orf397a* | *_* |
| R1184 | 41 | F | *Z. caespitosa* | 9129 | 9169 | *Z. caespitosa* | 10210 | 10250 | -3 | 2.47E-09 | *orf397a* | *_* |
| R1185 | 41 | F | *Z. caespitosa* | 15976 | 16016 | *Z. caespitosa* | 184970 | 185010 | -3 | 2.47E-09 | *orf284a* | *_* |
| R1186 | 41 | F | *Z. caespitosa* | 7609 | 7649 | *Z. caespitosa* | 184970 | 185010 | -3 | 2.47E-09 | *_* | *_* |
| R1187 | 41 | F | *Z. caespitosa* | 14573 | 14613 | *Z. caespitosa* | 111680 | 111720 | 0 | 8.60E-15 | *orf113a* | *_* |
| R1188 | 41 | F | *Z. caespitosa* | 69860 | 69900 | *Z. caespitosa* | 138801 | 138841 | 0 | 8.60E-15 | *_* | *_* |
| R1189 | 41 | F | *Z. caespitosa* | 131652 | 131692 | *Z. caespitosa* | 182718 | 182758 | -3 | 2.47E-09 | *_* | *_* |
| R1190 | 41 | F | *Z. caespitosa* | 25545 | 25585 | *Z. caespitosa* | 176058 | 176098 | -1 | 1.06E-12 | *orf340a* | *orf110b* |
| R1191 | 41 | F | *Z. caespitosa* | 96275 | 96315 | *Z. caespitosa* | 176058 | 176098 | -1 | 1.06E-12 | *orf540a-2* | *orf110b* |
| R1192 | 41 | F | *Z. caespitosa* | 79673 | 79713 | *Z. caespitosa* | 163015 | 163055 | 0 | 8.60E-15 | *_* | *_* |
| R1193 | 41 | F | *Z. caespitosa* | 153295 | 153335 | *Z. caespitosa* | 160760 | 160800 | 0 | 8.60E-15 | *_* | *atp9* |
| R1194 | 41 | F | *Z. caespitosa* | 24949 | 24989 | *Z. caespitosa* | 138799 | 138839 | 0 | 8.60E-15 | *_* | *_* |
| R1195 | 41 | F | *Z. caespitosa* | 95679 | 95719 | *Z. caespitosa* | 138799 | 138839 | 0 | 8.60E-15 | *_* | *_* |
| R1196 | 41 | F | *Z. caespitosa* | 138799 | 138839 | *Z. caespitosa* | 138823 | 138863 | 0 | 8.60E-15 | *_* | *_* |
| R1197 | 41 | F | *Z. caespitosa* | 77796 | 77836 | *Z. caespitosa* | 87373 | 87413 | 0 | 8.60E-15 | *_* | *orf122a* |
| R1198 | 41 | F | *Z. caespitosa* | 77734 | 77774 | *Z. caespitosa* | 87373 | 87413 | 0 | 8.60E-15 | *_* | *orf122a* |
| R1199 | 41 | F | *Z. caespitosa* | 79454 | 79494 | *Z. caespitosa* | 87373 | 87413 | 0 | 8.60E-15 | *_* | *orf122a* |
| R1200 | 41 | F | *Z. caespitosa* | 48643 | 48683 | *Z. caespitosa* | 172590 | 172630 | -3 | 2.47E-09 | *_* | *orf185a* |
| R1201 | 41 | F | *Z. caespitosa* | 9421 | 9461 | *Z. caespitosa* | 9805 | 9845 | -1 | 1.06E-12 | *orf397a* | *orf397a* |
| R1202 | 41 | F | *Z. caespitosa* | 9677 | 9717 | *Z. caespitosa* | 9805 | 9845 | -1 | 1.06E-12 | *orf397a* | *orf397a* |
| R1203 | 41 | P | *Z. caespitosa* | 15608 | 15648 | *Z. caespitosa* | 184138 | 184178 | -2 | 6.35E-11 | *_* | *orf130b* |
| R1204 | 41 | P | *Z. caespitosa* | 163015 | 163055 | *Z. caespitosa* | 181555 | 181595 | 0 | 8.60E-15 | *_* | *orf103d* |
| R1205 | 41 | P | *Z. caespitosa* | 138742 | 138782 | *Z. caespitosa* | 180606 | 180646 | 0 | 8.60E-15 | *_* | *_* |
| R1206 | 41 | P | *Z. caespitosa* | 138885 | 138925 | *Z. caespitosa* | 180606 | 180646 | 0 | 8.60E-15 | *_* | *_* |
| R1207 | 41 | P | *Z. caespitosa* | 177592 | 177632 | *Z. caespitosa* | 179942 | 179982 | 0 | 8.60E-15 | *_* | *_* |
| R1208 | 41 | P | *Z. caespitosa* | 83524 | 83564 | *Z. caespitosa* | 179942 | 179982 | 0 | 8.60E-15 | *_* | *_* |
| R1209 | 41 | P | *Z. caespitosa* | 87373 | 87413 | *Z. caespitosa* | 177982 | 178022 | 0 | 8.60E-15 | *orf122a* | *_* |
| R1210 | 41 | P | *Z. caespitosa* | 163015 | 163055 | *Z. caespitosa* | 177890 | 177930 | 0 | 8.60E-15 | *_* | *_* |
| R1211 | 41 | P | *Z. caespitosa* | 101729 | 101769 | *Z. caespitosa* | 177592 | 177632 | 0 | 8.60E-15 | *_* | *_* |
| R1212 | 41 | P | *Z. caespitosa* | 115440 | 115480 | *Z. caespitosa* | 176058 | 176098 | -1 | 1.06E-12 | *orf540a-1* | *orf110b* |
| R1213 | 41 | P | *Z. caespitosa* | 108718 | 108758 | *Z. caespitosa* | 164695 | 164735 | 0 | 8.60E-15 | *orf161a* | *_* |
| R1214 | 41 | P | *Z. caespitosa* | 148719 | 148759 | *Z. caespitosa* | 164695 | 164735 | 0 | 8.60E-15 | *_* | *_* |
| R1215 | 41 | P | *Z. caespitosa* | 137056 | 137096 | *Z. caespitosa* | 163044 | 163084 | 0 | 8.60E-15 | *_* | *_* |
| R1216 | 41 | P | *Z. caespitosa* | 84188 | 84228 | *Z. caespitosa* | 163044 | 163084 | 0 | 8.60E-15 | *_* | *_* |
| R1217 | 41 | P | *Z. caespitosa* | 35106 | 35146 | *Z. caespitosa* | 154861 | 154901 | -1 | 1.06E-12 | *_* | *_* |
| R1218 | 41 | P | *Z. caespitosa* | 79673 | 79713 | *Z. caespitosa* | 148811 | 148851 | 0 | 8.60E-15 | *_* | *_* |
| R1219 | 41 | P | *Z. caespitosa* | 79673 | 79713 | *Z. caespitosa* | 148748 | 148788 | 0 | 8.60E-15 | *_* | *_* |
| R1220 | 41 | P | *Z. caespitosa* | 116036 | 116076 | *Z. caespitosa* | 138799 | 138839 | 0 | 8.60E-15 | *_* | *_* |
| R1221 | 41 | P | *Z. caespitosa* | 87373 | 87413 | *Z. caespitosa* | 136942 | 136982 | 0 | 8.60E-15 | *orf122a* | *_* |
| R1222 | 41 | P | *Z. caespitosa* | 29910 | 29950 | *Z. caespitosa* | 126509 | 126549 | -3 | 2.47E-09 | *_* | *orf242a* |
| R1223 | 41 | P | *Z. caespitosa* | 75994 | 76034 | *Z. caespitosa* | 111664 | 111704 | -1 | 1.06E-12 | *_* | *_* |
| R1224 | 41 | P | *Z. caespitosa* | 83524 | 83564 | *Z. caespitosa* | 101729 | 101769 | 0 | 8.60E-15 | *_* | *_* |
| R1225 | 41 | P | *Z. caespitosa* | 14573 | 14613 | *Z. caespitosa* | 100036 | 100076 | 0 | 8.60E-15 | *orf113a* | *_* |
| R1226 | 41 | P | *Z. caespitosa* | 15987 | 16027 | *Z. caespitosa* | 100033 | 100073 | 0 | 8.60E-15 | *orf284a* | *_* |
| R1227 | 41 | P | *Z. caespitosa* | 7620 | 7660 | *Z. caespitosa* | 100033 | 100073 | 0 | 8.60E-15 | *_* | *_* |
| R1228 | 41 | P | *Z. caespitosa* | 72628 | 72668 | *Z. caespitosa* | 87373 | 87413 | 0 | 8.60E-15 | *_* | *orf122a* |
| R1229 | 41 | P | *Z. caespitosa* | 84003 | 84043 | *Z. caespitosa* | 87373 | 87413 | 0 | 8.60E-15 | *_* | *orf122a* |
| R1230 | 41 | P | *Z. caespitosa* | 37510 | 37550 | *Z. caespitosa* | 78603 | 78643 | 0 | 8.60E-15 | *_* | *_* |
| R1231 | 41 | P | *Z. caespitosa* | 14573 | 14613 | *Z. caespitosa* | 75978 | 76018 | -2 | 6.35E-11 | *orf113a* | *_* |
| R1232 | 41 | P | *Z. caespitosa* | 48643 | 48683 | *Z. caespitosa* | 75867 | 75907 | -3 | 2.47E-09 | *_* | *_* |
| R1233 | 41 | P | *Z. caespitosa* | 37750 | 37790 | *Z. caespitosa* | 74680 | 74720 | 0 | 8.60E-15 | *_* | *_* |
| R1234 | 41 | P | *Z. caespitosa* | 37875 | 37915 | *Z. caespitosa* | 72267 | 72307 | 0 | 8.60E-15 | *_* | *_* |
| R1235 | 40 | F | *Z. caespitosa* | 25003 | 25042 | *Z. caespitosa* | 111681 | 111720 | 0 | 3.44E-14 | *_* | *_* |
| R1236 | 40 | F | *Z. caespitosa* | 95733 | 95772 | *Z. caespitosa* | 111681 | 111720 | 0 | 3.44E-14 | *_* | *_* |
| R1237 | 40 | F | *Z. caespitosa* | 14574 | 14613 | *Z. caespitosa* | 138939 | 138978 | -1 | 4.13E-12 | *orf113a* | *_* |
| R1238 | 40 | F | *Z. caespitosa* | 111681 | 111720 | *Z. caespitosa* | 138939 | 138978 | -1 | 4.13E-12 | *_* | *_* |
| R1239 | 40 | F | *Z. caespitosa* | 9655 | 9694 | *Z. caespitosa* | 9846 | 9885 | -3 | 9.17E-09 | *orf397a* | *orf397a* |
| R1240 | 40 | F | *Z. caespitosa* | 24853 | 24892 | *Z. caespitosa* | 162655 | 162694 | -2 | 2.41E-10 | *trnE-UUC* | *_* |
| R1241 | 40 | F | *Z. caespitosa* | 24853 | 24892 | *Z. caespitosa* | 24890 | 24929 | -2 | 2.41E-10 | *trnE-UUC* | *_* |
| R1242 | 40 | F | *Z. caespitosa* | 24853 | 24892 | *Z. caespitosa* | 95620 | 95659 | -2 | 2.41E-10 | *trnE-UUC* | *_* |
| R1243 | 40 | F | *Z. caespitosa* | 95583 | 95622 | *Z. caespitosa* | 162655 | 162694 | -2 | 2.41E-10 | *trnE-UUC* | *_* |
| R1244 | 40 | F | *Z. caespitosa* | 24890 | 24929 | *Z. caespitosa* | 95583 | 95622 | -2 | 2.41E-10 | *_* | *trnE-UUC* |
| R1245 | 40 | F | *Z. caespitosa* | 95583 | 95622 | *Z. caespitosa* | 95620 | 95659 | -2 | 2.41E-10 | *trnE-UUC* | *_* |
| R1246 | 40 | F | *Z. caespitosa* | 57751 | 57790 | *Z. caespitosa* | 181554 | 181593 | 0 | 3.44E-14 | *orf117a* | *orf103d* |
| R1247 | 40 | F | *Z. caespitosa* | 57751 | 57790 | *Z. caespitosa* | 136849 | 136888 | 0 | 3.44E-14 | *orf117a* | *_* |
| R1248 | 40 | F | *Z. caespitosa* | 57751 | 57790 | *Z. caespitosa* | 177889 | 177928 | 0 | 3.44E-14 | *orf117a* | *_* |
| R1249 | 40 | F | *Z. caespitosa* | 57751 | 57790 | *Z. caespitosa* | 83910 | 83949 | 0 | 3.44E-14 | *orf117a* | *_* |
| R1250 | 40 | F | *Z. caespitosa* | 51686 | 51725 | *Z. caespitosa* | 51770 | 51809 | -2 | 2.41E-10 | *orf106b;ccmFn* | *orf106b;ccmFn* |
| R1251 | 40 | F | *Z. caespitosa* | 54458 | 54497 | *Z. caespitosa* | 188185 | 188224 | -3 | 9.17E-09 | *orf168a* | *_* |
| R1252 | 40 | F | *Z. caespitosa* | 72267 | 72306 | *Z. caespitosa* | 79663 | 79702 | 0 | 3.44E-14 | *_* | *_* |
| R1253 | 40 | F | *Z. caespitosa* | 53012 | 53051 | *Z. caespitosa* | 57632 | 57671 | 0 | 3.44E-14 | *_* | *orf117a* |
| R1254 | 40 | F | *Z. caespitosa* | 40323 | 40362 | *Z. caespitosa* | 126569 | 126608 | -3 | 9.17E-09 | *orf309a* | *orf242a* |
| R1255 | 40 | F | *Z. caespitosa* | 37811 | 37850 | *Z. caespitosa* | 186461 | 186500 | 0 | 3.44E-14 | *_* | *orf100b* |
| R1256 | 40 | F | *Z. caespitosa* | 37811 | 37850 | *Z. caespitosa* | 37875 | 37914 | 0 | 3.44E-14 | *_* | *_* |
| R1257 | 40 | F | *Z. caespitosa* | 41018 | 41057 | *Z. caespitosa* | 68430 | 68469 | -3 | 9.17E-09 | *orf309a* | *_* |
| R1258 | 40 | F | *Z. caespitosa* | 9719 | 9758 | *Z. caespitosa* | 9846 | 9885 | 0 | 3.44E-14 | *orf397a* | *orf397a* |
| R1259 | 40 | F | *Z. caespitosa* | 9783 | 9822 | *Z. caespitosa* | 9846 | 9885 | 0 | 3.44E-14 | *orf397a* | *orf397a* |
| R1260 | 40 | F | *Z. caespitosa* | 9335 | 9374 | *Z. caespitosa* | 9846 | 9885 | -1 | 4.13E-12 | *orf397a* | *orf397a* |
| R1261 | 40 | F | *Z. caespitosa* | 9271 | 9310 | *Z. caespitosa* | 9846 | 9885 | -1 | 4.13E-12 | *orf397a* | *orf397a* |
| R1262 | 40 | F | *Z. caespitosa* | 9207 | 9246 | *Z. caespitosa* | 9846 | 9885 | -1 | 4.13E-12 | *orf397a* | *orf397a* |
| R1263 | 40 | F | *Z. caespitosa* | 9463 | 9502 | *Z. caespitosa* | 9846 | 9885 | -1 | 4.13E-12 | *orf397a* | *orf397a* |
| R1264 | 40 | F | *Z. caespitosa* | 9527 | 9566 | *Z. caespitosa* | 9846 | 9885 | -1 | 4.13E-12 | *orf397a* | *orf397a* |
| R1265 | 40 | F | *Z. caespitosa* | 9591 | 9630 | *Z. caespitosa* | 9846 | 9885 | -1 | 4.13E-12 | *orf397a* | *orf397a* |
| R1266 | 40 | F | *Z. caespitosa* | 9399 | 9438 | *Z. caespitosa* | 9846 | 9885 | -2 | 2.41E-10 | *orf397a* | *orf397a* |
| R1267 | 40 | F | *Z. caespitosa* | 112718 | 112757 | *Z. caespitosa* | 152162 | 152201 | -3 | 9.17E-09 | *_* | *_* |
| R1268 | 40 | F | *Z. caespitosa* | 26217 | 26256 | *Z. caespitosa* | 174709 | 174748 | -1 | 4.13E-12 | *_* | *orf167b* |
| R1269 | 40 | F | *Z. caespitosa* | 32004 | 32043 | *Z. caespitosa* | 134036 | 134075 | -3 | 9.17E-09 | *orf127a* | *_* |
| R1270 | 40 | F | *Z. caespitosa* | 113574 | 113613 | *Z. caespitosa* | 169361 | 169400 | 0 | 3.44E-14 | *_* | *_* |
| R1271 | 40 | F | *Z. caespitosa* | 38004 | 38043 | *Z. caespitosa* | 178039 | 178078 | 0 | 3.44E-14 | *_* | *_* |
| R1272 | 40 | F | *Z. caespitosa* | 116096 | 116135 | *Z. caespitosa* | 116133 | 116172 | -2 | 2.41E-10 | *_* | *trnE-UUC* |
| R1273 | 40 | F | *Z. caespitosa* | 34290 | 34329 | *Z. caespitosa* | 34986 | 35025 | -1 | 4.13E-12 | *_* | *_* |
| R1274 | 40 | F | *Z. caespitosa* | 57249 | 57288 | *Z. caespitosa* | 74362 | 74401 | 0 | 3.44E-14 | *rrn18;orf152a* | *_* |
| R1275 | 40 | F | *Z. caespitosa* | 125238 | 125277 | *Z. caespitosa* | 159811 | 159850 | -3 | 9.17E-09 | *_* | *rrn26* |
| R1276 | 40 | F | *Z. caespitosa* | 10102 | 10141 | *Z. caespitosa* | 10165 | 10204 | -3 | 9.17E-09 | *orf397a* | *orf397a* |
| R1277 | 40 | F | *Z. caespitosa* | 10039 | 10078 | *Z. caespitosa* | 10102 | 10141 | -3 | 9.17E-09 | *orf397a* | *orf397a* |
| R1278 | 40 | F | *Z. caespitosa* | 1440 | 1479 | *Z. caespitosa* | 45955 | 45994 | -1 | 4.13E-12 | *_* | *_* |
| R1279 | 40 | F | *Z. caespitosa* | 1440 | 1479 | *Z. caespitosa* | 4009 | 4048 | -1 | 4.13E-12 | *_* | *_* |
| R1280 | 40 | F | *Z. caespitosa* | 43388 | 43427 | *Z. caespitosa* | 45955 | 45994 | -1 | 4.13E-12 | *_* | *_* |
| R1281 | 40 | F | *Z. caespitosa* | 4009 | 4048 | *Z. caespitosa* | 43388 | 43427 | -1 | 4.13E-12 | *_* | *_* |
| R1282 | 40 | F | *Z. caespitosa* | 100036 | 100075 | *Z. caespitosa* | 115983 | 116022 | 0 | 3.44E-14 | *_* | *_* |
| R1283 | 40 | F | *Z. caespitosa* | 75978 | 76017 | *Z. caespitosa* | 115983 | 116022 | -2 | 2.41E-10 | *_* | *_* |
| R1284 | 40 | F | *Z. caespitosa* | 40807 | 40846 | *Z. caespitosa* | 127053 | 127092 | -3 | 9.17E-09 | *orf309a* | *orf242a* |
| R1285 | 40 | F | *Z. caespitosa* | 52658 | 52697 | *Z. caespitosa* | 83525 | 83564 | 0 | 3.44E-14 | *orf172a* | *_* |
| R1286 | 40 | F | *Z. caespitosa* | 52658 | 52697 | *Z. caespitosa* | 177593 | 177632 | 0 | 3.44E-14 | *orf172a* | *_* |
| R1287 | 40 | F | *Z. caespitosa* | 37876 | 37915 | *Z. caespitosa* | 68546 | 68585 | 0 | 3.44E-14 | *_* | *_* |
| R1288 | 40 | F | *Z. caespitosa* | 37876 | 37915 | *Z. caespitosa* | 181566 | 181605 | 0 | 3.44E-14 | *_* | *orf103d* |
| R1289 | 40 | F | *Z. caespitosa* | 37876 | 37915 | *Z. caespitosa* | 136861 | 136900 | 0 | 3.44E-14 | *_* | *_* |
| R1290 | 40 | F | *Z. caespitosa* | 37876 | 37915 | *Z. caespitosa* | 177901 | 177940 | 0 | 3.44E-14 | *_* | *_* |
| R1291 | 40 | F | *Z. caespitosa* | 37876 | 37915 | *Z. caespitosa* | 83922 | 83961 | 0 | 3.44E-14 | *_* | *_* |
| R1292 | 40 | F | *Z. caespitosa* | 41607 | 41646 | *Z. caespitosa* | 69079 | 69118 | -3 | 9.17E-09 | *orf120b* | *_* |
| R1293 | 40 | P | *Z. caespitosa* | 177593 | 177632 | *Z. caespitosa* | 188116 | 188155 | 0 | 3.44E-14 | *_* | *_* |
| R1294 | 40 | P | *Z. caespitosa* | 83525 | 83564 | *Z. caespitosa* | 188116 | 188155 | 0 | 3.44E-14 | *_* | *_* |
| R1295 | 40 | P | *Z. caespitosa* | 79663 | 79702 | *Z. caespitosa* | 186462 | 186501 | 0 | 3.44E-14 | *_* | *orf100b* |
| R1296 | 40 | P | *Z. caespitosa* | 134036 | 134075 | *Z. caespitosa* | 173993 | 174032 | -3 | 9.17E-09 | *_* | *_* |
| R1297 | 40 | P | *Z. caespitosa* | 98143 | 98182 | *Z. caespitosa* | 169361 | 169400 | 0 | 3.44E-14 | *_* | *_* |
| R1298 | 40 | P | *Z. caespitosa* | 116133 | 116172 | *Z. caespitosa* | 162655 | 162694 | -2 | 2.41E-10 | *trnE-UUC* | *_* |
| R1299 | 40 | P | *Z. caespitosa* | 98999 | 99038 | *Z. caespitosa* | 152162 | 152201 | -3 | 9.17E-09 | *_* | *_* |
| R1300 | 40 | P | *Z. caespitosa* | 92111 | 92150 | *Z. caespitosa* | 149706 | 149745 | -2 | 2.41E-10 | *_* | *_* |
| R1301 | 40 | P | *Z. caespitosa* | 100036 | 100075 | *Z. caespitosa* | 138939 | 138978 | -1 | 4.13E-12 | *_* | *_* |
| R1302 | 40 | P | *Z. caespitosa* | 75978 | 76017 | *Z. caespitosa* | 138939 | 138978 | -3 | 9.17E-09 | *_* | *_* |
| R1303 | 40 | P | *Z. caespitosa* | 24890 | 24929 | *Z. caespitosa* | 116133 | 116172 | -2 | 2.41E-10 | *_* | *trnE-UUC* |
| R1304 | 40 | P | *Z. caespitosa* | 95620 | 95659 | *Z. caespitosa* | 116133 | 116172 | -2 | 2.41E-10 | *_* | *trnE-UUC* |
| R1305 | 40 | P | *Z. caespitosa* | 95583 | 95622 | *Z. caespitosa* | 116096 | 116135 | -2 | 2.41E-10 | *trnE-UUC* | *_* |
| R1306 | 40 | P | *Z. caespitosa* | 24853 | 24892 | *Z. caespitosa* | 116096 | 116135 | -2 | 2.41E-10 | *trnE-UUC* | *_* |
| R1307 | 40 | P | *Z. caespitosa* | 111681 | 111720 | *Z. caespitosa* | 115983 | 116022 | 0 | 3.44E-14 | *_* | *_* |
| R1308 | 40 | P | *Z. caespitosa* | 95733 | 95772 | *Z. caespitosa* | 100036 | 100075 | 0 | 3.44E-14 | *_* | *_* |
| R1309 | 40 | P | *Z. caespitosa* | 25003 | 25042 | *Z. caespitosa* | 100036 | 100075 | 0 | 3.44E-14 | *_* | *_* |
| R1310 | 40 | P | *Z. caespitosa* | 75978 | 76017 | *Z. caespitosa* | 95733 | 95772 | -2 | 2.41E-10 | *_* | *_* |
| R1311 | 40 | P | *Z. caespitosa* | 54458 | 54497 | *Z. caespitosa* | 83578 | 83617 | -3 | 9.17E-09 | *orf168a* | *_* |
| R1312 | 40 | P | *Z. caespitosa* | 51620 | 51659 | *Z. caespitosa* | 79522 | 79561 | -1 | 4.13E-12 | *orf106b;ccmFn* | *_* |
| R1313 | 40 | P | *Z. caespitosa* | 25003 | 25042 | *Z. caespitosa* | 75978 | 76017 | -2 | 2.41E-10 | *_* | *_* |
| R1314 | 40 | P | *Z. caespitosa* | 24928 | 24967 | *Z. caespitosa* | 75664 | 75703 | 0 | 3.44E-14 | *_* | *_* |
| R1315 | 40 | P | *Z. caespitosa* | 52638 | 52677 | *Z. caespitosa* | 74639 | 74678 | 0 | 3.44E-14 | *orf172a* | *_* |
| R1316 | 40 | P | *Z. caespitosa* | 37811 | 37850 | *Z. caespitosa* | 72268 | 72307 | 0 | 3.44E-14 | *_* | *_* |
| R1317 | 40 | P | *Z. caespitosa* | 4124 | 4163 | *Z. caespitosa* | 54458 | 54497 | -3 | 9.17E-09 | *_* | *orf168a* |
| R1318 | 40 | P | *Z. caespitosa* | 46070 | 46109 | *Z. caespitosa* | 54458 | 54497 | -3 | 9.17E-09 | *_* | *orf168a* |
| R1319 | 40 | P | *Z. caespitosa* | 43496 | 43535 | *Z. caespitosa* | 54458 | 54497 | -3 | 9.17E-09 | *_* | *orf168a* |
| R1320 | 40 | P | *Z. caespitosa* | 1548 | 1587 | *Z. caespitosa* | 54458 | 54497 | -3 | 9.17E-09 | *_* | *orf168a* |
| R1321 | 40 | P | *Z. caespitosa* | 16191 | 16230 | *Z. caespitosa* | 52638 | 52677 | 0 | 3.44E-14 | *orf284a* | *orf172a* |
| R1322 | 40 | P | *Z. caespitosa* | 28930 | 28969 | *Z. caespitosa* | 41255 | 41294 | -2 | 2.41E-10 | *_* | *_* |
| R1323 | 40 | P | *Z. caespitosa* | 34290 | 34329 | *Z. caespitosa* | 36818 | 36857 | -1 | 4.13E-12 | *_* | *_* |
| R1324 | 39 | F | *Z. caespitosa* | 15365 | 15403 | *Z. caespitosa* | 49701 | 49739 | 0 | 1.38E-13 | *orf295a;cox2* | *_* |
| R1325 | 39 | F | *Z. caespitosa* | 15600 | 15638 | *Z. caespitosa* | 79502 | 79540 | -3 | 3.39E-08 | *_* | *_* |
| R1326 | 39 | F | *Z. caespitosa* | 34106 | 34144 | *Z. caespitosa* | 79408 | 79446 | -2 | 9.17E-10 | *_* | *ccmFc;orf235a* |
| R1327 | 39 | F | *Z. caespitosa* | 72289 | 72327 | *Z. caespitosa* | 179964 | 180002 | -2 | 9.17E-10 | *_* | *_* |
| R1328 | 39 | F | *Z. caespitosa* | 7978 | 8016 | *Z. caespitosa* | 14209 | 14247 | -3 | 3.39E-08 | *_* | *_* |
| R1329 | 39 | F | *Z. caespitosa* | 156613 | 156651 | *Z. caespitosa* | 181644 | 181682 | -2 | 9.17E-10 | *_* | *orf103d* |
| R1330 | 39 | F | *Z. caespitosa* | 75914 | 75952 | *Z. caespitosa* | 156613 | 156651 | -2 | 9.17E-10 | *_* | *_* |
| R1331 | 39 | F | *Z. caespitosa* | 156613 | 156651 | *Z. caespitosa* | 184206 | 184244 | -2 | 9.17E-10 | *_* | *orf130b* |
| R1332 | 39 | F | *Z. caespitosa* | 136939 | 136977 | *Z. caespitosa* | 156613 | 156651 | -2 | 9.17E-10 | *_* | *_* |
| R1333 | 39 | F | *Z. caespitosa* | 72625 | 72663 | *Z. caespitosa* | 156613 | 156651 | -2 | 9.17E-10 | *_* | *_* |
| R1334 | 39 | F | *Z. caespitosa* | 156613 | 156651 | *Z. caespitosa* | 177979 | 178017 | -2 | 9.17E-10 | *_* | *_* |
| R1335 | 39 | F | *Z. caespitosa* | 84000 | 84038 | *Z. caespitosa* | 156613 | 156651 | -2 | 9.17E-10 | *_* | *_* |
| R1336 | 39 | F | *Z. caespitosa* | 51709 | 51747 | *Z. caespitosa* | 181600 | 181638 | -3 | 3.39E-08 | *orf106b;ccmFn* | *orf103d* |
| R1337 | 39 | F | *Z. caespitosa* | 51709 | 51747 | *Z. caespitosa* | 136895 | 136933 | -3 | 3.39E-08 | *orf106b;ccmFn* | *_* |
| R1338 | 39 | F | *Z. caespitosa* | 51709 | 51747 | *Z. caespitosa* | 177935 | 177973 | -3 | 3.39E-08 | *orf106b;ccmFn* | *_* |
| R1339 | 39 | F | *Z. caespitosa* | 51709 | 51747 | *Z. caespitosa* | 83956 | 83994 | -3 | 3.39E-08 | *orf106b;ccmFn* | *_* |
| R1340 | 39 | F | *Z. caespitosa* | 51709 | 51747 | *Z. caespitosa* | 75870 | 75908 | -3 | 3.39E-08 | *orf106b;ccmFn* | *_* |
| R1341 | 39 | F | *Z. caespitosa* | 57752 | 57790 | *Z. caespitosa* | 148748 | 148786 | 0 | 1.38E-13 | *orf117a* | *_* |
| R1342 | 39 | F | *Z. caespitosa* | 57752 | 57790 | *Z. caespitosa* | 148811 | 148849 | 0 | 1.38E-13 | *orf117a* | *_* |
| R1343 | 39 | F | *Z. caespitosa* | 57752 | 57790 | *Z. caespitosa* | 108747 | 108785 | -1 | 1.61E-11 | *orf117a* | *orf161a* |
| R1344 | 39 | F | *Z. caespitosa* | 57166 | 57204 | *Z. caespitosa* | 74278 | 74316 | -3 | 3.39E-08 | *rrn18;orf152a* | *_* |
| R1345 | 39 | F | *Z. caespitosa* | 14670 | 14708 | *Z. caespitosa* | 25104 | 25142 | -3 | 3.39E-08 | *orf295a;cox2* | *orf340a* |
| R1346 | 39 | F | *Z. caespitosa* | 14670 | 14708 | *Z. caespitosa* | 95834 | 95872 | -3 | 3.39E-08 | *orf295a;cox2* | *orf540a-2* |
| R1347 | 39 | F | *Z. caespitosa* | 69860 | 69898 | *Z. caespitosa* | 138744 | 138782 | 0 | 1.38E-13 | *_* | *_* |
| R1348 | 39 | F | *Z. caespitosa* | 69860 | 69898 | *Z. caespitosa* | 138887 | 138925 | 0 | 1.38E-13 | *_* | *_* |
| R1349 | 39 | F | *Z. caespitosa* | 69860 | 69898 | *Z. caespitosa* | 138825 | 138863 | 0 | 1.38E-13 | *_* | *_* |
| R1350 | 39 | F | *Z. caespitosa* | 87390 | 87428 | *Z. caespitosa* | 140231 | 140269 | -3 | 3.39E-08 | *orf122a* | *matR* |
| R1351 | 39 | F | *Z. caespitosa* | 51619 | 51657 | *Z. caespitosa* | 75819 | 75857 | -1 | 1.61E-11 | *orf106b;ccmFn* | *_* |
| R1352 | 39 | F | *Z. caespitosa* | 53013 | 53051 | *Z. caespitosa* | 75650 | 75688 | 0 | 1.38E-13 | *_* | *_* |
| R1353 | 39 | F | *Z. caespitosa* | 37812 | 37850 | *Z. caespitosa* | 108758 | 108796 | -1 | 1.61E-11 | *_* | *orf161a* |
| R1354 | 39 | F | *Z. caespitosa* | 33116 | 33154 | *Z. caespitosa* | 136779 | 136817 | -2 | 9.17E-10 | *_* | *_* |
| R1355 | 39 | F | *Z. caespitosa* | 135157 | 135195 | *Z. caespitosa* | 180814 | 180852 | -2 | 9.17E-10 | *_* | *_* |
| R1356 | 39 | F | *Z. caespitosa* | 37745 | 37783 | *Z. caespitosa* | 108693 | 108731 | 0 | 1.38E-13 | *_* | *orf161a* |
| R1357 | 39 | F | *Z. caespitosa* | 40767 | 40805 | *Z. caespitosa* | 127013 | 127051 | -3 | 3.39E-08 | *orf309a* | *orf242a* |
| R1358 | 39 | F | *Z. caespitosa* | 37812 | 37850 | *Z. caespitosa* | 68546 | 68584 | 0 | 1.38E-13 | *_* | *_* |
| R1359 | 39 | F | *Z. caespitosa* | 37812 | 37850 | *Z. caespitosa* | 181566 | 181604 | 0 | 1.38E-13 | *_* | *orf103d* |
| R1360 | 39 | F | *Z. caespitosa* | 37812 | 37850 | *Z. caespitosa* | 136861 | 136899 | 0 | 1.38E-13 | *_* | *_* |
| R1361 | 39 | F | *Z. caespitosa* | 37812 | 37850 | *Z. caespitosa* | 177901 | 177939 | 0 | 1.38E-13 | *_* | *_* |
| R1362 | 39 | F | *Z. caespitosa* | 37812 | 37850 | *Z. caespitosa* | 83922 | 83960 | 0 | 1.38E-13 | *_* | *_* |
| R1363 | 39 | F | *Z. caespitosa* | 148759 | 148797 | *Z. caespitosa* | 186462 | 186500 | -1 | 1.61E-11 | *_* | *orf100b* |
| R1364 | 39 | F | *Z. caespitosa* | 37876 | 37914 | *Z. caespitosa* | 148759 | 148797 | -1 | 1.61E-11 | *_* | *_* |
| R1365 | 39 | F | *Z. caespitosa* | 148822 | 148860 | *Z. caespitosa* | 186462 | 186500 | -1 | 1.61E-11 | *_* | *orf100b* |
| R1366 | 39 | F | *Z. caespitosa* | 37876 | 37914 | *Z. caespitosa* | 148822 | 148860 | -1 | 1.61E-11 | *_* | *_* |
| R1367 | 39 | F | *Z. caespitosa* | 9719 | 9757 | *Z. caespitosa* | 9972 | 10010 | -3 | 3.39E-08 | *orf397a* | *orf397a* |
| R1368 | 39 | F | *Z. caespitosa* | 9783 | 9821 | *Z. caespitosa* | 9972 | 10010 | -3 | 3.39E-08 | *orf397a* | *orf397a* |
| R1369 | 39 | P | *Z. caespitosa* | 101699 | 101737 | *Z. caespitosa* | 180814 | 180852 | -2 | 9.17E-10 | *_* | *_* |
| R1370 | 39 | P | *Z. caespitosa* | 162883 | 162921 | *Z. caespitosa* | 180814 | 180852 | -2 | 9.17E-10 | *_* | *_* |
| R1371 | 39 | P | *Z. caespitosa* | 51619 | 51657 | *Z. caespitosa* | 172640 | 172678 | -1 | 1.61E-11 | *orf106b;ccmFn* | *orf185a* |
| R1372 | 39 | P | *Z. caespitosa* | 51709 | 51747 | *Z. caespitosa* | 172589 | 172627 | -3 | 3.39E-08 | *orf106b;ccmFn* | *orf185a* |
| R1373 | 39 | P | *Z. caespitosa* | 156613 | 156651 | *Z. caespitosa* | 172545 | 172583 | -2 | 9.17E-10 | *_* | *_* |
| R1374 | 39 | P | *Z. caespitosa* | 57752 | 57790 | *Z. caespitosa* | 163017 | 163055 | 0 | 1.38E-13 | *orf117a* | *_* |
| R1375 | 39 | P | *Z. caespitosa* | 37812 | 37850 | *Z. caespitosa* | 163006 | 163044 | -1 | 1.61E-11 | *_* | *_* |
| R1376 | 39 | P | *Z. caespitosa* | 74685 | 74723 | *Z. caespitosa* | 156613 | 156651 | -2 | 9.17E-10 | *_* | *_* |
| R1377 | 39 | P | *Z. caespitosa* | 77801 | 77839 | *Z. caespitosa* | 156613 | 156651 | -2 | 9.17E-10 | *_* | *_* |
| R1378 | 39 | P | *Z. caespitosa* | 140114 | 140152 | *Z. caespitosa* | 156613 | 156651 | -2 | 9.17E-10 | *matR* | *_* |
| R1379 | 39 | P | *Z. caespitosa* | 77739 | 77777 | *Z. caespitosa* | 156613 | 156651 | -2 | 9.17E-10 | *_* | *_* |
| R1380 | 39 | P | *Z. caespitosa* | 77677 | 77715 | *Z. caespitosa* | 156613 | 156651 | -2 | 9.17E-10 | *_* | *_* |
| R1381 | 39 | P | *Z. caespitosa* | 79459 | 79497 | *Z. caespitosa* | 156613 | 156651 | -2 | 9.17E-10 | *_* | *_* |
| R1382 | 39 | P | *Z. caespitosa* | 140231 | 140269 | *Z. caespitosa* | 156571 | 156609 | -3 | 3.39E-08 | *matR* | *_* |
| R1383 | 39 | P | *Z. caespitosa* | 72268 | 72306 | *Z. caespitosa* | 148822 | 148860 | -1 | 1.61E-11 | *_* | *_* |
| R1384 | 39 | P | *Z. caespitosa* | 72268 | 72306 | *Z. caespitosa* | 148759 | 148797 | -1 | 1.61E-11 | *_* | *_* |
| R1385 | 39 | P | *Z. caespitosa* | 37735 | 37773 | *Z. caespitosa* | 140231 | 140269 | -3 | 3.39E-08 | *_* | *matR* |
| R1386 | 39 | P | *Z. caespitosa* | 52592 | 52630 | *Z. caespitosa* | 135916 | 135954 | 0 | 1.38E-13 | *orf172a* | *_* |
| R1387 | 39 | P | *Z. caespitosa* | 29921 | 29959 | *Z. caespitosa* | 126500 | 126538 | -2 | 9.17E-10 | *_* | *orf242a* |
| R1388 | 39 | P | *Z. caespitosa* | 14670 | 14708 | *Z. caespitosa* | 115883 | 115921 | -3 | 3.39E-08 | *orf295a;cox2* | *orf540a-1* |
| R1389 | 39 | P | *Z. caespitosa* | 87380 | 87418 | *Z. caespitosa* | 108693 | 108731 | 0 | 1.38E-13 | *orf122a* | *orf161a* |
| R1390 | 39 | P | *Z. caespitosa* | 41584 | 41622 | *Z. caespitosa* | 106211 | 106249 | 0 | 1.38E-13 | *orf120b* | *_* |
| R1391 | 39 | P | *Z. caespitosa* | 67185 | 67223 | *Z. caespitosa* | 84894 | 84932 | 0 | 1.38E-13 | *_* | *_* |
| R1392 | 39 | P | *Z. caespitosa* | 37812 | 37850 | *Z. caespitosa* | 79664 | 79702 | 0 | 1.38E-13 | *_* | *_* |
| R1393 | 39 | P | *Z. caespitosa* | 51709 | 51747 | *Z. caespitosa* | 77845 | 77883 | -3 | 3.39E-08 | *orf106b;ccmFn* | *_* |
| R1394 | 39 | P | *Z. caespitosa* | 4072 | 4110 | *Z. caespitosa* | 72231 | 72269 | -1 | 1.61E-11 | *_* | *_* |
| R1395 | 39 | P | *Z. caespitosa* | 46018 | 46056 | *Z. caespitosa* | 72231 | 72269 | -1 | 1.61E-11 | *_* | *_* |
| R1396 | 39 | P | *Z. caespitosa* | 43444 | 43482 | *Z. caespitosa* | 72231 | 72269 | -1 | 1.61E-11 | *_* | *_* |
| R1397 | 39 | P | *Z. caespitosa* | 1496 | 1534 | *Z. caespitosa* | 72231 | 72269 | -1 | 1.61E-11 | *_* | *_* |
| R1398 | 39 | P | *Z. caespitosa* | 51620 | 51658 | *Z. caespitosa* | 71945 | 71983 | -1 | 1.61E-11 | *orf106b;ccmFn* | *_* |
| R1399 | 38 | F | *Z. caespitosa* | 9949 | 9986 | *Z. caespitosa* | 10138 | 10175 | -2 | 3.48E-09 | *orf397a* | *orf397a* |
| R1400 | 38 | F | *Z. caespitosa* | 9949 | 9986 | *Z. caespitosa* | 10012 | 10049 | -2 | 3.48E-09 | *orf397a* | *orf397a* |
| R1401 | 38 | F | *Z. caespitosa* | 59173 | 59210 | *Z. caespitosa* | 101657 | 101694 | 0 | 5.50E-13 | *_* | *_* |
| R1402 | 38 | F | *Z. caespitosa* | 59173 | 59210 | *Z. caespitosa* | 162674 | 162711 | -3 | 1.25E-07 | *_* | *_* |
| R1403 | 38 | F | *Z. caespitosa* | 59173 | 59210 | *Z. caespitosa* | 68365 | 68402 | -3 | 1.25E-07 | *_* | *_* |
| R1404 | 38 | F | *Z. caespitosa* | 24909 | 24946 | *Z. caespitosa* | 59173 | 59210 | -3 | 1.25E-07 | *_* | *_* |
| R1405 | 38 | F | *Z. caespitosa* | 59173 | 59210 | *Z. caespitosa* | 95639 | 95676 | -3 | 1.25E-07 | *_* | *_* |
| R1406 | 38 | F | *Z. caespitosa* | 59173 | 59210 | *Z. caespitosa* | 172811 | 172848 | -3 | 1.25E-07 | *_* | *orf185a* |
| R1407 | 38 | F | *Z. caespitosa* | 59173 | 59210 | *Z. caespitosa* | 138669 | 138706 | -3 | 1.25E-07 | *_* | *_* |
| R1408 | 38 | F | *Z. caespitosa* | 117759 | 117796 | *Z. caespitosa* | 175083 | 175120 | -2 | 3.48E-09 | *_* | *_* |
| R1409 | 38 | F | *Z. caespitosa* | 24855 | 24892 | *Z. caespitosa* | 68348 | 68385 | -2 | 3.48E-09 | *trnE-UUC* | *_* |
| R1410 | 38 | F | *Z. caespitosa* | 24855 | 24892 | *Z. caespitosa* | 172794 | 172831 | -2 | 3.48E-09 | *trnE-UUC* | *orf185a* |
| R1411 | 38 | F | *Z. caespitosa* | 24855 | 24892 | *Z. caespitosa* | 138652 | 138689 | -2 | 3.48E-09 | *trnE-UUC* | *_* |
| R1412 | 38 | F | *Z. caespitosa* | 68348 | 68385 | *Z. caespitosa* | 95585 | 95622 | -2 | 3.48E-09 | *_* | *trnE-UUC* |
| R1413 | 38 | F | *Z. caespitosa* | 95585 | 95622 | *Z. caespitosa* | 172794 | 172831 | -2 | 3.48E-09 | *trnE-UUC* | *orf185a* |
| R1414 | 38 | F | *Z. caespitosa* | 95585 | 95622 | *Z. caespitosa* | 138652 | 138689 | -2 | 3.48E-09 | *trnE-UUC* | *_* |
| R1415 | 38 | F | *Z. caespitosa* | 48412 | 48449 | *Z. caespitosa* | 87380 | 87417 | -1 | 6.27E-11 | *_* | *orf122a* |
| R1416 | 38 | F | *Z. caespitosa* | 71946 | 71983 | *Z. caespitosa* | 172640 | 172677 | 0 | 5.50E-13 | *_* | *orf185a* |
| R1417 | 38 | F | *Z. caespitosa* | 79524 | 79561 | *Z. caespitosa* | 172640 | 172677 | 0 | 5.50E-13 | *_* | *orf185a* |
| R1418 | 38 | F | *Z. caespitosa* | 41499 | 41536 | *Z. caespitosa* | 68971 | 69008 | -3 | 1.25E-07 | *orf120b* | *_* |
| R1419 | 38 | F | *Z. caespitosa* | 108851 | 108888 | *Z. caespitosa* | 177864 | 177901 | -1 | 6.27E-11 | *orf161a* | *_* |
| R1420 | 38 | F | *Z. caespitosa* | 83885 | 83922 | *Z. caespitosa* | 108851 | 108888 | -1 | 6.27E-11 | *_* | *orf161a* |
| R1421 | 38 | F | *Z. caespitosa* | 40275 | 40312 | *Z. caespitosa* | 126521 | 126558 | -3 | 1.25E-07 | *orf309a* | *orf242a* |
| R1422 | 38 | F | *Z. caespitosa* | 131382 | 131419 | *Z. caespitosa* | 182455 | 182492 | -3 | 1.25E-07 | *orf131b* | *orf117c* |
| R1423 | 38 | F | *Z. caespitosa* | 15987 | 16024 | *Z. caespitosa* | 25005 | 25042 | 0 | 5.50E-13 | *orf284a* | *_* |
| R1424 | 38 | F | *Z. caespitosa* | 7620 | 7657 | *Z. caespitosa* | 25005 | 25042 | 0 | 5.50E-13 | *_* | *_* |
| R1425 | 38 | F | *Z. caespitosa* | 15987 | 16024 | *Z. caespitosa* | 95735 | 95772 | 0 | 5.50E-13 | *orf284a* | *_* |
| R1426 | 38 | F | *Z. caespitosa* | 7620 | 7657 | *Z. caespitosa* | 95735 | 95772 | 0 | 5.50E-13 | *_* | *_* |
| R1427 | 38 | F | *Z. caespitosa* | 14576 | 14613 | *Z. caespitosa* | 15987 | 16024 | 0 | 5.50E-13 | *orf113a* | *orf284a* |
| R1428 | 38 | F | *Z. caespitosa* | 7620 | 7657 | *Z. caespitosa* | 14576 | 14613 | 0 | 5.50E-13 | *_* | *orf113a* |
| R1429 | 38 | F | *Z. caespitosa* | 15987 | 16024 | *Z. caespitosa* | 138941 | 138978 | -1 | 6.27E-11 | *orf284a* | *_* |
| R1430 | 38 | F | *Z. caespitosa* | 7620 | 7657 | *Z. caespitosa* | 138941 | 138978 | -1 | 6.27E-11 | *_* | *_* |
| R1431 | 38 | F | *Z. caespitosa* | 51674 | 51711 | *Z. caespitosa* | 75820 | 75857 | 0 | 5.50E-13 | *orf106b;ccmFn* | *_* |
| R1432 | 38 | F | *Z. caespitosa* | 40368 | 40405 | *Z. caespitosa* | 126614 | 126651 | -3 | 1.25E-07 | *orf309a* | *orf242a* |
| R1433 | 38 | F | *Z. caespitosa* | 101646 | 101683 | *Z. caespitosa* | 172764 | 172801 | 0 | 5.50E-13 | *_* | *orf185a* |
| R1434 | 38 | F | *Z. caespitosa* | 162663 | 162700 | *Z. caespitosa* | 172764 | 172801 | -3 | 1.25E-07 | *_* | *orf185a* |
| R1435 | 38 | F | *Z. caespitosa* | 68354 | 68391 | *Z. caespitosa* | 172764 | 172801 | -3 | 1.25E-07 | *_* | *orf185a* |
| R1436 | 38 | F | *Z. caespitosa* | 24898 | 24935 | *Z. caespitosa* | 172764 | 172801 | -3 | 1.25E-07 | *_* | *orf185a* |
| R1437 | 38 | F | *Z. caespitosa* | 95628 | 95665 | *Z. caespitosa* | 172764 | 172801 | -3 | 1.25E-07 | *_* | *orf185a* |
| R1438 | 38 | F | *Z. caespitosa* | 172764 | 172801 | *Z. caespitosa* | 172800 | 172837 | -3 | 1.25E-07 | *orf185a* | *orf185a* |
| R1439 | 38 | F | *Z. caespitosa* | 138658 | 138695 | *Z. caespitosa* | 172764 | 172801 | -3 | 1.25E-07 | *_* | *orf185a* |
| R1440 | 38 | F | *Z. caespitosa* | 131400 | 131437 | *Z. caespitosa* | 182473 | 182510 | -3 | 1.25E-07 | *orf131b* | *orf117c* |
| R1441 | 38 | F | *Z. caespitosa* | 57399 | 57436 | *Z. caespitosa* | 74464 | 74501 | -3 | 1.25E-07 | *rrn18;orf152a* | *_* |
| R1442 | 38 | F | *Z. caespitosa* | 52831 | 52868 | *Z. caespitosa* | 75631 | 75668 | -3 | 1.25E-07 | *orf172a* | *_* |
| R1443 | 38 | F | *Z. caespitosa* | 23229 | 23266 | *Z. caespitosa* | 149185 | 149222 | 0 | 5.50E-13 | *_* | *_* |
| R1444 | 38 | F | *Z. caespitosa* | 93959 | 93996 | *Z. caespitosa* | 149185 | 149222 | 0 | 5.50E-13 | *_* | *_* |
| R1445 | 38 | F | *Z. caespitosa* | 15634 | 15671 | *Z. caespitosa* | 68316 | 68353 | -3 | 1.25E-07 | *_* | *_* |
| R1446 | 38 | F | *Z. caespitosa* | 48643 | 48680 | *Z. caespitosa* | 77846 | 77883 | -3 | 1.25E-07 | *_* | *_* |
| R1447 | 38 | F | *Z. caespitosa* | 181447 | 181484 | *Z. caespitosa* | 181465 | 181502 | -3 | 1.25E-07 | *orf103d* | *orf103d* |
| R1448 | 38 | F | *Z. caespitosa* | 1526 | 1563 | *Z. caespitosa* | 186514 | 186551 | -1 | 6.27E-11 | *_* | *orf100b* |
| R1449 | 38 | F | *Z. caespitosa* | 43474 | 43511 | *Z. caespitosa* | 186514 | 186551 | -1 | 6.27E-11 | *_* | *orf100b* |
| R1450 | 38 | F | *Z. caespitosa* | 46048 | 46085 | *Z. caespitosa* | 186514 | 186551 | -1 | 6.27E-11 | *_* | *orf100b* |
| R1451 | 38 | F | *Z. caespitosa* | 4102 | 4139 | *Z. caespitosa* | 186514 | 186551 | -1 | 6.27E-11 | *_* | *orf100b* |
| R1452 | 38 | F | *Z. caespitosa* | 83556 | 83593 | *Z. caespitosa* | 186514 | 186551 | -1 | 6.27E-11 | *_* | *orf100b* |
| R1453 | 38 | F | *Z. caespitosa* | 177624 | 177661 | *Z. caespitosa* | 186514 | 186551 | -1 | 6.27E-11 | *_* | *orf100b* |
| R1454 | 38 | F | *Z. caespitosa* | 180677 | 180714 | *Z. caespitosa* | 186514 | 186551 | -1 | 6.27E-11 | *_* | *orf100b* |
| R1455 | 38 | F | *Z. caespitosa* | 75664 | 75701 | *Z. caespitosa* | 180628 | 180665 | 0 | 5.50E-13 | *_* | *_* |
| R1456 | 38 | F | *Z. caespitosa* | 57647 | 57684 | *Z. caespitosa* | 180628 | 180665 | 0 | 5.50E-13 | *orf117a* | *_* |
| R1457 | 38 | F | *Z. caespitosa* | 34946 | 34983 | *Z. caespitosa* | 75867 | 75904 | -3 | 1.25E-07 | *_* | *_* |
| R1458 | 38 | F | *Z. caespitosa* | 45999 | 46036 | *Z. caespitosa* | 180658 | 180695 | 0 | 5.50E-13 | *_* | *_* |
| R1459 | 38 | F | *Z. caespitosa* | 4053 | 4090 | *Z. caespitosa* | 180658 | 180695 | 0 | 5.50E-13 | *_* | *_* |
| R1460 | 38 | F | *Z. caespitosa* | 108693 | 108730 | *Z. caespitosa* | 156581 | 156618 | 0 | 5.50E-13 | *orf161a* | *_* |
| R1461 | 38 | F | *Z. caespitosa* | 99301 | 99338 | *Z. caespitosa* | 150985 | 151022 | -3 | 1.25E-07 | *orf113c-2* | *orf132a* |
| R1462 | 38 | F | *Z. caespitosa* | 37718 | 37755 | *Z. caespitosa* | 51825 | 51862 | -3 | 1.25E-07 | *_* | *orf106b;ccmFn* |
| R1463 | 38 | F | *Z. caespitosa* | 9852 | 9889 | *Z. caespitosa* | 9978 | 10015 | -3 | 1.25E-07 | *orf397a* | *orf397a* |
| R1464 | 38 | P | *Z. caespitosa* | 186514 | 186551 | *Z. caespitosa* | 188209 | 188246 | -1 | 6.27E-11 | *orf100b* | *_* |
| R1465 | 38 | P | *Z. caespitosa* | 48643 | 48680 | *Z. caespitosa* | 184162 | 184199 | -3 | 1.25E-07 | *_* | *orf130b* |
| R1466 | 38 | P | *Z. caespitosa* | 172780 | 172817 | *Z. caespitosa* | 184102 | 184139 | 0 | 5.50E-13 | *orf185a* | *orf130b* |
| R1467 | 38 | P | *Z. caespitosa* | 138638 | 138675 | *Z. caespitosa* | 184102 | 184139 | 0 | 5.50E-13 | *_* | *orf130b* |
| R1468 | 38 | P | *Z. caespitosa* | 48643 | 48680 | *Z. caespitosa* | 181600 | 181637 | -3 | 1.25E-07 | *_* | *orf103d* |
| R1469 | 38 | P | *Z. caespitosa* | 172832 | 172869 | *Z. caespitosa* | 180628 | 180665 | 0 | 5.50E-13 | *orf185a* | *_* |
| R1470 | 38 | P | *Z. caespitosa* | 48643 | 48680 | *Z. caespitosa* | 177935 | 177972 | -3 | 1.25E-07 | *_* | *_* |
| R1471 | 38 | P | *Z. caespitosa* | 72176 | 72213 | *Z. caespitosa* | 177864 | 177901 | -2 | 3.48E-09 | *_* | *_* |
| R1472 | 38 | P | *Z. caespitosa* | 23229 | 23266 | *Z. caespitosa* | 175083 | 175120 | -2 | 3.48E-09 | *_* | *_* |
| R1473 | 38 | P | *Z. caespitosa* | 93959 | 93996 | *Z. caespitosa* | 175083 | 175120 | -2 | 3.48E-09 | *_* | *_* |
| R1474 | 38 | P | *Z. caespitosa* | 116133 | 116170 | *Z. caespitosa* | 172794 | 172831 | -2 | 3.48E-09 | *trnE-UUC* | *orf185a* |
| R1475 | 38 | P | *Z. caespitosa* | 57679 | 57716 | *Z. caespitosa* | 172764 | 172801 | -3 | 1.25E-07 | *orf117a* | *orf185a* |
| R1476 | 38 | P | *Z. caespitosa* | 116090 | 116127 | *Z. caespitosa* | 172764 | 172801 | -3 | 1.25E-07 | *_* | *orf185a* |
| R1477 | 38 | P | *Z. caespitosa* | 51674 | 51711 | *Z. caespitosa* | 172640 | 172677 | 0 | 5.50E-13 | *orf106b;ccmFn* | *orf185a* |
| R1478 | 38 | P | *Z. caespitosa* | 34946 | 34983 | *Z. caespitosa* | 172593 | 172630 | -3 | 1.25E-07 | *_* | *orf185a* |
| R1479 | 38 | P | *Z. caespitosa* | 112418 | 112455 | *Z. caespitosa* | 150985 | 151022 | -3 | 1.25E-07 | *orf113c-1* | *orf132a* |
| R1480 | 38 | P | *Z. caespitosa* | 117759 | 117796 | *Z. caespitosa* | 149185 | 149222 | 0 | 5.50E-13 | *_* | *_* |
| R1481 | 38 | P | *Z. caespitosa* | 116133 | 116170 | *Z. caespitosa* | 138652 | 138689 | -2 | 3.48E-09 | *trnE-UUC* | *_* |
| R1482 | 38 | P | *Z. caespitosa* | 48643 | 48680 | *Z. caespitosa* | 136895 | 136932 | -3 | 1.25E-07 | *_* | *_* |
| R1483 | 38 | P | *Z. caespitosa* | 28772 | 28809 | *Z. caespitosa* | 131300 | 131337 | -3 | 1.25E-07 | *_* | *orf131b* |
| R1484 | 38 | P | *Z. caespitosa* | 68348 | 68385 | *Z. caespitosa* | 116133 | 116170 | -2 | 3.48E-09 | *_* | *trnE-UUC* |
| R1485 | 38 | P | *Z. caespitosa* | 59173 | 59210 | *Z. caespitosa* | 116079 | 116116 | -3 | 1.25E-07 | *_* | *_* |
| R1486 | 38 | P | *Z. caespitosa* | 15987 | 16024 | *Z. caespitosa* | 115983 | 116020 | 0 | 5.50E-13 | *orf284a* | *_* |
| R1487 | 38 | P | *Z. caespitosa* | 7620 | 7657 | *Z. caespitosa* | 115983 | 116020 | 0 | 5.50E-13 | *_* | *_* |
| R1488 | 38 | P | *Z. caespitosa* | 48412 | 48449 | *Z. caespitosa* | 108694 | 108731 | -1 | 6.27E-11 | *_* | *orf161a* |
| R1489 | 38 | P | *Z. caespitosa* | 48643 | 48680 | *Z. caespitosa* | 83956 | 83993 | -3 | 1.25E-07 | *_* | *_* |
| R1490 | 38 | P | *Z. caespitosa* | 72176 | 72213 | *Z. caespitosa* | 83885 | 83922 | -2 | 3.48E-09 | *_* | *_* |
| R1491 | 38 | P | *Z. caespitosa* | 75820 | 75857 | *Z. caespitosa* | 79524 | 79561 | 0 | 5.50E-13 | *_* | *_* |
| R1492 | 38 | P | *Z. caespitosa* | 71946 | 71983 | *Z. caespitosa* | 75820 | 75857 | 0 | 5.50E-13 | *_* | *_* |
| R1493 | 38 | P | *Z. caespitosa* | 59173 | 59210 | *Z. caespitosa* | 75685 | 75722 | 0 | 5.50E-13 | *_* | *_* |
| R1494 | 38 | P | *Z. caespitosa* | 57668 | 57705 | *Z. caespitosa* | 59173 | 59210 | -3 | 1.25E-07 | *orf117a* | *_* |
| R1495 | 38 | P | *Z. caespitosa* | 48643 | 48680 | *Z. caespitosa* | 51709 | 51746 | -2 | 3.48E-09 | *_* | *orf106b;ccmFn* |
| R1496 | 38 | P | *Z. caespitosa* | 37746 | 37783 | *Z. caespitosa* | 48412 | 48449 | -1 | 6.27E-11 | *_* | *_* |
| R1497 | 38 | P | *Z. caespitosa* | 28882 | 28919 | *Z. caespitosa* | 41305 | 41342 | -3 | 1.25E-07 | *_* | *orf120b* |
| R1498 | 37 | F | *Z. caespitosa* | 101647 | 101683 | *Z. caespitosa* | 138623 | 138659 | 0 | 2.20E-12 | *_* | *_* |
| R1499 | 37 | F | *Z. caespitosa* | 138623 | 138659 | *Z. caespitosa* | 162664 | 162700 | -3 | 4.62E-07 | *_* | *_* |
| R1500 | 37 | F | *Z. caespitosa* | 68355 | 68391 | *Z. caespitosa* | 138623 | 138659 | -3 | 4.62E-07 | *_* | *_* |
| R1501 | 37 | F | *Z. caespitosa* | 24899 | 24935 | *Z. caespitosa* | 138623 | 138659 | -3 | 4.62E-07 | *_* | *_* |
| R1502 | 37 | F | *Z. caespitosa* | 95629 | 95665 | *Z. caespitosa* | 138623 | 138659 | -3 | 4.62E-07 | *_* | *_* |
| R1503 | 37 | F | *Z. caespitosa* | 138623 | 138659 | *Z. caespitosa* | 172801 | 172837 | -3 | 4.62E-07 | *_* | *orf185a* |
| R1504 | 37 | F | *Z. caespitosa* | 138623 | 138659 | *Z. caespitosa* | 138659 | 138695 | -3 | 4.62E-07 | *_* | *_* |
| R1505 | 37 | F | *Z. caespitosa* | 117073 | 117109 | *Z. caespitosa* | 149738 | 149774 | -1 | 2.44E-10 | *_* | *_* |
| R1506 | 37 | F | *Z. caespitosa* | 125328 | 125364 | *Z. caespitosa* | 159901 | 159937 | -2 | 1.32E-08 | *_* | *rrn26* |
| R1507 | 37 | F | *Z. caespitosa* | 23211 | 23247 | *Z. caespitosa* | 85086 | 85122 | 0 | 2.20E-12 | *_* | *_* |
| R1508 | 37 | F | *Z. caespitosa* | 85086 | 85122 | *Z. caespitosa* | 93941 | 93977 | 0 | 2.20E-12 | *_* | *_* |
| R1509 | 37 | F | *Z. caespitosa* | 140157 | 140193 | *Z. caespitosa* | 188177 | 188213 | 0 | 2.20E-12 | *matR* | *_* |
| R1510 | 37 | F | *Z. caespitosa* | 22154 | 22190 | *Z. caespitosa* | 55106 | 55142 | -3 | 4.62E-07 | *_* | *_* |
| R1511 | 37 | F | *Z. caespitosa* | 53009 | 53045 | *Z. caespitosa* | 53044 | 53080 | 0 | 2.20E-12 | *_* | *_* |
| R1512 | 37 | F | *Z. caespitosa* | 75776 | 75812 | *Z. caespitosa* | 108715 | 108751 | 0 | 2.20E-12 | *_* | *orf161a* |
| R1513 | 37 | F | *Z. caespitosa* | 53107 | 53143 | *Z. caespitosa* | 68548 | 68584 | 0 | 2.20E-12 | *_* | *_* |
| R1514 | 37 | F | *Z. caespitosa* | 37878 | 37914 | *Z. caespitosa* | 53107 | 53143 | 0 | 2.20E-12 | *_* | *_* |
| R1515 | 37 | F | *Z. caespitosa* | 83531 | 83567 | *Z. caespitosa* | 84257 | 84293 | -3 | 4.62E-07 | *_* | *_* |
| R1516 | 37 | F | *Z. caespitosa* | 53147 | 53183 | *Z. caespitosa* | 83531 | 83567 | -3 | 4.62E-07 | *_* | *_* |
| R1517 | 37 | F | *Z. caespitosa* | 83531 | 83567 | *Z. caespitosa* | 135132 | 135168 | -3 | 4.62E-07 | *_* | *_* |
| R1518 | 37 | F | *Z. caespitosa* | 84257 | 84293 | *Z. caespitosa* | 177599 | 177635 | -3 | 4.62E-07 | *_* | *_* |
| R1519 | 37 | F | *Z. caespitosa* | 53147 | 53183 | *Z. caespitosa* | 177599 | 177635 | -3 | 4.62E-07 | *_* | *_* |
| R1520 | 37 | F | *Z. caespitosa* | 135132 | 135168 | *Z. caespitosa* | 177599 | 177635 | -3 | 4.62E-07 | *_* | *_* |
| R1521 | 37 | F | *Z. caespitosa* | 50022 | 50058 | *Z. caespitosa* | 53045 | 53081 | -3 | 4.62E-07 | *_* | *_* |
| R1522 | 37 | F | *Z. caespitosa* | 33018 | 33054 | *Z. caespitosa* | 100040 | 100076 | 0 | 2.20E-12 | *_* | *_* |
| R1523 | 37 | F | *Z. caespitosa* | 33018 | 33054 | *Z. caespitosa* | 75982 | 76018 | -2 | 1.32E-08 | *_* | *_* |
| R1524 | 37 | F | *Z. caespitosa* | 87440 | 87476 | *Z. caespitosa* | 172963 | 172999 | -3 | 4.62E-07 | *orf122a* | *orf185a* |
| R1525 | 37 | F | *Z. caespitosa* | 48661 | 48697 | *Z. caespitosa* | 87440 | 87476 | -3 | 4.62E-07 | *_* | *orf122a* |
| R1526 | 37 | F | *Z. caespitosa* | 16186 | 16222 | *Z. caespitosa* | 101789 | 101825 | 0 | 2.20E-12 | *orf284a* | *_* |
| R1527 | 37 | F | *Z. caespitosa* | 27070 | 27106 | *Z. caespitosa* | 85280 | 85316 | -3 | 4.62E-07 | *_* | *orf100a* |
| R1528 | 37 | F | *Z. caespitosa* | 27070 | 27106 | *Z. caespitosa* | 77970 | 78006 | -3 | 4.62E-07 | *_* | *_* |
| R1529 | 37 | F | *Z. caespitosa* | 25549 | 25585 | *Z. caespitosa* | 25723 | 25759 | 0 | 2.20E-12 | *orf340a* | *orf340a* |
| R1530 | 37 | F | *Z. caespitosa* | 25723 | 25759 | *Z. caespitosa* | 96279 | 96315 | 0 | 2.20E-12 | *orf340a* | *orf540a-2* |
| R1531 | 37 | F | *Z. caespitosa* | 25549 | 25585 | *Z. caespitosa* | 96453 | 96489 | 0 | 2.20E-12 | *orf340a* | *orf540a-2* |
| R1532 | 37 | F | *Z. caespitosa* | 96279 | 96315 | *Z. caespitosa* | 96453 | 96489 | 0 | 2.20E-12 | *orf540a-2* | *orf540a-2* |
| R1533 | 37 | F | *Z. caespitosa* | 74579 | 74615 | *Z. caespitosa* | 172642 | 172678 | -1 | 2.44E-10 | *_* | *orf185a* |
| R1534 | 37 | F | *Z. caespitosa* | 16081 | 16117 | *Z. caespitosa* | 172851 | 172887 | 0 | 2.20E-12 | *orf284a* | *orf185a* |
| R1535 | 37 | F | *Z. caespitosa* | 1567 | 1603 | *Z. caespitosa* | 137033 | 137069 | -3 | 4.62E-07 | *_* | *_* |
| R1536 | 37 | F | *Z. caespitosa* | 43515 | 43551 | *Z. caespitosa* | 137033 | 137069 | -3 | 4.62E-07 | *_* | *_* |
| R1537 | 37 | F | *Z. caespitosa* | 46119 | 46155 | *Z. caespitosa* | 137033 | 137069 | -3 | 4.62E-07 | *_* | *_* |
| R1538 | 37 | F | *Z. caespitosa* | 46089 | 46125 | *Z. caespitosa* | 137033 | 137069 | -2 | 1.32E-08 | *_* | *_* |
| R1539 | 37 | F | *Z. caespitosa* | 137033 | 137069 | *Z. caespitosa* | 177694 | 177730 | -2 | 1.32E-08 | *_* | *_* |
| R1540 | 37 | F | *Z. caespitosa* | 137033 | 137069 | *Z. caespitosa* | 180747 | 180783 | -2 | 1.32E-08 | *_* | *_* |
| R1541 | 37 | F | *Z. caespitosa* | 4202 | 4238 | *Z. caespitosa* | 137033 | 137069 | -2 | 1.32E-08 | *_* | *_* |
| R1542 | 37 | F | *Z. caespitosa* | 83656 | 83692 | *Z. caespitosa* | 137033 | 137069 | -2 | 1.32E-08 | *_* | *_* |
| R1543 | 37 | F | *Z. caespitosa* | 4291 | 4327 | *Z. caespitosa* | 137033 | 137069 | -2 | 1.32E-08 | *_* | *_* |
| R1544 | 37 | F | *Z. caespitosa* | 137033 | 137069 | *Z. caespitosa* | 177724 | 177760 | -2 | 1.32E-08 | *_* | *_* |
| R1545 | 37 | F | *Z. caespitosa* | 83745 | 83781 | *Z. caespitosa* | 137033 | 137069 | -2 | 1.32E-08 | *_* | *_* |
| R1546 | 37 | F | *Z. caespitosa* | 4380 | 4416 | *Z. caespitosa* | 137033 | 137069 | -2 | 1.32E-08 | *_* | *_* |
| R1547 | 37 | F | *Z. caespitosa* | 137033 | 137069 | *Z. caespitosa* | 177813 | 177849 | -2 | 1.32E-08 | *_* | *_* |
| R1548 | 37 | F | *Z. caespitosa* | 83834 | 83870 | *Z. caespitosa* | 137033 | 137069 | -2 | 1.32E-08 | *_* | *_* |
| R1549 | 37 | F | *Z. caespitosa* | 4469 | 4505 | *Z. caespitosa* | 137033 | 137069 | -2 | 1.32E-08 | *_* | *_* |
| R1550 | 37 | F | *Z. caespitosa* | 137033 | 137069 | *Z. caespitosa* | 180777 | 180813 | -2 | 1.32E-08 | *_* | *_* |
| R1551 | 37 | F | *Z. caespitosa* | 4143 | 4179 | *Z. caespitosa* | 137033 | 137069 | -2 | 1.32E-08 | *_* | *_* |
| R1552 | 37 | F | *Z. caespitosa* | 83597 | 83633 | *Z. caespitosa* | 137033 | 137069 | -2 | 1.32E-08 | *_* | *_* |
| R1553 | 37 | F | *Z. caespitosa* | 4232 | 4268 | *Z. caespitosa* | 137033 | 137069 | -2 | 1.32E-08 | *_* | *_* |
| R1554 | 37 | F | *Z. caespitosa* | 83686 | 83722 | *Z. caespitosa* | 137033 | 137069 | -2 | 1.32E-08 | *_* | *_* |
| R1555 | 37 | F | *Z. caespitosa* | 4321 | 4357 | *Z. caespitosa* | 137033 | 137069 | -2 | 1.32E-08 | *_* | *_* |
| R1556 | 37 | F | *Z. caespitosa* | 137033 | 137069 | *Z. caespitosa* | 177754 | 177790 | -2 | 1.32E-08 | *_* | *_* |
| R1557 | 37 | F | *Z. caespitosa* | 83775 | 83811 | *Z. caespitosa* | 137033 | 137069 | -2 | 1.32E-08 | *_* | *_* |
| R1558 | 37 | F | *Z. caespitosa* | 4410 | 4446 | *Z. caespitosa* | 137033 | 137069 | -2 | 1.32E-08 | *_* | *_* |
| R1559 | 37 | F | *Z. caespitosa* | 162910 | 162946 | *Z. caespitosa* | 188235 | 188271 | -3 | 4.62E-07 | *_* | *_* |
| R1560 | 37 | F | *Z. caespitosa* | 72228 | 72264 | *Z. caespitosa* | 188235 | 188271 | -3 | 4.62E-07 | *_* | *_* |
| R1561 | 37 | F | *Z. caespitosa* | 25635 | 25671 | *Z. caespitosa* | 176222 | 176258 | -2 | 1.32E-08 | *orf340a* | *orf110b* |
| R1562 | 37 | F | *Z. caespitosa* | 96365 | 96401 | *Z. caespitosa* | 176222 | 176258 | -2 | 1.32E-08 | *orf540a-2* | *orf110b* |
| R1563 | 37 | F | *Z. caespitosa* | 172594 | 172630 | *Z. caespitosa* | 172949 | 172985 | -3 | 4.62E-07 | *orf185a* | *orf185a* |
| R1564 | 37 | F | *Z. caespitosa* | 41300 | 41336 | *Z. caespitosa* | 151828 | 151864 | -3 | 4.62E-07 | *orf120b* | *orf268a* |
| R1565 | 37 | F | *Z. caespitosa* | 137052 | 137088 | *Z. caespitosa* | 177832 | 177868 | -3 | 4.62E-07 | *_* | *_* |
| R1566 | 37 | F | *Z. caespitosa* | 83853 | 83889 | *Z. caespitosa* | 137052 | 137088 | -3 | 4.62E-07 | *_* | *_* |
| R1567 | 37 | F | *Z. caespitosa* | 4488 | 4524 | *Z. caespitosa* | 137052 | 137088 | -3 | 4.62E-07 | *_* | *_* |
| R1568 | 37 | F | *Z. caespitosa* | 137052 | 137088 | *Z. caespitosa* | 180796 | 180832 | -3 | 4.62E-07 | *_* | *_* |
| R1569 | 37 | F | *Z. caespitosa* | 4162 | 4198 | *Z. caespitosa* | 137052 | 137088 | -3 | 4.62E-07 | *_* | *_* |
| R1570 | 37 | F | *Z. caespitosa* | 83616 | 83652 | *Z. caespitosa* | 137052 | 137088 | -3 | 4.62E-07 | *_* | *_* |
| R1571 | 37 | F | *Z. caespitosa* | 4251 | 4287 | *Z. caespitosa* | 137052 | 137088 | -3 | 4.62E-07 | *_* | *_* |
| R1572 | 37 | F | *Z. caespitosa* | 83705 | 83741 | *Z. caespitosa* | 137052 | 137088 | -3 | 4.62E-07 | *_* | *_* |
| R1573 | 37 | F | *Z. caespitosa* | 4340 | 4376 | *Z. caespitosa* | 137052 | 137088 | -3 | 4.62E-07 | *_* | *_* |
| R1574 | 37 | F | *Z. caespitosa* | 137052 | 137088 | *Z. caespitosa* | 177773 | 177809 | -3 | 4.62E-07 | *_* | *_* |
| R1575 | 37 | F | *Z. caespitosa* | 83794 | 83830 | *Z. caespitosa* | 137052 | 137088 | -3 | 4.62E-07 | *_* | *_* |
| R1576 | 37 | F | *Z. caespitosa* | 4429 | 4465 | *Z. caespitosa* | 137052 | 137088 | -3 | 4.62E-07 | *_* | *_* |
| R1577 | 37 | F | *Z. caespitosa* | 34245 | 34281 | *Z. caespitosa* | 75867 | 75903 | -3 | 4.62E-07 | *_* | *_* |
| R1578 | 37 | F | *Z. caespitosa* | 55120 | 55156 | *Z. caespitosa* | 75867 | 75903 | -3 | 4.62E-07 | *_* | *_* |
| R1579 | 37 | F | *Z. caespitosa* | 7997 | 8033 | *Z. caespitosa* | 14228 | 14264 | -3 | 4.62E-07 | *_* | *_* |
| R1580 | 37 | F | *Z. caespitosa* | 10146 | 10182 | *Z. caespitosa* | 10209 | 10245 | -3 | 4.62E-07 | *orf397a* | *_* |
| R1581 | 37 | F | *Z. caespitosa* | 10020 | 10056 | *Z. caespitosa* | 10209 | 10245 | -3 | 4.62E-07 | *orf397a* | *_* |
| R1582 | 37 | F | *Z. caespitosa* | 115266 | 115302 | *Z. caespitosa* | 115440 | 115476 | 0 | 2.20E-12 | *orf540a-1* | *orf540a-1* |
| R1583 | 37 | F | *Z. caespitosa* | 135052 | 135088 | *Z. caespitosa* | 177557 | 177593 | -2 | 1.32E-08 | *_* | *_* |
| R1584 | 37 | F | *Z. caespitosa* | 61320 | 61356 | *Z. caespitosa* | 135149 | 135185 | -2 | 1.32E-08 | *orf189a* | *_* |
| R1585 | 37 | P | *Z. caespitosa* | 84257 | 84293 | *Z. caespitosa* | 188235 | 188271 | -3 | 4.62E-07 | *_* | *_* |
| R1586 | 37 | P | *Z. caespitosa* | 53147 | 53183 | *Z. caespitosa* | 188235 | 188271 | -3 | 4.62E-07 | *_* | *_* |
| R1587 | 37 | P | *Z. caespitosa* | 135132 | 135168 | *Z. caespitosa* | 188235 | 188271 | -3 | 4.62E-07 | *_* | *_* |
| R1588 | 37 | P | *Z. caespitosa* | 33034 | 33070 | *Z. caespitosa* | 184962 | 184998 | 0 | 2.20E-12 | *_* | *_* |
| R1589 | 37 | P | *Z. caespitosa* | 53064 | 53100 | *Z. caespitosa* | 184962 | 184998 | 0 | 2.20E-12 | *_* | *_* |
| R1590 | 37 | P | *Z. caespitosa* | 22176 | 22212 | *Z. caespitosa* | 184962 | 184998 | 0 | 2.20E-12 | *_* | *_* |
| R1591 | 37 | P | *Z. caespitosa* | 164703 | 164739 | *Z. caespitosa* | 180796 | 180832 | -3 | 4.62E-07 | *_* | *_* |
| R1592 | 37 | P | *Z. caespitosa* | 164722 | 164758 | *Z. caespitosa* | 180777 | 180813 | -2 | 1.32E-08 | *_* | *_* |
| R1593 | 37 | P | *Z. caespitosa* | 139959 | 139995 | *Z. caespitosa* | 180777 | 180813 | -2 | 1.32E-08 | *matR* | *_* |
| R1594 | 37 | P | *Z. caespitosa* | 77656 | 77692 | *Z. caespitosa* | 180770 | 180806 | -3 | 4.62E-07 | *_* | *_* |
| R1595 | 37 | P | *Z. caespitosa* | 164722 | 164758 | *Z. caespitosa* | 180747 | 180783 | -2 | 1.32E-08 | *_* | *_* |
| R1596 | 37 | P | *Z. caespitosa* | 139959 | 139995 | *Z. caespitosa* | 180747 | 180783 | -2 | 1.32E-08 | *matR* | *_* |
| R1597 | 37 | P | *Z. caespitosa* | 77656 | 77692 | *Z. caespitosa* | 180740 | 180776 | -3 | 4.62E-07 | *_* | *_* |
| R1598 | 37 | P | *Z. caespitosa* | 164660 | 164696 | *Z. caespitosa* | 180718 | 180754 | -2 | 1.32E-08 | *_* | *_* |
| R1599 | 37 | P | *Z. caespitosa* | 52591 | 52627 | *Z. caespitosa* | 179992 | 180028 | -1 | 2.44E-10 | *orf172a* | *_* |
| R1600 | 37 | P | *Z. caespitosa* | 164703 | 164739 | *Z. caespitosa* | 177832 | 177868 | -3 | 4.62E-07 | *_* | *_* |
| R1601 | 37 | P | *Z. caespitosa* | 164722 | 164758 | *Z. caespitosa* | 177813 | 177849 | -2 | 1.32E-08 | *_* | *_* |
| R1602 | 37 | P | *Z. caespitosa* | 139959 | 139995 | *Z. caespitosa* | 177813 | 177849 | -2 | 1.32E-08 | *matR* | *_* |
| R1603 | 37 | P | *Z. caespitosa* | 77656 | 77692 | *Z. caespitosa* | 177806 | 177842 | -3 | 4.62E-07 | *_* | *_* |
| R1604 | 37 | P | *Z. caespitosa* | 164660 | 164696 | *Z. caespitosa* | 177784 | 177820 | -2 | 1.32E-08 | *_* | *_* |
| R1605 | 37 | P | *Z. caespitosa* | 164703 | 164739 | *Z. caespitosa* | 177773 | 177809 | -3 | 4.62E-07 | *_* | *_* |
| R1606 | 37 | P | *Z. caespitosa* | 164722 | 164758 | *Z. caespitosa* | 177754 | 177790 | -2 | 1.32E-08 | *_* | *_* |
| R1607 | 37 | P | *Z. caespitosa* | 139959 | 139995 | *Z. caespitosa* | 177754 | 177790 | -2 | 1.32E-08 | *matR* | *_* |
| R1608 | 37 | P | *Z. caespitosa* | 77656 | 77692 | *Z. caespitosa* | 177747 | 177783 | -3 | 4.62E-07 | *_* | *_* |
| R1609 | 37 | P | *Z. caespitosa* | 164722 | 164758 | *Z. caespitosa* | 177724 | 177760 | -2 | 1.32E-08 | *_* | *_* |
| R1610 | 37 | P | *Z. caespitosa* | 139959 | 139995 | *Z. caespitosa* | 177724 | 177760 | -2 | 1.32E-08 | *matR* | *_* |
| R1611 | 37 | P | *Z. caespitosa* | 77656 | 77692 | *Z. caespitosa* | 177717 | 177753 | -3 | 4.62E-07 | *_* | *_* |
| R1612 | 37 | P | *Z. caespitosa* | 164722 | 164758 | *Z. caespitosa* | 177694 | 177730 | -2 | 1.32E-08 | *_* | *_* |
| R1613 | 37 | P | *Z. caespitosa* | 139959 | 139995 | *Z. caespitosa* | 177694 | 177730 | -2 | 1.32E-08 | *matR* | *_* |
| R1614 | 37 | P | *Z. caespitosa* | 77656 | 77692 | *Z. caespitosa* | 177687 | 177723 | -3 | 4.62E-07 | *_* | *_* |
| R1615 | 37 | P | *Z. caespitosa* | 164660 | 164696 | *Z. caespitosa* | 177665 | 177701 | -2 | 1.32E-08 | *_* | *_* |
| R1616 | 37 | P | *Z. caespitosa* | 72228 | 72264 | *Z. caespitosa* | 177599 | 177635 | -3 | 4.62E-07 | *_* | *_* |
| R1617 | 37 | P | *Z. caespitosa* | 162910 | 162946 | *Z. caespitosa* | 177599 | 177635 | -3 | 4.62E-07 | *_* | *_* |
| R1618 | 37 | P | *Z. caespitosa* | 162990 | 163026 | *Z. caespitosa* | 177557 | 177593 | -2 | 1.32E-08 | *_* | *_* |
| R1619 | 37 | P | *Z. caespitosa* | 79648 | 79684 | *Z. caespitosa* | 177557 | 177593 | -3 | 4.62E-07 | *_* | *_* |
| R1620 | 37 | P | *Z. caespitosa* | 115204 | 115240 | *Z. caespitosa* | 176228 | 176264 | 0 | 2.20E-12 | *orf540a-1* | *orf110b* |
| R1621 | 37 | P | *Z. caespitosa* | 75867 | 75903 | *Z. caespitosa* | 172949 | 172985 | -3 | 4.62E-07 | *_* | *orf185a* |
| R1622 | 37 | P | *Z. caespitosa* | 55106 | 55142 | *Z. caespitosa* | 172888 | 172924 | -3 | 4.62E-07 | *_* | *orf185a* |
| R1623 | 37 | P | *Z. caespitosa* | 108715 | 108751 | *Z. caespitosa* | 172685 | 172721 | 0 | 2.20E-12 | *orf161a* | *orf185a* |
| R1624 | 37 | P | *Z. caespitosa* | 34245 | 34281 | *Z. caespitosa* | 172594 | 172630 | -3 | 4.62E-07 | *_* | *orf185a* |
| R1625 | 37 | P | *Z. caespitosa* | 55120 | 55156 | *Z. caespitosa* | 172594 | 172630 | -3 | 4.62E-07 | *_* | *orf185a* |
| R1626 | 37 | P | *Z. caespitosa* | 1567 | 1603 | *Z. caespitosa* | 164722 | 164758 | -3 | 4.62E-07 | *_* | *_* |
| R1627 | 37 | P | *Z. caespitosa* | 43515 | 43551 | *Z. caespitosa* | 164722 | 164758 | -3 | 4.62E-07 | *_* | *_* |
| R1628 | 37 | P | *Z. caespitosa* | 46119 | 46155 | *Z. caespitosa* | 164722 | 164758 | -3 | 4.62E-07 | *_* | *_* |
| R1629 | 37 | P | *Z. caespitosa* | 46089 | 46125 | *Z. caespitosa* | 164722 | 164758 | -2 | 1.32E-08 | *_* | *_* |
| R1630 | 37 | P | *Z. caespitosa* | 4202 | 4238 | *Z. caespitosa* | 164722 | 164758 | -2 | 1.32E-08 | *_* | *_* |
| R1631 | 37 | P | *Z. caespitosa* | 83656 | 83692 | *Z. caespitosa* | 164722 | 164758 | -2 | 1.32E-08 | *_* | *_* |
| R1632 | 37 | P | *Z. caespitosa* | 4291 | 4327 | *Z. caespitosa* | 164722 | 164758 | -2 | 1.32E-08 | *_* | *_* |
| R1633 | 37 | P | *Z. caespitosa* | 83745 | 83781 | *Z. caespitosa* | 164722 | 164758 | -2 | 1.32E-08 | *_* | *_* |
| R1634 | 37 | P | *Z. caespitosa* | 4380 | 4416 | *Z. caespitosa* | 164722 | 164758 | -2 | 1.32E-08 | *_* | *_* |
| R1635 | 37 | P | *Z. caespitosa* | 83834 | 83870 | *Z. caespitosa* | 164722 | 164758 | -2 | 1.32E-08 | *_* | *_* |
| R1636 | 37 | P | *Z. caespitosa* | 4469 | 4505 | *Z. caespitosa* | 164722 | 164758 | -2 | 1.32E-08 | *_* | *_* |
| R1637 | 37 | P | *Z. caespitosa* | 4143 | 4179 | *Z. caespitosa* | 164722 | 164758 | -2 | 1.32E-08 | *_* | *_* |
| R1638 | 37 | P | *Z. caespitosa* | 83597 | 83633 | *Z. caespitosa* | 164722 | 164758 | -2 | 1.32E-08 | *_* | *_* |
| R1639 | 37 | P | *Z. caespitosa* | 4232 | 4268 | *Z. caespitosa* | 164722 | 164758 | -2 | 1.32E-08 | *_* | *_* |
| R1640 | 37 | P | *Z. caespitosa* | 83686 | 83722 | *Z. caespitosa* | 164722 | 164758 | -2 | 1.32E-08 | *_* | *_* |
| R1641 | 37 | P | *Z. caespitosa* | 4321 | 4357 | *Z. caespitosa* | 164722 | 164758 | -2 | 1.32E-08 | *_* | *_* |
| R1642 | 37 | P | *Z. caespitosa* | 83775 | 83811 | *Z. caespitosa* | 164722 | 164758 | -2 | 1.32E-08 | *_* | *_* |
| R1643 | 37 | P | *Z. caespitosa* | 4410 | 4446 | *Z. caespitosa* | 164722 | 164758 | -2 | 1.32E-08 | *_* | *_* |
| R1644 | 37 | P | *Z. caespitosa* | 83853 | 83889 | *Z. caespitosa* | 164703 | 164739 | -3 | 4.62E-07 | *_* | *_* |
| R1645 | 37 | P | *Z. caespitosa* | 4488 | 4524 | *Z. caespitosa* | 164703 | 164739 | -3 | 4.62E-07 | *_* | *_* |
| R1646 | 37 | P | *Z. caespitosa* | 4162 | 4198 | *Z. caespitosa* | 164703 | 164739 | -3 | 4.62E-07 | *_* | *_* |
| R1647 | 37 | P | *Z. caespitosa* | 83616 | 83652 | *Z. caespitosa* | 164703 | 164739 | -3 | 4.62E-07 | *_* | *_* |
| R1648 | 37 | P | *Z. caespitosa* | 4251 | 4287 | *Z. caespitosa* | 164703 | 164739 | -3 | 4.62E-07 | *_* | *_* |
| R1649 | 37 | P | *Z. caespitosa* | 83705 | 83741 | *Z. caespitosa* | 164703 | 164739 | -3 | 4.62E-07 | *_* | *_* |
| R1650 | 37 | P | *Z. caespitosa* | 4340 | 4376 | *Z. caespitosa* | 164703 | 164739 | -3 | 4.62E-07 | *_* | *_* |
| R1651 | 37 | P | *Z. caespitosa* | 83794 | 83830 | *Z. caespitosa* | 164703 | 164739 | -3 | 4.62E-07 | *_* | *_* |
| R1652 | 37 | P | *Z. caespitosa* | 4429 | 4465 | *Z. caespitosa* | 164703 | 164739 | -3 | 4.62E-07 | *_* | *_* |
| R1653 | 37 | P | *Z. caespitosa* | 4440 | 4476 | *Z. caespitosa* | 164660 | 164696 | -2 | 1.32E-08 | *_* | *_* |
| R1654 | 37 | P | *Z. caespitosa* | 83805 | 83841 | *Z. caespitosa* | 164660 | 164696 | -2 | 1.32E-08 | *_* | *_* |
| R1655 | 37 | P | *Z. caespitosa* | 4351 | 4387 | *Z. caespitosa* | 164660 | 164696 | -2 | 1.32E-08 | *_* | *_* |
| R1656 | 37 | P | *Z. caespitosa* | 83716 | 83752 | *Z. caespitosa* | 164660 | 164696 | -2 | 1.32E-08 | *_* | *_* |
| R1657 | 37 | P | *Z. caespitosa* | 4262 | 4298 | *Z. caespitosa* | 164660 | 164696 | -2 | 1.32E-08 | *_* | *_* |
| R1658 | 37 | P | *Z. caespitosa* | 83627 | 83663 | *Z. caespitosa* | 164660 | 164696 | -2 | 1.32E-08 | *_* | *_* |
| R1659 | 37 | P | *Z. caespitosa* | 4173 | 4209 | *Z. caespitosa* | 164660 | 164696 | -2 | 1.32E-08 | *_* | *_* |
| R1660 | 37 | P | *Z. caespitosa* | 53107 | 53143 | *Z. caespitosa* | 163006 | 163042 | -1 | 2.44E-10 | *_* | *_* |
| R1661 | 37 | P | *Z. caespitosa* | 83531 | 83567 | *Z. caespitosa* | 162910 | 162946 | -3 | 4.62E-07 | *_* | *_* |
| R1662 | 37 | P | *Z. caespitosa* | 61320 | 61356 | *Z. caespitosa* | 162893 | 162929 | -2 | 1.32E-08 | *orf189a* | *_* |
| R1663 | 37 | P | *Z. caespitosa* | 61320 | 61356 | *Z. caespitosa* | 162537 | 162573 | 0 | 2.20E-12 | *orf189a* | *_* |
| R1664 | 37 | P | *Z. caespitosa* | 48413 | 48449 | *Z. caespitosa* | 156582 | 156618 | -1 | 2.44E-10 | *_* | *_* |
| R1665 | 37 | P | *Z. caespitosa* | 94646 | 94682 | *Z. caespitosa* | 149738 | 149774 | -1 | 2.44E-10 | *_* | *_* |
| R1666 | 37 | P | *Z. caespitosa* | 23916 | 23952 | *Z. caespitosa* | 149738 | 149774 | -1 | 2.44E-10 | *_* | *_* |
| R1667 | 37 | P | *Z. caespitosa* | 50909 | 50945 | *Z. caespitosa* | 140219 | 140255 | -3 | 4.62E-07 | *ccmFn* | *matR* |
| R1668 | 37 | P | *Z. caespitosa* | 4410 | 4446 | *Z. caespitosa* | 139959 | 139995 | -2 | 1.32E-08 | *_* | *matR* |
| R1669 | 37 | P | *Z. caespitosa* | 83775 | 83811 | *Z. caespitosa* | 139959 | 139995 | -2 | 1.32E-08 | *_* | *matR* |
| R1670 | 37 | P | *Z. caespitosa* | 4321 | 4357 | *Z. caespitosa* | 139959 | 139995 | -2 | 1.32E-08 | *_* | *matR* |
| R1671 | 37 | P | *Z. caespitosa* | 83686 | 83722 | *Z. caespitosa* | 139959 | 139995 | -2 | 1.32E-08 | *_* | *matR* |
| R1672 | 37 | P | *Z. caespitosa* | 4232 | 4268 | *Z. caespitosa* | 139959 | 139995 | -2 | 1.32E-08 | *_* | *matR* |
| R1673 | 37 | P | *Z. caespitosa* | 83597 | 83633 | *Z. caespitosa* | 139959 | 139995 | -2 | 1.32E-08 | *_* | *matR* |
| R1674 | 37 | P | *Z. caespitosa* | 4143 | 4179 | *Z. caespitosa* | 139959 | 139995 | -2 | 1.32E-08 | *_* | *matR* |
| R1675 | 37 | P | *Z. caespitosa* | 4469 | 4505 | *Z. caespitosa* | 139959 | 139995 | -2 | 1.32E-08 | *_* | *matR* |
| R1676 | 37 | P | *Z. caespitosa* | 83834 | 83870 | *Z. caespitosa* | 139959 | 139995 | -2 | 1.32E-08 | *_* | *matR* |
| R1677 | 37 | P | *Z. caespitosa* | 4380 | 4416 | *Z. caespitosa* | 139959 | 139995 | -2 | 1.32E-08 | *_* | *matR* |
| R1678 | 37 | P | *Z. caespitosa* | 83745 | 83781 | *Z. caespitosa* | 139959 | 139995 | -2 | 1.32E-08 | *_* | *matR* |
| R1679 | 37 | P | *Z. caespitosa* | 4291 | 4327 | *Z. caespitosa* | 139959 | 139995 | -2 | 1.32E-08 | *_* | *matR* |
| R1680 | 37 | P | *Z. caespitosa* | 83656 | 83692 | *Z. caespitosa* | 139959 | 139995 | -2 | 1.32E-08 | *_* | *matR* |
| R1681 | 37 | P | *Z. caespitosa* | 4202 | 4238 | *Z. caespitosa* | 139959 | 139995 | -2 | 1.32E-08 | *_* | *matR* |
| R1682 | 37 | P | *Z. caespitosa* | 46089 | 46125 | *Z. caespitosa* | 139959 | 139995 | -2 | 1.32E-08 | *_* | *matR* |
| R1683 | 37 | P | *Z. caespitosa* | 46119 | 46155 | *Z. caespitosa* | 139959 | 139995 | -1 | 2.44E-10 | *_* | *matR* |
| R1684 | 37 | P | *Z. caespitosa* | 43515 | 43551 | *Z. caespitosa* | 139959 | 139995 | -1 | 2.44E-10 | *_* | *matR* |
| R1685 | 37 | P | *Z. caespitosa* | 1567 | 1603 | *Z. caespitosa* | 139959 | 139995 | -1 | 2.44E-10 | *_* | *matR* |
| R1686 | 37 | P | *Z. caespitosa* | 33017 | 33053 | *Z. caespitosa* | 138939 | 138975 | 0 | 2.20E-12 | *_* | *_* |
| R1687 | 37 | P | *Z. caespitosa* | 75696 | 75732 | *Z. caespitosa* | 138623 | 138659 | 0 | 2.20E-12 | *_* | *_* |
| R1688 | 37 | P | *Z. caespitosa* | 57679 | 57715 | *Z. caespitosa* | 138623 | 138659 | -3 | 4.62E-07 | *orf117a* | *_* |
| R1689 | 37 | P | *Z. caespitosa* | 116090 | 116126 | *Z. caespitosa* | 138623 | 138659 | -3 | 4.62E-07 | *_* | *_* |
| R1690 | 37 | P | *Z. caespitosa* | 55106 | 55142 | *Z. caespitosa* | 118835 | 118871 | -3 | 4.62E-07 | *_* | *_* |
| R1691 | 37 | P | *Z. caespitosa* | 85086 | 85122 | *Z. caespitosa* | 117778 | 117814 | 0 | 2.20E-12 | *_* | *_* |
| R1692 | 37 | P | *Z. caespitosa* | 96453 | 96489 | *Z. caespitosa* | 115440 | 115476 | 0 | 2.20E-12 | *orf540a-2* | *orf540a-1* |
| R1693 | 37 | P | *Z. caespitosa* | 25723 | 25759 | *Z. caespitosa* | 115440 | 115476 | 0 | 2.20E-12 | *orf340a* | *orf540a-1* |
| R1694 | 37 | P | *Z. caespitosa* | 25549 | 25585 | *Z. caespitosa* | 115266 | 115302 | 0 | 2.20E-12 | *orf340a* | *orf540a-1* |
| R1695 | 37 | P | *Z. caespitosa* | 96279 | 96315 | *Z. caespitosa* | 115266 | 115302 | 0 | 2.20E-12 | *orf540a-2* | *orf540a-1* |
| R1696 | 37 | P | *Z. caespitosa* | 33018 | 33054 | *Z. caespitosa* | 111680 | 111716 | 0 | 2.20E-12 | *_* | *_* |
| R1697 | 37 | P | *Z. caespitosa* | 61320 | 61356 | *Z. caespitosa* | 101709 | 101745 | -2 | 1.32E-08 | *orf189a* | *_* |
| R1698 | 37 | P | *Z. caespitosa* | 77656 | 77692 | *Z. caespitosa* | 83827 | 83863 | -3 | 4.62E-07 | *_* | *_* |
| R1699 | 37 | P | *Z. caespitosa* | 77656 | 77692 | *Z. caespitosa* | 83768 | 83804 | -3 | 4.62E-07 | *_* | *_* |
| R1700 | 37 | P | *Z. caespitosa* | 77656 | 77692 | *Z. caespitosa* | 83738 | 83774 | -3 | 4.62E-07 | *_* | *_* |
| R1701 | 37 | P | *Z. caespitosa* | 77656 | 77692 | *Z. caespitosa* | 83679 | 83715 | -3 | 4.62E-07 | *_* | *_* |
| R1702 | 37 | P | *Z. caespitosa* | 77656 | 77692 | *Z. caespitosa* | 83649 | 83685 | -3 | 4.62E-07 | *_* | *_* |
| R1703 | 37 | P | *Z. caespitosa* | 77656 | 77692 | *Z. caespitosa* | 83590 | 83626 | -3 | 4.62E-07 | *_* | *_* |
| R1704 | 37 | P | *Z. caespitosa* | 72228 | 72264 | *Z. caespitosa* | 83531 | 83567 | -3 | 4.62E-07 | *_* | *_* |
| R1705 | 37 | P | *Z. caespitosa* | 53107 | 53143 | *Z. caespitosa* | 79664 | 79700 | 0 | 2.20E-12 | *_* | *_* |
| R1706 | 37 | P | *Z. caespitosa* | 4403 | 4439 | *Z. caespitosa* | 77656 | 77692 | -3 | 4.62E-07 | *_* | *_* |
| R1707 | 37 | P | *Z. caespitosa* | 4314 | 4350 | *Z. caespitosa* | 77656 | 77692 | -3 | 4.62E-07 | *_* | *_* |
| R1708 | 37 | P | *Z. caespitosa* | 4225 | 4261 | *Z. caespitosa* | 77656 | 77692 | -3 | 4.62E-07 | *_* | *_* |
| R1709 | 37 | P | *Z. caespitosa* | 4136 | 4172 | *Z. caespitosa* | 77656 | 77692 | -3 | 4.62E-07 | *_* | *_* |
| R1710 | 37 | P | *Z. caespitosa* | 4462 | 4498 | *Z. caespitosa* | 77656 | 77692 | -3 | 4.62E-07 | *_* | *_* |
| R1711 | 37 | P | *Z. caespitosa* | 4373 | 4409 | *Z. caespitosa* | 77656 | 77692 | -3 | 4.62E-07 | *_* | *_* |
| R1712 | 37 | P | *Z. caespitosa* | 4284 | 4320 | *Z. caespitosa* | 77656 | 77692 | -3 | 4.62E-07 | *_* | *_* |
| R1713 | 37 | P | *Z. caespitosa* | 4195 | 4231 | *Z. caespitosa* | 77656 | 77692 | -3 | 4.62E-07 | *_* | *_* |
| R1714 | 37 | P | *Z. caespitosa* | 46082 | 46118 | *Z. caespitosa* | 77656 | 77692 | -3 | 4.62E-07 | *_* | *_* |
| R1715 | 37 | P | *Z. caespitosa* | 46112 | 46148 | *Z. caespitosa* | 77656 | 77692 | -3 | 4.62E-07 | *_* | *_* |
| R1716 | 37 | P | *Z. caespitosa* | 43508 | 43544 | *Z. caespitosa* | 77656 | 77692 | -3 | 4.62E-07 | *_* | *_* |
| R1717 | 37 | P | *Z. caespitosa* | 1560 | 1596 | *Z. caespitosa* | 77656 | 77692 | -3 | 4.62E-07 | *_* | *_* |
| R1718 | 37 | P | *Z. caespitosa* | 74579 | 74615 | *Z. caespitosa* | 75819 | 75855 | -1 | 2.44E-10 | *_* | *_* |
| R1719 | 37 | P | *Z. caespitosa* | 68384 | 68420 | *Z. caespitosa* | 75667 | 75703 | 0 | 2.20E-12 | *_* | *_* |
| R1720 | 37 | P | *Z. caespitosa* | 16080 | 16116 | *Z. caespitosa* | 50022 | 50058 | -3 | 4.62E-07 | *orf284a* | *_* |
| R1721 | 37 | P | *Z. caespitosa* | 7533 | 7569 | *Z. caespitosa* | 34971 | 35007 | 0 | 2.20E-12 | *_* | *_* |
| R1722 | 37 | P | *Z. caespitosa* | 15900 | 15936 | *Z. caespitosa* | 34971 | 35007 | 0 | 2.20E-12 | *_* | *_* |
| R1723 | 36 | F | *Z. caespitosa* | 107745 | 107780 | *Z. caespitosa* | 169359 | 169394 | 0 | 8.80E-12 | *orf177a* | *_* |
| R1724 | 36 | F | *Z. caespitosa* | 22442 | 22477 | *Z. caespitosa* | 22458 | 22493 | -2 | 4.99E-08 | *_* | *_* |
| R1725 | 36 | F | *Z. caespitosa* | 16243 | 16278 | *Z. caespitosa* | 87351 | 87386 | -3 | 1.70E-06 | *orf284a* | *orf122a* |
| R1726 | 36 | F | *Z. caespitosa* | 16276 | 16311 | *Z. caespitosa* | 87351 | 87386 | -3 | 1.70E-06 | *orf284a* | *orf122a* |
| R1727 | 36 | F | *Z. caespitosa* | 16309 | 16344 | *Z. caespitosa* | 87351 | 87386 | -3 | 1.70E-06 | *orf284a* | *orf122a* |
| R1728 | 36 | F | *Z. caespitosa* | 16342 | 16377 | *Z. caespitosa* | 87351 | 87386 | -3 | 1.70E-06 | *orf284a* | *orf122a* |
| R1729 | 36 | F | *Z. caespitosa* | 16375 | 16410 | *Z. caespitosa* | 87351 | 87386 | -3 | 1.70E-06 | *orf284a* | *orf122a* |
| R1730 | 36 | F | *Z. caespitosa* | 16408 | 16443 | *Z. caespitosa* | 87351 | 87386 | -3 | 1.70E-06 | *orf284a* | *orf122a* |
| R1731 | 36 | F | *Z. caespitosa* | 16441 | 16476 | *Z. caespitosa* | 87351 | 87386 | -3 | 1.70E-06 | *orf284a* | *orf122a* |
| R1732 | 36 | F | *Z. caespitosa* | 16474 | 16509 | *Z. caespitosa* | 87351 | 87386 | -3 | 1.70E-06 | *orf284a* | *orf122a* |
| R1733 | 36 | F | *Z. caespitosa* | 16507 | 16542 | *Z. caespitosa* | 87351 | 87386 | -3 | 1.70E-06 | *orf284a* | *orf122a* |
| R1734 | 36 | F | *Z. caespitosa* | 16540 | 16575 | *Z. caespitosa* | 87351 | 87386 | -3 | 1.70E-06 | *orf284a* | *orf122a* |
| R1735 | 36 | F | *Z. caespitosa* | 16573 | 16608 | *Z. caespitosa* | 87351 | 87386 | -3 | 1.70E-06 | *orf284a* | *orf122a* |
| R1736 | 36 | F | *Z. caespitosa* | 140157 | 140192 | *Z. caespitosa* | 172525 | 172560 | -3 | 1.70E-06 | *matR* | *_* |
| R1737 | 36 | F | *Z. caespitosa* | 172525 | 172560 | *Z. caespitosa* | 188177 | 188212 | -3 | 1.70E-06 | *_* | *_* |
| R1738 | 36 | F | *Z. caespitosa* | 118532 | 118567 | *Z. caespitosa* | 118548 | 118583 | -2 | 4.99E-08 | *_* | *_* |
| R1739 | 36 | F | *Z. caespitosa* | 16082 | 16117 | *Z. caespitosa* | 74746 | 74781 | -3 | 1.70E-06 | *orf284a* | *_* |
| R1740 | 36 | F | *Z. caespitosa* | 74746 | 74781 | *Z. caespitosa* | 172852 | 172887 | -3 | 1.70E-06 | *_* | *orf185a* |
| R1741 | 36 | F | *Z. caespitosa* | 32026 | 32061 | *Z. caespitosa* | 184316 | 184351 | -1 | 9.51E-10 | *orf127a* | *_* |
| R1742 | 36 | F | *Z. caespitosa* | 56135 | 56170 | *Z. caespitosa* | 73480 | 73515 | -3 | 1.70E-06 | *rrn18* | *_* |
| R1743 | 36 | F | *Z. caespitosa* | 55276 | 55311 | *Z. caespitosa* | 156466 | 156501 | 0 | 8.80E-12 | *_* | *_* |
| R1744 | 36 | F | *Z. caespitosa* | 72648 | 72683 | *Z. caespitosa* | 177836 | 177871 | -3 | 1.70E-06 | *_* | *_* |
| R1745 | 36 | F | *Z. caespitosa* | 177836 | 177871 | *Z. caespitosa* | 178002 | 178037 | -3 | 1.70E-06 | *_* | *_* |
| R1746 | 36 | F | *Z. caespitosa* | 72648 | 72683 | *Z. caespitosa* | 83857 | 83892 | -3 | 1.70E-06 | *_* | *_* |
| R1747 | 36 | F | *Z. caespitosa* | 83857 | 83892 | *Z. caespitosa* | 178002 | 178037 | -3 | 1.70E-06 | *_* | *_* |
| R1746 | 36 | F | *Z. caespitosa* | 72648 | 72683 | *Z. caespitosa* | 83857 | 83892 | -3 | 1.70E-06 | *_* | *_* |
| R1747 | 36 | F | *Z. caespitosa* | 83857 | 83892 | *Z. caespitosa* | 178002 | 178037 | -3 | 1.70E-06 | *_* | *_* |
| R1748 | 36 | F | *Z. caespitosa* | 4492 | 4527 | *Z. caespitosa* | 72648 | 72683 | -3 | 1.70E-06 | *_* | *_* |
| R1749 | 36 | F | *Z. caespitosa* | 4492 | 4527 | *Z. caespitosa* | 178002 | 178037 | -3 | 1.70E-06 | *_* | *_* |
| R1750 | 36 | F | *Z. caespitosa* | 72648 | 72683 | *Z. caespitosa* | 180800 | 180835 | -3 | 1.70E-06 | *_* | *_* |
| R1751 | 36 | F | *Z. caespitosa* | 178002 | 178037 | *Z. caespitosa* | 180800 | 180835 | -3 | 1.70E-06 | *_* | *_* |
| R1752 | 36 | F | *Z. caespitosa* | 72648 | 72683 | *Z. caespitosa* | 177658 | 177693 | -3 | 1.70E-06 | *_* | *_* |
| R1753 | 36 | F | *Z. caespitosa* | 177658 | 177693 | *Z. caespitosa* | 178002 | 178037 | -3 | 1.70E-06 | *_* | *_* |
| R1754 | 36 | F | *Z. caespitosa* | 72648 | 72683 | *Z. caespitosa* | 180711 | 180746 | -3 | 1.70E-06 | *_* | *_* |
| R1755 | 36 | F | *Z. caespitosa* | 178002 | 178037 | *Z. caespitosa* | 180711 | 180746 | -3 | 1.70E-06 | *_* | *_* |
| R1756 | 36 | F | *Z. caespitosa* | 4166 | 4201 | *Z. caespitosa* | 72648 | 72683 | -3 | 1.70E-06 | *_* | *_* |
| R1757 | 36 | F | *Z. caespitosa* | 4166 | 4201 | *Z. caespitosa* | 178002 | 178037 | -3 | 1.70E-06 | *_* | *_* |
| R1758 | 36 | F | *Z. caespitosa* | 72648 | 72683 | *Z. caespitosa* | 83620 | 83655 | -3 | 1.70E-06 | *_* | *_* |
| R1759 | 36 | F | *Z. caespitosa* | 83620 | 83655 | *Z. caespitosa* | 178002 | 178037 | -3 | 1.70E-06 | *_* | *_* |
| R1760 | 36 | F | *Z. caespitosa* | 4255 | 4290 | *Z. caespitosa* | 72648 | 72683 | -3 | 1.70E-06 | *_* | *_* |
| R1761 | 36 | F | *Z. caespitosa* | 4255 | 4290 | *Z. caespitosa* | 178002 | 178037 | -3 | 1.70E-06 | *_* | *_* |
| R1762 | 36 | F | *Z. caespitosa* | 72648 | 72683 | *Z. caespitosa* | 83709 | 83744 | -3 | 1.70E-06 | *_* | *_* |
| R1763 | 36 | F | *Z. caespitosa* | 83709 | 83744 | *Z. caespitosa* | 178002 | 178037 | -3 | 1.70E-06 | *_* | *_* |
| R1764 | 36 | F | *Z. caespitosa* | 4344 | 4379 | *Z. caespitosa* | 72648 | 72683 | -3 | 1.70E-06 | *_* | *_* |
| R1765 | 36 | F | *Z. caespitosa* | 4344 | 4379 | *Z. caespitosa* | 178002 | 178037 | -3 | 1.70E-06 | *_* | *_* |
| R1766 | 36 | F | *Z. caespitosa* | 72648 | 72683 | *Z. caespitosa* | 177777 | 177812 | -3 | 1.70E-06 | *_* | *_* |
| R1767 | 36 | F | *Z. caespitosa* | 177777 | 177812 | *Z. caespitosa* | 178002 | 178037 | -3 | 1.70E-06 | *_* | *_* |
| R1768 | 36 | F | *Z. caespitosa* | 72648 | 72683 | *Z. caespitosa* | 83798 | 83833 | -3 | 1.70E-06 | *_* | *_* |
| R1769 | 36 | F | *Z. caespitosa* | 83798 | 83833 | *Z. caespitosa* | 178002 | 178037 | -3 | 1.70E-06 | *_* | *_* |
| R1770 | 36 | F | *Z. caespitosa* | 4433 | 4468 | *Z. caespitosa* | 72648 | 72683 | -3 | 1.70E-06 | *_* | *_* |
| R1771 | 36 | F | *Z. caespitosa* | 4433 | 4468 | *Z. caespitosa* | 178002 | 178037 | -3 | 1.70E-06 | *_* | *_* |
| R1772 | 36 | F | *Z. caespitosa* | 50022 | 50057 | *Z. caespitosa* | 53010 | 53045 | -3 | 1.70E-06 | *_* | *_* |
| R1773 | 36 | F | *Z. caespitosa* | 175159 | 175194 | *Z. caespitosa* | 177843 | 177878 | -3 | 1.70E-06 | *_* | *_* |
| R1774 | 36 | F | *Z. caespitosa* | 83864 | 83899 | *Z. caespitosa* | 175159 | 175194 | -3 | 1.70E-06 | *_* | *_* |
| R1775 | 36 | F | *Z. caespitosa* | 4499 | 4534 | *Z. caespitosa* | 175159 | 175194 | -3 | 1.70E-06 | *_* | *_* |
| R1776 | 36 | F | *Z. caespitosa* | 175159 | 175194 | *Z. caespitosa* | 180807 | 180842 | -3 | 1.70E-06 | *_* | *_* |
| R1777 | 36 | F | *Z. caespitosa* | 33018 | 33053 | *Z. caespitosa* | 115987 | 116022 | 0 | 8.80E-12 | *_* | *_* |
| R1778 | 36 | F | *Z. caespitosa* | 41307 | 41342 | *Z. caespitosa* | 68749 | 68784 | -3 | 1.70E-06 | *orf120b* | *_* |
| R1779 | 36 | F | *Z. caespitosa* | 69323 | 69358 | *Z. caespitosa* | 127138 | 127173 | -2 | 4.99E-08 | *orf118b* | *orf242a* |
| R1780 | 36 | F | *Z. caespitosa* | 9832 | 9867 | *Z. caespitosa* | 10084 | 10119 | -3 | 1.70E-06 | *orf397a* | *orf397a* |
| R1781 | 36 | F | *Z. caespitosa* | 9129 | 9164 | *Z. caespitosa* | 10084 | 10119 | -3 | 1.70E-06 | *orf397a* | *orf397a* |
| R1782 | 36 | F | *Z. caespitosa* | 69916 | 69951 | *Z. caespitosa* | 172966 | 173001 | -3 | 1.70E-06 | *_* | *orf185a* |
| R1783 | 36 | F | *Z. caespitosa* | 48664 | 48699 | *Z. caespitosa* | 69916 | 69951 | -3 | 1.70E-06 | *_* | *_* |
| R1784 | 36 | F | *Z. caespitosa* | 16144 | 16179 | *Z. caespitosa* | 172966 | 173001 | -3 | 1.70E-06 | *orf284a* | *orf185a* |
| R1785 | 36 | F | *Z. caespitosa* | 16144 | 16179 | *Z. caespitosa* | 48664 | 48699 | -3 | 1.70E-06 | *orf284a* | *_* |
| R1786 | 36 | F | *Z. caespitosa* | 162777 | 162812 | *Z. caespitosa* | 172966 | 173001 | -3 | 1.70E-06 | *_* | *orf185a* |
| R1787 | 36 | F | *Z. caespitosa* | 48664 | 48699 | *Z. caespitosa* | 162777 | 162812 | -3 | 1.70E-06 | *_* | *_* |
| R1788 | 36 | F | *Z. caespitosa* | 69916 | 69951 | *Z. caespitosa* | 118898 | 118933 | -3 | 1.70E-06 | *_* | *_* |
| R1789 | 36 | F | *Z. caespitosa* | 16144 | 16179 | *Z. caespitosa* | 118898 | 118933 | -3 | 1.70E-06 | *orf284a* | *_* |
| R1790 | 36 | F | *Z. caespitosa* | 118898 | 118933 | *Z. caespitosa* | 162777 | 162812 | -3 | 1.70E-06 | *_* | *_* |
| R1791 | 36 | F | *Z. caespitosa* | 55902 | 55937 | *Z. caespitosa* | 73247 | 73282 | -3 | 1.70E-06 | *rrn18* | *_* |
| R1792 | 36 | F | *Z. caespitosa* | 172845 | 172880 | *Z. caespitosa* | 172880 | 172915 | 0 | 8.80E-12 | *orf185a* | *orf185a* |
| R1793 | 36 | F | *Z. caespitosa* | 71948 | 71983 | *Z. caespitosa* | 74579 | 74614 | -1 | 9.51E-10 | *_* | *_* |
| R1794 | 36 | F | *Z. caespitosa* | 74579 | 74614 | *Z. caespitosa* | 79526 | 79561 | -1 | 9.51E-10 | *_* | *_* |
| R1795 | 36 | F | *Z. caespitosa* | 69928 | 69963 | *Z. caespitosa* | 116598 | 116633 | -2 | 4.99E-08 | *_* | *_* |
| R1796 | 36 | F | *Z. caespitosa* | 16156 | 16191 | *Z. caespitosa* | 116598 | 116633 | -2 | 4.99E-08 | *orf284a* | *_* |
| R1797 | 36 | F | *Z. caespitosa* | 116598 | 116633 | *Z. caespitosa* | 162789 | 162824 | -2 | 4.99E-08 | *_* | *_* |
| R1798 | 36 | F | *Z. caespitosa* | 1783 | 1818 | *Z. caespitosa* | 149707 | 149742 | -2 | 4.99E-08 | *_* | *_* |
| R1799 | 36 | F | *Z. caespitosa* | 43731 | 43766 | *Z. caespitosa* | 149707 | 149742 | -2 | 4.99E-08 | *_* | *_* |
| R1800 | 36 | F | *Z. caespitosa* | 15688 | 15723 | *Z. caespitosa* | 15762 | 15797 | -1 | 9.51E-10 | *_* | *_* |
| R1801 | 36 | F | *Z. caespitosa* | 56709 | 56744 | *Z. caespitosa* | 74066 | 74101 | -3 | 1.70E-06 | *rrn18* | *_* |
| R1802 | 36 | F | *Z. caespitosa* | 48410 | 48445 | *Z. caespitosa* | 68266 | 68301 | 0 | 8.80E-12 | *_* | *nad9* |
| R1803 | 36 | F | *Z. caespitosa* | 137095 | 137130 | *Z. caespitosa* | 177843 | 177878 | -1 | 9.51E-10 | *_* | *_* |
| R1804 | 36 | F | *Z. caespitosa* | 83864 | 83899 | *Z. caespitosa* | 137095 | 137130 | -1 | 9.51E-10 | *_* | *_* |
| R1805 | 36 | F | *Z. caespitosa* | 4499 | 4534 | *Z. caespitosa* | 137095 | 137130 | -1 | 9.51E-10 | *_* | *_* |
| R1806 | 36 | F | *Z. caespitosa* | 137095 | 137130 | *Z. caespitosa* | 180807 | 180842 | -1 | 9.51E-10 | *_* | *_* |
| R1807 | 36 | F | *Z. caespitosa* | 137095 | 137130 | *Z. caespitosa* | 177665 | 177700 | -2 | 4.99E-08 | *_* | *_* |
| R1808 | 36 | F | *Z. caespitosa* | 137095 | 137130 | *Z. caespitosa* | 180718 | 180753 | -2 | 4.99E-08 | *_* | *_* |
| R1809 | 36 | F | *Z. caespitosa* | 4173 | 4208 | *Z. caespitosa* | 137095 | 137130 | -2 | 4.99E-08 | *_* | *_* |
| R1810 | 36 | F | *Z. caespitosa* | 83627 | 83662 | *Z. caespitosa* | 137095 | 137130 | -2 | 4.99E-08 | *_* | *_* |
| R1811 | 36 | F | *Z. caespitosa* | 4262 | 4297 | *Z. caespitosa* | 137095 | 137130 | -2 | 4.99E-08 | *_* | *_* |
| R1812 | 36 | F | *Z. caespitosa* | 83716 | 83751 | *Z. caespitosa* | 137095 | 137130 | -2 | 4.99E-08 | *_* | *_* |
| R1813 | 36 | F | *Z. caespitosa* | 4351 | 4386 | *Z. caespitosa* | 137095 | 137130 | -2 | 4.99E-08 | *_* | *_* |
| R1814 | 36 | F | *Z. caespitosa* | 137095 | 137130 | *Z. caespitosa* | 177784 | 177819 | -2 | 4.99E-08 | *_* | *_* |
| R1815 | 36 | F | *Z. caespitosa* | 83805 | 83840 | *Z. caespitosa* | 137095 | 137130 | -2 | 4.99E-08 | *_* | *_* |
| R1816 | 36 | F | *Z. caespitosa* | 4440 | 4475 | *Z. caespitosa* | 137095 | 137130 | -2 | 4.99E-08 | *_* | *_* |
| R1817 | 36 | F | *Z. caespitosa* | 128916 | 128951 | *Z. caespitosa* | 128939 | 128974 | -3 | 1.70E-06 | *_* | *_* |
| R1818 | 36 | F | *Z. caespitosa* | 188116 | 188151 | *Z. caespitosa* | 188238 | 188273 | 0 | 8.80E-12 | *_* | *_* |
| R1819 | 36 | F | *Z. caespitosa* | 101729 | 101764 | *Z. caespitosa* | 188238 | 188273 | 0 | 8.80E-12 | *_* | *_* |
| R1820 | 36 | F | *Z. caespitosa* | 179942 | 179977 | *Z. caespitosa* | 188238 | 188273 | 0 | 8.80E-12 | *_* | *_* |
| R1821 | 36 | F | *Z. caespitosa* | 108762 | 108797 | *Z. caespitosa* | 181570 | 181605 | 0 | 8.80E-12 | *orf161a* | *orf103d* |
| R1822 | 36 | F | *Z. caespitosa* | 108762 | 108797 | *Z. caespitosa* | 136865 | 136900 | 0 | 8.80E-12 | *orf161a* | *_* |
| R1823 | 36 | F | *Z. caespitosa* | 108762 | 108797 | *Z. caespitosa* | 177905 | 177940 | 0 | 8.80E-12 | *orf161a* | *_* |
| R1824 | 36 | F | *Z. caespitosa* | 83926 | 83961 | *Z. caespitosa* | 108762 | 108797 | 0 | 8.80E-12 | *_* | *orf161a* |
| R1825 | 36 | F | *Z. caespitosa* | 41037 | 41072 | *Z. caespitosa* | 68449 | 68484 | -3 | 1.70E-06 | *orf309a* | *_* |
| R1826 | 36 | F | *Z. caespitosa* | 24392 | 24427 | *Z. caespitosa* | 46092 | 46127 | -3 | 1.70E-06 | *_* | *_* |
| R1827 | 36 | F | *Z. caespitosa* | 46092 | 46127 | *Z. caespitosa* | 95122 | 95157 | -3 | 1.70E-06 | *_* | *_* |
| R1828 | 36 | F | *Z. caespitosa* | 24392 | 24427 | *Z. caespitosa* | 177697 | 177732 | -3 | 1.70E-06 | *_* | *_* |
| R1829 | 36 | F | *Z. caespitosa* | 95122 | 95157 | *Z. caespitosa* | 177697 | 177732 | -3 | 1.70E-06 | *_* | *_* |
| R1830 | 36 | F | *Z. caespitosa* | 24392 | 24427 | *Z. caespitosa* | 180750 | 180785 | -3 | 1.70E-06 | *_* | *_* |
| R1831 | 36 | F | *Z. caespitosa* | 95122 | 95157 | *Z. caespitosa* | 180750 | 180785 | -3 | 1.70E-06 | *_* | *_* |
| R1832 | 36 | F | *Z. caespitosa* | 4205 | 4240 | *Z. caespitosa* | 24392 | 24427 | -3 | 1.70E-06 | *_* | *_* |
| R1833 | 36 | F | *Z. caespitosa* | 4205 | 4240 | *Z. caespitosa* | 95122 | 95157 | -3 | 1.70E-06 | *_* | *_* |
| R1834 | 36 | F | *Z. caespitosa* | 24392 | 24427 | *Z. caespitosa* | 83659 | 83694 | -3 | 1.70E-06 | *_* | *_* |
| R1835 | 36 | F | *Z. caespitosa* | 83659 | 83694 | *Z. caespitosa* | 95122 | 95157 | -3 | 1.70E-06 | *_* | *_* |
| R1836 | 36 | F | *Z. caespitosa* | 4294 | 4329 | *Z. caespitosa* | 24392 | 24427 | -3 | 1.70E-06 | *_* | *_* |
| R1837 | 36 | F | *Z. caespitosa* | 4294 | 4329 | *Z. caespitosa* | 95122 | 95157 | -3 | 1.70E-06 | *_* | *_* |
| R1838 | 36 | F | *Z. caespitosa* | 24392 | 24427 | *Z. caespitosa* | 177727 | 177762 | -3 | 1.70E-06 | *_* | *_* |
| R1839 | 36 | F | *Z. caespitosa* | 95122 | 95157 | *Z. caespitosa* | 177727 | 177762 | -3 | 1.70E-06 | *_* | *_* |
| R1840 | 36 | F | *Z. caespitosa* | 24392 | 24427 | *Z. caespitosa* | 83748 | 83783 | -3 | 1.70E-06 | *_* | *_* |
| R1841 | 36 | F | *Z. caespitosa* | 83748 | 83783 | *Z. caespitosa* | 95122 | 95157 | -3 | 1.70E-06 | *_* | *_* |
| R1842 | 36 | F | *Z. caespitosa* | 4383 | 4418 | *Z. caespitosa* | 24392 | 24427 | -3 | 1.70E-06 | *_* | *_* |
| R1843 | 36 | F | *Z. caespitosa* | 4383 | 4418 | *Z. caespitosa* | 95122 | 95157 | -3 | 1.70E-06 | *_* | *_* |
| R1844 | 36 | F | *Z. caespitosa* | 24392 | 24427 | *Z. caespitosa* | 177816 | 177851 | -3 | 1.70E-06 | *_* | *_* |
| R1845 | 36 | F | *Z. caespitosa* | 95122 | 95157 | *Z. caespitosa* | 177816 | 177851 | -3 | 1.70E-06 | *_* | *_* |
| R1846 | 36 | F | *Z. caespitosa* | 24392 | 24427 | *Z. caespitosa* | 83837 | 83872 | -3 | 1.70E-06 | *_* | *_* |
| R1847 | 36 | F | *Z. caespitosa* | 83837 | 83872 | *Z. caespitosa* | 95122 | 95157 | -3 | 1.70E-06 | *_* | *_* |
| R1848 | 36 | F | *Z. caespitosa* | 4472 | 4507 | *Z. caespitosa* | 24392 | 24427 | -3 | 1.70E-06 | *_* | *_* |
| R1849 | 36 | F | *Z. caespitosa* | 4472 | 4507 | *Z. caespitosa* | 95122 | 95157 | -3 | 1.70E-06 | *_* | *_* |
| R1850 | 36 | F | *Z. caespitosa* | 24392 | 24427 | *Z. caespitosa* | 180780 | 180815 | -3 | 1.70E-06 | *_* | *_* |
| R1851 | 36 | F | *Z. caespitosa* | 95122 | 95157 | *Z. caespitosa* | 180780 | 180815 | -3 | 1.70E-06 | *_* | *_* |
| R1852 | 36 | F | *Z. caespitosa* | 4146 | 4181 | *Z. caespitosa* | 24392 | 24427 | -3 | 1.70E-06 | *_* | *_* |
| R1853 | 36 | F | *Z. caespitosa* | 4146 | 4181 | *Z. caespitosa* | 95122 | 95157 | -3 | 1.70E-06 | *_* | *_* |
| R1854 | 36 | F | *Z. caespitosa* | 24392 | 24427 | *Z. caespitosa* | 83600 | 83635 | -3 | 1.70E-06 | *_* | *_* |
| R1855 | 36 | F | *Z. caespitosa* | 83600 | 83635 | *Z. caespitosa* | 95122 | 95157 | -3 | 1.70E-06 | *_* | *_* |
| R1856 | 36 | F | *Z. caespitosa* | 4235 | 4270 | *Z. caespitosa* | 24392 | 24427 | -3 | 1.70E-06 | *_* | *_* |
| R1857 | 36 | F | *Z. caespitosa* | 4235 | 4270 | *Z. caespitosa* | 95122 | 95157 | -3 | 1.70E-06 | *_* | *_* |
| R1858 | 36 | F | *Z. caespitosa* | 24392 | 24427 | *Z. caespitosa* | 83689 | 83724 | -3 | 1.70E-06 | *_* | *_* |
| R1859 | 36 | F | *Z. caespitosa* | 83689 | 83724 | *Z. caespitosa* | 95122 | 95157 | -3 | 1.70E-06 | *_* | *_* |
| R1860 | 36 | F | *Z. caespitosa* | 4324 | 4359 | *Z. caespitosa* | 24392 | 24427 | -3 | 1.70E-06 | *_* | *_* |
| R1861 | 36 | F | *Z. caespitosa* | 4324 | 4359 | *Z. caespitosa* | 95122 | 95157 | -3 | 1.70E-06 | *_* | *_* |
| R1862 | 36 | F | *Z. caespitosa* | 24392 | 24427 | *Z. caespitosa* | 177757 | 177792 | -3 | 1.70E-06 | *_* | *_* |
| R1863 | 36 | F | *Z. caespitosa* | 95122 | 95157 | *Z. caespitosa* | 177757 | 177792 | -3 | 1.70E-06 | *_* | *_* |
| R1864 | 36 | F | *Z. caespitosa* | 24392 | 24427 | *Z. caespitosa* | 83778 | 83813 | -3 | 1.70E-06 | *_* | *_* |
| R1865 | 36 | F | *Z. caespitosa* | 83778 | 83813 | *Z. caespitosa* | 95122 | 95157 | -3 | 1.70E-06 | *_* | *_* |
| R1866 | 36 | F | *Z. caespitosa* | 4413 | 4448 | *Z. caespitosa* | 24392 | 24427 | -3 | 1.70E-06 | *_* | *_* |
| R1867 | 36 | F | *Z. caespitosa* | 4413 | 4448 | *Z. caespitosa* | 95122 | 95157 | -3 | 1.70E-06 | *_* | *_* |
| R1868 | 36 | F | *Z. caespitosa* | 24392 | 24427 | *Z. caespitosa* | 52801 | 52836 | -2 | 4.99E-08 | *_* | *orf172a* |
| R1869 | 36 | F | *Z. caespitosa* | 52801 | 52836 | *Z. caespitosa* | 95122 | 95157 | -2 | 4.99E-08 | *orf172a* | *_* |
| R1870 | 36 | F | *Z. caespitosa* | 24392 | 24427 | *Z. caespitosa* | 57584 | 57619 | -2 | 4.99E-08 | *_* | *orf117a* |
| R1871 | 36 | F | *Z. caespitosa* | 57584 | 57619 | *Z. caespitosa* | 95122 | 95157 | -2 | 4.99E-08 | *orf117a* | *_* |
| R1872 | 36 | F | *Z. caespitosa* | 24392 | 24427 | *Z. caespitosa* | 156616 | 156651 | -2 | 4.99E-08 | *_* | *_* |
| R1873 | 36 | F | *Z. caespitosa* | 95122 | 95157 | *Z. caespitosa* | 156616 | 156651 | -2 | 4.99E-08 | *_* | *_* |
| R1874 | 36 | F | *Z. caespitosa* | 22092 | 22127 | *Z. caespitosa* | 52813 | 52848 | -3 | 1.70E-06 | *_* | *orf172a* |
| R1875 | 36 | F | *Z. caespitosa* | 22092 | 22127 | *Z. caespitosa* | 57596 | 57631 | -3 | 1.70E-06 | *_* | *orf117a* |
| R1876 | 36 | F | *Z. caespitosa* | 22092 | 22127 | *Z. caespitosa* | 156598 | 156633 | -3 | 1.70E-06 | *_* | *_* |
| R1877 | 36 | F | *Z. caespitosa* | 52813 | 52848 | *Z. caespitosa* | 180582 | 180617 | -3 | 1.70E-06 | *orf172a* | *_* |
| R1878 | 36 | F | *Z. caespitosa* | 57596 | 57631 | *Z. caespitosa* | 180582 | 180617 | -3 | 1.70E-06 | *orf117a* | *_* |
| R1879 | 36 | F | *Z. caespitosa* | 156598 | 156633 | *Z. caespitosa* | 180582 | 180617 | -3 | 1.70E-06 | *_* | *_* |
| R1880 | 36 | F | *Z. caespitosa* | 34938 | 34973 | *Z. caespitosa* | 156636 | 156671 | -3 | 1.70E-06 | *_* | *_* |
| R1881 | 36 | F | *Z. caespitosa* | 34248 | 34283 | *Z. caespitosa* | 186496 | 186531 | -2 | 4.99E-08 | *_* | *orf100b* |
| R1882 | 36 | F | *Z. caespitosa* | 55123 | 55158 | *Z. caespitosa* | 186496 | 186531 | -2 | 4.99E-08 | *_* | *orf100b* |
| R1883 | 36 | F | *Z. caespitosa* | 22092 | 22127 | *Z. caespitosa* | 156628 | 156663 | -3 | 1.70E-06 | *_* | *_* |
| R1884 | 36 | F | *Z. caespitosa* | 156628 | 156663 | *Z. caespitosa* | 180582 | 180617 | -3 | 1.70E-06 | *_* | *_* |
| R1885 | 36 | F | *Z. caespitosa* | 98148 | 98183 | *Z. caespitosa* | 190832 | 190867 | -1 | 9.51E-10 | *_* | *_* |
| R1886 | 36 | F | *Z. caespitosa* | 79732 | 79767 | *Z. caespitosa* | 179992 | 180027 | -1 | 9.51E-10 | *_* | *_* |
| R1887 | 36 | F | *Z. caespitosa* | 68262 | 68297 | *Z. caespitosa* | 139953 | 139988 | -3 | 1.70E-06 | *nad9* | *matR* |
| R1888 | 36 | F | *Z. caespitosa* | 40598 | 40633 | *Z. caespitosa* | 126844 | 126879 | -3 | 1.70E-06 | *orf309a* | *orf242a* |
| R1889 | 36 | F | *Z. caespitosa* | 9335 | 9370 | *Z. caespitosa* | 9972 | 10007 | -3 | 1.70E-06 | *orf397a* | *orf397a* |
| R1890 | 36 | F | *Z. caespitosa* | 9271 | 9306 | *Z. caespitosa* | 9972 | 10007 | -3 | 1.70E-06 | *orf397a* | *orf397a* |
| R1891 | 36 | F | *Z. caespitosa* | 9207 | 9242 | *Z. caespitosa* | 9972 | 10007 | -3 | 1.70E-06 | *orf397a* | *orf397a* |
| R1892 | 36 | F | *Z. caespitosa* | 9463 | 9498 | *Z. caespitosa* | 9972 | 10007 | -3 | 1.70E-06 | *orf397a* | *orf397a* |
| R1893 | 36 | F | *Z. caespitosa* | 9527 | 9562 | *Z. caespitosa* | 9972 | 10007 | -3 | 1.70E-06 | *orf397a* | *orf397a* |
| R1894 | 36 | F | *Z. caespitosa* | 9591 | 9626 | *Z. caespitosa* | 9972 | 10007 | -3 | 1.70E-06 | *orf397a* | *orf397a* |
| R1895 | 36 | R | *Z. caespitosa* | 13285 | 13320 | *Z. caespitosa* | 13293 | 13328 | -3 | 1.70E-06 | *_* | *_* |
| R1896 | 36 | P | *Z. caespitosa* | 113573 | 113608 | *Z. caespitosa* | 190832 | 190867 | -1 | 9.51E-10 | *_* | *_* |
| R1897 | 36 | P | *Z. caespitosa* | 37916 | 37951 | *Z. caespitosa* | 188238 | 188273 | 0 | 8.80E-12 | *_* | *_* |
| R1898 | 36 | P | *Z. caespitosa* | 108798 | 108833 | *Z. caespitosa* | 188238 | 188273 | 0 | 8.80E-12 | *orf161a* | *_* |
| R1899 | 36 | P | *Z. caespitosa* | 52662 | 52697 | *Z. caespitosa* | 188238 | 188273 | 0 | 8.80E-12 | *orf172a* | *_* |
| R1900 | 36 | P | *Z. caespitosa* | 52931 | 52966 | *Z. caespitosa* | 188238 | 188273 | 0 | 8.80E-12 | *orf172a* | *_* |
| R1901 | 36 | P | *Z. caespitosa* | 75937 | 75972 | *Z. caespitosa* | 188177 | 188212 | -3 | 1.70E-06 | *_* | *_* |
| R1902 | 36 | P | *Z. caespitosa* | 184229 | 184264 | *Z. caespitosa* | 188177 | 188212 | -3 | 1.70E-06 | *orf130b* | *_* |
| R1903 | 36 | P | *Z. caespitosa* | 172947 | 172982 | *Z. caespitosa* | 186496 | 186531 | -2 | 4.99E-08 | *orf185a* | *orf100b* |
| R1904 | 36 | P | *Z. caespitosa* | 179992 | 180027 | *Z. caespitosa* | 186407 | 186442 | -1 | 9.51E-10 | *_* | *orf100b* |
| R1905 | 36 | P | *Z. caespitosa* | 173975 | 174010 | *Z. caespitosa* | 184316 | 184351 | 0 | 8.80E-12 | *_* | *_* |
| R1906 | 36 | P | *Z. caespitosa* | 140157 | 140192 | *Z. caespitosa* | 184229 | 184264 | -3 | 1.70E-06 | *matR* | *orf130b* |
| R1907 | 36 | P | *Z. caespitosa* | 116598 | 116633 | *Z. caespitosa* | 180780 | 180815 | -3 | 1.70E-06 | *_* | *_* |
| R1908 | 36 | P | *Z. caespitosa* | 116598 | 116633 | *Z. caespitosa* | 180750 | 180785 | -3 | 1.70E-06 | *_* | *_* |
| R1909 | 36 | P | *Z. caespitosa* | 54465 | 54500 | *Z. caespitosa* | 180696 | 180731 | -2 | 4.99E-08 | *orf168a* | *_* |
| R1910 | 36 | P | *Z. caespitosa* | 138690 | 138725 | *Z. caespitosa* | 180630 | 180665 | -3 | 1.70E-06 | *_* | *_* |
| R1911 | 36 | P | *Z. caespitosa* | 162777 | 162812 | *Z. caespitosa* | 180582 | 180617 | -3 | 1.70E-06 | *_* | *_* |
| R1912 | 36 | P | *Z. caespitosa* | 16144 | 16179 | *Z. caespitosa* | 180582 | 180617 | -3 | 1.70E-06 | *orf284a* | *_* |
| R1913 | 36 | P | *Z. caespitosa* | 69916 | 69951 | *Z. caespitosa* | 180582 | 180617 | -3 | 1.70E-06 | *_* | *_* |
| R1914 | 36 | P | *Z. caespitosa* | 15872 | 15907 | *Z. caespitosa* | 177858 | 177893 | 0 | 8.80E-12 | *_* | *_* |
| R1915 | 36 | P | *Z. caespitosa* | 116598 | 116633 | *Z. caespitosa* | 177816 | 177851 | -3 | 1.70E-06 | *_* | *_* |
| R1916 | 36 | P | *Z. caespitosa* | 116598 | 116633 | *Z. caespitosa* | 177757 | 177792 | -3 | 1.70E-06 | *_* | *_* |
| R1917 | 36 | P | *Z. caespitosa* | 116598 | 116633 | *Z. caespitosa* | 177727 | 177762 | -3 | 1.70E-06 | *_* | *_* |
| R1918 | 36 | P | *Z. caespitosa* | 116598 | 116633 | *Z. caespitosa* | 177697 | 177732 | -3 | 1.70E-06 | *_* | *_* |
| R1919 | 36 | P | *Z. caespitosa* | 54465 | 54500 | *Z. caespitosa* | 177643 | 177678 | -2 | 4.99E-08 | *orf168a* | *_* |
| R1920 | 36 | P | *Z. caespitosa* | 156598 | 156633 | *Z. caespitosa* | 172966 | 173001 | -3 | 1.70E-06 | *_* | *orf185a* |
| R1921 | 36 | P | *Z. caespitosa* | 57596 | 57631 | *Z. caespitosa* | 172966 | 173001 | -3 | 1.70E-06 | *orf117a* | *orf185a* |
| R1922 | 36 | P | *Z. caespitosa* | 52813 | 52848 | *Z. caespitosa* | 172966 | 173001 | -3 | 1.70E-06 | *orf172a* | *orf185a* |
| R1923 | 36 | P | *Z. caespitosa* | 57636 | 57671 | *Z. caespitosa* | 172880 | 172915 | 0 | 8.80E-12 | *orf117a* | *orf185a* |
| R1924 | 36 | P | *Z. caespitosa* | 75653 | 75688 | *Z. caespitosa* | 172880 | 172915 | 0 | 8.80E-12 | *_* | *orf185a* |
| R1925 | 36 | P | *Z. caespitosa* | 50022 | 50057 | *Z. caespitosa* | 172851 | 172886 | -3 | 1.70E-06 | *_* | *orf185a* |
| R1926 | 36 | P | *Z. caespitosa* | 92113 | 92148 | *Z. caespitosa* | 169359 | 169394 | 0 | 8.80E-12 | *_* | *_* |
| R1927 | 36 | P | *Z. caespitosa* | 84308 | 84343 | *Z. caespitosa* | 162872 | 162907 | 0 | 8.80E-12 | *_* | *_* |
| R1928 | 36 | P | *Z. caespitosa* | 24392 | 24427 | *Z. caespitosa* | 162789 | 162824 | -2 | 4.99E-08 | *_* | *_* |
| R1929 | 36 | P | *Z. caespitosa* | 95122 | 95157 | *Z. caespitosa* | 162789 | 162824 | -2 | 4.99E-08 | *_* | *_* |
| R1930 | 36 | P | *Z. caespitosa* | 22092 | 22127 | *Z. caespitosa* | 162777 | 162812 | -3 | 1.70E-06 | *_* | *_* |
| R1931 | 36 | P | *Z. caespitosa* | 45959 | 45994 | *Z. caespitosa* | 162582 | 162617 | -1 | 9.51E-10 | *_* | *_* |
| R1932 | 36 | P | *Z. caespitosa* | 4013 | 4048 | *Z. caespitosa* | 162582 | 162617 | -1 | 9.51E-10 | *_* | *_* |
| R1933 | 36 | P | *Z. caespitosa* | 118898 | 118933 | *Z. caespitosa* | 156628 | 156663 | -3 | 1.70E-06 | *_* | *_* |
| R1934 | 36 | P | *Z. caespitosa* | 116598 | 116633 | *Z. caespitosa* | 156616 | 156651 | -2 | 4.99E-08 | *_* | *_* |
| R1935 | 36 | P | *Z. caespitosa* | 118898 | 118933 | *Z. caespitosa* | 156598 | 156633 | -3 | 1.70E-06 | *_* | *_* |
| R1936 | 36 | P | *Z. caespitosa* | 48664 | 48699 | *Z. caespitosa* | 156598 | 156633 | -3 | 1.70E-06 | *_* | *_* |
| R1937 | 36 | P | *Z. caespitosa* | 75937 | 75972 | *Z. caespitosa* | 140157 | 140192 | -3 | 1.70E-06 | *_* | *matR* |
| R1938 | 36 | P | *Z. caespitosa* | 50023 | 50058 | *Z. caespitosa* | 138941 | 138976 | -2 | 4.99E-08 | *_* | *_* |
| R1939 | 36 | P | *Z. caespitosa* | 52813 | 52848 | *Z. caespitosa* | 118898 | 118933 | -3 | 1.70E-06 | *orf172a* | *_* |
| R1940 | 36 | P | *Z. caespitosa* | 57596 | 57631 | *Z. caespitosa* | 118898 | 118933 | -3 | 1.70E-06 | *orf117a* | *_* |
| R1941 | 36 | P | *Z. caespitosa* | 22458 | 22493 | *Z. caespitosa* | 118548 | 118583 | -2 | 4.99E-08 | *_* | *_* |
| R1942 | 36 | P | *Z. caespitosa* | 22442 | 22477 | *Z. caespitosa* | 118532 | 118567 | -2 | 4.99E-08 | *_* | *_* |
| R1943 | 36 | P | *Z. caespitosa* | 4413 | 4448 | *Z. caespitosa* | 116598 | 116633 | -3 | 1.70E-06 | *_* | *_* |
| R1944 | 36 | P | *Z. caespitosa* | 83778 | 83813 | *Z. caespitosa* | 116598 | 116633 | -3 | 1.70E-06 | *_* | *_* |
| R1945 | 36 | P | *Z. caespitosa* | 4324 | 4359 | *Z. caespitosa* | 116598 | 116633 | -3 | 1.70E-06 | *_* | *_* |
| R1946 | 36 | P | *Z. caespitosa* | 83689 | 83724 | *Z. caespitosa* | 116598 | 116633 | -3 | 1.70E-06 | *_* | *_* |
| R1947 | 36 | P | *Z. caespitosa* | 4235 | 4270 | *Z. caespitosa* | 116598 | 116633 | -3 | 1.70E-06 | *_* | *_* |
| R1948 | 36 | P | *Z. caespitosa* | 83600 | 83635 | *Z. caespitosa* | 116598 | 116633 | -3 | 1.70E-06 | *_* | *_* |
| R1949 | 36 | P | *Z. caespitosa* | 4146 | 4181 | *Z. caespitosa* | 116598 | 116633 | -3 | 1.70E-06 | *_* | *_* |
| R1950 | 36 | P | *Z. caespitosa* | 4472 | 4507 | *Z. caespitosa* | 116598 | 116633 | -3 | 1.70E-06 | *_* | *_* |
| R1951 | 36 | P | *Z. caespitosa* | 83837 | 83872 | *Z. caespitosa* | 116598 | 116633 | -3 | 1.70E-06 | *_* | *_* |
| R1952 | 36 | P | *Z. caespitosa* | 4383 | 4418 | *Z. caespitosa* | 116598 | 116633 | -3 | 1.70E-06 | *_* | *_* |
| R1953 | 36 | P | *Z. caespitosa* | 83748 | 83783 | *Z. caespitosa* | 116598 | 116633 | -3 | 1.70E-06 | *_* | *_* |
| R1954 | 36 | P | *Z. caespitosa* | 4294 | 4329 | *Z. caespitosa* | 116598 | 116633 | -3 | 1.70E-06 | *_* | *_* |
| R1955 | 36 | P | *Z. caespitosa* | 83659 | 83694 | *Z. caespitosa* | 116598 | 116633 | -3 | 1.70E-06 | *_* | *_* |
| R1956 | 36 | P | *Z. caespitosa* | 4205 | 4240 | *Z. caespitosa* | 116598 | 116633 | -3 | 1.70E-06 | *_* | *_* |
| R1957 | 36 | P | *Z. caespitosa* | 46092 | 46127 | *Z. caespitosa* | 116598 | 116633 | -3 | 1.70E-06 | *_* | *_* |
| R1958 | 36 | P | *Z. caespitosa* | 57584 | 57619 | *Z. caespitosa* | 116598 | 116633 | -2 | 4.99E-08 | *orf117a* | *_* |
| R1959 | 36 | P | *Z. caespitosa* | 52801 | 52836 | *Z. caespitosa* | 116598 | 116633 | -2 | 4.99E-08 | *orf172a* | *_* |
| R1960 | 36 | P | *Z. caespitosa* | 92113 | 92148 | *Z. caespitosa* | 107745 | 107780 | 0 | 8.80E-12 | *_* | *orf177a* |
| R1961 | 36 | P | *Z. caespitosa* | 33018 | 33053 | *Z. caespitosa* | 95733 | 95768 | 0 | 8.80E-12 | *_* | *_* |
| R1962 | 36 | P | *Z. caespitosa* | 69928 | 69963 | *Z. caespitosa* | 95122 | 95157 | -2 | 4.99E-08 | *_* | *_* |
| R1963 | 36 | P | *Z. caespitosa* | 16156 | 16191 | *Z. caespitosa* | 95122 | 95157 | -2 | 4.99E-08 | *orf284a* | *_* |
| R1964 | 36 | P | *Z. caespitosa* | 43731 | 43766 | *Z. caespitosa* | 92114 | 92149 | 0 | 8.80E-12 | *_* | *_* |
| R1965 | 36 | P | *Z. caespitosa* | 1783 | 1818 | *Z. caespitosa* | 92114 | 92149 | 0 | 8.80E-12 | *_* | *_* |
| R1966 | 36 | P | *Z. caespitosa* | 15872 | 15907 | *Z. caespitosa* | 83879 | 83914 | 0 | 8.80E-12 | *_* | *_* |
| R1967 | 36 | P | *Z. caespitosa* | 15578 | 15613 | *Z. caespitosa* | 76022 | 76057 | 0 | 8.80E-12 | *_* | *_* |
| R1968 | 36 | P | *Z. caespitosa* | 53044 | 53079 | *Z. caespitosa* | 74746 | 74781 | -3 | 1.70E-06 | *_* | *_* |
| R1969 | 36 | P | *Z. caespitosa* | 53009 | 53044 | *Z. caespitosa* | 74746 | 74781 | -3 | 1.70E-06 | *_* | *_* |
| R1970 | 36 | P | *Z. caespitosa* | 51674 | 51709 | *Z. caespitosa* | 74579 | 74614 | -1 | 9.51E-10 | *orf106b;ccmFn* | *_* |
| R1971 | 36 | P | *Z. caespitosa* | 24392 | 24427 | *Z. caespitosa* | 69928 | 69963 | -2 | 4.99E-08 | *_* | *_* |
| R1972 | 36 | P | *Z. caespitosa* | 22092 | 22127 | *Z. caespitosa* | 69916 | 69951 | -3 | 1.70E-06 | *_* | *_* |
| R1973 | 36 | P | *Z. caespitosa* | 28934 | 28969 | *Z. caespitosa* | 68697 | 68732 | -3 | 1.70E-06 | *_* | *_* |
| R1974 | 36 | P | *Z. caespitosa* | 48664 | 48699 | *Z. caespitosa* | 57596 | 57631 | -3 | 1.70E-06 | *_* | *orf117a* |
| R1975 | 36 | P | *Z. caespitosa* | 48664 | 48699 | *Z. caespitosa* | 52813 | 52848 | -3 | 1.70E-06 | *_* | *orf172a* |
| R1976 | 36 | P | *Z. caespitosa* | 15808 | 15843 | *Z. caespitosa* | 45957 | 45992 | -3 | 1.70E-06 | *_* | *_* |
| R1977 | 36 | P | *Z. caespitosa* | 15808 | 15843 | *Z. caespitosa* | 43390 | 43425 | -2 | 4.99E-08 | *_* | *_* |
| R1978 | 36 | P | *Z. caespitosa* | 25003 | 25038 | *Z. caespitosa* | 33018 | 33053 | 0 | 8.80E-12 | *_* | *_* |
| R1979 | 36 | P | *Z. caespitosa* | 16156 | 16191 | *Z. caespitosa* | 24392 | 24427 | -2 | 4.99E-08 | *orf284a* | *_* |
| R1980 | 36 | P | *Z. caespitosa* | 16144 | 16179 | *Z. caespitosa* | 22092 | 22127 | -3 | 1.70E-06 | *orf284a* | *_* |
| R1981 | 36 | P | *Z. caespitosa* | 1442 | 1477 | *Z. caespitosa* | 15808 | 15843 | -2 | 4.99E-08 | *_* | *_* |
| R1982 | 36 | P | *Z. caespitosa* | 4011 | 4046 | *Z. caespitosa* | 15808 | 15843 | -3 | 1.70E-06 | *_* | *_* |
| R1983 | 35 | F | *Z. caespitosa* | 69755 | 69789 | *Z. caespitosa* | 127559 | 127593 | -3 | 6.22E-06 | *_* | *orf149b* |
| R1984 | 35 | F | *Z. caespitosa* | 1784 | 1818 | *Z. caespitosa* | 169359 | 169393 | 0 | 3.52E-11 | *_* | *_* |
| R1985 | 35 | F | *Z. caespitosa* | 43732 | 43766 | *Z. caespitosa* | 169359 | 169393 | 0 | 3.52E-11 | *_* | *_* |
| R1986 | 35 | F | *Z. caespitosa* | 1784 | 1818 | *Z. caespitosa* | 107745 | 107779 | 0 | 3.52E-11 | *_* | *orf177a* |
| R1987 | 35 | F | *Z. caespitosa* | 43732 | 43766 | *Z. caespitosa* | 107745 | 107779 | 0 | 3.52E-11 | *_* | *orf177a* |
| R1988 | 35 | F | *Z. caespitosa* | 40347 | 40381 | *Z. caespitosa* | 126593 | 126627 | -3 | 6.22E-06 | *orf309a* | *orf242a* |
| R1989 | 35 | F | *Z. caespitosa* | 138591 | 138625 | *Z. caespitosa* | 139755 | 139789 | -3 | 6.22E-06 | *_* | *matR* |
| R1990 | 35 | F | *Z. caespitosa* | 69881 | 69915 | *Z. caespitosa* | 172850 | 172884 | 0 | 3.52E-11 | *_* | *orf185a* |
| R1991 | 35 | F | *Z. caespitosa* | 37821 | 37855 | *Z. caespitosa* | 135099 | 135133 | -1 | 3.70E-09 | *_* | *_* |
| R1992 | 35 | F | *Z. caespitosa* | 135099 | 135133 | *Z. caespitosa* | 148768 | 148802 | -2 | 1.89E-07 | *_* | *_* |
| R1993 | 35 | F | *Z. caespitosa* | 135099 | 135133 | *Z. caespitosa* | 148831 | 148865 | -2 | 1.89E-07 | *_* | *_* |
| R1994 | 35 | F | *Z. caespitosa* | 132090 | 132124 | *Z. caespitosa* | 183146 | 183180 | -3 | 6.22E-06 | *_* | *orf228a* |
| R1995 | 35 | F | *Z. caespitosa* | 653 | 687 | *Z. caespitosa* | 152561 | 152595 | -2 | 1.89E-07 | *_* | *_* |
| R1996 | 35 | F | *Z. caespitosa* | 42602 | 42636 | *Z. caespitosa* | 152561 | 152595 | -2 | 1.89E-07 | *_* | *_* |
| R1997 | 35 | F | *Z. caespitosa* | 1480 | 1514 | *Z. caespitosa* | 180661 | 180695 | 0 | 3.52E-11 | *_* | *_* |
| R1998 | 35 | F | *Z. caespitosa* | 43428 | 43462 | *Z. caespitosa* | 180661 | 180695 | 0 | 3.52E-11 | *_* | *_* |
| R1999 | 35 | F | *Z. caespitosa* | 155759 | 155793 | *Z. caespitosa* | 184273 | 184307 | 0 | 3.52E-11 | *_* | *_* |
| R2000 | 35 | F | *Z. caespitosa* | 155727 | 155761 | *Z. caespitosa* | 184273 | 184307 | 0 | 3.52E-11 | *_* | *_* |
| R2001 | 35 | F | *Z. caespitosa* | 155695 | 155729 | *Z. caespitosa* | 184273 | 184307 | 0 | 3.52E-11 | *_* | *_* |
| R2002 | 35 | F | *Z. caespitosa* | 155663 | 155697 | *Z. caespitosa* | 184273 | 184307 | 0 | 3.52E-11 | *_* | *_* |
| R2003 | 35 | F | *Z. caespitosa* | 16082 | 16116 | *Z. caespitosa* | 16631 | 16665 | -3 | 6.22E-06 | *orf284a* | *orf284a* |
| R2004 | 35 | F | *Z. caespitosa* | 16080 | 16114 | *Z. caespitosa* | 149206 | 149240 | 0 | 3.52E-11 | *orf284a* | *_* |
| R2005 | 35 | F | *Z. caespitosa* | 33017 | 33051 | *Z. caespitosa* | 50024 | 50058 | -2 | 1.89E-07 | *_* | *_* |
| R2006 | 35 | F | *Z. caespitosa* | 137100 | 137134 | *Z. caespitosa* | 175164 | 175198 | -3 | 6.22E-06 | *_* | *_* |
| R2007 | 35 | F | *Z. caespitosa* | 111680 | 111714 | *Z. caespitosa* | 118829 | 118863 | 0 | 3.52E-11 | *_* | *_* |
| R2008 | 35 | F | *Z. caespitosa* | 57198 | 57232 | *Z. caespitosa* | 74310 | 74344 | -3 | 6.22E-06 | *rrn18;orf152a* | *_* |
| R2009 | 35 | F | *Z. caespitosa* | 87375 | 87409 | *Z. caespitosa* | 116595 | 116629 | 0 | 3.52E-11 | *orf122a* | *_* |
| R2010 | 35 | F | *Z. caespitosa* | 74682 | 74716 | *Z. caespitosa* | 116595 | 116629 | 0 | 3.52E-11 | *_* | *_* |
| R2011 | 35 | F | *Z. caespitosa* | 116595 | 116629 | *Z. caespitosa* | 172542 | 172576 | 0 | 3.52E-11 | *_* | *_* |
| R2012 | 35 | F | *Z. caespitosa* | 77798 | 77832 | *Z. caespitosa* | 116595 | 116629 | 0 | 3.52E-11 | *_* | *_* |
| R2013 | 35 | F | *Z. caespitosa* | 116595 | 116629 | *Z. caespitosa* | 140111 | 140145 | 0 | 3.52E-11 | *_* | *matR* |
| R2014 | 35 | F | *Z. caespitosa* | 77736 | 77770 | *Z. caespitosa* | 116595 | 116629 | 0 | 3.52E-11 | *_* | *_* |
| R2015 | 35 | F | *Z. caespitosa* | 77674 | 77708 | *Z. caespitosa* | 116595 | 116629 | 0 | 3.52E-11 | *_* | *_* |
| R2016 | 35 | F | *Z. caespitosa* | 79456 | 79490 | *Z. caespitosa* | 116595 | 116629 | 0 | 3.52E-11 | *_* | *_* |
| R2017 | 35 | F | *Z. caespitosa* | 69882 | 69916 | *Z. caespitosa* | 149207 | 149241 | 0 | 3.52E-11 | *_* | *_* |
| R2018 | 35 | F | *Z. caespitosa* | 15950 | 15984 | *Z. caespitosa* | 85088 | 85122 | -1 | 3.70E-09 | *orf284a* | *_* |
| R2019 | 35 | F | *Z. caespitosa* | 7583 | 7617 | *Z. caespitosa* | 85088 | 85122 | -1 | 3.70E-09 | *_* | *_* |
| R2020 | 35 | F | *Z. caespitosa* | 23049 | 23083 | *Z. caespitosa* | 49770 | 49804 | 0 | 3.52E-11 | *_* | *_* |
| R2021 | 35 | F | *Z. caespitosa* | 49770 | 49804 | *Z. caespitosa* | 93779 | 93813 | 0 | 3.52E-11 | *_* | *_* |
| R2022 | 35 | F | *Z. caespitosa* | 53109 | 53143 | *Z. caespitosa* | 108762 | 108796 | 0 | 3.52E-11 | *_* | *orf161a* |
| R2023 | 35 | F | *Z. caespitosa* | 108762 | 108796 | *Z. caespitosa* | 148763 | 148797 | -1 | 3.70E-09 | *orf161a* | *_* |
| R2024 | 35 | F | *Z. caespitosa* | 108762 | 108796 | *Z. caespitosa* | 148826 | 148860 | -1 | 3.70E-09 | *orf161a* | *_* |
| R2025 | 35 | F | *Z. caespitosa* | 1432 | 1466 | *Z. caespitosa* | 73150 | 73184 | -3 | 6.22E-06 | *_* | *_* |
| R2026 | 35 | F | *Z. caespitosa* | 43380 | 43414 | *Z. caespitosa* | 73150 | 73184 | -3 | 6.22E-06 | *_* | *_* |
| R2027 | 35 | F | *Z. caespitosa* | 15765 | 15799 | *Z. caespitosa* | 139963 | 139997 | -3 | 6.22E-06 | *_* | *matR* |
| R2028 | 35 | F | *Z. caespitosa* | 139963 | 139997 | *Z. caespitosa* | 164726 | 164760 | -3 | 6.22E-06 | *matR* | *_* |
| R2029 | 35 | F | *Z. caespitosa* | 34934 | 34968 | *Z. caespitosa* | 100036 | 100070 | -2 | 1.89E-07 | *_* | *_* |
| R2030 | 35 | F | *Z. caespitosa* | 34934 | 34968 | *Z. caespitosa* | 115983 | 116017 | -2 | 1.89E-07 | *_* | *_* |
| R2031 | 35 | F | *Z. caespitosa* | 37943 | 37977 | *Z. caespitosa* | 175166 | 175200 | 0 | 3.52E-11 | *_* | *_* |
| R2032 | 35 | F | *Z. caespitosa* | 108825 | 108859 | *Z. caespitosa* | 175166 | 175200 | 0 | 3.52E-11 | *orf161a* | *_* |
| R2033 | 35 | F | *Z. caespitosa* | 84282 | 84316 | *Z. caespitosa* | 175166 | 175200 | 0 | 3.52E-11 | *_* | *_* |
| R2034 | 35 | F | *Z. caespitosa* | 52689 | 52723 | *Z. caespitosa* | 175166 | 175200 | 0 | 3.52E-11 | *orf172a* | *_* |
| R2035 | 35 | F | *Z. caespitosa* | 52958 | 52992 | *Z. caespitosa* | 175166 | 175200 | 0 | 3.52E-11 | *orf172a* | *_* |
| R2036 | 35 | F | *Z. caespitosa* | 53172 | 53206 | *Z. caespitosa* | 175166 | 175200 | 0 | 3.52E-11 | *_* | *_* |
| R2037 | 35 | F | *Z. caespitosa* | 24396 | 24430 | *Z. caespitosa* | 181651 | 181685 | 0 | 3.52E-11 | *_* | *orf103d* |
| R2038 | 35 | F | *Z. caespitosa* | 24396 | 24430 | *Z. caespitosa* | 75921 | 75955 | 0 | 3.52E-11 | *_* | *_* |
| R2039 | 35 | F | *Z. caespitosa* | 24396 | 24430 | *Z. caespitosa* | 184213 | 184247 | 0 | 3.52E-11 | *_* | *orf130b* |
| R2040 | 35 | F | *Z. caespitosa* | 24396 | 24430 | *Z. caespitosa* | 136946 | 136980 | 0 | 3.52E-11 | *_* | *_* |
| R2041 | 35 | F | *Z. caespitosa* | 24396 | 24430 | *Z. caespitosa* | 72632 | 72666 | 0 | 3.52E-11 | *_* | *_* |
| R2042 | 35 | F | *Z. caespitosa* | 24396 | 24430 | *Z. caespitosa* | 177986 | 178020 | 0 | 3.52E-11 | *_* | *_* |
| R2043 | 35 | F | *Z. caespitosa* | 24396 | 24430 | *Z. caespitosa* | 84007 | 84041 | 0 | 3.52E-11 | *_* | *_* |
| R2044 | 35 | F | *Z. caespitosa* | 24396 | 24430 | *Z. caespitosa* | 37754 | 37788 | 0 | 3.52E-11 | *_* | *_* |
| R2045 | 35 | F | *Z. caespitosa* | 95126 | 95160 | *Z. caespitosa* | 181651 | 181685 | 0 | 3.52E-11 | *_* | *orf103d* |
| R2046 | 35 | F | *Z. caespitosa* | 75921 | 75955 | *Z. caespitosa* | 95126 | 95160 | 0 | 3.52E-11 | *_* | *_* |
| R2047 | 35 | F | *Z. caespitosa* | 95126 | 95160 | *Z. caespitosa* | 184213 | 184247 | 0 | 3.52E-11 | *_* | *orf130b* |
| R2048 | 35 | F | *Z. caespitosa* | 95126 | 95160 | *Z. caespitosa* | 136946 | 136980 | 0 | 3.52E-11 | *_* | *_* |
| R2049 | 35 | F | *Z. caespitosa* | 72632 | 72666 | *Z. caespitosa* | 95126 | 95160 | 0 | 3.52E-11 | *_* | *_* |
| R2050 | 35 | F | *Z. caespitosa* | 95126 | 95160 | *Z. caespitosa* | 177986 | 178020 | 0 | 3.52E-11 | *_* | *_* |
| R2051 | 35 | F | *Z. caespitosa* | 84007 | 84041 | *Z. caespitosa* | 95126 | 95160 | 0 | 3.52E-11 | *_* | *_* |
| R2052 | 35 | F | *Z. caespitosa* | 37754 | 37788 | *Z. caespitosa* | 95126 | 95160 | 0 | 3.52E-11 | *_* | *_* |
| R2053 | 35 | F | *Z. caespitosa* | 24393 | 24427 | *Z. caespitosa* | 156587 | 156621 | -2 | 1.89E-07 | *_* | *_* |
| R2054 | 35 | F | *Z. caespitosa* | 95123 | 95157 | *Z. caespitosa* | 156587 | 156621 | -2 | 1.89E-07 | *_* | *_* |
| R2055 | 35 | F | *Z. caespitosa* | 34949 | 34983 | *Z. caespitosa* | 181600 | 181634 | -3 | 6.22E-06 | *_* | *orf103d* |
| R2056 | 35 | F | *Z. caespitosa* | 34949 | 34983 | *Z. caespitosa* | 184162 | 184196 | -3 | 6.22E-06 | *_* | *orf130b* |
| R2057 | 35 | F | *Z. caespitosa* | 34949 | 34983 | *Z. caespitosa* | 136895 | 136929 | -3 | 6.22E-06 | *_* | *_* |
| R2058 | 35 | F | *Z. caespitosa* | 34949 | 34983 | *Z. caespitosa* | 177935 | 177969 | -3 | 6.22E-06 | *_* | *_* |
| R2059 | 35 | F | *Z. caespitosa* | 34949 | 34983 | *Z. caespitosa* | 83956 | 83990 | -3 | 6.22E-06 | *_* | *_* |
| R2060 | 35 | F | *Z. caespitosa* | 22162 | 22196 | *Z. caespitosa* | 100042 | 100076 | 0 | 3.52E-11 | *_* | *_* |
| R2061 | 35 | F | *Z. caespitosa* | 22162 | 22196 | *Z. caespitosa* | 75984 | 76018 | -2 | 1.89E-07 | *_* | *_* |
| R2062 | 35 | F | *Z. caespitosa* | 113573 | 113607 | *Z. caespitosa* | 149709 | 149743 | -1 | 3.70E-09 | *_* | *_* |
| R2063 | 35 | F | *Z. caespitosa* | 14657 | 14691 | *Z. caespitosa* | 25091 | 25125 | -3 | 6.22E-06 | *orf295a;cox2* | *orf340a* |
| R2064 | 35 | F | *Z. caespitosa* | 14657 | 14691 | *Z. caespitosa* | 95821 | 95855 | -3 | 6.22E-06 | *orf295a;cox2* | *orf540a-2* |
| R2065 | 35 | F | *Z. caespitosa* | 37894 | 37928 | *Z. caespitosa* | 177557 | 177591 | -3 | 6.22E-06 | *_* | *_* |
| R2066 | 35 | F | *Z. caespitosa* | 108776 | 108810 | *Z. caespitosa* | 177557 | 177591 | -3 | 6.22E-06 | *orf161a* | *_* |
| R2067 | 35 | F | *Z. caespitosa* | 51828 | 51862 | *Z. caespitosa* | 156557 | 156591 | -2 | 1.89E-07 | *orf106b;ccmFn* | *_* |
| R2068 | 35 | P | *Z. caespitosa* | 46111 | 46145 | *Z. caespitosa* | 188179 | 188213 | 0 | 3.52E-11 | *_* | *_* |
| R2069 | 35 | P | *Z. caespitosa* | 177686 | 177720 | *Z. caespitosa* | 188179 | 188213 | 0 | 3.52E-11 | *_* | *_* |
| R2070 | 35 | P | *Z. caespitosa* | 180739 | 180773 | *Z. caespitosa* | 188179 | 188213 | 0 | 3.52E-11 | *_* | *_* |
| R2071 | 35 | P | *Z. caespitosa* | 4194 | 4228 | *Z. caespitosa* | 188179 | 188213 | 0 | 3.52E-11 | *_* | *_* |
| R2072 | 35 | P | *Z. caespitosa* | 83648 | 83682 | *Z. caespitosa* | 188179 | 188213 | 0 | 3.52E-11 | *_* | *_* |
| R2073 | 35 | P | *Z. caespitosa* | 4283 | 4317 | *Z. caespitosa* | 188179 | 188213 | 0 | 3.52E-11 | *_* | *_* |
| R2074 | 35 | P | *Z. caespitosa* | 177716 | 177750 | *Z. caespitosa* | 188179 | 188213 | 0 | 3.52E-11 | *_* | *_* |
| R2075 | 35 | P | *Z. caespitosa* | 83737 | 83771 | *Z. caespitosa* | 188179 | 188213 | 0 | 3.52E-11 | *_* | *_* |
| R2076 | 35 | P | *Z. caespitosa* | 4372 | 4406 | *Z. caespitosa* | 188179 | 188213 | 0 | 3.52E-11 | *_* | *_* |
| R2077 | 35 | P | *Z. caespitosa* | 177805 | 177839 | *Z. caespitosa* | 188179 | 188213 | 0 | 3.52E-11 | *_* | *_* |
| R2078 | 35 | P | *Z. caespitosa* | 83826 | 83860 | *Z. caespitosa* | 188179 | 188213 | 0 | 3.52E-11 | *_* | *_* |
| R2079 | 35 | P | *Z. caespitosa* | 4461 | 4495 | *Z. caespitosa* | 188179 | 188213 | 0 | 3.52E-11 | *_* | *_* |
| R2080 | 35 | P | *Z. caespitosa* | 180769 | 180803 | *Z. caespitosa* | 188179 | 188213 | 0 | 3.52E-11 | *_* | *_* |
| R2081 | 35 | P | *Z. caespitosa* | 4224 | 4258 | *Z. caespitosa* | 188179 | 188213 | 0 | 3.52E-11 | *_* | *_* |
| R2082 | 35 | P | *Z. caespitosa* | 83678 | 83712 | *Z. caespitosa* | 188179 | 188213 | 0 | 3.52E-11 | *_* | *_* |
| R2083 | 35 | P | *Z. caespitosa* | 4313 | 4347 | *Z. caespitosa* | 188179 | 188213 | 0 | 3.52E-11 | *_* | *_* |
| R2084 | 35 | P | *Z. caespitosa* | 177746 | 177780 | *Z. caespitosa* | 188179 | 188213 | 0 | 3.52E-11 | *_* | *_* |
| R2085 | 35 | P | *Z. caespitosa* | 83767 | 83801 | *Z. caespitosa* | 188179 | 188213 | 0 | 3.52E-11 | *_* | *_* |
| R2086 | 35 | P | *Z. caespitosa* | 4402 | 4436 | *Z. caespitosa* | 188179 | 188213 | 0 | 3.52E-11 | *_* | *_* |
| R2087 | 35 | P | *Z. caespitosa* | 181667 | 181701 | *Z. caespitosa* | 188178 | 188212 | -3 | 6.22E-06 | *orf103d* | *_* |
| R2088 | 35 | P | *Z. caespitosa* | 175166 | 175200 | *Z. caespitosa* | 188090 | 188124 | 0 | 3.52E-11 | *_* | *_* |
| R2089 | 35 | P | *Z. caespitosa* | 116595 | 116629 | *Z. caespitosa* | 184213 | 184247 | 0 | 3.52E-11 | *_* | *orf130b* |
| R2090 | 35 | P | *Z. caespitosa* | 140158 | 140192 | *Z. caespitosa* | 181667 | 181701 | -3 | 6.22E-06 | *matR* | *orf103d* |
| R2091 | 35 | P | *Z. caespitosa* | 116595 | 116629 | *Z. caespitosa* | 181651 | 181685 | 0 | 3.52E-11 | *_* | *orf103d* |
| R2092 | 35 | P | *Z. caespitosa* | 140159 | 140193 | *Z. caespitosa* | 180769 | 180803 | 0 | 3.52E-11 | *matR* | *_* |
| R2093 | 35 | P | *Z. caespitosa* | 140159 | 140193 | *Z. caespitosa* | 180739 | 180773 | 0 | 3.52E-11 | *matR* | *_* |
| R2094 | 35 | P | *Z. caespitosa* | 68386 | 68420 | *Z. caespitosa* | 180631 | 180665 | 0 | 3.52E-11 | *_* | *_* |
| R2095 | 35 | P | *Z. caespitosa* | 175166 | 175200 | *Z. caespitosa* | 179916 | 179950 | 0 | 3.52E-11 | *_* | *_* |
| R2096 | 35 | P | *Z. caespitosa* | 116595 | 116629 | *Z. caespitosa* | 177986 | 178020 | 0 | 3.52E-11 | *_* | *_* |
| R2097 | 35 | P | *Z. caespitosa* | 140159 | 140193 | *Z. caespitosa* | 177805 | 177839 | 0 | 3.52E-11 | *matR* | *_* |
| R2098 | 35 | P | *Z. caespitosa* | 140159 | 140193 | *Z. caespitosa* | 177746 | 177780 | 0 | 3.52E-11 | *matR* | *_* |
| R2099 | 35 | P | *Z. caespitosa* | 140159 | 140193 | *Z. caespitosa* | 177716 | 177750 | 0 | 3.52E-11 | *matR* | *_* |
| R2100 | 35 | P | *Z. caespitosa* | 140159 | 140193 | *Z. caespitosa* | 177686 | 177720 | 0 | 3.52E-11 | *matR* | *_* |
| R2101 | 35 | P | *Z. caespitosa* | 72205 | 72239 | *Z. caespitosa* | 175166 | 175200 | 0 | 3.52E-11 | *_* | *_* |
| R2102 | 35 | P | *Z. caespitosa* | 95126 | 95160 | *Z. caespitosa* | 172542 | 172576 | 0 | 3.52E-11 | *_* | *_* |
| R2103 | 35 | P | *Z. caespitosa* | 24396 | 24430 | *Z. caespitosa* | 172542 | 172576 | 0 | 3.52E-11 | *_* | *_* |
| R2104 | 35 | P | *Z. caespitosa* | 148831 | 148865 | *Z. caespitosa* | 162945 | 162979 | -2 | 1.89E-07 | *_* | *_* |
| R2105 | 35 | P | *Z. caespitosa* | 148768 | 148802 | *Z. caespitosa* | 162945 | 162979 | -2 | 1.89E-07 | *_* | *_* |
| R2106 | 35 | P | *Z. caespitosa* | 37821 | 37855 | *Z. caespitosa* | 162945 | 162979 | -1 | 3.70E-09 | *_* | *_* |
| R2107 | 35 | P | *Z. caespitosa* | 149060 | 149094 | *Z. caespitosa* | 157582 | 157616 | -1 | 3.70E-09 | *_* | *rrn26* |
| R2108 | 35 | P | *Z. caespitosa* | 116598 | 116632 | *Z. caespitosa* | 156587 | 156621 | -2 | 1.89E-07 | *_* | *_* |
| R2109 | 35 | P | *Z. caespitosa* | 98149 | 98183 | *Z. caespitosa* | 149709 | 149743 | -1 | 3.70E-09 | *_* | *_* |
| R2110 | 35 | P | *Z. caespitosa* | 57631 | 57665 | *Z. caespitosa* | 149207 | 149241 | 0 | 3.52E-11 | *orf117a* | *_* |
| R2111 | 35 | P | *Z. caespitosa* | 46111 | 46145 | *Z. caespitosa* | 140159 | 140193 | 0 | 3.52E-11 | *_* | *matR* |
| R2112 | 35 | P | *Z. caespitosa* | 43507 | 43541 | *Z. caespitosa* | 140159 | 140193 | 0 | 3.52E-11 | *_* | *matR* |
| R2113 | 35 | P | *Z. caespitosa* | 1559 | 1593 | *Z. caespitosa* | 140159 | 140193 | 0 | 3.52E-11 | *_* | *matR* |
| R2114 | 35 | P | *Z. caespitosa* | 46081 | 46115 | *Z. caespitosa* | 140159 | 140193 | 0 | 3.52E-11 | *_* | *matR* |
| R2115 | 35 | P | *Z. caespitosa* | 4194 | 4228 | *Z. caespitosa* | 140159 | 140193 | 0 | 3.52E-11 | *_* | *matR* |
| R2116 | 35 | P | *Z. caespitosa* | 83648 | 83682 | *Z. caespitosa* | 140159 | 140193 | 0 | 3.52E-11 | *_* | *matR* |
| R2117 | 35 | P | *Z. caespitosa* | 4283 | 4317 | *Z. caespitosa* | 140159 | 140193 | 0 | 3.52E-11 | *_* | *matR* |
| R2118 | 35 | P | *Z. caespitosa* | 83737 | 83771 | *Z. caespitosa* | 140159 | 140193 | 0 | 3.52E-11 | *_* | *matR* |
| R2119 | 35 | P | *Z. caespitosa* | 4372 | 4406 | *Z. caespitosa* | 140159 | 140193 | 0 | 3.52E-11 | *_* | *matR* |
| R2120 | 35 | P | *Z. caespitosa* | 83826 | 83860 | *Z. caespitosa* | 140159 | 140193 | 0 | 3.52E-11 | *_* | *matR* |
| R2121 | 35 | P | *Z. caespitosa* | 4461 | 4495 | *Z. caespitosa* | 140159 | 140193 | 0 | 3.52E-11 | *_* | *matR* |
| R2122 | 35 | P | *Z. caespitosa* | 4135 | 4169 | *Z. caespitosa* | 140159 | 140193 | 0 | 3.52E-11 | *_* | *matR* |
| R2123 | 35 | P | *Z. caespitosa* | 83589 | 83623 | *Z. caespitosa* | 140159 | 140193 | 0 | 3.52E-11 | *_* | *matR* |
| R2124 | 35 | P | *Z. caespitosa* | 4224 | 4258 | *Z. caespitosa* | 140159 | 140193 | 0 | 3.52E-11 | *_* | *matR* |
| R2125 | 35 | P | *Z. caespitosa* | 83678 | 83712 | *Z. caespitosa* | 140159 | 140193 | 0 | 3.52E-11 | *_* | *matR* |
| R2126 | 35 | P | *Z. caespitosa* | 4313 | 4347 | *Z. caespitosa* | 140159 | 140193 | 0 | 3.52E-11 | *_* | *matR* |
| R2127 | 35 | P | *Z. caespitosa* | 83767 | 83801 | *Z. caespitosa* | 140159 | 140193 | 0 | 3.52E-11 | *_* | *matR* |
| R2128 | 35 | P | *Z. caespitosa* | 4402 | 4436 | *Z. caespitosa* | 140159 | 140193 | 0 | 3.52E-11 | *_* | *matR* |
| R2129 | 35 | P | *Z. caespitosa* | 109088 | 109122 | *Z. caespitosa* | 140144 | 140178 | -3 | 6.22E-06 | *orf161a* | *matR* |
| R2130 | 35 | P | *Z. caespitosa* | 95126 | 95160 | *Z. caespitosa* | 140111 | 140145 | 0 | 3.52E-11 | *_* | *matR* |
| R2131 | 35 | P | *Z. caespitosa* | 24396 | 24430 | *Z. caespitosa* | 140111 | 140145 | 0 | 3.52E-11 | *_* | *matR* |
| R2132 | 35 | P | *Z. caespitosa* | 137031 | 137065 | *Z. caespitosa* | 139963 | 139997 | -3 | 6.22E-06 | *_* | *matR* |
| R2133 | 35 | P | *Z. caespitosa* | 51653 | 51687 | *Z. caespitosa* | 139914 | 139948 | -3 | 6.22E-06 | *orf106b;ccmFn* | *matR* |
| R2134 | 35 | P | *Z. caespitosa* | 51713 | 51747 | *Z. caespitosa* | 139809 | 139843 | -2 | 1.89E-07 | *orf106b;ccmFn* | *matR* |
| R2135 | 35 | P | *Z. caespitosa* | 116595 | 116629 | *Z. caespitosa* | 136946 | 136980 | 0 | 3.52E-11 | *_* | *_* |
| R2136 | 35 | P | *Z. caespitosa* | 14657 | 14691 | *Z. caespitosa* | 122181 | 122215 | -3 | 6.22E-06 | *orf295a;cox2* | *nad5* |
| R2137 | 35 | P | *Z. caespitosa* | 35731 | 35765 | *Z. caespitosa* | 120656 | 120690 | 0 | 3.52E-11 | *_* | *nad5;orf399a* |
| R2138 | 35 | P | *Z. caespitosa* | 100042 | 100076 | *Z. caespitosa* | 118829 | 118863 | 0 | 3.52E-11 | *_* | *_* |
| R2139 | 35 | P | *Z. caespitosa* | 75984 | 76018 | *Z. caespitosa* | 118829 | 118863 | -2 | 1.89E-07 | *_* | *_* |
| R2140 | 35 | P | *Z. caespitosa* | 49770 | 49804 | *Z. caespitosa* | 117942 | 117976 | 0 | 3.52E-11 | *_* | *_* |
| R2141 | 35 | P | *Z. caespitosa* | 75921 | 75955 | *Z. caespitosa* | 116595 | 116629 | 0 | 3.52E-11 | *_* | *_* |
| R2142 | 35 | P | *Z. caespitosa* | 72632 | 72666 | *Z. caespitosa* | 116595 | 116629 | 0 | 3.52E-11 | *_* | *_* |
| R2143 | 35 | P | *Z. caespitosa* | 84007 | 84041 | *Z. caespitosa* | 116595 | 116629 | 0 | 3.52E-11 | *_* | *_* |
| R2144 | 35 | P | *Z. caespitosa* | 37754 | 37788 | *Z. caespitosa* | 116595 | 116629 | 0 | 3.52E-11 | *_* | *_* |
| R2145 | 35 | P | *Z. caespitosa* | 14657 | 14691 | *Z. caespitosa* | 115900 | 115934 | -3 | 6.22E-06 | *orf295a;cox2* | *orf540a-1* |
| R2146 | 35 | P | *Z. caespitosa* | 34934 | 34968 | *Z. caespitosa* | 111686 | 111720 | -2 | 1.89E-07 | *_* | *_* |
| R2147 | 35 | P | *Z. caespitosa* | 22162 | 22196 | *Z. caespitosa* | 111680 | 111714 | 0 | 3.52E-11 | *_* | *_* |
| R2148 | 35 | P | *Z. caespitosa* | 16563 | 16597 | *Z. caespitosa* | 109092 | 109126 | -3 | 6.22E-06 | *orf284a* | *orf161a* |
| R2149 | 35 | P | *Z. caespitosa* | 16530 | 16564 | *Z. caespitosa* | 109092 | 109126 | -3 | 6.22E-06 | *orf284a* | *orf161a* |
| R2150 | 35 | P | *Z. caespitosa* | 16497 | 16531 | *Z. caespitosa* | 109092 | 109126 | -3 | 6.22E-06 | *orf284a* | *orf161a* |
| R2151 | 35 | P | *Z. caespitosa* | 16464 | 16498 | *Z. caespitosa* | 109092 | 109126 | -3 | 6.22E-06 | *orf284a* | *orf161a* |
| R2152 | 35 | P | *Z. caespitosa* | 16431 | 16465 | *Z. caespitosa* | 109092 | 109126 | -3 | 6.22E-06 | *orf284a* | *orf161a* |
| R2153 | 35 | P | *Z. caespitosa* | 16398 | 16432 | *Z. caespitosa* | 109092 | 109126 | -3 | 6.22E-06 | *orf284a* | *orf161a* |
| R2154 | 35 | P | *Z. caespitosa* | 16365 | 16399 | *Z. caespitosa* | 109092 | 109126 | -3 | 6.22E-06 | *orf284a* | *orf161a* |
| R2155 | 35 | P | *Z. caespitosa* | 16332 | 16366 | *Z. caespitosa* | 109092 | 109126 | -3 | 6.22E-06 | *orf284a* | *orf161a* |
| R2156 | 35 | P | *Z. caespitosa* | 16299 | 16333 | *Z. caespitosa* | 109092 | 109126 | -3 | 6.22E-06 | *orf284a* | *orf161a* |
| R2157 | 35 | P | *Z. caespitosa* | 16266 | 16300 | *Z. caespitosa* | 109092 | 109126 | -3 | 6.22E-06 | *orf284a* | *orf161a* |
| R2158 | 35 | P | *Z. caespitosa* | 16233 | 16267 | *Z. caespitosa* | 109092 | 109126 | -3 | 6.22E-06 | *orf284a* | *orf161a* |
| R2159 | 35 | P | *Z. caespitosa* | 34934 | 34968 | *Z. caespitosa* | 95738 | 95772 | -2 | 1.89E-07 | *_* | *_* |
| R2160 | 35 | P | *Z. caespitosa* | 87375 | 87409 | *Z. caespitosa* | 95126 | 95160 | 0 | 3.52E-11 | *orf122a* | *_* |
| R2161 | 35 | P | *Z. caespitosa* | 74682 | 74716 | *Z. caespitosa* | 95126 | 95160 | 0 | 3.52E-11 | *_* | *_* |
| R2162 | 35 | P | *Z. caespitosa* | 77798 | 77832 | *Z. caespitosa* | 95126 | 95160 | 0 | 3.52E-11 | *_* | *_* |
| R2163 | 35 | P | *Z. caespitosa* | 77736 | 77770 | *Z. caespitosa* | 95126 | 95160 | 0 | 3.52E-11 | *_* | *_* |
| R2164 | 35 | P | *Z. caespitosa* | 77674 | 77708 | *Z. caespitosa* | 95126 | 95160 | 0 | 3.52E-11 | *_* | *_* |
| R2165 | 35 | P | *Z. caespitosa* | 79456 | 79490 | *Z. caespitosa* | 95126 | 95160 | 0 | 3.52E-11 | *_* | *_* |
| R2166 | 35 | P | *Z. caespitosa* | 24396 | 24430 | *Z. caespitosa* | 87375 | 87409 | 0 | 3.52E-11 | *_* | *orf122a* |
| R2167 | 35 | P | *Z. caespitosa* | 74779 | 74813 | *Z. caespitosa* | 83491 | 83525 | 0 | 3.52E-11 | *_* | *_* |
| R2168 | 35 | P | *Z. caespitosa* | 24396 | 24430 | *Z. caespitosa* | 79456 | 79490 | 0 | 3.52E-11 | *_* | *_* |
| R2169 | 35 | P | *Z. caespitosa* | 34949 | 34983 | *Z. caespitosa* | 77849 | 77883 | -3 | 6.22E-06 | *_* | *_* |
| R2170 | 35 | P | *Z. caespitosa* | 24396 | 24430 | *Z. caespitosa* | 77798 | 77832 | 0 | 3.52E-11 | *_* | *_* |
| R2171 | 35 | P | *Z. caespitosa* | 24396 | 24430 | *Z. caespitosa* | 77736 | 77770 | 0 | 3.52E-11 | *_* | *_* |
| R2172 | 35 | P | *Z. caespitosa* | 24396 | 24430 | *Z. caespitosa* | 77674 | 77708 | 0 | 3.52E-11 | *_* | *_* |
| R2173 | 35 | P | *Z. caespitosa* | 75978 | 76012 | *Z. caespitosa* | 77620 | 77654 | 0 | 3.52E-11 | *_* | *_* |
| R2174 | 35 | P | *Z. caespitosa* | 50022 | 50056 | *Z. caespitosa* | 74746 | 74780 | 0 | 3.52E-11 | *_* | *_* |
| R2175 | 35 | P | *Z. caespitosa* | 24396 | 24430 | *Z. caespitosa* | 74682 | 74716 | 0 | 3.52E-11 | *_* | *_* |
| R2176 | 35 | P | *Z. caespitosa* | 53012 | 53046 | *Z. caespitosa* | 69881 | 69915 | 0 | 3.52E-11 | *_* | *_* |
| R2177 | 35 | P | *Z. caespitosa* | 28832 | 28866 | *Z. caespitosa* | 68800 | 68834 | -3 | 6.22E-06 | *_* | *_* |
| R2178 | 35 | P | *Z. caespitosa* | 16631 | 16665 | *Z. caespitosa* | 53045 | 53079 | -3 | 6.22E-06 | *orf284a* | *_* |
| R2179 | 35 | P | *Z. caespitosa* | 7619 | 7653 | *Z. caespitosa* | 50025 | 50059 | -2 | 1.89E-07 | *_* | *_* |
| R2180 | 35 | P | *Z. caespitosa* | 15986 | 16020 | *Z. caespitosa* | 50025 | 50059 | -2 | 1.89E-07 | *orf284a* | *_* |
| R2181 | 35 | P | *Z. caespitosa* | 29177 | 29211 | *Z. caespitosa* | 41004 | 41038 | -3 | 6.22E-06 | *orf129a* | *orf309a* |
| R2182 | 35 | P | *Z. caespitosa* | 33184 | 33218 | *Z. caespitosa* | 38544 | 38578 | -2 | 1.89E-07 | *_* | *_* |
| R2183 | 35 | P | *Z. caespitosa* | 25008 | 25042 | *Z. caespitosa* | 34934 | 34968 | -2 | 1.89E-07 | *_* | *_* |
| R2184 | 35 | P | *Z. caespitosa* | 14579 | 14613 | *Z. caespitosa* | 34934 | 34968 | -2 | 1.89E-07 | *orf113a* | *_* |
| R2185 | 35 | P | *Z. caespitosa* | 15990 | 16024 | *Z. caespitosa* | 34934 | 34968 | -2 | 1.89E-07 | *orf284a* | *_* |
| R2186 | 35 | P | *Z. caespitosa* | 7623 | 7657 | *Z. caespitosa* | 34934 | 34968 | -2 | 1.89E-07 | *_* | *_* |
| R2187 | 35 | P | *Z. caespitosa* | 15563 | 15597 | *Z. caespitosa* | 34884 | 34918 | -2 | 1.89E-07 | *orf295a* | *_* |
| R2188 | 34 | F | *Z. caespitosa* | 182 | 215 | *Z. caespitosa* | 112103 | 112136 | 0 | 1.41E-10 | *_* | *_* |
| R2189 | 34 | F | *Z. caespitosa* | 135169 | 135202 | *Z. caespitosa* | 177862 | 177895 | 0 | 1.41E-10 | *_* | *_* |
| R2190 | 34 | F | *Z. caespitosa* | 83883 | 83916 | *Z. caespitosa* | 135169 | 135202 | 0 | 1.41E-10 | *_* | *_* |
| R2191 | 34 | F | *Z. caespitosa* | 25003 | 25036 | *Z. caespitosa* | 118830 | 118863 | 0 | 1.41E-10 | *_* | *_* |
| R2192 | 34 | F | *Z. caespitosa* | 95733 | 95766 | *Z. caespitosa* | 118830 | 118863 | 0 | 1.41E-10 | *_* | *_* |
| R2193 | 34 | F | *Z. caespitosa* | 118830 | 118863 | *Z. caespitosa* | 138939 | 138972 | 0 | 1.41E-10 | *_* | *_* |
| R2194 | 34 | F | *Z. caespitosa* | 163044 | 163077 | *Z. caespitosa* | 164757 | 164790 | 0 | 1.41E-10 | *_* | *_* |
| R2195 | 34 | F | *Z. caespitosa* | 101794 | 101827 | *Z. caespitosa* | 188136 | 188169 | 0 | 1.41E-10 | *_* | *_* |
| R2196 | 34 | F | *Z. caespitosa* | 68268 | 68301 | *Z. caespitosa* | 87380 | 87413 | 0 | 1.41E-10 | *nad9* | *orf122a* |
| R2197 | 34 | F | *Z. caespitosa* | 68268 | 68301 | *Z. caespitosa* | 74687 | 74720 | 0 | 1.41E-10 | *nad9* | *_* |
| R2198 | 34 | F | *Z. caespitosa* | 68268 | 68301 | *Z. caespitosa* | 172547 | 172580 | 0 | 1.41E-10 | *nad9* | *_* |
| R2199 | 34 | F | *Z. caespitosa* | 68268 | 68301 | *Z. caespitosa* | 77803 | 77836 | 0 | 1.41E-10 | *nad9* | *_* |
| R2200 | 34 | F | *Z. caespitosa* | 68268 | 68301 | *Z. caespitosa* | 140116 | 140149 | 0 | 1.41E-10 | *nad9* | *matR* |
| R2201 | 34 | F | *Z. caespitosa* | 68268 | 68301 | *Z. caespitosa* | 77741 | 77774 | 0 | 1.41E-10 | *nad9* | *_* |
| R2202 | 34 | F | *Z. caespitosa* | 68268 | 68301 | *Z. caespitosa* | 77679 | 77712 | 0 | 1.41E-10 | *nad9* | *_* |
| R2203 | 34 | F | *Z. caespitosa* | 68268 | 68301 | *Z. caespitosa* | 79461 | 79494 | 0 | 1.41E-10 | *nad9* | *_* |
| R2204 | 34 | F | *Z. caespitosa* | 48412 | 48445 | *Z. caespitosa* | 74687 | 74720 | 0 | 1.41E-10 | *_* | *_* |
| R2205 | 34 | F | *Z. caespitosa* | 48412 | 48445 | *Z. caespitosa* | 172547 | 172580 | 0 | 1.41E-10 | *_* | *_* |
| R2206 | 34 | F | *Z. caespitosa* | 48412 | 48445 | *Z. caespitosa* | 77803 | 77836 | 0 | 1.41E-10 | *_* | *_* |
| R2207 | 34 | F | *Z. caespitosa* | 48412 | 48445 | *Z. caespitosa* | 140116 | 140149 | 0 | 1.41E-10 | *_* | *matR* |
| R2208 | 34 | F | *Z. caespitosa* | 48412 | 48445 | *Z. caespitosa* | 77741 | 77774 | 0 | 1.41E-10 | *_* | *_* |
| R2209 | 34 | F | *Z. caespitosa* | 48412 | 48445 | *Z. caespitosa* | 77679 | 77712 | 0 | 1.41E-10 | *_* | *_* |
| R2210 | 34 | F | *Z. caespitosa* | 48412 | 48445 | *Z. caespitosa* | 79461 | 79494 | 0 | 1.41E-10 | *_* | *_* |
| R2211 | 34 | F | *Z. caespitosa* | 68305 | 68338 | *Z. caespitosa* | 161548 | 161581 | 0 | 1.41E-10 | *_* | *_* |
| R2212 | 34 | F | *Z. caespitosa* | 15874 | 15907 | *Z. caespitosa* | 164648 | 164681 | 0 | 1.41E-10 | *_* | *_* |
| R2213 | 34 | F | *Z. caespitosa* | 40130 | 40163 | *Z. caespitosa* | 116975 | 117008 | -3 | 2.28E-05 | *_* | *_* |
| R2214 | 34 | F | *Z. caespitosa* | 108698 | 108731 | *Z. caespitosa* | 181647 | 181680 | 0 | 1.41E-10 | *orf161a* | *orf103d* |
| R2215 | 34 | F | *Z. caespitosa* | 75917 | 75950 | *Z. caespitosa* | 108698 | 108731 | 0 | 1.41E-10 | *_* | *orf161a* |
| R2216 | 34 | F | *Z. caespitosa* | 108698 | 108731 | *Z. caespitosa* | 184209 | 184242 | 0 | 1.41E-10 | *orf161a* | *orf130b* |
| R2217 | 34 | F | *Z. caespitosa* | 108698 | 108731 | *Z. caespitosa* | 136942 | 136975 | 0 | 1.41E-10 | *orf161a* | *_* |
| R2218 | 34 | F | *Z. caespitosa* | 72628 | 72661 | *Z. caespitosa* | 108698 | 108731 | 0 | 1.41E-10 | *_* | *orf161a* |
| R2219 | 34 | F | *Z. caespitosa* | 108698 | 108731 | *Z. caespitosa* | 177982 | 178015 | 0 | 1.41E-10 | *orf161a* | *_* |
| R2220 | 34 | F | *Z. caespitosa* | 84003 | 84036 | *Z. caespitosa* | 108698 | 108731 | 0 | 1.41E-10 | *_* | *orf161a* |
| R2221 | 34 | F | *Z. caespitosa* | 137055 | 137088 | *Z. caespitosa* | 177657 | 177690 | -3 | 2.28E-05 | *_* | *_* |
| R2222 | 34 | F | *Z. caespitosa* | 137055 | 137088 | *Z. caespitosa* | 180710 | 180743 | -3 | 2.28E-05 | *_* | *_* |
| R2223 | 34 | F | *Z. caespitosa* | 15868 | 15901 | *Z. caespitosa* | 188065 | 188098 | 0 | 1.41E-10 | *_* | *_* |
| R2224 | 34 | F | *Z. caespitosa* | 74746 | 74779 | *Z. caespitosa* | 138943 | 138976 | -2 | 7.11E-07 | *_* | *_* |
| R2225 | 34 | F | *Z. caespitosa* | 16631 | 16664 | *Z. caespitosa* | 138943 | 138976 | -2 | 7.11E-07 | *orf284a* | *_* |
| R2226 | 34 | F | *Z. caespitosa* | 74749 | 74782 | *Z. caespitosa* | 149211 | 149244 | -3 | 2.28E-05 | *_* | *_* |
| R2227 | 34 | F | *Z. caespitosa* | 53047 | 53080 | *Z. caespitosa* | 57632 | 57665 | 0 | 1.41E-10 | *_* | *orf117a* |
| R2228 | 34 | F | *Z. caespitosa* | 75779 | 75812 | *Z. caespitosa* | 84188 | 84221 | 0 | 1.41E-10 | *_* | *_* |
| R2229 | 34 | F | *Z. caespitosa* | 75779 | 75812 | *Z. caespitosa* | 137056 | 137089 | 0 | 1.41E-10 | *_* | *_* |
| R2230 | 34 | F | *Z. caespitosa* | 1560 | 1593 | *Z. caespitosa* | 181667 | 181700 | -3 | 2.28E-05 | *_* | *orf103d* |
| R2231 | 34 | F | *Z. caespitosa* | 1560 | 1593 | *Z. caespitosa* | 75937 | 75970 | -3 | 2.28E-05 | *_* | *_* |
| R2232 | 34 | F | *Z. caespitosa* | 1560 | 1593 | *Z. caespitosa* | 184229 | 184262 | -3 | 2.28E-05 | *_* | *orf130b* |
| R2233 | 34 | F | *Z. caespitosa* | 43508 | 43541 | *Z. caespitosa* | 181667 | 181700 | -3 | 2.28E-05 | *_* | *orf103d* |
| R2234 | 34 | F | *Z. caespitosa* | 43508 | 43541 | *Z. caespitosa* | 75937 | 75970 | -3 | 2.28E-05 | *_* | *_* |
| R2235 | 34 | F | *Z. caespitosa* | 43508 | 43541 | *Z. caespitosa* | 184229 | 184262 | -3 | 2.28E-05 | *_* | *orf130b* |
| R2236 | 34 | F | *Z. caespitosa* | 46112 | 46145 | *Z. caespitosa* | 181667 | 181700 | -3 | 2.28E-05 | *_* | *orf103d* |
| R2237 | 34 | F | *Z. caespitosa* | 46112 | 46145 | *Z. caespitosa* | 75937 | 75970 | -3 | 2.28E-05 | *_* | *_* |
| R2238 | 34 | F | *Z. caespitosa* | 46112 | 46145 | *Z. caespitosa* | 184229 | 184262 | -3 | 2.28E-05 | *_* | *orf130b* |
| R2239 | 34 | F | *Z. caespitosa* | 46082 | 46115 | *Z. caespitosa* | 181667 | 181700 | -3 | 2.28E-05 | *_* | *orf103d* |
| R2240 | 34 | F | *Z. caespitosa* | 46082 | 46115 | *Z. caespitosa* | 75937 | 75970 | -3 | 2.28E-05 | *_* | *_* |
| R2241 | 34 | F | *Z. caespitosa* | 46082 | 46115 | *Z. caespitosa* | 184229 | 184262 | -3 | 2.28E-05 | *_* | *orf130b* |
| R2242 | 34 | F | *Z. caespitosa* | 177687 | 177720 | *Z. caespitosa* | 181667 | 181700 | -3 | 2.28E-05 | *_* | *orf103d* |
| R2243 | 34 | F | *Z. caespitosa* | 75937 | 75970 | *Z. caespitosa* | 177687 | 177720 | -3 | 2.28E-05 | *_* | *_* |
| R2244 | 34 | F | *Z. caespitosa* | 177687 | 177720 | *Z. caespitosa* | 184229 | 184262 | -3 | 2.28E-05 | *_* | *orf130b* |
| R2245 | 34 | F | *Z. caespitosa* | 180740 | 180773 | *Z. caespitosa* | 181667 | 181700 | -3 | 2.28E-05 | *_* | *orf103d* |
| R2246 | 34 | F | *Z. caespitosa* | 75937 | 75970 | *Z. caespitosa* | 180740 | 180773 | -3 | 2.28E-05 | *_* | *_* |
| R2247 | 34 | F | *Z. caespitosa* | 180740 | 180773 | *Z. caespitosa* | 184229 | 184262 | -3 | 2.28E-05 | *_* | *orf130b* |
| R2248 | 34 | F | *Z. caespitosa* | 4195 | 4228 | *Z. caespitosa* | 181667 | 181700 | -3 | 2.28E-05 | *_* | *orf103d* |
| R2249 | 34 | F | *Z. caespitosa* | 4195 | 4228 | *Z. caespitosa* | 75937 | 75970 | -3 | 2.28E-05 | *_* | *_* |
| R2250 | 34 | F | *Z. caespitosa* | 4195 | 4228 | *Z. caespitosa* | 184229 | 184262 | -3 | 2.28E-05 | *_* | *orf130b* |
| R2251 | 34 | F | *Z. caespitosa* | 83649 | 83682 | *Z. caespitosa* | 181667 | 181700 | -3 | 2.28E-05 | *_* | *orf103d* |
| R2252 | 34 | F | *Z. caespitosa* | 75937 | 75970 | *Z. caespitosa* | 83649 | 83682 | -3 | 2.28E-05 | *_* | *_* |
| R2253 | 34 | F | *Z. caespitosa* | 83649 | 83682 | *Z. caespitosa* | 184229 | 184262 | -3 | 2.28E-05 | *_* | *orf130b* |
| R2254 | 34 | F | *Z. caespitosa* | 4284 | 4317 | *Z. caespitosa* | 181667 | 181700 | -3 | 2.28E-05 | *_* | *orf103d* |
| R2255 | 34 | F | *Z. caespitosa* | 4284 | 4317 | *Z. caespitosa* | 75937 | 75970 | -3 | 2.28E-05 | *_* | *_* |
| R2256 | 34 | F | *Z. caespitosa* | 4284 | 4317 | *Z. caespitosa* | 184229 | 184262 | -3 | 2.28E-05 | *_* | *orf130b* |
| R2257 | 34 | F | *Z. caespitosa* | 177717 | 177750 | *Z. caespitosa* | 181667 | 181700 | -3 | 2.28E-05 | *_* | *orf103d* |
| R2258 | 34 | F | *Z. caespitosa* | 75937 | 75970 | *Z. caespitosa* | 177717 | 177750 | -3 | 2.28E-05 | *_* | *_* |
| R2259 | 34 | F | *Z. caespitosa* | 177717 | 177750 | *Z. caespitosa* | 184229 | 184262 | -3 | 2.28E-05 | *_* | *orf130b* |
| R2260 | 34 | F | *Z. caespitosa* | 83738 | 83771 | *Z. caespitosa* | 181667 | 181700 | -3 | 2.28E-05 | *_* | *orf103d* |
| R2261 | 34 | F | *Z. caespitosa* | 75937 | 75970 | *Z. caespitosa* | 83738 | 83771 | -3 | 2.28E-05 | *_* | *_* |
| R2262 | 34 | F | *Z. caespitosa* | 83738 | 83771 | *Z. caespitosa* | 184229 | 184262 | -3 | 2.28E-05 | *_* | *orf130b* |
| R2263 | 34 | F | *Z. caespitosa* | 4373 | 4406 | *Z. caespitosa* | 181667 | 181700 | -3 | 2.28E-05 | *_* | *orf103d* |
| R2264 | 34 | F | *Z. caespitosa* | 4373 | 4406 | *Z. caespitosa* | 75937 | 75970 | -3 | 2.28E-05 | *_* | *_* |
| R2265 | 34 | F | *Z. caespitosa* | 4373 | 4406 | *Z. caespitosa* | 184229 | 184262 | -3 | 2.28E-05 | *_* | *orf130b* |
| R2266 | 34 | F | *Z. caespitosa* | 177806 | 177839 | *Z. caespitosa* | 181667 | 181700 | -3 | 2.28E-05 | *_* | *orf103d* |
| R2267 | 34 | F | *Z. caespitosa* | 75937 | 75970 | *Z. caespitosa* | 177806 | 177839 | -3 | 2.28E-05 | *_* | *_* |
| R2268 | 34 | F | *Z. caespitosa* | 177806 | 177839 | *Z. caespitosa* | 184229 | 184262 | -3 | 2.28E-05 | *_* | *orf130b* |
| R2269 | 34 | F | *Z. caespitosa* | 83827 | 83860 | *Z. caespitosa* | 181667 | 181700 | -3 | 2.28E-05 | *_* | *orf103d* |
| R2270 | 34 | F | *Z. caespitosa* | 75937 | 75970 | *Z. caespitosa* | 83827 | 83860 | -3 | 2.28E-05 | *_* | *_* |
| R2271 | 34 | F | *Z. caespitosa* | 83827 | 83860 | *Z. caespitosa* | 184229 | 184262 | -3 | 2.28E-05 | *_* | *orf130b* |
| R2272 | 34 | F | *Z. caespitosa* | 4462 | 4495 | *Z. caespitosa* | 181667 | 181700 | -3 | 2.28E-05 | *_* | *orf103d* |
| R2273 | 34 | F | *Z. caespitosa* | 4462 | 4495 | *Z. caespitosa* | 75937 | 75970 | -3 | 2.28E-05 | *_* | *_* |
| R2274 | 34 | F | *Z. caespitosa* | 4462 | 4495 | *Z. caespitosa* | 184229 | 184262 | -3 | 2.28E-05 | *_* | *orf130b* |
| R2275 | 34 | F | *Z. caespitosa* | 180770 | 180803 | *Z. caespitosa* | 181667 | 181700 | -3 | 2.28E-05 | *_* | *orf103d* |
| R2276 | 34 | F | *Z. caespitosa* | 75937 | 75970 | *Z. caespitosa* | 180770 | 180803 | -3 | 2.28E-05 | *_* | *_* |
| R2277 | 34 | F | *Z. caespitosa* | 180770 | 180803 | *Z. caespitosa* | 184229 | 184262 | -3 | 2.28E-05 | *_* | *orf130b* |
| R2278 | 34 | F | *Z. caespitosa* | 4136 | 4169 | *Z. caespitosa* | 181667 | 181700 | -3 | 2.28E-05 | *_* | *orf103d* |
| R2279 | 34 | F | *Z. caespitosa* | 4136 | 4169 | *Z. caespitosa* | 75937 | 75970 | -3 | 2.28E-05 | *_* | *_* |
| R2280 | 34 | F | *Z. caespitosa* | 4136 | 4169 | *Z. caespitosa* | 184229 | 184262 | -3 | 2.28E-05 | *_* | *orf130b* |
| R2281 | 34 | F | *Z. caespitosa* | 83590 | 83623 | *Z. caespitosa* | 181667 | 181700 | -3 | 2.28E-05 | *_* | *orf103d* |
| R2282 | 34 | F | *Z. caespitosa* | 75937 | 75970 | *Z. caespitosa* | 83590 | 83623 | -3 | 2.28E-05 | *_* | *_* |
| R2283 | 34 | F | *Z. caespitosa* | 83590 | 83623 | *Z. caespitosa* | 184229 | 184262 | -3 | 2.28E-05 | *_* | *orf130b* |
| R2284 | 34 | F | *Z. caespitosa* | 4225 | 4258 | *Z. caespitosa* | 181667 | 181700 | -3 | 2.28E-05 | *_* | *orf103d* |
| R2285 | 34 | F | *Z. caespitosa* | 4225 | 4258 | *Z. caespitosa* | 75937 | 75970 | -3 | 2.28E-05 | *_* | *_* |
| R2286 | 34 | F | *Z. caespitosa* | 4225 | 4258 | *Z. caespitosa* | 184229 | 184262 | -3 | 2.28E-05 | *_* | *orf130b* |
| R2287 | 34 | F | *Z. caespitosa* | 83679 | 83712 | *Z. caespitosa* | 181667 | 181700 | -3 | 2.28E-05 | *_* | *orf103d* |
| R2288 | 34 | F | *Z. caespitosa* | 75937 | 75970 | *Z. caespitosa* | 83679 | 83712 | -3 | 2.28E-05 | *_* | *_* |
| R2289 | 34 | F | *Z. caespitosa* | 83679 | 83712 | *Z. caespitosa* | 184229 | 184262 | -3 | 2.28E-05 | *_* | *orf130b* |
| R2290 | 34 | F | *Z. caespitosa* | 4314 | 4347 | *Z. caespitosa* | 181667 | 181700 | -3 | 2.28E-05 | *_* | *orf103d* |
| R2291 | 34 | F | *Z. caespitosa* | 4314 | 4347 | *Z. caespitosa* | 75937 | 75970 | -3 | 2.28E-05 | *_* | *_* |
| R2292 | 34 | F | *Z. caespitosa* | 4314 | 4347 | *Z. caespitosa* | 184229 | 184262 | -3 | 2.28E-05 | *_* | *orf130b* |
| R2293 | 34 | F | *Z. caespitosa* | 177747 | 177780 | *Z. caespitosa* | 181667 | 181700 | -3 | 2.28E-05 | *_* | *orf103d* |
| R2294 | 34 | F | *Z. caespitosa* | 75937 | 75970 | *Z. caespitosa* | 177747 | 177780 | -3 | 2.28E-05 | *_* | *_* |
| R2295 | 34 | F | *Z. caespitosa* | 177747 | 177780 | *Z. caespitosa* | 184229 | 184262 | -3 | 2.28E-05 | *_* | *orf130b* |
| R2296 | 34 | F | *Z. caespitosa* | 83768 | 83801 | *Z. caespitosa* | 181667 | 181700 | -3 | 2.28E-05 | *_* | *orf103d* |
| R2297 | 34 | F | *Z. caespitosa* | 75937 | 75970 | *Z. caespitosa* | 83768 | 83801 | -3 | 2.28E-05 | *_* | *_* |
| R2298 | 34 | F | *Z. caespitosa* | 83768 | 83801 | *Z. caespitosa* | 184229 | 184262 | -3 | 2.28E-05 | *_* | *orf130b* |
| R2299 | 34 | F | *Z. caespitosa* | 4403 | 4436 | *Z. caespitosa* | 181667 | 181700 | -3 | 2.28E-05 | *_* | *orf103d* |
| R2300 | 34 | F | *Z. caespitosa* | 4403 | 4436 | *Z. caespitosa* | 75937 | 75970 | -3 | 2.28E-05 | *_* | *_* |
| R2301 | 34 | F | *Z. caespitosa* | 4403 | 4436 | *Z. caespitosa* | 184229 | 184262 | -3 | 2.28E-05 | *_* | *orf130b* |
| R2302 | 34 | F | *Z. caespitosa* | 135046 | 135079 | *Z. caespitosa* | 186474 | 186507 | -3 | 2.28E-05 | *_* | *orf100b* |
| R2303 | 34 | F | *Z. caespitosa* | 135046 | 135079 | *Z. caespitosa* | 181578 | 181611 | -3 | 2.28E-05 | *_* | *orf103d* |
| R2304 | 34 | F | *Z. caespitosa* | 135046 | 135079 | *Z. caespitosa* | 136873 | 136906 | -3 | 2.28E-05 | *_* | *_* |
| R2305 | 34 | F | *Z. caespitosa* | 135046 | 135079 | *Z. caespitosa* | 177913 | 177946 | -3 | 2.28E-05 | *_* | *_* |
| R2306 | 34 | F | *Z. caespitosa* | 83934 | 83967 | *Z. caespitosa* | 135046 | 135079 | -3 | 2.28E-05 | *_* | *_* |
| R2307 | 34 | F | *Z. caespitosa* | 1481 | 1514 | *Z. caespitosa* | 1511 | 1544 | 0 | 1.41E-10 | *_* | *_* |
| R2308 | 34 | F | *Z. caespitosa* | 1481 | 1514 | *Z. caespitosa* | 43459 | 43492 | 0 | 1.41E-10 | *_* | *_* |
| R2309 | 34 | F | *Z. caespitosa* | 1481 | 1514 | *Z. caespitosa* | 46033 | 46066 | 0 | 1.41E-10 | *_* | *_* |
| R2310 | 34 | F | *Z. caespitosa* | 1481 | 1514 | *Z. caespitosa* | 4087 | 4120 | 0 | 1.41E-10 | *_* | *_* |
| R2311 | 34 | F | *Z. caespitosa* | 1481 | 1514 | *Z. caespitosa* | 83541 | 83574 | 0 | 1.41E-10 | *_* | *_* |
| R2312 | 34 | F | *Z. caespitosa* | 1481 | 1514 | *Z. caespitosa* | 177609 | 177642 | 0 | 1.41E-10 | *_* | *_* |
| R2313 | 34 | F | *Z. caespitosa* | 1511 | 1544 | *Z. caespitosa* | 43429 | 43462 | 0 | 1.41E-10 | *_* | *_* |
| R2314 | 34 | F | *Z. caespitosa* | 43429 | 43462 | *Z. caespitosa* | 43459 | 43492 | 0 | 1.41E-10 | *_* | *_* |
| R2315 | 34 | F | *Z. caespitosa* | 43429 | 43462 | *Z. caespitosa* | 46033 | 46066 | 0 | 1.41E-10 | *_* | *_* |
| R2316 | 34 | F | *Z. caespitosa* | 4087 | 4120 | *Z. caespitosa* | 43429 | 43462 | 0 | 1.41E-10 | *_* | *_* |
| R2317 | 34 | F | *Z. caespitosa* | 43429 | 43462 | *Z. caespitosa* | 83541 | 83574 | 0 | 1.41E-10 | *_* | *_* |
| R2318 | 34 | F | *Z. caespitosa* | 43429 | 43462 | *Z. caespitosa* | 177609 | 177642 | 0 | 1.41E-10 | *_* | *_* |
| R2319 | 34 | F | *Z. caespitosa* | 1511 | 1544 | *Z. caespitosa* | 46003 | 46036 | 0 | 1.41E-10 | *_* | *_* |
| R2320 | 34 | F | *Z. caespitosa* | 43459 | 43492 | *Z. caespitosa* | 46003 | 46036 | 0 | 1.41E-10 | *_* | *_* |
| R2321 | 34 | F | *Z. caespitosa* | 46003 | 46036 | *Z. caespitosa* | 46033 | 46066 | 0 | 1.41E-10 | *_* | *_* |
| R2322 | 34 | F | *Z. caespitosa* | 4087 | 4120 | *Z. caespitosa* | 46003 | 46036 | 0 | 1.41E-10 | *_* | *_* |
| R2323 | 34 | F | *Z. caespitosa* | 46003 | 46036 | *Z. caespitosa* | 83541 | 83574 | 0 | 1.41E-10 | *_* | *_* |
| R2324 | 34 | F | *Z. caespitosa* | 46003 | 46036 | *Z. caespitosa* | 177609 | 177642 | 0 | 1.41E-10 | *_* | *_* |
| R2325 | 34 | F | *Z. caespitosa* | 1511 | 1544 | *Z. caespitosa* | 4057 | 4090 | 0 | 1.41E-10 | *_* | *_* |
| R2326 | 34 | F | *Z. caespitosa* | 4057 | 4090 | *Z. caespitosa* | 43459 | 43492 | 0 | 1.41E-10 | *_* | *_* |
| R2327 | 34 | F | *Z. caespitosa* | 4057 | 4090 | *Z. caespitosa* | 46033 | 46066 | 0 | 1.41E-10 | *_* | *_* |
| R2328 | 34 | F | *Z. caespitosa* | 4057 | 4090 | *Z. caespitosa* | 4087 | 4120 | 0 | 1.41E-10 | *_* | *_* |
| R2329 | 34 | F | *Z. caespitosa* | 4057 | 4090 | *Z. caespitosa* | 83541 | 83574 | 0 | 1.41E-10 | *_* | *_* |
| R2330 | 34 | F | *Z. caespitosa* | 4057 | 4090 | *Z. caespitosa* | 177609 | 177642 | 0 | 1.41E-10 | *_* | *_* |
| R2331 | 34 | F | *Z. caespitosa* | 100043 | 100076 | *Z. caespitosa* | 175069 | 175102 | 0 | 1.41E-10 | *_* | *_* |
| R2332 | 34 | F | *Z. caespitosa* | 53051 | 53084 | *Z. caespitosa* | 100043 | 100076 | 0 | 1.41E-10 | *_* | *_* |
| R2333 | 34 | F | *Z. caespitosa* | 50025 | 50058 | *Z. caespitosa* | 100040 | 100073 | -2 | 7.11E-07 | *_* | *_* |
| R2334 | 34 | F | *Z. caespitosa* | 50025 | 50058 | *Z. caespitosa* | 115987 | 116020 | -2 | 7.11E-07 | *_* | *_* |
| R2335 | 34 | F | *Z. caespitosa* | 22094 | 22127 | *Z. caespitosa* | 55106 | 55139 | -3 | 2.28E-05 | *_* | *_* |
| R2336 | 34 | F | *Z. caespitosa* | 55106 | 55139 | *Z. caespitosa* | 180584 | 180617 | -3 | 2.28E-05 | *_* | *_* |
| R2337 | 34 | F | *Z. caespitosa* | 50025 | 50058 | *Z. caespitosa* | 75982 | 76015 | -2 | 7.11E-07 | *_* | *_* |
| R2338 | 34 | F | *Z. caespitosa* | 75985 | 76018 | *Z. caespitosa* | 175069 | 175102 | -2 | 7.11E-07 | *_* | *_* |
| R2339 | 34 | F | *Z. caespitosa* | 53051 | 53084 | *Z. caespitosa* | 75985 | 76018 | -2 | 7.11E-07 | *_* | *_* |
| R2340 | 34 | F | *Z. caespitosa* | 34938 | 34971 | *Z. caespitosa* | 109074 | 109107 | -3 | 2.28E-05 | *_* | *orf161a* |
| R2341 | 34 | F | *Z. caespitosa* | 16077 | 16110 | *Z. caespitosa* | 111680 | 111713 | 0 | 1.41E-10 | *orf284a* | *_* |
| R2342 | 34 | F | *Z. caespitosa* | 83378 | 83411 | *Z. caespitosa* | 111680 | 111713 | 0 | 1.41E-10 | *_* | *_* |
| R2343 | 34 | F | *Z. caespitosa* | 48643 | 48676 | *Z. caespitosa* | 139810 | 139843 | -2 | 7.11E-07 | *_* | *matR* |
| R2344 | 34 | F | *Z. caespitosa* | 87443 | 87476 | *Z. caespitosa* | 118898 | 118931 | -3 | 2.28E-05 | *orf122a* | *_* |
| R2345 | 34 | F | *Z. caespitosa* | 56180 | 56213 | *Z. caespitosa* | 73526 | 73559 | -3 | 2.28E-05 | *rrn18* | *_* |
| R2346 | 34 | F | *Z. caespitosa* | 107747 | 107780 | *Z. caespitosa* | 113574 | 113607 | 0 | 1.41E-10 | *orf177a* | *_* |
| R2347 | 34 | F | *Z. caespitosa* | 89211 | 89244 | *Z. caespitosa* | 169356 | 169389 | -2 | 7.11E-07 | *_* | *_* |
| R2348 | 34 | F | *Z. caespitosa* | 89216 | 89249 | *Z. caespitosa* | 107747 | 107780 | -2 | 7.11E-07 | *_* | *orf177a* |
| R2349 | 34 | F | *Z. caespitosa* | 149710 | 149743 | *Z. caespitosa* | 169361 | 169394 | -1 | 1.44E-08 | *_* | *_* |
| R2350 | 34 | F | *Z. caespitosa* | 107747 | 107780 | *Z. caespitosa* | 149710 | 149743 | -1 | 1.44E-08 | *orf177a* | *_* |
| R2351 | 34 | F | *Z. caespitosa* | 149207 | 149240 | *Z. caespitosa* | 172851 | 172884 | 0 | 1.41E-10 | *_* | *orf185a* |
| R2352 | 34 | F | *Z. caespitosa* | 16081 | 16114 | *Z. caespitosa* | 69882 | 69915 | 0 | 1.41E-10 | *orf284a* | *_* |
| R2353 | 34 | F | *Z. caespitosa* | 9844 | 9877 | *Z. caespitosa* | 10582 | 10615 | -3 | 2.28E-05 | *orf397a* | *_* |
| R2354 | 34 | F | *Z. caespitosa* | 69587 | 69620 | *Z. caespitosa* | 127390 | 127423 | 0 | 1.41E-10 | *_* | *_* |
| R2355 | 34 | F | *Z. caespitosa* | 164702 | 164735 | *Z. caespitosa* | 172685 | 172718 | 0 | 1.41E-10 | *_* | *orf185a* |
| R2356 | 34 | F | *Z. caespitosa* | 108725 | 108758 | *Z. caespitosa* | 137001 | 137034 | 0 | 1.41E-10 | *orf161a* | *_* |
| R2357 | 34 | F | *Z. caespitosa* | 137001 | 137034 | *Z. caespitosa* | 148726 | 148759 | 0 | 1.41E-10 | *_* | *_* |
| R2358 | 34 | F | *Z. caespitosa* | 125155 | 125188 | *Z. caespitosa* | 159728 | 159761 | -3 | 2.28E-05 | *_* | *rrn26* |
| R2359 | 34 | F | *Z. caespitosa* | 15758 | 15791 | *Z. caespitosa* | 48630 | 48663 | -3 | 2.28E-05 | *_* | *_* |
| R2360 | 34 | F | *Z. caespitosa* | 116600 | 116633 | *Z. caespitosa* | 139959 | 139992 | -3 | 2.28E-05 | *_* | *matR* |
| R2361 | 34 | F | *Z. caespitosa* | 77850 | 77883 | *Z. caespitosa* | 172949 | 172982 | -3 | 2.28E-05 | *_* | *orf185a* |
| R2362 | 34 | F | *Z. caespitosa* | 61328 | 61361 | *Z. caespitosa* | 175166 | 175199 | -3 | 2.28E-05 | *orf189a* | *_* |
| R2363 | 34 | F | *Z. caespitosa* | 34949 | 34982 | *Z. caespitosa* | 186496 | 186529 | -2 | 7.11E-07 | *_* | *orf100b* |
| R2364 | 34 | F | *Z. caespitosa* | 34248 | 34281 | *Z. caespitosa* | 181600 | 181633 | -3 | 2.28E-05 | *_* | *orf103d* |
| R2365 | 34 | F | *Z. caespitosa* | 55123 | 55156 | *Z. caespitosa* | 181600 | 181633 | -3 | 2.28E-05 | *_* | *orf103d* |
| R2366 | 34 | F | *Z. caespitosa* | 34248 | 34281 | *Z. caespitosa* | 184162 | 184195 | -3 | 2.28E-05 | *_* | *orf130b* |
| R2367 | 34 | F | *Z. caespitosa* | 55123 | 55156 | *Z. caespitosa* | 184162 | 184195 | -3 | 2.28E-05 | *_* | *orf130b* |
| R2368 | 34 | F | *Z. caespitosa* | 34248 | 34281 | *Z. caespitosa* | 136895 | 136928 | -3 | 2.28E-05 | *_* | *_* |
| R2369 | 34 | F | *Z. caespitosa* | 55123 | 55156 | *Z. caespitosa* | 136895 | 136928 | -3 | 2.28E-05 | *_* | *_* |
| R2370 | 34 | F | *Z. caespitosa* | 34248 | 34281 | *Z. caespitosa* | 177935 | 177968 | -3 | 2.28E-05 | *_* | *_* |
| R2371 | 34 | F | *Z. caespitosa* | 55123 | 55156 | *Z. caespitosa* | 177935 | 177968 | -3 | 2.28E-05 | *_* | *_* |
| R2372 | 34 | F | *Z. caespitosa* | 34248 | 34281 | *Z. caespitosa* | 83956 | 83989 | -3 | 2.28E-05 | *_* | *_* |
| R2373 | 34 | F | *Z. caespitosa* | 55123 | 55156 | *Z. caespitosa* | 83956 | 83989 | -3 | 2.28E-05 | *_* | *_* |
| R2374 | 34 | F | *Z. caespitosa* | 92113 | 92146 | *Z. caespitosa* | 190833 | 190866 | -1 | 1.44E-08 | *_* | *_* |
| R2375 | 34 | F | *Z. caespitosa* | 9109 | 9142 | *Z. caespitosa* | 9364 | 9397 | -1 | 1.44E-08 | *orf397a* | *orf397a* |
| R2376 | 34 | F | *Z. caespitosa* | 9109 | 9142 | *Z. caespitosa* | 9300 | 9333 | -1 | 1.44E-08 | *orf397a* | *orf397a* |
| R2377 | 34 | F | *Z. caespitosa* | 9109 | 9142 | *Z. caespitosa* | 9236 | 9269 | -1 | 1.44E-08 | *orf397a* | *orf397a* |
| R2378 | 34 | F | *Z. caespitosa* | 9109 | 9142 | *Z. caespitosa* | 9172 | 9205 | -1 | 1.44E-08 | *orf397a* | *orf397a* |
| R2379 | 34 | F | *Z. caespitosa* | 9109 | 9142 | *Z. caespitosa* | 9428 | 9461 | -1 | 1.44E-08 | *orf397a* | *orf397a* |
| R2380 | 34 | F | *Z. caespitosa* | 9109 | 9142 | *Z. caespitosa* | 9492 | 9525 | -1 | 1.44E-08 | *orf397a* | *orf397a* |
| R2381 | 34 | F | *Z. caespitosa* | 9109 | 9142 | *Z. caespitosa* | 9556 | 9589 | -1 | 1.44E-08 | *orf397a* | *orf397a* |
| R2382 | 34 | F | *Z. caespitosa* | 9109 | 9142 | *Z. caespitosa* | 9684 | 9717 | -1 | 1.44E-08 | *orf397a* | *orf397a* |
| R2383 | 34 | F | *Z. caespitosa* | 9109 | 9142 | *Z. caespitosa* | 9620 | 9653 | -2 | 7.11E-07 | *orf397a* | *orf397a* |
| R2384 | 34 | F | *Z. caespitosa* | 22162 | 22195 | *Z. caespitosa* | 115989 | 116022 | 0 | 1.41E-10 | *_* | *_* |
| R2385 | 34 | F | *Z. caespitosa* | 9109 | 9142 | *Z. caespitosa* | 9748 | 9781 | 0 | 1.41E-10 | *orf397a* | *orf397a* |
| R2386 | 34 | F | *Z. caespitosa* | 77659 | 77692 | *Z. caespitosa* | 140159 | 140192 | -3 | 2.28E-05 | *_* | *matR* |
| R2387 | 34 | F | *Z. caespitosa* | 77659 | 77692 | *Z. caespitosa* | 188179 | 188212 | -3 | 2.28E-05 | *_* | *_* |
| R2388 | 34 | F | *Z. caespitosa* | 92113 | 92146 | *Z. caespitosa* | 98149 | 98182 | 0 | 1.41E-10 | *_* | *_* |
| R2389 | 34 | F | *Z. caespitosa* | 8583 | 8616 | *Z. caespitosa* | 8611 | 8644 | -2 | 7.11E-07 | *_* | *_* |
| R2390 | 34 | F | *Z. caespitosa* | 8537 | 8570 | *Z. caespitosa* | 8583 | 8616 | -2 | 7.11E-07 | *_* | *_* |
| R2391 | 34 | F | *Z. caespitosa* | 9725 | 9758 | *Z. caespitosa* | 9915 | 9948 | -3 | 2.28E-05 | *orf397a* | *orf397a* |
| R2392 | 34 | F | *Z. caespitosa* | 9789 | 9822 | *Z. caespitosa* | 9915 | 9948 | -3 | 2.28E-05 | *orf397a* | *orf397a* |
| R2393 | 34 | F | *Z. caespitosa* | 15808 | 15841 | *Z. caespitosa* | 162584 | 162617 | -2 | 7.11E-07 | *_* | *_* |
| R2394 | 34 | P | *Z. caespitosa* | 107747 | 107780 | *Z. caespitosa* | 190833 | 190866 | -1 | 1.44E-08 | *orf177a* | *_* |
| R2395 | 34 | P | *Z. caespitosa* | 4057 | 4090 | *Z. caespitosa* | 188228 | 188261 | 0 | 1.41E-10 | *_* | *_* |
| R2396 | 34 | P | *Z. caespitosa* | 46003 | 46036 | *Z. caespitosa* | 188228 | 188261 | 0 | 1.41E-10 | *_* | *_* |
| R2397 | 34 | P | *Z. caespitosa* | 43429 | 43462 | *Z. caespitosa* | 188228 | 188261 | 0 | 1.41E-10 | *_* | *_* |
| R2398 | 34 | P | *Z. caespitosa* | 1481 | 1514 | *Z. caespitosa* | 188228 | 188261 | 0 | 1.41E-10 | *_* | *_* |
| R2399 | 34 | P | *Z. caespitosa* | 4520 | 4553 | *Z. caespitosa* | 188065 | 188098 | 0 | 1.41E-10 | *_* | *_* |
| R2400 | 34 | P | *Z. caespitosa* | 46141 | 46174 | *Z. caespitosa* | 188065 | 188098 | 0 | 1.41E-10 | *_* | *_* |
| R2401 | 34 | P | *Z. caespitosa* | 43537 | 43570 | *Z. caespitosa* | 188065 | 188098 | 0 | 1.41E-10 | *_* | *_* |
| R2402 | 34 | P | *Z. caespitosa* | 1589 | 1622 | *Z. caespitosa* | 188065 | 188098 | 0 | 1.41E-10 | *_* | *_* |
| R2403 | 34 | P | *Z. caespitosa* | 48647 | 48680 | *Z. caespitosa* | 186496 | 186529 | -2 | 7.11E-07 | *_* | *orf100b* |
| R2404 | 34 | P | *Z. caespitosa* | 48412 | 48445 | *Z. caespitosa* | 184209 | 184242 | 0 | 1.41E-10 | *_* | *orf130b* |
| R2405 | 34 | P | *Z. caespitosa* | 68268 | 68301 | *Z. caespitosa* | 184209 | 184242 | 0 | 1.41E-10 | *nad9* | *orf130b* |
| R2406 | 34 | P | *Z. caespitosa* | 172949 | 172982 | *Z. caespitosa* | 184162 | 184195 | -3 | 2.28E-05 | *orf185a* | *orf130b* |
| R2407 | 34 | P | *Z. caespitosa* | 48412 | 48445 | *Z. caespitosa* | 181647 | 181680 | 0 | 1.41E-10 | *_* | *orf103d* |
| R2408 | 34 | P | *Z. caespitosa* | 68268 | 68301 | *Z. caespitosa* | 181647 | 181680 | 0 | 1.41E-10 | *nad9* | *orf103d* |
| R2409 | 34 | P | *Z. caespitosa* | 172949 | 172982 | *Z. caespitosa* | 181600 | 181633 | -3 | 2.28E-05 | *orf185a* | *orf103d* |
| R2410 | 34 | P | *Z. caespitosa* | 172527 | 172560 | *Z. caespitosa* | 180770 | 180803 | -3 | 2.28E-05 | *_* | *_* |
| R2411 | 34 | P | *Z. caespitosa* | 172527 | 172560 | *Z. caespitosa* | 180740 | 180773 | -3 | 2.28E-05 | *_* | *_* |
| R2412 | 34 | P | *Z. caespitosa* | 164703 | 164736 | *Z. caespitosa* | 180710 | 180743 | -3 | 2.28E-05 | *_* | *_* |
| R2413 | 34 | P | *Z. caespitosa* | 87443 | 87476 | *Z. caespitosa* | 180584 | 180617 | -3 | 2.28E-05 | *orf122a* | *_* |
| R2414 | 34 | P | *Z. caespitosa* | 48412 | 48445 | *Z. caespitosa* | 177982 | 178015 | 0 | 1.41E-10 | *_* | *_* |
| R2415 | 34 | P | *Z. caespitosa* | 68268 | 68301 | *Z. caespitosa* | 177982 | 178015 | 0 | 1.41E-10 | *nad9* | *_* |
| R2416 | 34 | P | *Z. caespitosa* | 172949 | 172982 | *Z. caespitosa* | 177935 | 177968 | -3 | 2.28E-05 | *orf185a* | *_* |
| R2417 | 34 | P | *Z. caespitosa* | 172527 | 172560 | *Z. caespitosa* | 177806 | 177839 | -3 | 2.28E-05 | *_* | *_* |
| R2418 | 34 | P | *Z. caespitosa* | 172527 | 172560 | *Z. caespitosa* | 177747 | 177780 | -3 | 2.28E-05 | *_* | *_* |
| R2419 | 34 | P | *Z. caespitosa* | 172527 | 172560 | *Z. caespitosa* | 177717 | 177750 | -3 | 2.28E-05 | *_* | *_* |
| R2420 | 34 | P | *Z. caespitosa* | 172527 | 172560 | *Z. caespitosa* | 177687 | 177720 | -3 | 2.28E-05 | *_* | *_* |
| R2421 | 34 | P | *Z. caespitosa* | 164703 | 164736 | *Z. caespitosa* | 177657 | 177690 | -3 | 2.28E-05 | *_* | *_* |
| R2422 | 34 | P | *Z. caespitosa* | 111680 | 111713 | *Z. caespitosa* | 175069 | 175102 | 0 | 1.41E-10 | *_* | *_* |
| R2423 | 34 | P | *Z. caespitosa* | 158632 | 158665 | *Z. caespitosa* | 173227 | 173260 | 0 | 1.41E-10 | *rrn26* | *_* |
| R2424 | 34 | P | *Z. caespitosa* | 83956 | 83989 | *Z. caespitosa* | 172949 | 172982 | -3 | 2.28E-05 | *_* | *orf185a* |
| R2425 | 34 | P | *Z. caespitosa* | 136895 | 136928 | *Z. caespitosa* | 172949 | 172982 | -3 | 2.28E-05 | *_* | *orf185a* |
| R2426 | 34 | P | *Z. caespitosa* | 84188 | 84221 | *Z. caespitosa* | 172685 | 172718 | 0 | 1.41E-10 | *_* | *orf185a* |
| R2427 | 34 | P | *Z. caespitosa* | 137056 | 137089 | *Z. caespitosa* | 172685 | 172718 | 0 | 1.41E-10 | *_* | *orf185a* |
| R2428 | 34 | P | *Z. caespitosa* | 108698 | 108731 | *Z. caespitosa* | 172547 | 172580 | 0 | 1.41E-10 | *orf161a* | *_* |
| R2429 | 34 | P | *Z. caespitosa* | 4403 | 4436 | *Z. caespitosa* | 172527 | 172560 | -3 | 2.28E-05 | *_* | *_* |
| R2430 | 34 | P | *Z. caespitosa* | 83768 | 83801 | *Z. caespitosa* | 172527 | 172560 | -3 | 2.28E-05 | *_* | *_* |
| R2431 | 34 | P | *Z. caespitosa* | 4314 | 4347 | *Z. caespitosa* | 172527 | 172560 | -3 | 2.28E-05 | *_* | *_* |
| R2432 | 34 | P | *Z. caespitosa* | 83679 | 83712 | *Z. caespitosa* | 172527 | 172560 | -3 | 2.28E-05 | *_* | *_* |
| R2433 | 34 | P | *Z. caespitosa* | 4225 | 4258 | *Z. caespitosa* | 172527 | 172560 | -3 | 2.28E-05 | *_* | *_* |
| R2434 | 34 | P | *Z. caespitosa* | 83590 | 83623 | *Z. caespitosa* | 172527 | 172560 | -3 | 2.28E-05 | *_* | *_* |
| R2435 | 34 | P | *Z. caespitosa* | 4136 | 4169 | *Z. caespitosa* | 172527 | 172560 | -3 | 2.28E-05 | *_* | *_* |
| R2436 | 34 | P | *Z. caespitosa* | 4462 | 4495 | *Z. caespitosa* | 172527 | 172560 | -3 | 2.28E-05 | *_* | *_* |
| R2437 | 34 | P | *Z. caespitosa* | 83827 | 83860 | *Z. caespitosa* | 172527 | 172560 | -3 | 2.28E-05 | *_* | *_* |
| R2438 | 34 | P | *Z. caespitosa* | 4373 | 4406 | *Z. caespitosa* | 172527 | 172560 | -3 | 2.28E-05 | *_* | *_* |
| R2439 | 34 | P | *Z. caespitosa* | 83738 | 83771 | *Z. caespitosa* | 172527 | 172560 | -3 | 2.28E-05 | *_* | *_* |
| R2440 | 34 | P | *Z. caespitosa* | 4284 | 4317 | *Z. caespitosa* | 172527 | 172560 | -3 | 2.28E-05 | *_* | *_* |
| R2441 | 34 | P | *Z. caespitosa* | 83649 | 83682 | *Z. caespitosa* | 172527 | 172560 | -3 | 2.28E-05 | *_* | *_* |
| R2442 | 34 | P | *Z. caespitosa* | 4195 | 4228 | *Z. caespitosa* | 172527 | 172560 | -3 | 2.28E-05 | *_* | *_* |
| R2443 | 34 | P | *Z. caespitosa* | 46082 | 46115 | *Z. caespitosa* | 172527 | 172560 | -3 | 2.28E-05 | *_* | *_* |
| R2444 | 34 | P | *Z. caespitosa* | 46112 | 46145 | *Z. caespitosa* | 172527 | 172560 | -3 | 2.28E-05 | *_* | *_* |
| R2445 | 34 | P | *Z. caespitosa* | 43508 | 43541 | *Z. caespitosa* | 172527 | 172560 | -3 | 2.28E-05 | *_* | *_* |
| R2446 | 34 | P | *Z. caespitosa* | 1560 | 1593 | *Z. caespitosa* | 172527 | 172560 | -3 | 2.28E-05 | *_* | *_* |
| R2447 | 34 | P | *Z. caespitosa* | 108725 | 108758 | *Z. caespitosa* | 164757 | 164790 | 0 | 1.41E-10 | *orf161a* | *_* |
| R2448 | 34 | P | *Z. caespitosa* | 148726 | 148759 | *Z. caespitosa* | 164757 | 164790 | 0 | 1.41E-10 | *_* | *_* |
| R2449 | 34 | P | *Z. caespitosa* | 75779 | 75812 | *Z. caespitosa* | 164702 | 164735 | 0 | 1.41E-10 | *_* | *_* |
| R2450 | 34 | P | *Z. caespitosa* | 83879 | 83912 | *Z. caespitosa* | 164648 | 164681 | 0 | 1.41E-10 | *_* | *_* |
| R2451 | 34 | P | *Z. caespitosa* | 4514 | 4547 | *Z. caespitosa* | 164648 | 164681 | 0 | 1.41E-10 | *_* | *_* |
| R2452 | 34 | P | *Z. caespitosa* | 137001 | 137034 | *Z. caespitosa* | 163044 | 163077 | 0 | 1.41E-10 | *_* | *_* |
| R2453 | 34 | P | *Z. caespitosa* | 83883 | 83916 | *Z. caespitosa* | 162876 | 162909 | 0 | 1.41E-10 | *_* | *_* |
| R2454 | 34 | P | *Z. caespitosa* | 53012 | 53045 | *Z. caespitosa* | 149207 | 149240 | 0 | 1.41E-10 | *_* | *_* |
| R2455 | 34 | P | *Z. caespitosa* | 108698 | 108731 | *Z. caespitosa* | 140116 | 140149 | 0 | 1.41E-10 | *orf161a* | *matR* |
| R2456 | 34 | P | *Z. caespitosa* | 24392 | 24425 | *Z. caespitosa* | 139959 | 139992 | -3 | 2.28E-05 | *_* | *matR* |
| R2457 | 34 | P | *Z. caespitosa* | 95122 | 95155 | *Z. caespitosa* | 139959 | 139992 | -3 | 2.28E-05 | *_* | *matR* |
| R2458 | 34 | P | *Z. caespitosa* | 22162 | 22195 | *Z. caespitosa* | 138939 | 138972 | 0 | 1.41E-10 | *_* | *_* |
| R2459 | 34 | P | *Z. caespitosa* | 67462 | 67495 | *Z. caespitosa* | 136974 | 137007 | -2 | 7.11E-07 | *_* | *_* |
| R2460 | 34 | P | *Z. caespitosa* | 48412 | 48445 | *Z. caespitosa* | 136942 | 136975 | 0 | 1.41E-10 | *_* | *_* |
| R2461 | 34 | P | *Z. caespitosa* | 68268 | 68301 | *Z. caespitosa* | 136942 | 136975 | 0 | 1.41E-10 | *nad9* | *_* |
| R2462 | 34 | P | *Z. caespitosa* | 72261 | 72294 | *Z. caespitosa* | 135046 | 135079 | -3 | 2.28E-05 | *_* | *_* |
| R2463 | 34 | P | *Z. caespitosa* | 55106 | 55139 | *Z. caespitosa* | 118898 | 118931 | -3 | 2.28E-05 | *_* | *_* |
| R2464 | 34 | P | *Z. caespitosa* | 115989 | 116022 | *Z. caespitosa* | 118830 | 118863 | 0 | 1.41E-10 | *_* | *_* |
| R2465 | 34 | P | *Z. caespitosa* | 92113 | 92146 | *Z. caespitosa* | 113574 | 113607 | 0 | 1.41E-10 | *_* | *_* |
| R2466 | 34 | P | *Z. caespitosa* | 53051 | 53084 | *Z. caespitosa* | 111680 | 111713 | 0 | 1.41E-10 | *_* | *_* |
| R2467 | 34 | P | *Z. caespitosa* | 50025 | 50058 | *Z. caespitosa* | 111683 | 111716 | -2 | 7.11E-07 | *_* | *_* |
| R2468 | 34 | P | *Z. caespitosa* | 48417 | 48450 | *Z. caespitosa* | 109921 | 109954 | 0 | 1.41E-10 | *_* | *_* |
| R2469 | 34 | P | *Z. caespitosa* | 68268 | 68301 | *Z. caespitosa* | 108698 | 108731 | 0 | 1.41E-10 | *nad9* | *orf161a* |
| R2470 | 34 | P | *Z. caespitosa* | 74687 | 74720 | *Z. caespitosa* | 108698 | 108731 | 0 | 1.41E-10 | *_* | *orf161a* |
| R2471 | 34 | P | *Z. caespitosa* | 77803 | 77836 | *Z. caespitosa* | 108698 | 108731 | 0 | 1.41E-10 | *_* | *orf161a* |
| R2472 | 34 | P | *Z. caespitosa* | 77741 | 77774 | *Z. caespitosa* | 108698 | 108731 | 0 | 1.41E-10 | *_* | *orf161a* |
| R2473 | 34 | P | *Z. caespitosa* | 77679 | 77712 | *Z. caespitosa* | 108698 | 108731 | 0 | 1.41E-10 | *_* | *orf161a* |
| R2474 | 34 | P | *Z. caespitosa* | 79461 | 79494 | *Z. caespitosa* | 108698 | 108731 | 0 | 1.41E-10 | *_* | *orf161a* |
| R2475 | 34 | P | *Z. caespitosa* | 98149 | 98182 | *Z. caespitosa* | 107747 | 107780 | 0 | 1.41E-10 | *_* | *orf177a* |
| R2476 | 34 | P | *Z. caespitosa* | 16077 | 16110 | *Z. caespitosa* | 100043 | 100076 | 0 | 1.41E-10 | *orf284a* | *_* |
| R2477 | 34 | P | *Z. caespitosa* | 83378 | 83411 | *Z. caespitosa* | 100043 | 100076 | 0 | 1.41E-10 | *_* | *_* |
| R2478 | 34 | P | *Z. caespitosa* | 182 | 215 | *Z. caespitosa* | 99620 | 99653 | 0 | 1.41E-10 | *_* | *_* |
| R2479 | 34 | P | *Z. caespitosa* | 22162 | 22195 | *Z. caespitosa* | 95733 | 95766 | 0 | 1.41E-10 | *_* | *_* |
| R2480 | 34 | P | *Z. caespitosa* | 50025 | 50058 | *Z. caespitosa* | 95735 | 95768 | -2 | 7.11E-07 | *_* | *_* |
| R2481 | 34 | P | *Z. caespitosa* | 40130 | 40163 | *Z. caespitosa* | 94747 | 94780 | -3 | 2.28E-05 | *_* | *_* |
| R2482 | 34 | P | *Z. caespitosa* | 89216 | 89249 | *Z. caespitosa* | 92113 | 92146 | -2 | 7.11E-07 | *_* | *_* |
| R2483 | 34 | P | *Z. caespitosa* | 22094 | 22127 | *Z. caespitosa* | 87443 | 87476 | -3 | 2.28E-05 | *_* | *orf122a* |
| R2484 | 34 | P | *Z. caespitosa* | 33114 | 33147 | *Z. caespitosa* | 85283 | 85316 | -2 | 7.11E-07 | *_* | *orf100a* |
| R2485 | 34 | P | *Z. caespitosa* | 48412 | 48445 | *Z. caespitosa* | 84003 | 84036 | 0 | 1.41E-10 | *_* | *_* |
| R2486 | 34 | P | *Z. caespitosa* | 68268 | 68301 | *Z. caespitosa* | 84003 | 84036 | 0 | 1.41E-10 | *nad9* | *_* |
| R2487 | 34 | P | *Z. caespitosa* | 75985 | 76018 | *Z. caespitosa* | 83378 | 83411 | -2 | 7.11E-07 | *_* | *_* |
| R2488 | 34 | P | *Z. caespitosa* | 51782 | 51815 | *Z. caespitosa* | 79504 | 79537 | -1 | 1.44E-08 | *orf106b;ccmFn* | *_* |
| R2489 | 34 | P | *Z. caespitosa* | 33114 | 33147 | *Z. caespitosa* | 77973 | 78006 | -2 | 7.11E-07 | *_* | *_* |
| R2490 | 34 | P | *Z. caespitosa* | 34248 | 34281 | *Z. caespitosa* | 77850 | 77883 | -3 | 2.28E-05 | *_* | *_* |
| R2491 | 34 | P | *Z. caespitosa* | 55123 | 55156 | *Z. caespitosa* | 77850 | 77883 | -3 | 2.28E-05 | *_* | *_* |
| R2492 | 34 | P | *Z. caespitosa* | 33018 | 33051 | *Z. caespitosa* | 77617 | 77650 | -3 | 2.28E-05 | *_* | *_* |
| R2493 | 34 | P | *Z. caespitosa* | 50025 | 50058 | *Z. caespitosa* | 77617 | 77650 | -3 | 2.28E-05 | *_* | *_* |
| R2494 | 34 | P | *Z. caespitosa* | 16077 | 16110 | *Z. caespitosa* | 75985 | 76018 | -2 | 7.11E-07 | *orf284a* | *_* |
| R2495 | 34 | P | *Z. caespitosa* | 48412 | 48445 | *Z. caespitosa* | 75917 | 75950 | 0 | 1.41E-10 | *_* | *_* |
| R2496 | 34 | P | *Z. caespitosa* | 68268 | 68301 | *Z. caespitosa* | 75917 | 75950 | 0 | 1.41E-10 | *nad9* | *_* |
| R2497 | 34 | P | *Z. caespitosa* | 48412 | 48445 | *Z. caespitosa* | 72628 | 72661 | 0 | 1.41E-10 | *_* | *_* |
| R2498 | 34 | P | *Z. caespitosa* | 68268 | 68301 | *Z. caespitosa* | 72628 | 72661 | 0 | 1.41E-10 | *nad9* | *_* |
| R2499 | 34 | P | *Z. caespitosa* | 53047 | 53080 | *Z. caespitosa* | 69882 | 69915 | 0 | 1.41E-10 | *_* | *_* |
| R2500 | 34 | P | *Z. caespitosa* | 37750 | 37783 | *Z. caespitosa* | 68268 | 68301 | 0 | 1.41E-10 | *_* | *nad9* |
| R2501 | 34 | P | *Z. caespitosa* | 16081 | 16114 | *Z. caespitosa* | 57632 | 57665 | 0 | 1.41E-10 | *orf284a* | *orf117a* |
| R2502 | 34 | P | *Z. caespitosa* | 36859 | 36892 | *Z. caespitosa* | 50912 | 50945 | -3 | 2.28E-05 | *_* | *ccmFn* |
| R2503 | 34 | P | *Z. caespitosa* | 14576 | 14609 | *Z. caespitosa* | 50025 | 50058 | -2 | 7.11E-07 | *orf113a* | *_* |
| R2504 | 34 | P | *Z. caespitosa* | 25005 | 25038 | *Z. caespitosa* | 50025 | 50058 | -2 | 7.11E-07 | *_* | *_* |
| R2505 | 34 | P | *Z. caespitosa* | 28787 | 28820 | *Z. caespitosa* | 41379 | 41412 | -3 | 2.28E-05 | *_* | *orf120b* |
| R2506 | 34 | P | *Z. caespitosa* | 29160 | 29193 | *Z. caespitosa* | 41022 | 41055 | -3 | 2.28E-05 | *_* | *orf309a* |
| R2507 | 34 | P | *Z. caespitosa* | 24017 | 24050 | *Z. caespitosa* | 40130 | 40163 | -3 | 2.28E-05 | *_* | *_* |
| R2508 | 34 | P | *Z. caespitosa* | 30221 | 30254 | *Z. caespitosa* | 39956 | 39989 | -3 | 2.28E-05 | *_* | *_* |
| R2509 | 34 | P | *Z. caespitosa* | 7620 | 7653 | *Z. caespitosa* | 33018 | 33051 | 0 | 1.41E-10 | *_* | *_* |
| R2510 | 34 | P | *Z. caespitosa* | 15987 | 16020 | *Z. caespitosa* | 33018 | 33051 | 0 | 1.41E-10 | *orf284a* | *_* |
| R2511 | 34 | P | *Z. caespitosa* | 22162 | 22195 | *Z. caespitosa* | 25003 | 25036 | 0 | 1.41E-10 | *_* | *_* |
| R2512 | 33 | F | *Z. caespitosa* | 40857 | 40889 | *Z. caespitosa* | 127103 | 127135 | -2 | 2.68E-06 | *orf309a* | *orf242a* |
| R2513 | 33 | F | *Z. caespitosa* | 37943 | 37975 | *Z. caespitosa* | 137102 | 137134 | -3 | 8.30E-05 | *_* | *_* |
| R2514 | 33 | F | *Z. caespitosa* | 108825 | 108857 | *Z. caespitosa* | 137102 | 137134 | -3 | 8.30E-05 | *orf161a* | *_* |
| R2515 | 33 | F | *Z. caespitosa* | 84282 | 84314 | *Z. caespitosa* | 137102 | 137134 | -3 | 8.30E-05 | *_* | *_* |
| R2516 | 33 | F | *Z. caespitosa* | 52689 | 52721 | *Z. caespitosa* | 137102 | 137134 | -3 | 8.30E-05 | *orf172a* | *_* |
| R2517 | 33 | F | *Z. caespitosa* | 52958 | 52990 | *Z. caespitosa* | 137102 | 137134 | -3 | 8.30E-05 | *orf172a* | *_* |
| R2518 | 33 | F | *Z. caespitosa* | 53172 | 53204 | *Z. caespitosa* | 137102 | 137134 | -3 | 8.30E-05 | *_* | *_* |
| R2519 | 33 | F | *Z. caespitosa* | 36149 | 36181 | *Z. caespitosa* | 49049 | 49081 | 0 | 5.63E-10 | *_* | *_* |
| R2520 | 33 | F | *Z. caespitosa* | 16078 | 16110 | *Z. caespitosa* | 25003 | 25035 | 0 | 5.63E-10 | *orf284a* | *_* |
| R2521 | 33 | F | *Z. caespitosa* | 16078 | 16110 | *Z. caespitosa* | 95733 | 95765 | 0 | 5.63E-10 | *orf284a* | *_* |
| R2522 | 33 | F | *Z. caespitosa* | 16078 | 16110 | *Z. caespitosa* | 138939 | 138971 | 0 | 5.63E-10 | *orf284a* | *_* |
| R2523 | 33 | F | *Z. caespitosa* | 25003 | 25035 | *Z. caespitosa* | 83379 | 83411 | 0 | 5.63E-10 | *_* | *_* |
| R2524 | 33 | F | *Z. caespitosa* | 83379 | 83411 | *Z. caespitosa* | 95733 | 95765 | 0 | 5.63E-10 | *_* | *_* |
| R2525 | 33 | F | *Z. caespitosa* | 83379 | 83411 | *Z. caespitosa* | 138939 | 138971 | 0 | 5.63E-10 | *_* | *_* |
| R2526 | 33 | F | *Z. caespitosa* | 79696 | 79728 | *Z. caespitosa* | 179980 | 180012 | -3 | 8.30E-05 | *_* | *_* |
| R2527 | 33 | F | *Z. caespitosa* | 33085 | 33117 | *Z. caespitosa* | 84132 | 84164 | -3 | 8.30E-05 | *_* | *_* |
| R2528 | 33 | F | *Z. caespitosa* | 40586 | 40618 | *Z. caespitosa* | 126832 | 126864 | -3 | 8.30E-05 | *orf309a* | *orf242a* |
| R2529 | 33 | F | *Z. caespitosa* | 40206 | 40238 | *Z. caespitosa* | 126452 | 126484 | -2 | 2.68E-06 | *orf309a* | *orf242a* |
| R2530 | 33 | F | *Z. caespitosa* | 27032 | 27064 | *Z. caespitosa* | 85090 | 85122 | 0 | 5.63E-10 | *_* | *_* |
| R2531 | 33 | F | *Z. caespitosa* | 164643 | 164675 | *Z. caespitosa* | 179892 | 179924 | -2 | 2.68E-06 | *_* | *_* |
| R2532 | 33 | F | *Z. caespitosa* | 41841 | 41873 | *Z. caespitosa* | 99362 | 99394 | -2 | 2.68E-06 | *_* | *orf113c-2* |
| R2533 | 33 | F | *Z. caespitosa* | 156586 | 156618 | *Z. caespitosa* | 181647 | 181679 | 0 | 5.63E-10 | *_* | *orf103d* |
| R2534 | 33 | F | *Z. caespitosa* | 75917 | 75949 | *Z. caespitosa* | 156586 | 156618 | 0 | 5.63E-10 | *_* | *_* |
| R2535 | 33 | F | *Z. caespitosa* | 156586 | 156618 | *Z. caespitosa* | 184209 | 184241 | 0 | 5.63E-10 | *_* | *orf130b* |
| R2536 | 33 | F | *Z. caespitosa* | 136942 | 136974 | *Z. caespitosa* | 156586 | 156618 | 0 | 5.63E-10 | *_* | *_* |
| R2537 | 33 | F | *Z. caespitosa* | 72628 | 72660 | *Z. caespitosa* | 156586 | 156618 | 0 | 5.63E-10 | *_* | *_* |
| R2538 | 33 | F | *Z. caespitosa* | 156586 | 156618 | *Z. caespitosa* | 177982 | 178014 | 0 | 5.63E-10 | *_* | *_* |
| R2539 | 33 | F | *Z. caespitosa* | 84003 | 84035 | *Z. caespitosa* | 156586 | 156618 | 0 | 5.63E-10 | *_* | *_* |
| R2540 | 33 | F | *Z. caespitosa* | 109922 | 109954 | *Z. caespitosa* | 156582 | 156614 | -1 | 5.58E-08 | *_* | *_* |
| R2541 | 33 | F | *Z. caespitosa* | 108694 | 108726 | *Z. caespitosa* | 109922 | 109954 | -1 | 5.58E-08 | *orf161a* | *_* |
| R2542 | 33 | F | *Z. caespitosa* | 37746 | 37778 | *Z. caespitosa* | 109922 | 109954 | -1 | 5.58E-08 | *_* | *_* |
| R2543 | 33 | F | *Z. caespitosa* | 51709 | 51741 | *Z. caespitosa* | 186496 | 186528 | -1 | 5.58E-08 | *orf106b;ccmFn* | *orf100b* |
| R2544 | 33 | F | *Z. caespitosa* | 74746 | 74778 | *Z. caespitosa* | 118834 | 118866 | -3 | 8.30E-05 | *_* | *_* |
| R2545 | 33 | F | *Z. caespitosa* | 16631 | 16663 | *Z. caespitosa* | 118834 | 118866 | -3 | 8.30E-05 | *orf284a* | *_* |
| R2546 | 33 | F | *Z. caespitosa* | 74742 | 74774 | *Z. caespitosa* | 172883 | 172915 | -2 | 2.68E-06 | *_* | *orf185a* |
| R2547 | 33 | F | *Z. caespitosa* | 16627 | 16659 | *Z. caespitosa* | 172883 | 172915 | -2 | 2.68E-06 | *orf284a* | *orf185a* |
| R2548 | 33 | F | *Z. caespitosa* | 16627 | 16659 | *Z. caespitosa* | 172848 | 172880 | -2 | 2.68E-06 | *orf284a* | *orf185a* |
| R2549 | 33 | F | *Z. caespitosa* | 25010 | 25042 | *Z. caespitosa* | 77622 | 77654 | -1 | 5.58E-08 | *_* | *_* |
| R2550 | 33 | F | *Z. caespitosa* | 77622 | 77654 | *Z. caespitosa* | 95740 | 95772 | -1 | 5.58E-08 | *_* | *_* |
| R2551 | 33 | F | *Z. caespitosa* | 14581 | 14613 | *Z. caespitosa* | 77622 | 77654 | -1 | 5.58E-08 | *orf113a* | *_* |
| R2552 | 33 | F | *Z. caespitosa* | 77622 | 77654 | *Z. caespitosa* | 111688 | 111720 | -1 | 5.58E-08 | *_* | *_* |
| R2553 | 33 | F | *Z. caespitosa* | 15992 | 16024 | *Z. caespitosa* | 77622 | 77654 | -1 | 5.58E-08 | *orf284a* | *_* |
| R2554 | 33 | F | *Z. caespitosa* | 7625 | 7657 | *Z. caespitosa* | 77622 | 77654 | -1 | 5.58E-08 | *_* | *_* |
| R2555 | 33 | F | *Z. caespitosa* | 61328 | 61360 | *Z. caespitosa* | 137102 | 137134 | -2 | 2.68E-06 | *orf189a* | *_* |
| R2556 | 33 | F | *Z. caespitosa* | 50025 | 50057 | *Z. caespitosa* | 180590 | 180622 | -2 | 2.68E-06 | *_* | *_* |
| R2557 | 33 | F | *Z. caespitosa* | 136962 | 136994 | *Z. caespitosa* | 177836 | 177868 | -2 | 2.68E-06 | *_* | *_* |
| R2558 | 33 | F | *Z. caespitosa* | 84023 | 84055 | *Z. caespitosa* | 177836 | 177868 | -2 | 2.68E-06 | *_* | *_* |
| R2559 | 33 | F | *Z. caespitosa* | 37770 | 37802 | *Z. caespitosa* | 177836 | 177868 | -3 | 8.30E-05 | *_* | *_* |
| R2560 | 33 | F | *Z. caespitosa* | 83857 | 83889 | *Z. caespitosa* | 136962 | 136994 | -2 | 2.68E-06 | *_* | *_* |
| R2561 | 33 | F | *Z. caespitosa* | 83857 | 83889 | *Z. caespitosa* | 84023 | 84055 | -2 | 2.68E-06 | *_* | *_* |
| R2562 | 33 | F | *Z. caespitosa* | 37770 | 37802 | *Z. caespitosa* | 83857 | 83889 | -3 | 8.30E-05 | *_* | *_* |
| R2563 | 33 | F | *Z. caespitosa* | 4492 | 4524 | *Z. caespitosa* | 136962 | 136994 | -2 | 2.68E-06 | *_* | *_* |
| R2564 | 33 | F | *Z. caespitosa* | 4492 | 4524 | *Z. caespitosa* | 84023 | 84055 | -2 | 2.68E-06 | *_* | *_* |
| R2565 | 33 | F | *Z. caespitosa* | 4492 | 4524 | *Z. caespitosa* | 37770 | 37802 | -3 | 8.30E-05 | *_* | *_* |
| R2566 | 33 | F | *Z. caespitosa* | 136962 | 136994 | *Z. caespitosa* | 180800 | 180832 | -2 | 2.68E-06 | *_* | *_* |
| R2567 | 33 | F | *Z. caespitosa* | 84023 | 84055 | *Z. caespitosa* | 180800 | 180832 | -2 | 2.68E-06 | *_* | *_* |
| R2568 | 33 | F | *Z. caespitosa* | 37770 | 37802 | *Z. caespitosa* | 180800 | 180832 | -3 | 8.30E-05 | *_* | *_* |
| R2569 | 33 | F | *Z. caespitosa* | 136962 | 136994 | *Z. caespitosa* | 177658 | 177690 | -2 | 2.68E-06 | *_* | *_* |
| R2570 | 33 | F | *Z. caespitosa* | 84023 | 84055 | *Z. caespitosa* | 177658 | 177690 | -2 | 2.68E-06 | *_* | *_* |
| R2571 | 33 | F | *Z. caespitosa* | 37770 | 37802 | *Z. caespitosa* | 177658 | 177690 | -3 | 8.30E-05 | *_* | *_* |
| R2572 | 33 | F | *Z. caespitosa* | 136962 | 136994 | *Z. caespitosa* | 180711 | 180743 | -2 | 2.68E-06 | *_* | *_* |
| R2573 | 33 | F | *Z. caespitosa* | 84023 | 84055 | *Z. caespitosa* | 180711 | 180743 | -2 | 2.68E-06 | *_* | *_* |
| R2574 | 33 | F | *Z. caespitosa* | 37770 | 37802 | *Z. caespitosa* | 180711 | 180743 | -3 | 8.30E-05 | *_* | *_* |
| R2575 | 33 | F | *Z. caespitosa* | 4166 | 4198 | *Z. caespitosa* | 136962 | 136994 | -2 | 2.68E-06 | *_* | *_* |
| R2576 | 33 | F | *Z. caespitosa* | 4166 | 4198 | *Z. caespitosa* | 84023 | 84055 | -2 | 2.68E-06 | *_* | *_* |
| R2577 | 33 | F | *Z. caespitosa* | 4166 | 4198 | *Z. caespitosa* | 37770 | 37802 | -3 | 8.30E-05 | *_* | *_* |
| R2578 | 33 | F | *Z. caespitosa* | 83620 | 83652 | *Z. caespitosa* | 136962 | 136994 | -2 | 2.68E-06 | *_* | *_* |
| R2579 | 33 | F | *Z. caespitosa* | 83620 | 83652 | *Z. caespitosa* | 84023 | 84055 | -2 | 2.68E-06 | *_* | *_* |
| R2580 | 33 | F | *Z. caespitosa* | 37770 | 37802 | *Z. caespitosa* | 83620 | 83652 | -3 | 8.30E-05 | *_* | *_* |
| R2581 | 33 | F | *Z. caespitosa* | 4255 | 4287 | *Z. caespitosa* | 136962 | 136994 | -2 | 2.68E-06 | *_* | *_* |
| R2582 | 33 | F | *Z. caespitosa* | 4255 | 4287 | *Z. caespitosa* | 84023 | 84055 | -2 | 2.68E-06 | *_* | *_* |
| R2583 | 33 | F | *Z. caespitosa* | 4255 | 4287 | *Z. caespitosa* | 37770 | 37802 | -3 | 8.30E-05 | *_* | *_* |
| R2584 | 33 | F | *Z. caespitosa* | 83709 | 83741 | *Z. caespitosa* | 136962 | 136994 | -2 | 2.68E-06 | *_* | *_* |
| R2585 | 33 | F | *Z. caespitosa* | 83709 | 83741 | *Z. caespitosa* | 84023 | 84055 | -2 | 2.68E-06 | *_* | *_* |
| R2586 | 33 | F | *Z. caespitosa* | 37770 | 37802 | *Z. caespitosa* | 83709 | 83741 | -3 | 8.30E-05 | *_* | *_* |
| R2587 | 33 | F | *Z. caespitosa* | 4344 | 4376 | *Z. caespitosa* | 136962 | 136994 | -2 | 2.68E-06 | *_* | *_* |
| R2588 | 33 | F | *Z. caespitosa* | 4344 | 4376 | *Z. caespitosa* | 84023 | 84055 | -2 | 2.68E-06 | *_* | *_* |
| R2589 | 33 | F | *Z. caespitosa* | 4344 | 4376 | *Z. caespitosa* | 37770 | 37802 | -3 | 8.30E-05 | *_* | *_* |
| R2590 | 33 | F | *Z. caespitosa* | 136962 | 136994 | *Z. caespitosa* | 177777 | 177809 | -2 | 2.68E-06 | *_* | *_* |
| R2591 | 33 | F | *Z. caespitosa* | 84023 | 84055 | *Z. caespitosa* | 177777 | 177809 | -2 | 2.68E-06 | *_* | *_* |
| R2592 | 33 | F | *Z. caespitosa* | 37770 | 37802 | *Z. caespitosa* | 177777 | 177809 | -3 | 8.30E-05 | *_* | *_* |
| R2593 | 33 | F | *Z. caespitosa* | 83798 | 83830 | *Z. caespitosa* | 136962 | 136994 | -2 | 2.68E-06 | *_* | *_* |
| R2594 | 33 | F | *Z. caespitosa* | 83798 | 83830 | *Z. caespitosa* | 84023 | 84055 | -2 | 2.68E-06 | *_* | *_* |
| R2595 | 33 | F | *Z. caespitosa* | 37770 | 37802 | *Z. caespitosa* | 83798 | 83830 | -3 | 8.30E-05 | *_* | *_* |
| R2596 | 33 | F | *Z. caespitosa* | 4433 | 4465 | *Z. caespitosa* | 136962 | 136994 | -2 | 2.68E-06 | *_* | *_* |
| R2597 | 33 | F | *Z. caespitosa* | 4433 | 4465 | *Z. caespitosa* | 84023 | 84055 | -2 | 2.68E-06 | *_* | *_* |
| R2598 | 33 | F | *Z. caespitosa* | 4433 | 4465 | *Z. caespitosa* | 37770 | 37802 | -3 | 8.30E-05 | *_* | *_* |
| R2599 | 33 | F | *Z. caespitosa* | 84188 | 84220 | *Z. caespitosa* | 177836 | 177868 | -3 | 8.30E-05 | *_* | *_* |
| R2600 | 33 | F | *Z. caespitosa* | 83857 | 83889 | *Z. caespitosa* | 84188 | 84220 | -3 | 8.30E-05 | *_* | *_* |
| R2601 | 33 | F | *Z. caespitosa* | 4492 | 4524 | *Z. caespitosa* | 84188 | 84220 | -3 | 8.30E-05 | *_* | *_* |
| R2602 | 33 | F | *Z. caespitosa* | 84188 | 84220 | *Z. caespitosa* | 180800 | 180832 | -3 | 8.30E-05 | *_* | *_* |
| R2603 | 33 | F | *Z. caespitosa* | 84188 | 84220 | *Z. caespitosa* | 177658 | 177690 | -3 | 8.30E-05 | *_* | *_* |
| R2604 | 33 | F | *Z. caespitosa* | 84188 | 84220 | *Z. caespitosa* | 180711 | 180743 | -3 | 8.30E-05 | *_* | *_* |
| R2605 | 33 | F | *Z. caespitosa* | 4166 | 4198 | *Z. caespitosa* | 84188 | 84220 | -3 | 8.30E-05 | *_* | *_* |
| R2606 | 33 | F | *Z. caespitosa* | 83620 | 83652 | *Z. caespitosa* | 84188 | 84220 | -3 | 8.30E-05 | *_* | *_* |
| R2607 | 33 | F | *Z. caespitosa* | 4255 | 4287 | *Z. caespitosa* | 84188 | 84220 | -3 | 8.30E-05 | *_* | *_* |
| R2608 | 33 | F | *Z. caespitosa* | 83709 | 83741 | *Z. caespitosa* | 84188 | 84220 | -3 | 8.30E-05 | *_* | *_* |
| R2609 | 33 | F | *Z. caespitosa* | 4344 | 4376 | *Z. caespitosa* | 84188 | 84220 | -3 | 8.30E-05 | *_* | *_* |
| R2610 | 33 | F | *Z. caespitosa* | 84188 | 84220 | *Z. caespitosa* | 177777 | 177809 | -3 | 8.30E-05 | *_* | *_* |
| R2611 | 33 | F | *Z. caespitosa* | 83798 | 83830 | *Z. caespitosa* | 84188 | 84220 | -3 | 8.30E-05 | *_* | *_* |
| R2612 | 33 | F | *Z. caespitosa* | 4433 | 4465 | *Z. caespitosa* | 84188 | 84220 | -3 | 8.30E-05 | *_* | *_* |
| R2613 | 33 | F | *Z. caespitosa* | 75779 | 75811 | *Z. caespitosa* | 177836 | 177868 | -3 | 8.30E-05 | *_* | *_* |
| R2614 | 33 | F | *Z. caespitosa* | 75779 | 75811 | *Z. caespitosa* | 83857 | 83889 | -3 | 8.30E-05 | *_* | *_* |
| R2615 | 33 | F | *Z. caespitosa* | 4492 | 4524 | *Z. caespitosa* | 75779 | 75811 | -3 | 8.30E-05 | *_* | *_* |
| R2616 | 33 | F | *Z. caespitosa* | 75779 | 75811 | *Z. caespitosa* | 180800 | 180832 | -3 | 8.30E-05 | *_* | *_* |
| R2617 | 33 | F | *Z. caespitosa* | 75779 | 75811 | *Z. caespitosa* | 177658 | 177690 | -3 | 8.30E-05 | *_* | *_* |
| R2618 | 33 | F | *Z. caespitosa* | 75779 | 75811 | *Z. caespitosa* | 180711 | 180743 | -3 | 8.30E-05 | *_* | *_* |
| R2619 | 33 | F | *Z. caespitosa* | 4166 | 4198 | *Z. caespitosa* | 75779 | 75811 | -3 | 8.30E-05 | *_* | *_* |
| R2620 | 33 | F | *Z. caespitosa* | 75779 | 75811 | *Z. caespitosa* | 83620 | 83652 | -3 | 8.30E-05 | *_* | *_* |
| R2621 | 33 | F | *Z. caespitosa* | 4255 | 4287 | *Z. caespitosa* | 75779 | 75811 | -3 | 8.30E-05 | *_* | *_* |
| R2622 | 33 | F | *Z. caespitosa* | 75779 | 75811 | *Z. caespitosa* | 83709 | 83741 | -3 | 8.30E-05 | *_* | *_* |
| R2623 | 33 | F | *Z. caespitosa* | 4344 | 4376 | *Z. caespitosa* | 75779 | 75811 | -3 | 8.30E-05 | *_* | *_* |
| R2624 | 33 | F | *Z. caespitosa* | 75779 | 75811 | *Z. caespitosa* | 177777 | 177809 | -3 | 8.30E-05 | *_* | *_* |
| R2625 | 33 | F | *Z. caespitosa* | 75779 | 75811 | *Z. caespitosa* | 83798 | 83830 | -3 | 8.30E-05 | *_* | *_* |
| R2626 | 33 | F | *Z. caespitosa* | 4433 | 4465 | *Z. caespitosa* | 75779 | 75811 | -3 | 8.30E-05 | *_* | *_* |
| R2627 | 33 | F | *Z. caespitosa* | 108718 | 108750 | *Z. caespitosa* | 177836 | 177868 | -3 | 8.30E-05 | *orf161a* | *_* |
| R2628 | 33 | F | *Z. caespitosa* | 83857 | 83889 | *Z. caespitosa* | 108718 | 108750 | -3 | 8.30E-05 | *_* | *orf161a* |
| R2629 | 33 | F | *Z. caespitosa* | 4492 | 4524 | *Z. caespitosa* | 108718 | 108750 | -3 | 8.30E-05 | *_* | *orf161a* |
| R2630 | 33 | F | *Z. caespitosa* | 108718 | 108750 | *Z. caespitosa* | 180800 | 180832 | -3 | 8.30E-05 | *orf161a* | *_* |
| R2631 | 33 | F | *Z. caespitosa* | 108718 | 108750 | *Z. caespitosa* | 177658 | 177690 | -3 | 8.30E-05 | *orf161a* | *_* |
| R2632 | 33 | F | *Z. caespitosa* | 108718 | 108750 | *Z. caespitosa* | 180711 | 180743 | -3 | 8.30E-05 | *orf161a* | *_* |
| R2633 | 33 | F | *Z. caespitosa* | 4166 | 4198 | *Z. caespitosa* | 108718 | 108750 | -3 | 8.30E-05 | *_* | *orf161a* |
| R2634 | 33 | F | *Z. caespitosa* | 83620 | 83652 | *Z. caespitosa* | 108718 | 108750 | -3 | 8.30E-05 | *_* | *orf161a* |
| R2635 | 33 | F | *Z. caespitosa* | 4255 | 4287 | *Z. caespitosa* | 108718 | 108750 | -3 | 8.30E-05 | *_* | *orf161a* |
| R2636 | 33 | F | *Z. caespitosa* | 83709 | 83741 | *Z. caespitosa* | 108718 | 108750 | -3 | 8.30E-05 | *_* | *orf161a* |
| R2637 | 33 | F | *Z. caespitosa* | 4344 | 4376 | *Z. caespitosa* | 108718 | 108750 | -3 | 8.30E-05 | *_* | *orf161a* |
| R2638 | 33 | F | *Z. caespitosa* | 108718 | 108750 | *Z. caespitosa* | 177777 | 177809 | -3 | 8.30E-05 | *orf161a* | *_* |
| R2639 | 33 | F | *Z. caespitosa* | 83798 | 83830 | *Z. caespitosa* | 108718 | 108750 | -3 | 8.30E-05 | *_* | *orf161a* |
| R2640 | 33 | F | *Z. caespitosa* | 4433 | 4465 | *Z. caespitosa* | 108718 | 108750 | -3 | 8.30E-05 | *_* | *orf161a* |
| R2641 | 33 | F | *Z. caespitosa* | 148719 | 148751 | *Z. caespitosa* | 177836 | 177868 | -3 | 8.30E-05 | *_* | *_* |
| R2642 | 33 | F | *Z. caespitosa* | 83857 | 83889 | *Z. caespitosa* | 148719 | 148751 | -3 | 8.30E-05 | *_* | *_* |
| R2643 | 33 | F | *Z. caespitosa* | 4492 | 4524 | *Z. caespitosa* | 148719 | 148751 | -3 | 8.30E-05 | *_* | *_* |
| R2644 | 33 | F | *Z. caespitosa* | 148719 | 148751 | *Z. caespitosa* | 180800 | 180832 | -3 | 8.30E-05 | *_* | *_* |
| R2645 | 33 | F | *Z. caespitosa* | 148719 | 148751 | *Z. caespitosa* | 177658 | 177690 | -3 | 8.30E-05 | *_* | *_* |
| R2646 | 33 | F | *Z. caespitosa* | 148719 | 148751 | *Z. caespitosa* | 180711 | 180743 | -3 | 8.30E-05 | *_* | *_* |
| R2647 | 33 | F | *Z. caespitosa* | 4166 | 4198 | *Z. caespitosa* | 148719 | 148751 | -3 | 8.30E-05 | *_* | *_* |
| R2648 | 33 | F | *Z. caespitosa* | 83620 | 83652 | *Z. caespitosa* | 148719 | 148751 | -3 | 8.30E-05 | *_* | *_* |
| R2649 | 33 | F | *Z. caespitosa* | 4255 | 4287 | *Z. caespitosa* | 148719 | 148751 | -3 | 8.30E-05 | *_* | *_* |
| R2650 | 33 | F | *Z. caespitosa* | 83709 | 83741 | *Z. caespitosa* | 148719 | 148751 | -3 | 8.30E-05 | *_* | *_* |
| R2651 | 33 | F | *Z. caespitosa* | 4344 | 4376 | *Z. caespitosa* | 148719 | 148751 | -3 | 8.30E-05 | *_* | *_* |
| R2652 | 33 | F | *Z. caespitosa* | 148719 | 148751 | *Z. caespitosa* | 177777 | 177809 | -3 | 8.30E-05 | *_* | *_* |
| R2653 | 33 | F | *Z. caespitosa* | 83798 | 83830 | *Z. caespitosa* | 148719 | 148751 | -3 | 8.30E-05 | *_* | *_* |
| R2654 | 33 | F | *Z. caespitosa* | 4433 | 4465 | *Z. caespitosa* | 148719 | 148751 | -3 | 8.30E-05 | *_* | *_* |
| R2655 | 33 | F | *Z. caespitosa* | 99297 | 99329 | *Z. caespitosa* | 182448 | 182480 | -3 | 8.30E-05 | *orf113c-2* | *orf117c* |
| R2656 | 33 | F | *Z. caespitosa* | 115990 | 116022 | *Z. caespitosa* | 175069 | 175101 | 0 | 5.63E-10 | *_* | *_* |
| R2657 | 33 | F | *Z. caespitosa* | 53051 | 53083 | *Z. caespitosa* | 115990 | 116022 | 0 | 5.63E-10 | *_* | *_* |
| R2658 | 33 | F | *Z. caespitosa* | 75982 | 76014 | *Z. caespitosa* | 180590 | 180622 | -2 | 2.68E-06 | *_* | *_* |
| R2659 | 33 | F | *Z. caespitosa* | 33018 | 33050 | *Z. caespitosa* | 180590 | 180622 | -2 | 2.68E-06 | *_* | *_* |
| R2660 | 33 | F | *Z. caespitosa* | 100040 | 100072 | *Z. caespitosa* | 180590 | 180622 | -2 | 2.68E-06 | *_* | *_* |
| R2661 | 33 | F | *Z. caespitosa* | 115987 | 116019 | *Z. caespitosa* | 180590 | 180622 | -2 | 2.68E-06 | *_* | *_* |
| R2662 | 33 | F | *Z. caespitosa* | 137016 | 137048 | *Z. caespitosa* | 175142 | 175174 | -3 | 8.30E-05 | *_* | *_* |
| R2663 | 33 | F | *Z. caespitosa* | 84210 | 84242 | *Z. caespitosa* | 175142 | 175174 | -3 | 8.30E-05 | *_* | *_* |
| R2664 | 33 | F | *Z. caespitosa* | 9828 | 9860 | *Z. caespitosa* | 9891 | 9923 | -3 | 8.30E-05 | *orf397a* | *orf397a* |
| R2665 | 33 | F | *Z. caespitosa* | 111680 | 111712 | *Z. caespitosa* | 184978 | 185010 | -1 | 5.58E-08 | *_* | *_* |
| R2666 | 33 | F | *Z. caespitosa* | 139774 | 139806 | *Z. caespitosa* | 164647 | 164679 | -2 | 2.68E-06 | *matR* | *_* |
| R2667 | 33 | F | *Z. caespitosa* | 1786 | 1818 | *Z. caespitosa* | 113574 | 113606 | 0 | 5.63E-10 | *_* | *_* |
| R2668 | 33 | F | *Z. caespitosa* | 43734 | 43766 | *Z. caespitosa* | 113574 | 113606 | 0 | 5.63E-10 | *_* | *_* |
| R2669 | 33 | F | *Z. caespitosa* | 15774 | 15806 | *Z. caespitosa* | 139927 | 139959 | -3 | 8.30E-05 | *_* | *matR* |
| R2670 | 33 | F | *Z. caespitosa* | 57685 | 57717 | *Z. caespitosa* | 116133 | 116165 | -1 | 5.58E-08 | *orf117a* | *_* |
| R2671 | 33 | F | *Z. caespitosa* | 77893 | 77925 | *Z. caespitosa* | 162827 | 162859 | -3 | 8.30E-05 | *_* | *_* |
| R2672 | 33 | F | *Z. caespitosa* | 53048 | 53080 | *Z. caespitosa* | 75650 | 75682 | 0 | 5.63E-10 | *_* | *_* |
| R2673 | 33 | F | *Z. caespitosa* | 34983 | 35015 | *Z. caespitosa* | 55162 | 55194 | -2 | 2.68E-06 | *_* | *_* |
| R2674 | 33 | F | *Z. caespitosa* | 16600 | 16632 | *Z. caespitosa* | 179979 | 180011 | -3 | 8.30E-05 | *orf284a* | *_* |
| R2675 | 33 | F | *Z. caespitosa* | 9149 | 9181 | *Z. caespitosa* | 9978 | 10010 | -3 | 8.30E-05 | *orf397a* | *orf397a* |
| R2676 | 33 | F | *Z. caespitosa* | 40449 | 40481 | *Z. caespitosa* | 126695 | 126727 | -2 | 2.68E-06 | *orf309a* | *orf242a* |
| R2677 | 33 | F | *Z. caespitosa* | 1265 | 1297 | *Z. caespitosa* | 152505 | 152537 | -3 | 8.30E-05 | *_* | *_* |
| R2678 | 33 | F | *Z. caespitosa* | 43213 | 43245 | *Z. caespitosa* | 152505 | 152537 | -3 | 8.30E-05 | *_* | *_* |
| R2679 | 33 | F | *Z. caespitosa* | 41085 | 41117 | *Z. caespitosa* | 68497 | 68529 | -3 | 8.30E-05 | *orf309a* | *_* |
| R2680 | 33 | F | *Z. caespitosa* | 37899 | 37931 | *Z. caespitosa* | 51662 | 51694 | -3 | 8.30E-05 | *_* | *orf106b;ccmFn* |
| R2681 | 33 | F | *Z. caespitosa* | 51662 | 51694 | *Z. caespitosa* | 108781 | 108813 | -3 | 8.30E-05 | *orf106b;ccmFn* | *orf161a* |
| R2682 | 33 | F | *Z. caespitosa* | 34240 | 34272 | *Z. caespitosa* | 156639 | 156671 | -2 | 2.68E-06 | *_* | *_* |
| R2683 | 33 | F | *Z. caespitosa* | 55115 | 55147 | *Z. caespitosa* | 156639 | 156671 | -2 | 2.68E-06 | *_* | *_* |
| R2684 | 33 | F | *Z. caespitosa* | 55107 | 55139 | *Z. caespitosa* | 109069 | 109101 | -3 | 8.30E-05 | *_* | *orf161a* |
| R2685 | 33 | F | *Z. caespitosa* | 69984 | 70016 | *Z. caespitosa* | 79638 | 79670 | -2 | 2.68E-06 | *_* | *_* |
| R2686 | 33 | F | *Z. caespitosa* | 69984 | 70016 | *Z. caespitosa* | 162980 | 163012 | -2 | 2.68E-06 | *_* | *_* |
| R2687 | 33 | F | *Z. caespitosa* | 84233 | 84265 | *Z. caespitosa* | 186480 | 186512 | -1 | 5.58E-08 | *_* | *orf100b* |
| R2688 | 33 | F | *Z. caespitosa* | 84233 | 84265 | *Z. caespitosa* | 181584 | 181616 | -1 | 5.58E-08 | *_* | *orf103d* |
| R2689 | 33 | F | *Z. caespitosa* | 84233 | 84265 | *Z. caespitosa* | 136879 | 136911 | -1 | 5.58E-08 | *_* | *_* |
| R2690 | 33 | F | *Z. caespitosa* | 84233 | 84265 | *Z. caespitosa* | 177919 | 177951 | -1 | 5.58E-08 | *_* | *_* |
| R2691 | 33 | F | *Z. caespitosa* | 83940 | 83972 | *Z. caespitosa* | 84233 | 84265 | -1 | 5.58E-08 | *_* | *_* |
| R2692 | 33 | F | *Z. caespitosa* | 34238 | 34270 | *Z. caespitosa* | 109075 | 109107 | -3 | 8.30E-05 | *_* | *orf161a* |
| R2693 | 33 | F | *Z. caespitosa* | 9852 | 9884 | *Z. caespitosa* | 10104 | 10136 | -3 | 8.30E-05 | *orf397a* | *orf397a* |
| R2694 | 33 | F | *Z. caespitosa* | 9725 | 9757 | *Z. caespitosa* | 10104 | 10136 | -3 | 8.30E-05 | *orf397a* | *orf397a* |
| R2695 | 33 | F | *Z. caespitosa* | 9789 | 9821 | *Z. caespitosa* | 10104 | 10136 | -3 | 8.30E-05 | *orf397a* | *orf397a* |
| R2696 | 33 | R | *Z. caespitosa* | 13293 | 13325 | *Z. caespitosa* | 13293 | 13325 | 0 | 5.63E-10 | *_* | *_* |
| R2697 | 33 | P | *Z. caespitosa* | 43734 | 43766 | *Z. caespitosa* | 190834 | 190866 | -1 | 5.58E-08 | *_* | *_* |
| R2698 | 33 | P | *Z. caespitosa* | 1786 | 1818 | *Z. caespitosa* | 190834 | 190866 | -1 | 5.58E-08 | *_* | *_* |
| R2699 | 33 | P | *Z. caespitosa* | 137102 | 137134 | *Z. caespitosa* | 188092 | 188124 | -3 | 8.30E-05 | *_* | *_* |
| R2700 | 33 | P | *Z. caespitosa* | 100044 | 100076 | *Z. caespitosa* | 184978 | 185010 | -1 | 5.58E-08 | *_* | *_* |
| R2701 | 33 | P | *Z. caespitosa* | 75986 | 76018 | *Z. caespitosa* | 184978 | 185010 | -3 | 8.30E-05 | *_* | *_* |
| R2702 | 33 | P | *Z. caespitosa* | 175082 | 175114 | *Z. caespitosa* | 184966 | 184998 | 0 | 5.63E-10 | *_* | *_* |
| R2703 | 33 | P | *Z. caespitosa* | 112427 | 112459 | *Z. caespitosa* | 182448 | 182480 | -3 | 8.30E-05 | *orf113c-1* | *orf117c* |
| R2704 | 33 | P | *Z. caespitosa* | 85203 | 85235 | *Z. caespitosa* | 181450 | 181482 | -1 | 5.58E-08 | *orf100a* | *orf103d* |
| R2705 | 33 | P | *Z. caespitosa* | 77722 | 77754 | *Z. caespitosa* | 180800 | 180832 | -2 | 2.68E-06 | *_* | *_* |
| R2706 | 33 | P | *Z. caespitosa* | 77784 | 77816 | *Z. caespitosa* | 180800 | 180832 | -2 | 2.68E-06 | *_* | *_* |
| R2707 | 33 | P | *Z. caespitosa* | 163052 | 163084 | *Z. caespitosa* | 180800 | 180832 | -3 | 8.30E-05 | *_* | *_* |
| R2708 | 33 | P | *Z. caespitosa* | 172686 | 172718 | *Z. caespitosa* | 180800 | 180832 | -3 | 8.30E-05 | *orf185a* | *_* |
| R2709 | 33 | P | *Z. caespitosa* | 77722 | 77754 | *Z. caespitosa* | 180711 | 180743 | -2 | 2.68E-06 | *_* | *_* |
| R2710 | 33 | P | *Z. caespitosa* | 77784 | 77816 | *Z. caespitosa* | 180711 | 180743 | -2 | 2.68E-06 | *_* | *_* |
| R2711 | 33 | P | *Z. caespitosa* | 163052 | 163084 | *Z. caespitosa* | 180711 | 180743 | -3 | 8.30E-05 | *_* | *_* |
| R2712 | 33 | P | *Z. caespitosa* | 172686 | 172718 | *Z. caespitosa* | 180711 | 180743 | -3 | 8.30E-05 | *orf185a* | *_* |
| R2713 | 33 | P | *Z. caespitosa* | 25006 | 25038 | *Z. caespitosa* | 180590 | 180622 | -2 | 2.68E-06 | *_* | *_* |
| R2714 | 33 | P | *Z. caespitosa* | 95736 | 95768 | *Z. caespitosa* | 180590 | 180622 | -2 | 2.68E-06 | *_* | *_* |
| R2715 | 33 | P | *Z. caespitosa* | 14577 | 14609 | *Z. caespitosa* | 180590 | 180622 | -2 | 2.68E-06 | *orf113a* | *_* |
| R2716 | 33 | P | *Z. caespitosa* | 111684 | 111716 | *Z. caespitosa* | 180590 | 180622 | -2 | 2.68E-06 | *_* | *_* |
| R2717 | 33 | P | *Z. caespitosa* | 15988 | 16020 | *Z. caespitosa* | 180590 | 180622 | -2 | 2.68E-06 | *orf284a* | *_* |
| R2718 | 33 | P | *Z. caespitosa* | 7621 | 7653 | *Z. caespitosa* | 180590 | 180622 | -2 | 2.68E-06 | *_* | *_* |
| R2719 | 33 | P | *Z. caespitosa* | 138942 | 138974 | *Z. caespitosa* | 180590 | 180622 | -2 | 2.68E-06 | *_* | *_* |
| R2720 | 33 | P | *Z. caespitosa* | 137102 | 137134 | *Z. caespitosa* | 179918 | 179950 | -3 | 8.30E-05 | *_* | *_* |
| R2721 | 33 | P | *Z. caespitosa* | 77722 | 77754 | *Z. caespitosa* | 177836 | 177868 | -2 | 2.68E-06 | *_* | *_* |
| R2722 | 33 | P | *Z. caespitosa* | 77784 | 77816 | *Z. caespitosa* | 177836 | 177868 | -2 | 2.68E-06 | *_* | *_* |
| R2723 | 33 | P | *Z. caespitosa* | 163052 | 163084 | *Z. caespitosa* | 177836 | 177868 | -3 | 8.30E-05 | *_* | *_* |
| R2724 | 33 | P | *Z. caespitosa* | 172686 | 172718 | *Z. caespitosa* | 177836 | 177868 | -3 | 8.30E-05 | *orf185a* | *_* |
| R2725 | 33 | P | *Z. caespitosa* | 77722 | 77754 | *Z. caespitosa* | 177777 | 177809 | -2 | 2.68E-06 | *_* | *_* |
| R2726 | 33 | P | *Z. caespitosa* | 77784 | 77816 | *Z. caespitosa* | 177777 | 177809 | -2 | 2.68E-06 | *_* | *_* |
| R2727 | 33 | P | *Z. caespitosa* | 163052 | 163084 | *Z. caespitosa* | 177777 | 177809 | -3 | 8.30E-05 | *_* | *_* |
| R2728 | 33 | P | *Z. caespitosa* | 172686 | 172718 | *Z. caespitosa* | 177777 | 177809 | -3 | 8.30E-05 | *orf185a* | *_* |
| R2729 | 33 | P | *Z. caespitosa* | 77722 | 77754 | *Z. caespitosa* | 177658 | 177690 | -2 | 2.68E-06 | *_* | *_* |
| R2730 | 33 | P | *Z. caespitosa* | 77784 | 77816 | *Z. caespitosa* | 177658 | 177690 | -2 | 2.68E-06 | *_* | *_* |
| R2731 | 33 | P | *Z. caespitosa* | 163052 | 163084 | *Z. caespitosa* | 177658 | 177690 | -3 | 8.30E-05 | *_* | *_* |
| R2732 | 33 | P | *Z. caespitosa* | 172686 | 172718 | *Z. caespitosa* | 177658 | 177690 | -3 | 8.30E-05 | *orf185a* | *_* |
| R2733 | 33 | P | *Z. caespitosa* | 164743 | 164775 | *Z. caespitosa* | 175142 | 175174 | -3 | 8.30E-05 | *_* | *_* |
| R2734 | 33 | P | *Z. caespitosa* | 138939 | 138971 | *Z. caespitosa* | 175069 | 175101 | 0 | 5.63E-10 | *_* | *_* |
| R2735 | 33 | P | *Z. caespitosa* | 95733 | 95765 | *Z. caespitosa* | 175069 | 175101 | 0 | 5.63E-10 | *_* | *_* |
| R2736 | 33 | P | *Z. caespitosa* | 25003 | 25035 | *Z. caespitosa* | 175069 | 175101 | 0 | 5.63E-10 | *_* | *_* |
| R2737 | 33 | P | *Z. caespitosa* | 83857 | 83889 | *Z. caespitosa* | 172686 | 172718 | -3 | 8.30E-05 | *_* | *orf185a* |
| R2738 | 33 | P | *Z. caespitosa* | 4492 | 4524 | *Z. caespitosa* | 172686 | 172718 | -3 | 8.30E-05 | *_* | *orf185a* |
| R2739 | 33 | P | *Z. caespitosa* | 4166 | 4198 | *Z. caespitosa* | 172686 | 172718 | -3 | 8.30E-05 | *_* | *orf185a* |
| R2740 | 33 | P | *Z. caespitosa* | 83620 | 83652 | *Z. caespitosa* | 172686 | 172718 | -3 | 8.30E-05 | *_* | *orf185a* |
| R2741 | 33 | P | *Z. caespitosa* | 4255 | 4287 | *Z. caespitosa* | 172686 | 172718 | -3 | 8.30E-05 | *_* | *orf185a* |
| R2742 | 33 | P | *Z. caespitosa* | 83709 | 83741 | *Z. caespitosa* | 172686 | 172718 | -3 | 8.30E-05 | *_* | *orf185a* |
| R2743 | 33 | P | *Z. caespitosa* | 4344 | 4376 | *Z. caespitosa* | 172686 | 172718 | -3 | 8.30E-05 | *_* | *orf185a* |
| R2744 | 33 | P | *Z. caespitosa* | 83798 | 83830 | *Z. caespitosa* | 172686 | 172718 | -3 | 8.30E-05 | *_* | *orf185a* |
| R2745 | 33 | P | *Z. caespitosa* | 4433 | 4465 | *Z. caespitosa* | 172686 | 172718 | -3 | 8.30E-05 | *_* | *orf185a* |
| R2746 | 33 | P | *Z. caespitosa* | 156586 | 156618 | *Z. caespitosa* | 172548 | 172580 | 0 | 5.63E-10 | *_* | *_* |
| R2747 | 33 | P | *Z. caespitosa* | 155041 | 155073 | *Z. caespitosa* | 166162 | 166194 | -1 | 5.58E-08 | *_* | *_* |
| R2748 | 33 | P | *Z. caespitosa* | 83857 | 83889 | *Z. caespitosa* | 163052 | 163084 | -3 | 8.30E-05 | *_* | *_* |
| R2749 | 33 | P | *Z. caespitosa* | 4492 | 4524 | *Z. caespitosa* | 163052 | 163084 | -3 | 8.30E-05 | *_* | *_* |
| R2750 | 33 | P | *Z. caespitosa* | 4166 | 4198 | *Z. caespitosa* | 163052 | 163084 | -3 | 8.30E-05 | *_* | *_* |
| R2751 | 33 | P | *Z. caespitosa* | 83620 | 83652 | *Z. caespitosa* | 163052 | 163084 | -3 | 8.30E-05 | *_* | *_* |
| R2752 | 33 | P | *Z. caespitosa* | 4255 | 4287 | *Z. caespitosa* | 163052 | 163084 | -3 | 8.30E-05 | *_* | *_* |
| R2753 | 33 | P | *Z. caespitosa* | 83709 | 83741 | *Z. caespitosa* | 163052 | 163084 | -3 | 8.30E-05 | *_* | *_* |
| R2754 | 33 | P | *Z. caespitosa* | 4344 | 4376 | *Z. caespitosa* | 163052 | 163084 | -3 | 8.30E-05 | *_* | *_* |
| R2755 | 33 | P | *Z. caespitosa* | 83798 | 83830 | *Z. caespitosa* | 163052 | 163084 | -3 | 8.30E-05 | *_* | *_* |
| R2756 | 33 | P | *Z. caespitosa* | 4433 | 4465 | *Z. caespitosa* | 163052 | 163084 | -3 | 8.30E-05 | *_* | *_* |
| R2757 | 33 | P | *Z. caespitosa* | 139963 | 139995 | *Z. caespitosa* | 156613 | 156645 | -3 | 8.30E-05 | *matR* | *_* |
| R2758 | 33 | P | *Z. caespitosa* | 68269 | 68301 | *Z. caespitosa* | 156586 | 156618 | 0 | 5.63E-10 | *nad9* | *_* |
| R2759 | 33 | P | *Z. caespitosa* | 74688 | 74720 | *Z. caespitosa* | 156586 | 156618 | 0 | 5.63E-10 | *_* | *_* |
| R2760 | 33 | P | *Z. caespitosa* | 77804 | 77836 | *Z. caespitosa* | 156586 | 156618 | 0 | 5.63E-10 | *_* | *_* |
| R2761 | 33 | P | *Z. caespitosa* | 140117 | 140149 | *Z. caespitosa* | 156586 | 156618 | 0 | 5.63E-10 | *matR* | *_* |
| R2762 | 33 | P | *Z. caespitosa* | 77742 | 77774 | *Z. caespitosa* | 156586 | 156618 | 0 | 5.63E-10 | *_* | *_* |
| R2763 | 33 | P | *Z. caespitosa* | 77680 | 77712 | *Z. caespitosa* | 156586 | 156618 | 0 | 5.63E-10 | *_* | *_* |
| R2764 | 33 | P | *Z. caespitosa* | 79462 | 79494 | *Z. caespitosa* | 156586 | 156618 | 0 | 5.63E-10 | *_* | *_* |
| R2765 | 33 | P | *Z. caespitosa* | 28446 | 28478 | *Z. caespitosa* | 151225 | 151257 | -3 | 8.30E-05 | *orf113b* | *orf268a;orf132a* |
| R2766 | 33 | P | *Z. caespitosa* | 75650 | 75682 | *Z. caespitosa* | 149207 | 149239 | 0 | 5.63E-10 | *_* | *_* |
| R2767 | 33 | P | *Z. caespitosa* | 33106 | 33138 | *Z. caespitosa* | 138956 | 138988 | -3 | 8.30E-05 | *_* | *_* |
| R2768 | 33 | P | *Z. caespitosa* | 53051 | 53083 | *Z. caespitosa* | 138939 | 138971 | 0 | 5.63E-10 | *_* | *_* |
| R2769 | 33 | P | *Z. caespitosa* | 72207 | 72239 | *Z. caespitosa* | 137102 | 137134 | -3 | 8.30E-05 | *_* | *_* |
| R2770 | 33 | P | *Z. caespitosa* | 69984 | 70016 | *Z. caespitosa* | 135066 | 135098 | -2 | 2.68E-06 | *_* | *_* |
| R2771 | 33 | P | *Z. caespitosa* | 16078 | 16110 | *Z. caespitosa* | 115990 | 116022 | 0 | 5.63E-10 | *orf284a* | *_* |
| R2772 | 33 | P | *Z. caespitosa* | 83379 | 83411 | *Z. caespitosa* | 115990 | 116022 | 0 | 5.63E-10 | *_* | *_* |
| R2773 | 33 | P | *Z. caespitosa* | 77622 | 77654 | *Z. caespitosa* | 115983 | 116015 | -1 | 5.58E-08 | *_* | *_* |
| R2774 | 33 | P | *Z. caespitosa* | 41841 | 41873 | *Z. caespitosa* | 112362 | 112394 | -2 | 2.68E-06 | *_* | *orf113c-1* |
| R2775 | 33 | P | *Z. caespitosa* | 87385 | 87417 | *Z. caespitosa* | 109922 | 109954 | -1 | 5.58E-08 | *orf122a* | *_* |
| R2776 | 33 | P | *Z. caespitosa* | 52984 | 53016 | *Z. caespitosa* | 101691 | 101723 | 0 | 5.63E-10 | *_* | *_* |
| R2777 | 33 | P | *Z. caespitosa* | 77622 | 77654 | *Z. caespitosa* | 100036 | 100068 | -1 | 5.58E-08 | *_* | *_* |
| R2778 | 33 | P | *Z. caespitosa* | 1786 | 1818 | *Z. caespitosa* | 98150 | 98182 | 0 | 5.63E-10 | *_* | *_* |
| R2779 | 33 | P | *Z. caespitosa* | 43734 | 43766 | *Z. caespitosa* | 98150 | 98182 | 0 | 5.63E-10 | *_* | *_* |
| R2780 | 33 | P | *Z. caespitosa* | 53051 | 53083 | *Z. caespitosa* | 95733 | 95765 | 0 | 5.63E-10 | *_* | *_* |
| R2781 | 33 | P | *Z. caespitosa* | 57685 | 57717 | *Z. caespitosa* | 95590 | 95622 | -1 | 5.58E-08 | *orf117a* | *_* |
| R2782 | 33 | P | *Z. caespitosa* | 77722 | 77754 | *Z. caespitosa* | 83857 | 83889 | -2 | 2.68E-06 | *_* | *_* |
| R2783 | 33 | P | *Z. caespitosa* | 77784 | 77816 | *Z. caespitosa* | 83857 | 83889 | -2 | 2.68E-06 | *_* | *_* |
| R2784 | 33 | P | *Z. caespitosa* | 77722 | 77754 | *Z. caespitosa* | 83798 | 83830 | -2 | 2.68E-06 | *_* | *_* |
| R2785 | 33 | P | *Z. caespitosa* | 77784 | 77816 | *Z. caespitosa* | 83798 | 83830 | -2 | 2.68E-06 | *_* | *_* |
| R2786 | 33 | P | *Z. caespitosa* | 77722 | 77754 | *Z. caespitosa* | 83709 | 83741 | -2 | 2.68E-06 | *_* | *_* |
| R2787 | 33 | P | *Z. caespitosa* | 77784 | 77816 | *Z. caespitosa* | 83709 | 83741 | -2 | 2.68E-06 | *_* | *_* |
| R2788 | 33 | P | *Z. caespitosa* | 77722 | 77754 | *Z. caespitosa* | 83620 | 83652 | -2 | 2.68E-06 | *_* | *_* |
| R2789 | 33 | P | *Z. caespitosa* | 77784 | 77816 | *Z. caespitosa* | 83620 | 83652 | -2 | 2.68E-06 | *_* | *_* |
| R2790 | 33 | P | *Z. caespitosa* | 4433 | 4465 | *Z. caespitosa* | 77784 | 77816 | -2 | 2.68E-06 | *_* | *_* |
| R2791 | 33 | P | *Z. caespitosa* | 4344 | 4376 | *Z. caespitosa* | 77784 | 77816 | -2 | 2.68E-06 | *_* | *_* |
| R2792 | 33 | P | *Z. caespitosa* | 4255 | 4287 | *Z. caespitosa* | 77784 | 77816 | -2 | 2.68E-06 | *_* | *_* |
| R2793 | 33 | P | *Z. caespitosa* | 4166 | 4198 | *Z. caespitosa* | 77784 | 77816 | -2 | 2.68E-06 | *_* | *_* |
| R2794 | 33 | P | *Z. caespitosa* | 4492 | 4524 | *Z. caespitosa* | 77784 | 77816 | -2 | 2.68E-06 | *_* | *_* |
| R2795 | 33 | P | *Z. caespitosa* | 4433 | 4465 | *Z. caespitosa* | 77722 | 77754 | -2 | 2.68E-06 | *_* | *_* |
| R2796 | 33 | P | *Z. caespitosa* | 4344 | 4376 | *Z. caespitosa* | 77722 | 77754 | -2 | 2.68E-06 | *_* | *_* |
| R2797 | 33 | P | *Z. caespitosa* | 4255 | 4287 | *Z. caespitosa* | 77722 | 77754 | -2 | 2.68E-06 | *_* | *_* |
| R2798 | 33 | P | *Z. caespitosa* | 4166 | 4198 | *Z. caespitosa* | 77722 | 77754 | -2 | 2.68E-06 | *_* | *_* |
| R2799 | 33 | P | *Z. caespitosa* | 4492 | 4524 | *Z. caespitosa* | 77722 | 77754 | -2 | 2.68E-06 | *_* | *_* |
| R2800 | 33 | P | *Z. caespitosa* | 16081 | 16113 | *Z. caespitosa* | 75650 | 75682 | 0 | 5.63E-10 | *orf284a* | *_* |
| R2801 | 33 | P | *Z. caespitosa* | 74742 | 74774 | *Z. caespitosa* | 75653 | 75685 | -2 | 2.68E-06 | *_* | *_* |
| R2802 | 33 | P | *Z. caespitosa* | 16627 | 16659 | *Z. caespitosa* | 75653 | 75685 | -2 | 2.68E-06 | *orf284a* | *_* |
| R2803 | 33 | P | *Z. caespitosa* | 33017 | 33049 | *Z. caespitosa* | 74746 | 74778 | -2 | 2.68E-06 | *_* | *_* |
| R2804 | 33 | P | *Z. caespitosa* | 22159 | 22191 | *Z. caespitosa* | 74746 | 74778 | -3 | 8.30E-05 | *_* | *_* |
| R2805 | 33 | P | *Z. caespitosa* | 57636 | 57668 | *Z. caespitosa* | 74742 | 74774 | -2 | 2.68E-06 | *orf117a* | *_* |
| R2806 | 33 | P | *Z. caespitosa* | 29177 | 29209 | *Z. caespitosa* | 68418 | 68450 | -3 | 8.30E-05 | *orf129a* | *_* |
| R2807 | 33 | P | *Z. caespitosa* | 24860 | 24892 | *Z. caespitosa* | 57685 | 57717 | -1 | 5.58E-08 | *_* | *orf117a* |
| R2808 | 33 | P | *Z. caespitosa* | 16627 | 16659 | *Z. caespitosa* | 57636 | 57668 | -2 | 2.68E-06 | *orf284a* | *orf117a* |
| R2809 | 33 | P | *Z. caespitosa* | 36828 | 36860 | *Z. caespitosa* | 55162 | 55194 | -2 | 2.68E-06 | *_* | *_* |
| R2810 | 33 | P | *Z. caespitosa* | 25003 | 25035 | *Z. caespitosa* | 53051 | 53083 | 0 | 5.63E-10 | *_* | *_* |
| R2811 | 33 | P | *Z. caespitosa* | 16627 | 16659 | *Z. caespitosa* | 53016 | 53048 | -2 | 2.68E-06 | *orf284a* | *_* |
| R2812 | 33 | P | *Z. caespitosa* | 16631 | 16663 | *Z. caespitosa* | 33017 | 33049 | -2 | 2.68E-06 | *orf284a* | *_* |
| R2813 | 33 | P | *Z. caespitosa* | 16631 | 16663 | *Z. caespitosa* | 22159 | 22191 | -3 | 8.30E-05 | *orf284a* | *_* |
| R2814 | 32 | F | *Z. caespitosa* | 151352 | 151383 | *Z. caespitosa* | 182840 | 182871 | -3 | 3.02E-04 | *orf268a* | *_* |
| R2815 | 32 | F | *Z. caespitosa* | 4518 | 4549 | *Z. caespitosa* | 135169 | 135200 | 0 | 2.25E-09 | *_* | *_* |
| R2816 | 32 | F | *Z. caespitosa* | 69631 | 69662 | *Z. caespitosa* | 127434 | 127465 | -3 | 3.02E-04 | *_* | *_* |
| R2817 | 32 | F | *Z. caespitosa* | 13637 | 13668 | *Z. caespitosa* | 13730 | 13761 | -3 | 3.02E-04 | *_* | *_* |
| R2818 | 32 | F | *Z. caespitosa* | 25003 | 25034 | *Z. caespitosa* | 184979 | 185010 | -1 | 2.16E-07 | *_* | *_* |
| R2819 | 32 | F | *Z. caespitosa* | 95733 | 95764 | *Z. caespitosa* | 184979 | 185010 | -1 | 2.16E-07 | *_* | *_* |
| R2820 | 32 | F | *Z. caespitosa* | 138939 | 138970 | *Z. caespitosa* | 184979 | 185010 | -1 | 2.16E-07 | *_* | *_* |
| R2821 | 32 | F | *Z. caespitosa* | 142114 | 142145 | *Z. caespitosa* | 184254 | 184285 | -1 | 2.16E-07 | *orf101b* | *orf130b* |
| R2822 | 32 | F | *Z. caespitosa* | 33035 | 33066 | *Z. caespitosa* | 117759 | 117790 | -2 | 1.01E-05 | *_* | *_* |
| R2823 | 32 | F | *Z. caespitosa* | 53065 | 53096 | *Z. caespitosa* | 117759 | 117790 | -2 | 1.01E-05 | *_* | *_* |
| R2824 | 32 | F | *Z. caespitosa* | 22177 | 22208 | *Z. caespitosa* | 117759 | 117790 | -2 | 1.01E-05 | *_* | *_* |
| R2825 | 32 | F | *Z. caespitosa* | 50027 | 50058 | *Z. caespitosa* | 175068 | 175099 | -1 | 2.16E-07 | *_* | *_* |
| R2826 | 32 | F | *Z. caespitosa* | 74639 | 74670 | *Z. caespitosa* | 188136 | 188167 | 0 | 2.25E-09 | *_* | *_* |
| R2827 | 32 | F | *Z. caespitosa* | 16191 | 16222 | *Z. caespitosa* | 188136 | 188167 | 0 | 2.25E-09 | *orf284a* | *_* |
| R2828 | 32 | F | *Z. caespitosa* | 56308 | 56339 | *Z. caespitosa* | 73655 | 73686 | -3 | 3.02E-04 | *rrn18* | *_* |
| R2829 | 32 | F | *Z. caespitosa* | 15874 | 15905 | *Z. caespitosa* | 139775 | 139806 | -2 | 1.01E-05 | *_* | *matR* |
| R2830 | 32 | F | *Z. caespitosa* | 32922 | 32953 | *Z. caespitosa* | 49050 | 49081 | 0 | 2.25E-09 | *_* | *_* |
| R2831 | 32 | F | *Z. caespitosa* | 16601 | 16632 | *Z. caespitosa* | 135907 | 135938 | -3 | 3.02E-04 | *orf284a* | *_* |
| R2832 | 32 | F | *Z. caespitosa* | 72182 | 72213 | *Z. caespitosa* | 162876 | 162907 | -1 | 2.16E-07 | *_* | *_* |
| R2833 | 32 | F | *Z. caespitosa* | 31974 | 32005 | *Z. caespitosa* | 76041 | 76072 | 0 | 2.25E-09 | *_* | *_* |
| R2834 | 32 | F | *Z. caespitosa* | 27082 | 27113 | *Z. caespitosa* | 138956 | 138987 | -2 | 1.01E-05 | *_* | *_* |
| R2835 | 32 | F | *Z. caespitosa* | 7910 | 7941 | *Z. caespitosa* | 14141 | 14172 | -2 | 1.01E-05 | *_* | *_* |
| R2836 | 32 | F | *Z. caespitosa* | 33143 | 33174 | *Z. caespitosa* | 34976 | 35007 | -1 | 2.16E-07 | *_* | *_* |
| R2837 | 32 | F | *Z. caespitosa* | 57523 | 57554 | *Z. caespitosa* | 136998 | 137029 | -3 | 3.02E-04 | *orf117a* | *_* |
| R2838 | 32 | F | *Z. caespitosa* | 52802 | 52833 | *Z. caespitosa* | 108699 | 108730 | 0 | 2.25E-09 | *orf172a* | *orf161a* |
| R2839 | 32 | F | *Z. caespitosa* | 52802 | 52833 | *Z. caespitosa* | 181648 | 181679 | 0 | 2.25E-09 | *orf172a* | *orf103d* |
| R2840 | 32 | F | *Z. caespitosa* | 52802 | 52833 | *Z. caespitosa* | 75918 | 75949 | 0 | 2.25E-09 | *orf172a* | *_* |
| R2841 | 32 | F | *Z. caespitosa* | 52802 | 52833 | *Z. caespitosa* | 184210 | 184241 | 0 | 2.25E-09 | *orf172a* | *orf130b* |
| R2842 | 32 | F | *Z. caespitosa* | 52802 | 52833 | *Z. caespitosa* | 136943 | 136974 | 0 | 2.25E-09 | *orf172a* | *_* |
| R2843 | 32 | F | *Z. caespitosa* | 52802 | 52833 | *Z. caespitosa* | 72629 | 72660 | 0 | 2.25E-09 | *orf172a* | *_* |
| R2844 | 32 | F | *Z. caespitosa* | 52802 | 52833 | *Z. caespitosa* | 177983 | 178014 | 0 | 2.25E-09 | *orf172a* | *_* |
| R2845 | 32 | F | *Z. caespitosa* | 52802 | 52833 | *Z. caespitosa* | 84004 | 84035 | 0 | 2.25E-09 | *orf172a* | *_* |
| R2846 | 32 | F | *Z. caespitosa* | 37751 | 37782 | *Z. caespitosa* | 52802 | 52833 | 0 | 2.25E-09 | *_* | *orf172a* |
| R2847 | 32 | F | *Z. caespitosa* | 57585 | 57616 | *Z. caespitosa* | 108699 | 108730 | 0 | 2.25E-09 | *orf117a* | *orf161a* |
| R2848 | 32 | F | *Z. caespitosa* | 57585 | 57616 | *Z. caespitosa* | 181648 | 181679 | 0 | 2.25E-09 | *orf117a* | *orf103d* |
| R2849 | 32 | F | *Z. caespitosa* | 57585 | 57616 | *Z. caespitosa* | 75918 | 75949 | 0 | 2.25E-09 | *orf117a* | *_* |
| R2850 | 32 | F | *Z. caespitosa* | 57585 | 57616 | *Z. caespitosa* | 184210 | 184241 | 0 | 2.25E-09 | *orf117a* | *orf130b* |
| R2851 | 32 | F | *Z. caespitosa* | 57585 | 57616 | *Z. caespitosa* | 136943 | 136974 | 0 | 2.25E-09 | *orf117a* | *_* |
| R2852 | 32 | F | *Z. caespitosa* | 57585 | 57616 | *Z. caespitosa* | 72629 | 72660 | 0 | 2.25E-09 | *orf117a* | *_* |
| R2853 | 32 | F | *Z. caespitosa* | 57585 | 57616 | *Z. caespitosa* | 177983 | 178014 | 0 | 2.25E-09 | *orf117a* | *_* |
| R2854 | 32 | F | *Z. caespitosa* | 57585 | 57616 | *Z. caespitosa* | 84004 | 84035 | 0 | 2.25E-09 | *orf117a* | *_* |
| R2855 | 32 | F | *Z. caespitosa* | 37751 | 37782 | *Z. caespitosa* | 57585 | 57616 | 0 | 2.25E-09 | *_* | *orf117a* |
| R2856 | 32 | F | *Z. caespitosa* | 108699 | 108730 | *Z. caespitosa* | 156617 | 156648 | 0 | 2.25E-09 | *orf161a* | *_* |
| R2857 | 32 | F | *Z. caespitosa* | 37751 | 37782 | *Z. caespitosa* | 156617 | 156648 | 0 | 2.25E-09 | *_* | *_* |
| R2858 | 32 | F | *Z. caespitosa* | 75855 | 75886 | *Z. caespitosa* | 181633 | 181664 | -3 | 3.02E-04 | *_* | *orf103d* |
| R2859 | 32 | F | *Z. caespitosa* | 75855 | 75886 | *Z. caespitosa* | 75903 | 75934 | -3 | 3.02E-04 | *_* | *_* |
| R2860 | 32 | F | *Z. caespitosa* | 75855 | 75886 | *Z. caespitosa* | 184195 | 184226 | -3 | 3.02E-04 | *_* | *orf130b* |
| R2861 | 32 | F | *Z. caespitosa* | 75855 | 75886 | *Z. caespitosa* | 136928 | 136959 | -3 | 3.02E-04 | *_* | *_* |
| R2862 | 32 | F | *Z. caespitosa* | 72614 | 72645 | *Z. caespitosa* | 75855 | 75886 | -3 | 3.02E-04 | *_* | *_* |
| R2863 | 32 | F | *Z. caespitosa* | 75855 | 75886 | *Z. caespitosa* | 177968 | 177999 | -3 | 3.02E-04 | *_* | *_* |
| R2864 | 32 | F | *Z. caespitosa* | 75855 | 75886 | *Z. caespitosa* | 83989 | 84020 | -3 | 3.02E-04 | *_* | *_* |
| R2865 | 32 | F | *Z. caespitosa* | 84308 | 84339 | *Z. caespitosa* | 177864 | 177895 | 0 | 2.25E-09 | *_* | *_* |
| R2866 | 32 | F | *Z. caespitosa* | 83885 | 83916 | *Z. caespitosa* | 84308 | 84339 | 0 | 2.25E-09 | *_* | *_* |
| R2867 | 32 | F | *Z. caespitosa* | 108851 | 108882 | *Z. caespitosa* | 135171 | 135202 | 0 | 2.25E-09 | *orf161a* | *_* |
| R2868 | 32 | F | *Z. caespitosa* | 25007 | 25038 | *Z. caespitosa* | 74746 | 74777 | -2 | 1.01E-05 | *_* | *_* |
| R2869 | 32 | F | *Z. caespitosa* | 16631 | 16662 | *Z. caespitosa* | 25007 | 25038 | -2 | 1.01E-05 | *orf284a* | *_* |
| R2870 | 32 | F | *Z. caespitosa* | 74746 | 74777 | *Z. caespitosa* | 95737 | 95768 | -2 | 1.01E-05 | *_* | *_* |
| R2871 | 32 | F | *Z. caespitosa* | 16631 | 16662 | *Z. caespitosa* | 95737 | 95768 | -2 | 1.01E-05 | *orf284a* | *_* |
| R2872 | 32 | F | *Z. caespitosa* | 14578 | 14609 | *Z. caespitosa* | 74746 | 74777 | -2 | 1.01E-05 | *orf113a* | *_* |
| R2873 | 32 | F | *Z. caespitosa* | 14578 | 14609 | *Z. caespitosa* | 16631 | 16662 | -2 | 1.01E-05 | *orf113a* | *orf284a* |
| R2874 | 32 | F | *Z. caespitosa* | 74746 | 74777 | *Z. caespitosa* | 111685 | 111716 | -2 | 1.01E-05 | *_* | *_* |
| R2875 | 32 | F | *Z. caespitosa* | 16631 | 16662 | *Z. caespitosa* | 111685 | 111716 | -2 | 1.01E-05 | *orf284a* | *_* |
| R2876 | 32 | F | *Z. caespitosa* | 15989 | 16020 | *Z. caespitosa* | 74746 | 74777 | -2 | 1.01E-05 | *orf284a* | *_* |
| R2877 | 32 | F | *Z. caespitosa* | 15989 | 16020 | *Z. caespitosa* | 16631 | 16662 | -2 | 1.01E-05 | *orf284a* | *orf284a* |
| R2878 | 32 | F | *Z. caespitosa* | 7622 | 7653 | *Z. caespitosa* | 74746 | 74777 | -2 | 1.01E-05 | *_* | *_* |
| R2879 | 32 | F | *Z. caespitosa* | 7622 | 7653 | *Z. caespitosa* | 16631 | 16662 | -2 | 1.01E-05 | *_* | *orf284a* |
| R2880 | 32 | F | *Z. caespitosa* | 77617 | 77648 | *Z. caespitosa* | 118832 | 118863 | -3 | 3.02E-04 | *_* | *_* |
| R2881 | 32 | F | *Z. caespitosa* | 15987 | 16018 | *Z. caespitosa* | 118832 | 118863 | 0 | 2.25E-09 | *orf284a* | *_* |
| R2882 | 32 | F | *Z. caespitosa* | 7620 | 7651 | *Z. caespitosa* | 118832 | 118863 | 0 | 2.25E-09 | *_* | *_* |
| R2883 | 32 | F | *Z. caespitosa* | 109088 | 109119 | *Z. caespitosa* | 181618 | 181649 | -3 | 3.02E-04 | *orf161a* | *orf103d* |
| R2884 | 32 | F | *Z. caespitosa* | 75888 | 75919 | *Z. caespitosa* | 109088 | 109119 | -3 | 3.02E-04 | *_* | *orf161a* |
| R2885 | 32 | F | *Z. caespitosa* | 109088 | 109119 | *Z. caespitosa* | 184180 | 184211 | -3 | 3.02E-04 | *orf161a* | *orf130b* |
| R2886 | 32 | F | *Z. caespitosa* | 109088 | 109119 | *Z. caespitosa* | 136913 | 136944 | -3 | 3.02E-04 | *orf161a* | *_* |
| R2887 | 32 | F | *Z. caespitosa* | 72599 | 72630 | *Z. caespitosa* | 109088 | 109119 | -3 | 3.02E-04 | *_* | *orf161a* |
| R2888 | 32 | F | *Z. caespitosa* | 109088 | 109119 | *Z. caespitosa* | 177953 | 177984 | -3 | 3.02E-04 | *orf161a* | *_* |
| R2889 | 32 | F | *Z. caespitosa* | 83974 | 84005 | *Z. caespitosa* | 109088 | 109119 | -3 | 3.02E-04 | *_* | *orf161a* |
| R2890 | 32 | F | *Z. caespitosa* | 46096 | 46127 | *Z. caespitosa* | 136946 | 136977 | -3 | 3.02E-04 | *_* | *_* |
| R2891 | 32 | F | *Z. caespitosa* | 46096 | 46127 | *Z. caespitosa* | 72632 | 72663 | -3 | 3.02E-04 | *_* | *_* |
| R2892 | 32 | F | *Z. caespitosa* | 46096 | 46127 | *Z. caespitosa* | 177986 | 178017 | -3 | 3.02E-04 | *_* | *_* |
| R2893 | 32 | F | *Z. caespitosa* | 46096 | 46127 | *Z. caespitosa* | 84007 | 84038 | -3 | 3.02E-04 | *_* | *_* |
| R2894 | 32 | F | *Z. caespitosa* | 37754 | 37785 | *Z. caespitosa* | 46096 | 46127 | -3 | 3.02E-04 | *_* | *_* |
| R2895 | 32 | F | *Z. caespitosa* | 136946 | 136977 | *Z. caespitosa* | 177701 | 177732 | -3 | 3.02E-04 | *_* | *_* |
| R2896 | 32 | F | *Z. caespitosa* | 72632 | 72663 | *Z. caespitosa* | 177701 | 177732 | -3 | 3.02E-04 | *_* | *_* |
| R2897 | 32 | F | *Z. caespitosa* | 177701 | 177732 | *Z. caespitosa* | 177986 | 178017 | -3 | 3.02E-04 | *_* | *_* |
| R2898 | 32 | F | *Z. caespitosa* | 84007 | 84038 | *Z. caespitosa* | 177701 | 177732 | -3 | 3.02E-04 | *_* | *_* |
| R2899 | 32 | F | *Z. caespitosa* | 37754 | 37785 | *Z. caespitosa* | 177701 | 177732 | -3 | 3.02E-04 | *_* | *_* |
| R2900 | 32 | F | *Z. caespitosa* | 136946 | 136977 | *Z. caespitosa* | 180754 | 180785 | -3 | 3.02E-04 | *_* | *_* |
| R2901 | 32 | F | *Z. caespitosa* | 72632 | 72663 | *Z. caespitosa* | 180754 | 180785 | -3 | 3.02E-04 | *_* | *_* |
| R2902 | 32 | F | *Z. caespitosa* | 177986 | 178017 | *Z. caespitosa* | 180754 | 180785 | -3 | 3.02E-04 | *_* | *_* |
| R2903 | 32 | F | *Z. caespitosa* | 84007 | 84038 | *Z. caespitosa* | 180754 | 180785 | -3 | 3.02E-04 | *_* | *_* |
| R2904 | 32 | F | *Z. caespitosa* | 37754 | 37785 | *Z. caespitosa* | 180754 | 180785 | -3 | 3.02E-04 | *_* | *_* |
| R2905 | 32 | F | *Z. caespitosa* | 4209 | 4240 | *Z. caespitosa* | 136946 | 136977 | -3 | 3.02E-04 | *_* | *_* |
| R2906 | 32 | F | *Z. caespitosa* | 4209 | 4240 | *Z. caespitosa* | 72632 | 72663 | -3 | 3.02E-04 | *_* | *_* |
| R2907 | 32 | F | *Z. caespitosa* | 4209 | 4240 | *Z. caespitosa* | 177986 | 178017 | -3 | 3.02E-04 | *_* | *_* |
| R2908 | 32 | F | *Z. caespitosa* | 4209 | 4240 | *Z. caespitosa* | 84007 | 84038 | -3 | 3.02E-04 | *_* | *_* |
| R2909 | 32 | F | *Z. caespitosa* | 4209 | 4240 | *Z. caespitosa* | 37754 | 37785 | -3 | 3.02E-04 | *_* | *_* |
| R2910 | 32 | F | *Z. caespitosa* | 83663 | 83694 | *Z. caespitosa* | 136946 | 136977 | -3 | 3.02E-04 | *_* | *_* |
| R2911 | 32 | F | *Z. caespitosa* | 72632 | 72663 | *Z. caespitosa* | 83663 | 83694 | -3 | 3.02E-04 | *_* | *_* |
| R2912 | 32 | F | *Z. caespitosa* | 83663 | 83694 | *Z. caespitosa* | 177986 | 178017 | -3 | 3.02E-04 | *_* | *_* |
| R2913 | 32 | F | *Z. caespitosa* | 83663 | 83694 | *Z. caespitosa* | 84007 | 84038 | -3 | 3.02E-04 | *_* | *_* |
| R2914 | 32 | F | *Z. caespitosa* | 37754 | 37785 | *Z. caespitosa* | 83663 | 83694 | -3 | 3.02E-04 | *_* | *_* |
| R2915 | 32 | F | *Z. caespitosa* | 4298 | 4329 | *Z. caespitosa* | 136946 | 136977 | -3 | 3.02E-04 | *_* | *_* |
| R2916 | 32 | F | *Z. caespitosa* | 4298 | 4329 | *Z. caespitosa* | 72632 | 72663 | -3 | 3.02E-04 | *_* | *_* |
| R2917 | 32 | F | *Z. caespitosa* | 4298 | 4329 | *Z. caespitosa* | 177986 | 178017 | -3 | 3.02E-04 | *_* | *_* |
| R2918 | 32 | F | *Z. caespitosa* | 4298 | 4329 | *Z. caespitosa* | 84007 | 84038 | -3 | 3.02E-04 | *_* | *_* |
| R2919 | 32 | F | *Z. caespitosa* | 4298 | 4329 | *Z. caespitosa* | 37754 | 37785 | -3 | 3.02E-04 | *_* | *_* |
| R2920 | 32 | F | *Z. caespitosa* | 136946 | 136977 | *Z. caespitosa* | 177731 | 177762 | -3 | 3.02E-04 | *_* | *_* |
| R2921 | 32 | F | *Z. caespitosa* | 72632 | 72663 | *Z. caespitosa* | 177731 | 177762 | -3 | 3.02E-04 | *_* | *_* |
| R2922 | 32 | F | *Z. caespitosa* | 177731 | 177762 | *Z. caespitosa* | 177986 | 178017 | -3 | 3.02E-04 | *_* | *_* |
| R2923 | 32 | F | *Z. caespitosa* | 84007 | 84038 | *Z. caespitosa* | 177731 | 177762 | -3 | 3.02E-04 | *_* | *_* |
| R2924 | 32 | F | *Z. caespitosa* | 37754 | 37785 | *Z. caespitosa* | 177731 | 177762 | -3 | 3.02E-04 | *_* | *_* |
| R2925 | 32 | F | *Z. caespitosa* | 83752 | 83783 | *Z. caespitosa* | 136946 | 136977 | -3 | 3.02E-04 | *_* | *_* |
| R2926 | 32 | F | *Z. caespitosa* | 72632 | 72663 | *Z. caespitosa* | 83752 | 83783 | -3 | 3.02E-04 | *_* | *_* |
| R2927 | 32 | F | *Z. caespitosa* | 83752 | 83783 | *Z. caespitosa* | 177986 | 178017 | -3 | 3.02E-04 | *_* | *_* |
| R2928 | 32 | F | *Z. caespitosa* | 83752 | 83783 | *Z. caespitosa* | 84007 | 84038 | -3 | 3.02E-04 | *_* | *_* |
| R2929 | 32 | F | *Z. caespitosa* | 37754 | 37785 | *Z. caespitosa* | 83752 | 83783 | -3 | 3.02E-04 | *_* | *_* |
| R2930 | 32 | F | *Z. caespitosa* | 4387 | 4418 | *Z. caespitosa* | 136946 | 136977 | -3 | 3.02E-04 | *_* | *_* |
| R2931 | 32 | F | *Z. caespitosa* | 4387 | 4418 | *Z. caespitosa* | 72632 | 72663 | -3 | 3.02E-04 | *_* | *_* |
| R2932 | 32 | F | *Z. caespitosa* | 4387 | 4418 | *Z. caespitosa* | 177986 | 178017 | -3 | 3.02E-04 | *_* | *_* |
| R2933 | 32 | F | *Z. caespitosa* | 4387 | 4418 | *Z. caespitosa* | 84007 | 84038 | -3 | 3.02E-04 | *_* | *_* |
| R2934 | 32 | F | *Z. caespitosa* | 4387 | 4418 | *Z. caespitosa* | 37754 | 37785 | -3 | 3.02E-04 | *_* | *_* |
| R2935 | 32 | F | *Z. caespitosa* | 177820 | 177851 | *Z. caespitosa* | 181651 | 181682 | -3 | 3.02E-04 | *_* | *orf103d* |
| R2936 | 32 | F | *Z. caespitosa* | 75921 | 75952 | *Z. caespitosa* | 177820 | 177851 | -3 | 3.02E-04 | *_* | *_* |
| R2937 | 32 | F | *Z. caespitosa* | 177820 | 177851 | *Z. caespitosa* | 184213 | 184244 | -3 | 3.02E-04 | *_* | *orf130b* |
| R2938 | 32 | F | *Z. caespitosa* | 83841 | 83872 | *Z. caespitosa* | 181651 | 181682 | -3 | 3.02E-04 | *_* | *orf103d* |
| R2939 | 32 | F | *Z. caespitosa* | 75921 | 75952 | *Z. caespitosa* | 83841 | 83872 | -3 | 3.02E-04 | *_* | *_* |
| R2940 | 32 | F | *Z. caespitosa* | 83841 | 83872 | *Z. caespitosa* | 184213 | 184244 | -3 | 3.02E-04 | *_* | *orf130b* |
| R2941 | 32 | F | *Z. caespitosa* | 4476 | 4507 | *Z. caespitosa* | 181651 | 181682 | -3 | 3.02E-04 | *_* | *orf103d* |
| R2942 | 32 | F | *Z. caespitosa* | 4476 | 4507 | *Z. caespitosa* | 75921 | 75952 | -3 | 3.02E-04 | *_* | *_* |
| R2943 | 32 | F | *Z. caespitosa* | 4476 | 4507 | *Z. caespitosa* | 184213 | 184244 | -3 | 3.02E-04 | *_* | *orf130b* |
| R2944 | 32 | F | *Z. caespitosa* | 180784 | 180815 | *Z. caespitosa* | 181651 | 181682 | -3 | 3.02E-04 | *_* | *orf103d* |
| R2945 | 32 | F | *Z. caespitosa* | 75921 | 75952 | *Z. caespitosa* | 180784 | 180815 | -3 | 3.02E-04 | *_* | *_* |
| R2946 | 32 | F | *Z. caespitosa* | 180784 | 180815 | *Z. caespitosa* | 184213 | 184244 | -3 | 3.02E-04 | *_* | *orf130b* |
| R2947 | 32 | F | *Z. caespitosa* | 4150 | 4181 | *Z. caespitosa* | 181651 | 181682 | -3 | 3.02E-04 | *_* | *orf103d* |
| R2948 | 32 | F | *Z. caespitosa* | 4150 | 4181 | *Z. caespitosa* | 75921 | 75952 | -3 | 3.02E-04 | *_* | *_* |
| R2949 | 32 | F | *Z. caespitosa* | 4150 | 4181 | *Z. caespitosa* | 184213 | 184244 | -3 | 3.02E-04 | *_* | *orf130b* |
| R2950 | 32 | F | *Z. caespitosa* | 83604 | 83635 | *Z. caespitosa* | 181651 | 181682 | -3 | 3.02E-04 | *_* | *orf103d* |
| R2951 | 32 | F | *Z. caespitosa* | 75921 | 75952 | *Z. caespitosa* | 83604 | 83635 | -3 | 3.02E-04 | *_* | *_* |
| R2952 | 32 | F | *Z. caespitosa* | 83604 | 83635 | *Z. caespitosa* | 184213 | 184244 | -3 | 3.02E-04 | *_* | *orf130b* |
| R2953 | 32 | F | *Z. caespitosa* | 4239 | 4270 | *Z. caespitosa* | 181651 | 181682 | -3 | 3.02E-04 | *_* | *orf103d* |
| R2954 | 32 | F | *Z. caespitosa* | 4239 | 4270 | *Z. caespitosa* | 75921 | 75952 | -3 | 3.02E-04 | *_* | *_* |
| R2955 | 32 | F | *Z. caespitosa* | 4239 | 4270 | *Z. caespitosa* | 184213 | 184244 | -3 | 3.02E-04 | *_* | *orf130b* |
| R2956 | 32 | F | *Z. caespitosa* | 83693 | 83724 | *Z. caespitosa* | 181651 | 181682 | -3 | 3.02E-04 | *_* | *orf103d* |
| R2957 | 32 | F | *Z. caespitosa* | 75921 | 75952 | *Z. caespitosa* | 83693 | 83724 | -3 | 3.02E-04 | *_* | *_* |
| R2958 | 32 | F | *Z. caespitosa* | 83693 | 83724 | *Z. caespitosa* | 184213 | 184244 | -3 | 3.02E-04 | *_* | *orf130b* |
| R2959 | 32 | F | *Z. caespitosa* | 4328 | 4359 | *Z. caespitosa* | 181651 | 181682 | -3 | 3.02E-04 | *_* | *orf103d* |
| R2960 | 32 | F | *Z. caespitosa* | 4328 | 4359 | *Z. caespitosa* | 75921 | 75952 | -3 | 3.02E-04 | *_* | *_* |
| R2961 | 32 | F | *Z. caespitosa* | 4328 | 4359 | *Z. caespitosa* | 184213 | 184244 | -3 | 3.02E-04 | *_* | *orf130b* |
| R2962 | 32 | F | *Z. caespitosa* | 177761 | 177792 | *Z. caespitosa* | 181651 | 181682 | -3 | 3.02E-04 | *_* | *orf103d* |
| R2963 | 32 | F | *Z. caespitosa* | 75921 | 75952 | *Z. caespitosa* | 177761 | 177792 | -3 | 3.02E-04 | *_* | *_* |
| R2964 | 32 | F | *Z. caespitosa* | 177761 | 177792 | *Z. caespitosa* | 184213 | 184244 | -3 | 3.02E-04 | *_* | *orf130b* |
| R2965 | 32 | F | *Z. caespitosa* | 83782 | 83813 | *Z. caespitosa* | 181651 | 181682 | -3 | 3.02E-04 | *_* | *orf103d* |
| R2966 | 32 | F | *Z. caespitosa* | 75921 | 75952 | *Z. caespitosa* | 83782 | 83813 | -3 | 3.02E-04 | *_* | *_* |
| R2967 | 32 | F | *Z. caespitosa* | 83782 | 83813 | *Z. caespitosa* | 184213 | 184244 | -3 | 3.02E-04 | *_* | *orf130b* |
| R2968 | 32 | F | *Z. caespitosa* | 4417 | 4448 | *Z. caespitosa* | 181651 | 181682 | -3 | 3.02E-04 | *_* | *orf103d* |
| R2969 | 32 | F | *Z. caespitosa* | 4417 | 4448 | *Z. caespitosa* | 75921 | 75952 | -3 | 3.02E-04 | *_* | *_* |
| R2970 | 32 | F | *Z. caespitosa* | 4417 | 4448 | *Z. caespitosa* | 184213 | 184244 | -3 | 3.02E-04 | *_* | *orf130b* |
| R2971 | 32 | F | *Z. caespitosa* | 51663 | 51694 | *Z. caespitosa* | 177581 | 177612 | -2 | 1.01E-05 | *orf106b;ccmFn* | *_* |
| R2972 | 32 | F | *Z. caespitosa* | 135046 | 135077 | *Z. caespitosa* | 148771 | 148802 | -3 | 3.02E-04 | *_* | *_* |
| R2973 | 32 | F | *Z. caespitosa* | 135046 | 135077 | *Z. caespitosa* | 148834 | 148865 | -3 | 3.02E-04 | *_* | *_* |
| R2974 | 32 | F | *Z. caespitosa* | 49992 | 50023 | *Z. caespitosa* | 83494 | 83525 | -1 | 2.16E-07 | *_* | *_* |
| R2975 | 32 | F | *Z. caespitosa* | 175159 | 175190 | *Z. caespitosa* | 177665 | 177696 | -3 | 3.02E-04 | *_* | *_* |
| R2976 | 32 | F | *Z. caespitosa* | 175159 | 175190 | *Z. caespitosa* | 180718 | 180749 | -3 | 3.02E-04 | *_* | *_* |
| R2977 | 32 | F | *Z. caespitosa* | 4173 | 4204 | *Z. caespitosa* | 175159 | 175190 | -3 | 3.02E-04 | *_* | *_* |
| R2978 | 32 | F | *Z. caespitosa* | 83627 | 83658 | *Z. caespitosa* | 175159 | 175190 | -3 | 3.02E-04 | *_* | *_* |
| R2979 | 32 | F | *Z. caespitosa* | 4262 | 4293 | *Z. caespitosa* | 175159 | 175190 | -3 | 3.02E-04 | *_* | *_* |
| R2980 | 32 | F | *Z. caespitosa* | 83716 | 83747 | *Z. caespitosa* | 175159 | 175190 | -3 | 3.02E-04 | *_* | *_* |
| R2981 | 32 | F | *Z. caespitosa* | 4351 | 4382 | *Z. caespitosa* | 175159 | 175190 | -3 | 3.02E-04 | *_* | *_* |
| R2982 | 32 | F | *Z. caespitosa* | 175159 | 175190 | *Z. caespitosa* | 177784 | 177815 | -3 | 3.02E-04 | *_* | *_* |
| R2983 | 32 | F | *Z. caespitosa* | 83805 | 83836 | *Z. caespitosa* | 175159 | 175190 | -3 | 3.02E-04 | *_* | *_* |
| R2984 | 32 | F | *Z. caespitosa* | 4440 | 4471 | *Z. caespitosa* | 175159 | 175190 | -3 | 3.02E-04 | *_* | *_* |
| R2985 | 32 | F | *Z. caespitosa* | 69958 | 69989 | *Z. caespitosa* | 101789 | 101820 | 0 | 2.25E-09 | *_* | *_* |
| R2986 | 32 | F | *Z. caespitosa* | 69958 | 69989 | *Z. caespitosa* | 74634 | 74665 | 0 | 2.25E-09 | *_* | *_* |
| R2987 | 32 | F | *Z. caespitosa* | 139975 | 140006 | *Z. caespitosa* | 172611 | 172642 | -1 | 2.16E-07 | *matR* | *orf185a* |
| R2988 | 32 | F | *Z. caespitosa* | 172563 | 172594 | *Z. caespitosa* | 172611 | 172642 | -3 | 3.02E-04 | *_* | *orf185a* |
| R2989 | 32 | F | *Z. caespitosa* | 77819 | 77850 | *Z. caespitosa* | 172611 | 172642 | -3 | 3.02E-04 | *_* | *orf185a* |
| R2990 | 32 | F | *Z. caespitosa* | 140132 | 140163 | *Z. caespitosa* | 172611 | 172642 | -3 | 3.02E-04 | *matR* | *orf185a* |
| R2991 | 32 | F | *Z. caespitosa* | 77757 | 77788 | *Z. caespitosa* | 172611 | 172642 | -3 | 3.02E-04 | *_* | *orf185a* |
| R2992 | 32 | F | *Z. caespitosa* | 77695 | 77726 | *Z. caespitosa* | 172611 | 172642 | -3 | 3.02E-04 | *_* | *orf185a* |
| R2993 | 32 | F | *Z. caespitosa* | 79477 | 79508 | *Z. caespitosa* | 172611 | 172642 | -3 | 3.02E-04 | *_* | *orf185a* |
| R2994 | 32 | F | *Z. caespitosa* | 9335 | 9366 | *Z. caespitosa* | 10584 | 10615 | -3 | 3.02E-04 | *orf397a* | *_* |
| R2995 | 32 | F | *Z. caespitosa* | 9271 | 9302 | *Z. caespitosa* | 10584 | 10615 | -3 | 3.02E-04 | *orf397a* | *_* |
| R2996 | 32 | F | *Z. caespitosa* | 9207 | 9238 | *Z. caespitosa* | 10584 | 10615 | -3 | 3.02E-04 | *orf397a* | *_* |
| R2997 | 32 | F | *Z. caespitosa* | 9463 | 9494 | *Z. caespitosa* | 10584 | 10615 | -3 | 3.02E-04 | *orf397a* | *_* |
| R2998 | 32 | F | *Z. caespitosa* | 9527 | 9558 | *Z. caespitosa* | 10584 | 10615 | -3 | 3.02E-04 | *orf397a* | *_* |
| R2999 | 32 | F | *Z. caespitosa* | 9591 | 9622 | *Z. caespitosa* | 10584 | 10615 | -3 | 3.02E-04 | *orf397a* | *_* |
| R3000 | 32 | F | *Z. caespitosa* | 9719 | 9750 | *Z. caespitosa* | 10584 | 10615 | -3 | 3.02E-04 | *orf397a* | *_* |
| R3001 | 32 | F | *Z. caespitosa* | 9783 | 9814 | *Z. caespitosa* | 10584 | 10615 | -3 | 3.02E-04 | *orf397a* | *_* |
| R3002 | 32 | F | *Z. caespitosa* | 41730 | 41761 | *Z. caespitosa* | 69206 | 69237 | -3 | 3.02E-04 | *_* | *orf118b* |
| R3003 | 32 | F | *Z. caespitosa* | 23235 | 23266 | *Z. caespitosa* | 184966 | 184997 | -2 | 1.01E-05 | *_* | *_* |
| R3004 | 32 | F | *Z. caespitosa* | 93965 | 93996 | *Z. caespitosa* | 184966 | 184997 | -2 | 1.01E-05 | *_* | *_* |
| R3005 | 32 | F | *Z. caespitosa* | 14561 | 14592 | *Z. caespitosa* | 23235 | 23266 | -2 | 1.01E-05 | *orf113a* | *_* |
| R3006 | 32 | F | *Z. caespitosa* | 14561 | 14592 | *Z. caespitosa* | 93965 | 93996 | -2 | 1.01E-05 | *orf113a* | *_* |
| R3007 | 32 | F | *Z. caespitosa* | 23235 | 23266 | *Z. caespitosa* | 118817 | 118848 | -2 | 1.01E-05 | *_* | *_* |
| R3008 | 32 | F | *Z. caespitosa* | 93965 | 93996 | *Z. caespitosa* | 118817 | 118848 | -2 | 1.01E-05 | *_* | *_* |
| R3009 | 32 | F | *Z. caespitosa* | 16065 | 16096 | *Z. caespitosa* | 23235 | 23266 | -2 | 1.01E-05 | *orf284a* | *_* |
| R3010 | 32 | F | *Z. caespitosa* | 16065 | 16096 | *Z. caespitosa* | 93965 | 93996 | -2 | 1.01E-05 | *orf284a* | *_* |
| R3011 | 32 | F | *Z. caespitosa* | 23235 | 23266 | *Z. caespitosa* | 83366 | 83397 | -2 | 1.01E-05 | *_* | *_* |
| R3012 | 32 | F | *Z. caespitosa* | 83366 | 83397 | *Z. caespitosa* | 93965 | 93996 | -2 | 1.01E-05 | *_* | *_* |
| R3013 | 32 | F | *Z. caespitosa* | 242 | 273 | *Z. caespitosa* | 112159 | 112190 | 0 | 2.25E-09 | *_* | *orf113c-1* |
| R3014 | 32 | F | *Z. caespitosa* | 24993 | 25024 | *Z. caespitosa* | 138729 | 138760 | 0 | 2.25E-09 | *_* | *_* |
| R3015 | 32 | F | *Z. caespitosa* | 95723 | 95754 | *Z. caespitosa* | 138729 | 138760 | 0 | 2.25E-09 | *_* | *_* |
| R3016 | 32 | F | *Z. caespitosa* | 138729 | 138760 | *Z. caespitosa* | 138929 | 138960 | 0 | 2.25E-09 | *_* | *_* |
| R3017 | 32 | F | *Z. caespitosa* | 68269 | 68300 | *Z. caespitosa* | 69931 | 69962 | 0 | 2.25E-09 | *nad9* | *_* |
| R3018 | 32 | F | *Z. caespitosa* | 69931 | 69962 | *Z. caespitosa* | 87381 | 87412 | 0 | 2.25E-09 | *_* | *orf122a* |
| R3019 | 32 | F | *Z. caespitosa* | 48413 | 48444 | *Z. caespitosa* | 69931 | 69962 | 0 | 2.25E-09 | *_* | *_* |
| R3020 | 32 | F | *Z. caespitosa* | 69931 | 69962 | *Z. caespitosa* | 74688 | 74719 | 0 | 2.25E-09 | *_* | *_* |
| R3021 | 32 | F | *Z. caespitosa* | 69931 | 69962 | *Z. caespitosa* | 172548 | 172579 | 0 | 2.25E-09 | *_* | *_* |
| R3022 | 32 | F | *Z. caespitosa* | 69931 | 69962 | *Z. caespitosa* | 77804 | 77835 | 0 | 2.25E-09 | *_* | *_* |
| R3023 | 32 | F | *Z. caespitosa* | 69931 | 69962 | *Z. caespitosa* | 140117 | 140148 | 0 | 2.25E-09 | *_* | *matR* |
| R3024 | 32 | F | *Z. caespitosa* | 69931 | 69962 | *Z. caespitosa* | 77742 | 77773 | 0 | 2.25E-09 | *_* | *_* |
| R3025 | 32 | F | *Z. caespitosa* | 69931 | 69962 | *Z. caespitosa* | 77680 | 77711 | 0 | 2.25E-09 | *_* | *_* |
| R3026 | 32 | F | *Z. caespitosa* | 69931 | 69962 | *Z. caespitosa* | 79462 | 79493 | 0 | 2.25E-09 | *_* | *_* |
| R3027 | 32 | F | *Z. caespitosa* | 16159 | 16190 | *Z. caespitosa* | 68269 | 68300 | 0 | 2.25E-09 | *orf284a* | *nad9* |
| R3028 | 32 | F | *Z. caespitosa* | 16159 | 16190 | *Z. caespitosa* | 87381 | 87412 | 0 | 2.25E-09 | *orf284a* | *orf122a* |
| R3029 | 32 | F | *Z. caespitosa* | 16159 | 16190 | *Z. caespitosa* | 48413 | 48444 | 0 | 2.25E-09 | *orf284a* | *_* |
| R3030 | 32 | F | *Z. caespitosa* | 16159 | 16190 | *Z. caespitosa* | 74688 | 74719 | 0 | 2.25E-09 | *orf284a* | *_* |
| R3031 | 32 | F | *Z. caespitosa* | 16159 | 16190 | *Z. caespitosa* | 172548 | 172579 | 0 | 2.25E-09 | *orf284a* | *_* |
| R3032 | 32 | F | *Z. caespitosa* | 16159 | 16190 | *Z. caespitosa* | 77804 | 77835 | 0 | 2.25E-09 | *orf284a* | *_* |
| R3033 | 32 | F | *Z. caespitosa* | 16159 | 16190 | *Z. caespitosa* | 140117 | 140148 | 0 | 2.25E-09 | *orf284a* | *matR* |
| R3034 | 32 | F | *Z. caespitosa* | 16159 | 16190 | *Z. caespitosa* | 77742 | 77773 | 0 | 2.25E-09 | *orf284a* | *_* |
| R3035 | 32 | F | *Z. caespitosa* | 16159 | 16190 | *Z. caespitosa* | 77680 | 77711 | 0 | 2.25E-09 | *orf284a* | *_* |
| R3036 | 32 | F | *Z. caespitosa* | 16159 | 16190 | *Z. caespitosa* | 79462 | 79493 | 0 | 2.25E-09 | *orf284a* | *_* |
| R3037 | 32 | F | *Z. caespitosa* | 68269 | 68300 | *Z. caespitosa* | 162792 | 162823 | 0 | 2.25E-09 | *nad9* | *_* |
| R3038 | 32 | F | *Z. caespitosa* | 87381 | 87412 | *Z. caespitosa* | 162792 | 162823 | 0 | 2.25E-09 | *orf122a* | *_* |
| R3039 | 32 | F | *Z. caespitosa* | 48413 | 48444 | *Z. caespitosa* | 162792 | 162823 | 0 | 2.25E-09 | *_* | *_* |
| R3040 | 32 | F | *Z. caespitosa* | 74688 | 74719 | *Z. caespitosa* | 162792 | 162823 | 0 | 2.25E-09 | *_* | *_* |
| R3041 | 32 | F | *Z. caespitosa* | 162792 | 162823 | *Z. caespitosa* | 172548 | 172579 | 0 | 2.25E-09 | *_* | *_* |
| R3042 | 32 | F | *Z. caespitosa* | 77804 | 77835 | *Z. caespitosa* | 162792 | 162823 | 0 | 2.25E-09 | *_* | *_* |
| R3043 | 32 | F | *Z. caespitosa* | 140117 | 140148 | *Z. caespitosa* | 162792 | 162823 | 0 | 2.25E-09 | *matR* | *_* |
| R3044 | 32 | F | *Z. caespitosa* | 77742 | 77773 | *Z. caespitosa* | 162792 | 162823 | 0 | 2.25E-09 | *_* | *_* |
| R3045 | 32 | F | *Z. caespitosa* | 77680 | 77711 | *Z. caespitosa* | 162792 | 162823 | 0 | 2.25E-09 | *_* | *_* |
| R3046 | 32 | F | *Z. caespitosa* | 79462 | 79493 | *Z. caespitosa* | 162792 | 162823 | 0 | 2.25E-09 | *_* | *_* |
| R3047 | 32 | F | *Z. caespitosa* | 131529 | 131560 | *Z. caespitosa* | 182602 | 182633 | -2 | 1.01E-05 | *orf131b* | *orf117c* |
| R3048 | 32 | F | *Z. caespitosa* | 80700 | 80731 | *Z. caespitosa* | 86576 | 86607 | -2 | 1.01E-05 | *_* | *_* |
| R3049 | 32 | F | *Z. caespitosa* | 15872 | 15903 | *Z. caespitosa* | 162878 | 162909 | 0 | 2.25E-09 | *_* | *_* |
| R3050 | 32 | F | *Z. caespitosa* | 136806 | 136837 | *Z. caespitosa* | 175089 | 175120 | -2 | 1.01E-05 | *_* | *_* |
| R3051 | 32 | F | *Z. caespitosa* | 27046 | 27077 | *Z. caespitosa* | 149185 | 149216 | 0 | 2.25E-09 | *_* | *_* |
| R3052 | 32 | F | *Z. caespitosa* | 48410 | 48441 | *Z. caespitosa* | 139957 | 139988 | -3 | 3.02E-04 | *_* | *matR* |
| R3053 | 32 | F | *Z. caespitosa* | 34934 | 34965 | *Z. caespitosa* | 75978 | 76009 | 0 | 2.25E-09 | *_* | *_* |
| R3054 | 32 | F | *Z. caespitosa* | 22159 | 22190 | *Z. caespitosa* | 109073 | 109104 | -2 | 1.01E-05 | *_* | *orf161a* |
| R3055 | 32 | F | *Z. caespitosa* | 33017 | 33048 | *Z. caespitosa* | 55111 | 55142 | -3 | 3.02E-04 | *_* | *_* |
| R3056 | 32 | F | *Z. caespitosa* | 16257 | 16288 | *Z. caespitosa* | 139929 | 139960 | 0 | 2.25E-09 | *orf284a* | *matR* |
| R3057 | 32 | F | *Z. caespitosa* | 16290 | 16321 | *Z. caespitosa* | 139929 | 139960 | 0 | 2.25E-09 | *orf284a* | *matR* |
| R3058 | 32 | F | *Z. caespitosa* | 16323 | 16354 | *Z. caespitosa* | 139929 | 139960 | 0 | 2.25E-09 | *orf284a* | *matR* |
| R3059 | 32 | F | *Z. caespitosa* | 16356 | 16387 | *Z. caespitosa* | 139929 | 139960 | 0 | 2.25E-09 | *orf284a* | *matR* |
| R3060 | 32 | F | *Z. caespitosa* | 16389 | 16420 | *Z. caespitosa* | 139929 | 139960 | 0 | 2.25E-09 | *orf284a* | *matR* |
| R3061 | 32 | F | *Z. caespitosa* | 16422 | 16453 | *Z. caespitosa* | 139929 | 139960 | 0 | 2.25E-09 | *orf284a* | *matR* |
| R3062 | 32 | F | *Z. caespitosa* | 16455 | 16486 | *Z. caespitosa* | 139929 | 139960 | 0 | 2.25E-09 | *orf284a* | *matR* |
| R3063 | 32 | F | *Z. caespitosa* | 16488 | 16519 | *Z. caespitosa* | 139929 | 139960 | 0 | 2.25E-09 | *orf284a* | *matR* |
| R3064 | 32 | F | *Z. caespitosa* | 16521 | 16552 | *Z. caespitosa* | 139929 | 139960 | 0 | 2.25E-09 | *orf284a* | *matR* |
| R3065 | 32 | F | *Z. caespitosa* | 16554 | 16585 | *Z. caespitosa* | 139929 | 139960 | 0 | 2.25E-09 | *orf284a* | *matR* |
| R3066 | 32 | F | *Z. caespitosa* | 16587 | 16618 | *Z. caespitosa* | 139929 | 139960 | 0 | 2.25E-09 | *orf284a* | *matR* |
| R3067 | 32 | F | *Z. caespitosa* | 16224 | 16255 | *Z. caespitosa* | 139929 | 139960 | 0 | 2.25E-09 | *orf284a* | *matR* |
| R3068 | 32 | F | *Z. caespitosa* | 28700 | 28731 | *Z. caespitosa* | 112421 | 112452 | -3 | 3.02E-04 | *_* | *orf113c-1* |
| R3069 | 32 | F | *Z. caespitosa* | 131538 | 131569 | *Z. caespitosa* | 182611 | 182642 | -3 | 3.02E-04 | *orf131b* | *orf117c* |
| R3070 | 32 | R | *Z. caespitosa* | 13294 | 13325 | *Z. caespitosa* | 13294 | 13325 | 0 | 2.25E-09 | *_* | *_* |
| R3071 | 32 | F | *Z. caespitosa* | 13293 | 13324 | *Z. caespitosa* | 13294 | 13325 | 0 | 2.25E-09 | *_* | *_* |
| R3072 | 32 | R | *Z. caespitosa* | 13293 | 13324 | *Z. caespitosa* | 13293 | 13324 | 0 | 2.25E-09 | *_* | *_* |
| R3073 | 32 | P | *Z. caespitosa* | 115991 | 116022 | *Z. caespitosa* | 184979 | 185010 | -1 | 2.16E-07 | *_* | *_* |
| R3074 | 32 | P | *Z. caespitosa* | 117759 | 117790 | *Z. caespitosa* | 184966 | 184997 | -2 | 1.01E-05 | *_* | *_* |
| R3075 | 32 | P | *Z. caespitosa* | 85128 | 85159 | *Z. caespitosa* | 184254 | 184285 | -2 | 1.01E-05 | *orf100a* | *orf130b* |
| R3076 | 32 | P | *Z. caespitosa* | 162792 | 162823 | *Z. caespitosa* | 184210 | 184241 | 0 | 2.25E-09 | *_* | *orf130b* |
| R3077 | 32 | P | *Z. caespitosa* | 16159 | 16190 | *Z. caespitosa* | 184210 | 184241 | 0 | 2.25E-09 | *orf284a* | *orf130b* |
| R3078 | 32 | P | *Z. caespitosa* | 69931 | 69962 | *Z. caespitosa* | 184210 | 184241 | 0 | 2.25E-09 | *_* | *orf130b* |
| R3079 | 32 | P | *Z. caespitosa* | 172611 | 172642 | *Z. caespitosa* | 184195 | 184226 | -3 | 3.02E-04 | *orf185a* | *orf130b* |
| R3080 | 32 | P | *Z. caespitosa* | 6384 | 6415 | *Z. caespitosa* | 183449 | 183480 | -3 | 3.02E-04 | *_* | *orf228a* |
| R3081 | 32 | P | *Z. caespitosa* | 162792 | 162823 | *Z. caespitosa* | 181648 | 181679 | 0 | 2.25E-09 | *_* | *orf103d* |
| R3082 | 32 | P | *Z. caespitosa* | 16159 | 16190 | *Z. caespitosa* | 181648 | 181679 | 0 | 2.25E-09 | *orf284a* | *orf103d* |
| R3083 | 32 | P | *Z. caespitosa* | 69931 | 69962 | *Z. caespitosa* | 181648 | 181679 | 0 | 2.25E-09 | *_* | *orf103d* |
| R3084 | 32 | P | *Z. caespitosa* | 172611 | 172642 | *Z. caespitosa* | 181633 | 181664 | -3 | 3.02E-04 | *orf185a* | *orf103d* |
| R3085 | 32 | P | *Z. caespitosa* | 79443 | 79474 | *Z. caespitosa* | 180800 | 180831 | -2 | 1.01E-05 | *_* | *_* |
| R3086 | 32 | P | *Z. caespitosa* | 79459 | 79490 | *Z. caespitosa* | 180784 | 180815 | -3 | 3.02E-04 | *_* | *_* |
| R3087 | 32 | P | *Z. caespitosa* | 77677 | 77708 | *Z. caespitosa* | 180784 | 180815 | -3 | 3.02E-04 | *_* | *_* |
| R3088 | 32 | P | *Z. caespitosa* | 77739 | 77770 | *Z. caespitosa* | 180784 | 180815 | -3 | 3.02E-04 | *_* | *_* |
| R3089 | 32 | P | *Z. caespitosa* | 140114 | 140145 | *Z. caespitosa* | 180784 | 180815 | -3 | 3.02E-04 | *matR* | *_* |
| R3090 | 32 | P | *Z. caespitosa* | 77801 | 77832 | *Z. caespitosa* | 180784 | 180815 | -3 | 3.02E-04 | *_* | *_* |
| R3091 | 32 | P | *Z. caespitosa* | 172545 | 172576 | *Z. caespitosa* | 180784 | 180815 | -3 | 3.02E-04 | *_* | *_* |
| R3092 | 32 | P | *Z. caespitosa* | 74685 | 74716 | *Z. caespitosa* | 180784 | 180815 | -3 | 3.02E-04 | *_* | *_* |
| R3093 | 32 | P | *Z. caespitosa* | 87378 | 87409 | *Z. caespitosa* | 180784 | 180815 | -3 | 3.02E-04 | *orf122a* | *_* |
| R3094 | 32 | P | *Z. caespitosa* | 79459 | 79490 | *Z. caespitosa* | 180754 | 180785 | -3 | 3.02E-04 | *_* | *_* |
| R3095 | 32 | P | *Z. caespitosa* | 77677 | 77708 | *Z. caespitosa* | 180754 | 180785 | -3 | 3.02E-04 | *_* | *_* |
| R3096 | 32 | P | *Z. caespitosa* | 77739 | 77770 | *Z. caespitosa* | 180754 | 180785 | -3 | 3.02E-04 | *_* | *_* |
| R3097 | 32 | P | *Z. caespitosa* | 140114 | 140145 | *Z. caespitosa* | 180754 | 180785 | -3 | 3.02E-04 | *matR* | *_* |
| R3098 | 32 | P | *Z. caespitosa* | 77801 | 77832 | *Z. caespitosa* | 180754 | 180785 | -3 | 3.02E-04 | *_* | *_* |
| R3099 | 32 | P | *Z. caespitosa* | 172545 | 172576 | *Z. caespitosa* | 180754 | 180785 | -3 | 3.02E-04 | *_* | *_* |
| R3100 | 32 | P | *Z. caespitosa* | 74685 | 74716 | *Z. caespitosa* | 180754 | 180785 | -3 | 3.02E-04 | *_* | *_* |
| R3101 | 32 | P | *Z. caespitosa* | 87378 | 87409 | *Z. caespitosa* | 180754 | 180785 | -3 | 3.02E-04 | *orf122a* | *_* |
| R3102 | 32 | P | *Z. caespitosa* | 79443 | 79474 | *Z. caespitosa* | 180711 | 180742 | -2 | 1.01E-05 | *_* | *_* |
| R3103 | 32 | P | *Z. caespitosa* | 180034 | 180065 | *Z. caespitosa* | 180647 | 180678 | 0 | 2.25E-09 | *_* | *_* |
| R3104 | 32 | P | *Z. caespitosa* | 74746 | 74777 | *Z. caespitosa* | 180590 | 180621 | -2 | 1.01E-05 | *_* | *_* |
| R3105 | 32 | P | *Z. caespitosa* | 16631 | 16662 | *Z. caespitosa* | 180590 | 180621 | -2 | 1.01E-05 | *orf284a* | *_* |
| R3106 | 32 | P | *Z. caespitosa* | 72659 | 72690 | *Z. caespitosa* | 179868 | 179899 | -3 | 3.02E-04 | *_* | *_* |
| R3107 | 32 | P | *Z. caespitosa* | 162792 | 162823 | *Z. caespitosa* | 177983 | 178014 | 0 | 2.25E-09 | *_* | *_* |
| R3108 | 32 | P | *Z. caespitosa* | 16159 | 16190 | *Z. caespitosa* | 177983 | 178014 | 0 | 2.25E-09 | *orf284a* | *_* |
| R3109 | 32 | P | *Z. caespitosa* | 69931 | 69962 | *Z. caespitosa* | 177983 | 178014 | 0 | 2.25E-09 | *_* | *_* |
| R3110 | 32 | P | *Z. caespitosa* | 172611 | 172642 | *Z. caespitosa* | 177968 | 177999 | -3 | 3.02E-04 | *orf185a* | *_* |
| R3111 | 32 | P | *Z. caespitosa* | 139775 | 139806 | *Z. caespitosa* | 177860 | 177891 | -2 | 1.01E-05 | *matR* | *_* |
| R3112 | 32 | P | *Z. caespitosa* | 79443 | 79474 | *Z. caespitosa* | 177836 | 177867 | -2 | 1.01E-05 | *_* | *_* |
| R3113 | 32 | P | *Z. caespitosa* | 79459 | 79490 | *Z. caespitosa* | 177820 | 177851 | -3 | 3.02E-04 | *_* | *_* |
| R3114 | 32 | P | *Z. caespitosa* | 77677 | 77708 | *Z. caespitosa* | 177820 | 177851 | -3 | 3.02E-04 | *_* | *_* |
| R3115 | 32 | P | *Z. caespitosa* | 77739 | 77770 | *Z. caespitosa* | 177820 | 177851 | -3 | 3.02E-04 | *_* | *_* |
| R3116 | 32 | P | *Z. caespitosa* | 140114 | 140145 | *Z. caespitosa* | 177820 | 177851 | -3 | 3.02E-04 | *matR* | *_* |
| R3117 | 32 | P | *Z. caespitosa* | 77801 | 77832 | *Z. caespitosa* | 177820 | 177851 | -3 | 3.02E-04 | *_* | *_* |
| R3118 | 32 | P | *Z. caespitosa* | 172545 | 172576 | *Z. caespitosa* | 177820 | 177851 | -3 | 3.02E-04 | *_* | *_* |
| R3119 | 32 | P | *Z. caespitosa* | 74685 | 74716 | *Z. caespitosa* | 177820 | 177851 | -3 | 3.02E-04 | *_* | *_* |
| R3120 | 32 | P | *Z. caespitosa* | 87378 | 87409 | *Z. caespitosa* | 177820 | 177851 | -3 | 3.02E-04 | *orf122a* | *_* |
| R3121 | 32 | P | *Z. caespitosa* | 79443 | 79474 | *Z. caespitosa* | 177777 | 177808 | -2 | 1.01E-05 | *_* | *_* |
| R3122 | 32 | P | *Z. caespitosa* | 79459 | 79490 | *Z. caespitosa* | 177761 | 177792 | -3 | 3.02E-04 | *_* | *_* |
| R3123 | 32 | P | *Z. caespitosa* | 77677 | 77708 | *Z. caespitosa* | 177761 | 177792 | -3 | 3.02E-04 | *_* | *_* |
| R3124 | 32 | P | *Z. caespitosa* | 77739 | 77770 | *Z. caespitosa* | 177761 | 177792 | -3 | 3.02E-04 | *_* | *_* |
| R3125 | 32 | P | *Z. caespitosa* | 140114 | 140145 | *Z. caespitosa* | 177761 | 177792 | -3 | 3.02E-04 | *matR* | *_* |
| R3126 | 32 | P | *Z. caespitosa* | 77801 | 77832 | *Z. caespitosa* | 177761 | 177792 | -3 | 3.02E-04 | *_* | *_* |
| R3127 | 32 | P | *Z. caespitosa* | 172545 | 172576 | *Z. caespitosa* | 177761 | 177792 | -3 | 3.02E-04 | *_* | *_* |
| R3128 | 32 | P | *Z. caespitosa* | 74685 | 74716 | *Z. caespitosa* | 177761 | 177792 | -3 | 3.02E-04 | *_* | *_* |
| R3129 | 32 | P | *Z. caespitosa* | 87378 | 87409 | *Z. caespitosa* | 177761 | 177792 | -3 | 3.02E-04 | *orf122a* | *_* |
| R3130 | 32 | P | *Z. caespitosa* | 79459 | 79490 | *Z. caespitosa* | 177731 | 177762 | -3 | 3.02E-04 | *_* | *_* |
| R3131 | 32 | P | *Z. caespitosa* | 77677 | 77708 | *Z. caespitosa* | 177731 | 177762 | -3 | 3.02E-04 | *_* | *_* |
| R3132 | 32 | P | *Z. caespitosa* | 77739 | 77770 | *Z. caespitosa* | 177731 | 177762 | -3 | 3.02E-04 | *_* | *_* |
| R3133 | 32 | P | *Z. caespitosa* | 140114 | 140145 | *Z. caespitosa* | 177731 | 177762 | -3 | 3.02E-04 | *matR* | *_* |
| R3134 | 32 | P | *Z. caespitosa* | 77801 | 77832 | *Z. caespitosa* | 177731 | 177762 | -3 | 3.02E-04 | *_* | *_* |
| R3135 | 32 | P | *Z. caespitosa* | 172545 | 172576 | *Z. caespitosa* | 177731 | 177762 | -3 | 3.02E-04 | *_* | *_* |
| R3136 | 32 | P | *Z. caespitosa* | 74685 | 74716 | *Z. caespitosa* | 177731 | 177762 | -3 | 3.02E-04 | *_* | *_* |
| R3137 | 32 | P | *Z. caespitosa* | 87378 | 87409 | *Z. caespitosa* | 177731 | 177762 | -3 | 3.02E-04 | *orf122a* | *_* |
| R3138 | 32 | P | *Z. caespitosa* | 79459 | 79490 | *Z. caespitosa* | 177701 | 177732 | -3 | 3.02E-04 | *_* | *_* |
| R3139 | 32 | P | *Z. caespitosa* | 77677 | 77708 | *Z. caespitosa* | 177701 | 177732 | -3 | 3.02E-04 | *_* | *_* |
| R3140 | 32 | P | *Z. caespitosa* | 77739 | 77770 | *Z. caespitosa* | 177701 | 177732 | -3 | 3.02E-04 | *_* | *_* |
| R3141 | 32 | P | *Z. caespitosa* | 140114 | 140145 | *Z. caespitosa* | 177701 | 177732 | -3 | 3.02E-04 | *matR* | *_* |
| R3142 | 32 | P | *Z. caespitosa* | 77801 | 77832 | *Z. caespitosa* | 177701 | 177732 | -3 | 3.02E-04 | *_* | *_* |
| R3143 | 32 | P | *Z. caespitosa* | 172545 | 172576 | *Z. caespitosa* | 177701 | 177732 | -3 | 3.02E-04 | *_* | *_* |
| R3144 | 32 | P | *Z. caespitosa* | 74685 | 74716 | *Z. caespitosa* | 177701 | 177732 | -3 | 3.02E-04 | *_* | *_* |
| R3145 | 32 | P | *Z. caespitosa* | 87378 | 87409 | *Z. caespitosa* | 177701 | 177732 | -3 | 3.02E-04 | *orf122a* | *_* |
| R3146 | 32 | P | *Z. caespitosa* | 79443 | 79474 | *Z. caespitosa* | 177658 | 177689 | -2 | 1.01E-05 | *_* | *_* |
| R3147 | 32 | P | *Z. caespitosa* | 162534 | 162565 | *Z. caespitosa* | 175166 | 175197 | -3 | 3.02E-04 | *_* | *_* |
| R3148 | 32 | P | *Z. caespitosa* | 27046 | 27077 | *Z. caespitosa* | 175089 | 175120 | -2 | 1.01E-05 | *_* | *_* |
| R3149 | 32 | P | *Z. caespitosa* | 76041 | 76072 | *Z. caespitosa* | 174031 | 174062 | 0 | 2.25E-09 | *_* | *_* |
| R3150 | 32 | P | *Z. caespitosa* | 109073 | 109104 | *Z. caespitosa* | 172888 | 172919 | -2 | 1.01E-05 | *orf161a* | *orf185a* |
| R3151 | 32 | P | *Z. caespitosa* | 75903 | 75934 | *Z. caespitosa* | 172611 | 172642 | -3 | 3.02E-04 | *_* | *orf185a* |
| R3152 | 32 | P | *Z. caespitosa* | 136928 | 136959 | *Z. caespitosa* | 172611 | 172642 | -3 | 3.02E-04 | *_* | *orf185a* |
| R3153 | 32 | P | *Z. caespitosa* | 72614 | 72645 | *Z. caespitosa* | 172611 | 172642 | -3 | 3.02E-04 | *_* | *orf185a* |
| R3154 | 32 | P | *Z. caespitosa* | 83989 | 84020 | *Z. caespitosa* | 172611 | 172642 | -3 | 3.02E-04 | *_* | *orf185a* |
| R3155 | 32 | P | *Z. caespitosa* | 75855 | 75886 | *Z. caespitosa* | 172563 | 172594 | -3 | 3.02E-04 | *_* | *_* |
| R3156 | 32 | P | *Z. caespitosa* | 109088 | 109119 | *Z. caespitosa* | 172578 | 172609 | -3 | 3.02E-04 | *orf161a* | *orf185a* |
| R3157 | 32 | P | *Z. caespitosa* | 57585 | 57616 | *Z. caespitosa* | 172548 | 172579 | 0 | 2.25E-09 | *orf117a* | *_* |
| R3158 | 32 | P | *Z. caespitosa* | 52802 | 52833 | *Z. caespitosa* | 172548 | 172579 | 0 | 2.25E-09 | *orf172a* | *_* |
| R3159 | 32 | P | *Z. caespitosa* | 4417 | 4448 | *Z. caespitosa* | 172545 | 172576 | -3 | 3.02E-04 | *_* | *_* |
| R3160 | 32 | P | *Z. caespitosa* | 83782 | 83813 | *Z. caespitosa* | 172545 | 172576 | -3 | 3.02E-04 | *_* | *_* |
| R3161 | 32 | P | *Z. caespitosa* | 4328 | 4359 | *Z. caespitosa* | 172545 | 172576 | -3 | 3.02E-04 | *_* | *_* |
| R3162 | 32 | P | *Z. caespitosa* | 83693 | 83724 | *Z. caespitosa* | 172545 | 172576 | -3 | 3.02E-04 | *_* | *_* |
| R3163 | 32 | P | *Z. caespitosa* | 4239 | 4270 | *Z. caespitosa* | 172545 | 172576 | -3 | 3.02E-04 | *_* | *_* |
| R3164 | 32 | P | *Z. caespitosa* | 83604 | 83635 | *Z. caespitosa* | 172545 | 172576 | -3 | 3.02E-04 | *_* | *_* |
| R3165 | 32 | P | *Z. caespitosa* | 4150 | 4181 | *Z. caespitosa* | 172545 | 172576 | -3 | 3.02E-04 | *_* | *_* |
| R3166 | 32 | P | *Z. caespitosa* | 4476 | 4507 | *Z. caespitosa* | 172545 | 172576 | -3 | 3.02E-04 | *_* | *_* |
| R3167 | 32 | P | *Z. caespitosa* | 83841 | 83872 | *Z. caespitosa* | 172545 | 172576 | -3 | 3.02E-04 | *_* | *_* |
| R3168 | 32 | P | *Z. caespitosa* | 148618 | 148649 | *Z. caespitosa* | 168277 | 168308 | -3 | 3.02E-04 | *_* | *_* |
| R3169 | 32 | P | *Z. caespitosa* | 57523 | 57554 | *Z. caespitosa* | 164762 | 164793 | -3 | 3.02E-04 | *orf117a* | *_* |
| R3170 | 32 | P | *Z. caespitosa* | 4518 | 4549 | *Z. caespitosa* | 162878 | 162909 | 0 | 2.25E-09 | *_* | *_* |
| R3171 | 32 | P | *Z. caespitosa* | 108851 | 108882 | *Z. caespitosa* | 162876 | 162907 | 0 | 2.25E-09 | *orf161a* | *_* |
| R3172 | 32 | P | *Z. caespitosa* | 108699 | 108730 | *Z. caespitosa* | 162792 | 162823 | 0 | 2.25E-09 | *orf161a* | *_* |
| R3173 | 32 | P | *Z. caespitosa* | 75918 | 75949 | *Z. caespitosa* | 162792 | 162823 | 0 | 2.25E-09 | *_* | *_* |
| R3174 | 32 | P | *Z. caespitosa* | 136943 | 136974 | *Z. caespitosa* | 162792 | 162823 | 0 | 2.25E-09 | *_* | *_* |
| R3175 | 32 | P | *Z. caespitosa* | 72629 | 72660 | *Z. caespitosa* | 162792 | 162823 | 0 | 2.25E-09 | *_* | *_* |
| R3176 | 32 | P | *Z. caespitosa* | 84004 | 84035 | *Z. caespitosa* | 162792 | 162823 | 0 | 2.25E-09 | *_* | *_* |
| R3177 | 32 | P | *Z. caespitosa* | 37751 | 37782 | *Z. caespitosa* | 162792 | 162823 | 0 | 2.25E-09 | *_* | *_* |
| R3178 | 32 | P | *Z. caespitosa* | 137102 | 137133 | *Z. caespitosa* | 162534 | 162565 | -2 | 1.01E-05 | *_* | *_* |
| R3179 | 32 | P | *Z. caespitosa* | 68269 | 68300 | *Z. caespitosa* | 156617 | 156648 | 0 | 2.25E-09 | *nad9* | *_* |
| R3180 | 32 | P | *Z. caespitosa* | 87381 | 87412 | *Z. caespitosa* | 156617 | 156648 | 0 | 2.25E-09 | *orf122a* | *_* |
| R3181 | 32 | P | *Z. caespitosa* | 48413 | 48444 | *Z. caespitosa* | 156617 | 156648 | 0 | 2.25E-09 | *_* | *_* |
| R3182 | 32 | P | *Z. caespitosa* | 112427 | 112458 | *Z. caespitosa* | 150982 | 151013 | -3 | 3.02E-04 | *orf113c-1* | *orf132a* |
| R3183 | 32 | P | *Z. caespitosa* | 50028 | 50059 | *Z. caespitosa* | 149205 | 149236 | -1 | 2.16E-07 | *_* | *_* |
| R3184 | 32 | P | *Z. caespitosa* | 136806 | 136837 | *Z. caespitosa* | 149185 | 149216 | 0 | 2.25E-09 | *_* | *_* |
| R3185 | 32 | P | *Z. caespitosa* | 75855 | 75886 | *Z. caespitosa* | 140132 | 140163 | -3 | 3.02E-04 | *_* | *matR* |
| R3186 | 32 | P | *Z. caespitosa* | 57585 | 57616 | *Z. caespitosa* | 140117 | 140148 | 0 | 2.25E-09 | *orf117a* | *matR* |
| R3187 | 32 | P | *Z. caespitosa* | 52802 | 52833 | *Z. caespitosa* | 140117 | 140148 | 0 | 2.25E-09 | *orf172a* | *matR* |
| R3188 | 32 | P | *Z. caespitosa* | 4417 | 4448 | *Z. caespitosa* | 140114 | 140145 | -3 | 3.02E-04 | *_* | *matR* |
| R3189 | 32 | P | *Z. caespitosa* | 83782 | 83813 | *Z. caespitosa* | 140114 | 140145 | -3 | 3.02E-04 | *_* | *matR* |
| R3190 | 32 | P | *Z. caespitosa* | 4328 | 4359 | *Z. caespitosa* | 140114 | 140145 | -3 | 3.02E-04 | *_* | *matR* |
| R3191 | 32 | P | *Z. caespitosa* | 83693 | 83724 | *Z. caespitosa* | 140114 | 140145 | -3 | 3.02E-04 | *_* | *matR* |
| R3192 | 32 | P | *Z. caespitosa* | 4239 | 4270 | *Z. caespitosa* | 140114 | 140145 | -3 | 3.02E-04 | *_* | *matR* |
| R3193 | 32 | P | *Z. caespitosa* | 83604 | 83635 | *Z. caespitosa* | 140114 | 140145 | -3 | 3.02E-04 | *_* | *matR* |
| R3194 | 32 | P | *Z. caespitosa* | 4150 | 4181 | *Z. caespitosa* | 140114 | 140145 | -3 | 3.02E-04 | *_* | *matR* |
| R3195 | 32 | P | *Z. caespitosa* | 4476 | 4507 | *Z. caespitosa* | 140114 | 140145 | -3 | 3.02E-04 | *_* | *matR* |
| R3196 | 32 | P | *Z. caespitosa* | 83841 | 83872 | *Z. caespitosa* | 140114 | 140145 | -3 | 3.02E-04 | *_* | *matR* |
| R3197 | 32 | P | *Z. caespitosa* | 4387 | 4418 | *Z. caespitosa* | 140114 | 140145 | -3 | 3.02E-04 | *_* | *matR* |
| R3198 | 32 | P | *Z. caespitosa* | 83752 | 83783 | *Z. caespitosa* | 140114 | 140145 | -3 | 3.02E-04 | *_* | *matR* |
| R3199 | 32 | P | *Z. caespitosa* | 4298 | 4329 | *Z. caespitosa* | 140114 | 140145 | -3 | 3.02E-04 | *_* | *matR* |
| R3200 | 32 | P | *Z. caespitosa* | 83663 | 83694 | *Z. caespitosa* | 140114 | 140145 | -3 | 3.02E-04 | *_* | *matR* |
| R3201 | 32 | P | *Z. caespitosa* | 4209 | 4240 | *Z. caespitosa* | 140114 | 140145 | -3 | 3.02E-04 | *_* | *matR* |
| R3202 | 32 | P | *Z. caespitosa* | 46096 | 46127 | *Z. caespitosa* | 140114 | 140145 | -3 | 3.02E-04 | *_* | *matR* |
| R3203 | 32 | P | *Z. caespitosa* | 75855 | 75886 | *Z. caespitosa* | 139975 | 140006 | -1 | 2.16E-07 | *_* | *matR* |
| R3204 | 32 | P | *Z. caespitosa* | 4516 | 4547 | *Z. caespitosa* | 139775 | 139806 | -2 | 1.01E-05 | *_* | *matR* |
| R3205 | 32 | P | *Z. caespitosa* | 83881 | 83912 | *Z. caespitosa* | 139775 | 139806 | -2 | 1.01E-05 | *_* | *matR* |
| R3206 | 32 | P | *Z. caespitosa* | 109073 | 109104 | *Z. caespitosa* | 138944 | 138975 | -1 | 2.16E-07 | *orf161a* | *_* |
| R3207 | 32 | P | *Z. caespitosa* | 55111 | 55142 | *Z. caespitosa* | 138944 | 138975 | -3 | 3.02E-04 | *_* | *_* |
| R3208 | 32 | P | *Z. caespitosa* | 116001 | 116032 | *Z. caespitosa* | 138729 | 138760 | 0 | 2.25E-09 | *_* | *_* |
| R3209 | 32 | P | *Z. caespitosa* | 57690 | 57721 | *Z. caespitosa* | 138617 | 138648 | -2 | 1.01E-05 | *orf117a* | *_* |
| R3210 | 32 | P | *Z. caespitosa* | 116101 | 116132 | *Z. caespitosa* | 138617 | 138648 | -3 | 3.02E-04 | *_* | *_* |
| R3211 | 32 | P | *Z. caespitosa* | 7574 | 7605 | *Z. caespitosa* | 137110 | 137141 | 0 | 2.25E-09 | *_* | *_* |
| R3212 | 32 | P | *Z. caespitosa* | 15941 | 15972 | *Z. caespitosa* | 137110 | 137141 | 0 | 2.25E-09 | *orf284a* | *_* |
| R3213 | 32 | P | *Z. caespitosa* | 16159 | 16190 | *Z. caespitosa* | 136943 | 136974 | 0 | 2.25E-09 | *orf284a* | *_* |
| R3214 | 32 | P | *Z. caespitosa* | 69931 | 69962 | *Z. caespitosa* | 136943 | 136974 | 0 | 2.25E-09 | *_* | *_* |
| R3215 | 32 | P | *Z. caespitosa* | 72182 | 72213 | *Z. caespitosa* | 135171 | 135202 | -1 | 2.16E-07 | *_* | *_* |
| R3216 | 32 | P | *Z. caespitosa* | 15872 | 15903 | *Z. caespitosa* | 135169 | 135200 | 0 | 2.25E-09 | *_* | *_* |
| R3217 | 32 | P | *Z. caespitosa* | 5519 | 5550 | *Z. caespitosa* | 129455 | 129486 | -2 | 1.01E-05 | *_* | *ccmC* |
| R3218 | 32 | P | *Z. caespitosa* | 30008 | 30039 | *Z. caespitosa* | 126405 | 126436 | -3 | 3.02E-04 | *_* | *_* |
| R3219 | 32 | P | *Z. caespitosa* | 109073 | 109104 | *Z. caespitosa* | 118835 | 118866 | -2 | 1.01E-05 | *orf161a* | *_* |
| R3220 | 32 | P | *Z. caespitosa* | 117759 | 117790 | *Z. caespitosa* | 118817 | 118848 | -2 | 1.01E-05 | *_* | *_* |
| R3221 | 32 | P | *Z. caespitosa* | 83366 | 83397 | *Z. caespitosa* | 117759 | 117790 | -2 | 1.01E-05 | *_* | *_* |
| R3222 | 32 | P | *Z. caespitosa* | 16065 | 16096 | *Z. caespitosa* | 117759 | 117790 | -2 | 1.01E-05 | *orf284a* | *_* |
| R3223 | 32 | P | *Z. caespitosa* | 14561 | 14592 | *Z. caespitosa* | 117759 | 117790 | -2 | 1.01E-05 | *orf113a* | *_* |
| R3224 | 32 | P | *Z. caespitosa* | 74746 | 74777 | *Z. caespitosa* | 115987 | 116018 | -2 | 1.01E-05 | *_* | *_* |
| R3225 | 32 | P | *Z. caespitosa* | 16631 | 16662 | *Z. caespitosa* | 115987 | 116018 | -2 | 1.01E-05 | *orf284a* | *_* |
| R3226 | 32 | P | *Z. caespitosa* | 77834 | 77865 | *Z. caespitosa* | 109088 | 109119 | -3 | 3.02E-04 | *_* | *orf161a* |
| R3227 | 32 | P | *Z. caespitosa* | 79492 | 79523 | *Z. caespitosa* | 109088 | 109119 | -3 | 3.02E-04 | *_* | *orf161a* |
| R3228 | 32 | P | *Z. caespitosa* | 77619 | 77650 | *Z. caespitosa* | 109074 | 109105 | -3 | 3.02E-04 | *_* | *orf161a* |
| R3229 | 32 | P | *Z. caespitosa* | 16159 | 16190 | *Z. caespitosa* | 108699 | 108730 | 0 | 2.25E-09 | *orf284a* | *orf161a* |
| R3230 | 32 | P | *Z. caespitosa* | 69931 | 69962 | *Z. caespitosa* | 108699 | 108730 | 0 | 2.25E-09 | *_* | *orf161a* |
| R3231 | 32 | P | *Z. caespitosa* | 52646 | 52677 | *Z. caespitosa* | 101794 | 101825 | 0 | 2.25E-09 | *orf172a* | *_* |
| R3232 | 32 | P | *Z. caespitosa* | 57690 | 57721 | *Z. caespitosa* | 101641 | 101672 | -2 | 1.01E-05 | *orf117a* | *_* |
| R3233 | 32 | P | *Z. caespitosa* | 74746 | 74777 | *Z. caespitosa* | 100040 | 100071 | -2 | 1.01E-05 | *_* | *_* |
| R3234 | 32 | P | *Z. caespitosa* | 16631 | 16662 | *Z. caespitosa* | 100040 | 100071 | -2 | 1.01E-05 | *orf284a* | *_* |
| R3235 | 32 | P | *Z. caespitosa* | 242 | 273 | *Z. caespitosa* | 99566 | 99597 | 0 | 2.25E-09 | *_* | *orf113c-2* |
| R3236 | 32 | P | *Z. caespitosa* | 28700 | 28731 | *Z. caespitosa* | 99304 | 99335 | -3 | 3.02E-04 | *_* | *orf113c-2* |
| R3237 | 32 | P | *Z. caespitosa* | 22177 | 22208 | *Z. caespitosa* | 93965 | 93996 | -2 | 1.01E-05 | *_* | *_* |
| R3238 | 32 | P | *Z. caespitosa* | 53065 | 53096 | *Z. caespitosa* | 93965 | 93996 | -2 | 1.01E-05 | *_* | *_* |
[truncated: 265,321 more chars]
